# Ceramide‐mediated mitochondrial dysfunction in nonobese nonalcoholic fatty liver disease: A regulatory role for serine palmitoyltransferase subunit 2

**DOI:** 10.1002/ccs3.70091

**Published:** 2026-06-25

**Authors:** Li‐Jun Xue, Guang‐Yan Yao, Fei‐Fei Fan, Kai‐Min Li, Wen‐Ming Wu

**Affiliations:** ^1^ Department of Gastroenterology Jinan Central Hospital Affiliated to Shandong First Medical University Jinan Shandong China; ^2^ Department of Gastroenterology and Hepatology The 960(th) Hospital of Joint Logistics Support Force PLA Jinan Shandong China; ^3^ Department of Gastroenterology China‐Japan Friendship Hospital Beijing China

**Keywords:** ceramide, mitochondrial dysfunction, non‐obese NAFLD, precision therapy, Sptlc2

## Abstract

This study investigated the pathological relevance of the serine palmitoyltransferase long chain base subunit 2 (Sptlc2)–ceramide axis in nonobese nonalcoholic fatty liver disease (NAFLD), focusing on hepatic steatosis, inflammation, oxidative stress, and mitochondrial dysfunction. A nonobese NAFLD rat model was established using a high‐temperature dry‐fried soybean diet. Integrated liquid chromatography‐mass spectrometry‐based proteomic and metabolomic analyses were used to identify candidate pathways. Sptlc2 function was validated by AAV2/8‐mediated liver‐directed knockdown in vivo, lentiviral knockdown in primary hepatocytes, and C2‐ceramide rescue experiments. Multi‐omics profiling identified Sptlc2 as a sphingolipid metabolism‐related candidate in the model. Sptlc2 knockdown reduced long‐chain ceramide accumulation, hepatic lipid deposition, inflammatory cytokine expression, oxidative stress, and hepatocyte injury. In primary hepatocytes, Sptlc2 silencing improved mitochondrial respiration, membrane potential, calcium and reactive oxygen species homeostasis, and mitochondrial ultrastructure. These protective effects were partially reversed by C2‐ceramide in vitro and in vivo. The Sptlc2–ceramide axis contributes to ceramide accumulation, hepatic lipotoxicity, inflammatory activation, and mitochondrial dysfunction in this nonobese NAFLD model, suggesting its potential relevance as a therapeutic target for further investigation.

## Graphical abstract

Serine palmitoyltransferase long chain base subunit 2 (Sptlc2) promotes ceramide‐associated mitochondrial dysfunction in nonobese nonalcoholic fatty liver disease (NAFLD). A dry‐fried soybean‐induced nonobese NAFLD model shows Sptlc2 upregulation, ceramide accumulation, and mitochondrial dysfunction. Sptlc2 knockdown reduces ceramides and ameliorates steatosis, inflammation, and apoptosis, whereas exogenous C2‐ceramide partially reverses these effects.

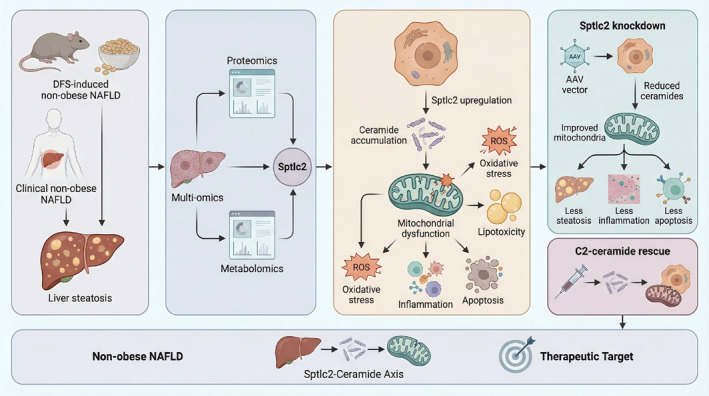

## 1 INTRODUCTION

Nonobese nonalcoholic fatty liver disease (NAFLD) has emerged as an increasingly recognized clinical phenotype, particularly in Asian populations, in which hepatic steatosis develops despite a body mass index within the nonoverweight range.^1^, ^2^ Although these individuals do not exhibit overt obesity, they may still show steatosis, inflammation and fibrotic progression comparable to those observed in obese NAFLD.^3^, ^4^ Because conventional metabolic risk profiles are often less apparent, nonobese NAFLD may be under‐recognized in clinical practice.^5^ These observations suggest that pathogenic drivers within this phenotype require further clarification. At present, however, the molecular basis of lipotoxic injury in nonobese NAFLD remains incompletely defined, and the extent to which it overlaps with or differs from obese NAFLD has not been directly established.^3^, ^6^

Lipotoxicity is a central process in NAFLD progression and is closely linked to the hepatic accumulation of free fatty acids (FFAs) and their bioactive lipid derivatives.^7^, ^8^ This process contributes to oxidative stress, organelle dysfunction, inflammatory activation and hepatocyte death.^9^, ^10^, ^11^ Among lipotoxic mediators, ceramides have attracted particular attention because they are implicated in insulin resistance, inflammatory signaling, and mitochondrial injury.^8^, ^12^, ^13^ However, although ceramide accumulation has been documented in NAFLD more broadly, direct evidence remains limited regarding whether dysregulated ceramide synthesis is a major driver of pathological changes in nonobese NAFLD, and whether this process is linked to a defined molecular axis in this setting.^14^

In the de novo ceramide synthesis pathway, serine palmitoyltransferase long chain base subunit 2 (Sptlc2) is a key enzymatic component that influences both ceramide production and ceramide species composition.^8^, ^15^ Previous studies in obesity‐related or high‐fat diet‐associated metabolic disease have linked Sptlc2 upregulation to ceramide accumulation, hepatocyte dysfunction, inflammatory activation, and mitochondrial impairment.^16^, ^17^, ^18^, ^19^, ^20^ These studies support a role for Sptlc2 in lipid‐induced cellular stress, but they do not establish whether the same pathway is operative in a nonobese NAFLD context, nor whether Sptlc2 is integrated with mitochondrial dysfunction, inflammation and cell injury in a reproducible mechanistic sequence within this phenotype.^14^, ^21^

Accordingly, the present study focused on three unresolved questions in a dry‐fried soybean (DFS)‐induced nonobese NAFLD model. First, the lipotoxic drivers of hepatic injury in nonobese NAFLD remain insufficiently characterized. Second, evidence is limited regarding whether de novo ceramide synthesis is a dominant source of ceramide dysregulation in this model. Third, the extent to which Sptlc2 is functionally linked to ceramide accumulation, mitochondrial dysfunction, inflammatory responses, and hepatocyte injury has not been systematically examined. In this context, the purpose of the present study was not to define differences between nonobese and obese NAFLD directly, because an obesogenic‐diet comparator was not included, but rather to characterize a reproducible pathological axis within a nonobese model. Using integrated metabolomic and proteomic analyses, combined with in vivo and in vitro functional experiments, we evaluated the association of Sptlc2 with sphingolipid dysregulation and tested the functional significance of the Sptlc2–ceramide pathway in the DFS model.

## 2 MATERIALS AND METHODS

### 2.1 Experimental animals and grouping

Specific pathogen‐free male Sprague–Dawley rats (6–8 weeks old) were obtained from Hunan Silaike Jingda Laboratory Animal Co., Ltd. Animals were housed at 23 ± 3°C under a 12 h light/dark cycle with free access to standard chow and tap water. After a 2‐week acclimation period, rats were used for subsequent experiments. All animal procedures were approved by the Institutional Animal Care and Use Committee (Approval No. JNCH2024‐059).

For in vivo Sptlc2 knockdown, recombinant adeno‐associated virus serotype 2/8 (AAV2/8) carrying sh‐Sptlc2 or negative control short hairpin RNA was administered through the lateral tail vein. The viral titer was 1.3 × 10^11^ vg/mL, and the injection volume was 150 μL per rat.

The term “sh‐Sptlc2” in the animal experiments refers to AAV2/8‐mediated liver‐directed knockdown. Co‐immunofluorescence staining of Sptlc2 and hepatocyte nuclear factor 4 alpha (HNF4α) was performed on liver sections, and Sptlc2 fluorescence intensity was quantified within HNF4α‐positive cells.^22^, ^23^

The NAFLD model was established using DFS as previously reported.^24^ Rats in the DFS groups received a diet consisting of 40% basal feed and 60% high‐temperature DFS for 12 weeks. Control rats received standard chow. Body weight, body length, Lee's index, serum alanine aminotransferase (ALT), and serum aspartate aminotransferase (AST) were measured at week 12. Lee's index was calculated as [body weight (g)^(1/3)^ × 1000]/body length (cm).

Rats were assigned to the following groups: control, DFS, control + sh‐NC, control + sh‐Sptlc2, DFS + sh‐NC, DFS + sh‐Sptlc2, and DFS + sh‐Sptlc2 + C2‐ceramide. Each group contained 6 rats. In the rescue group, C2‐ceramide was administered by intraperitoneal injection at 1.0 mg/kg/day during the experimental period.

At sacrifice, blood was collected from the abdominal aorta and centrifuged to obtain serum. Serum and liver biochemical indices, including ALT, AST, triglyceride (TG), and total cholesterol (TC), were measured using an automated biochemical analyzer. Hepatic FFA levels were determined using the Amplex Red FFA Assay Kit according to the manufacturer's instructions.

### 2.2 Hematoxylin and eosin staining

Liver tissues were fixed in 10% neutral buffered formalin, embedded in paraffin, and sectioned at 4 μm. After deparaffinization and rehydration, sections were stained with hematoxylin and eosin using a standard protocol, followed by dehydration, clearing, and mounting.

Histological evaluation was performed on hematoxylin and eosin staining (H&E)‐stained sections under light microscopy. Ten nonoverlapping fields per section were assessed at ×200 magnification. Histological activity was evaluated using a semiquantitative scoring system adapted from the NAFLD activity score framework, with a total score ranging from 0 to 8, including steatosis (0–3), lobular inflammation (0–3), and hepatocellular injury/ballooning (0–2).^25^, ^26^ Scoring was performed independently by two observers blinded to group allocation. The mean score across fields for each animal was used for statistical analysis. Each animal was treated as one biological replicate.

### 2.3 Oil Red O staining

Frozen liver sections (8 μm) were fixed in 4% paraformaldehyde for 10–15 min, rinsed with distilled water, stained with freshly prepared Oil Red O working solution for 8–10 min, briefly differentiated in 75% ethanol, counterstained with hematoxylin, and mounted with aqueous mounting medium.

Images were acquired under identical exposure conditions. Lipid accumulation was quantified in ImageJ/Fiji as the percentage of Oil Red O‐positive area per field. Multiple nonoverlapping fields were analyzed for each section using a uniform threshold and identical background settings across groups. The mean value for each animal was used for statistical analysis.

### 2.4 Immunofluorescence staining

Paraffin‐embedded liver sections were deparaffinized, rehydrated, and subjected to antigen retrieval. Sections were permeabilized with 0.5% Triton X‐100 for 10 min and blocked in phosphate‐buffered saline (PBS) containing 5% bovine serum albumin and 0.5% Triton X‐100 for 1 h at room temperature.

For macrophage infiltration analysis, sections were incubated overnight at 4°C with anti‐F4/80 antibody (ab300421, Abcam, 1:50). For hepatocyte colocalization analysis, sections were incubated with anti‐Sptlc2 antibody (ab307432, Abcam, 1:50) and anti‐HNF4α antibody. After washing with PBS, sections were incubated with species‐matched fluorescent secondary antibodies for 1.5 h at room temperature and mounted with DAPI‐containing mounting medium.

Images were acquired using an Olympus VS120 microscope. Quantification was performed in Fiji/ImageJ using identical acquisition settings in all groups. For Sptlc2/HNF4α double staining, Sptlc2 fluorescence intensity was quantified within HNF4α‐positive cells. For F4/80 staining, the F4/80‐positive area was quantified as a percentage of the total tissue area. Background subtraction and threshold settings were kept constant within each experiment. Image analysis was performed in a blinded manner.

### 2.5 Detection of GSH, MDA, and SOD

A 10% liver tissue homogenate was prepared for biochemical analysis. Reduced glutathione (GSH), malondialdehyde (MDA), and total superoxide dismutase (SOD) were measured using commercial assay kits according to the manufacturer's instructions.

### 2.6 Proteomic detection and analysis

Liver tissues from the control and DFS groups were subjected to quantitative proteomic analysis. Protein concentrations were determined by bicinchoninic acid (BCA) assay. For each sample, 2 μL of protein solution was mixed with 18 μL of water and 200 μL of BCA working reagent, incubated at 37°C for 30 min, and measured at 562 nm using a microplate reader. Protein concentrations were calculated from a standard curve.

For sample preparation, 100 μg of protein per sample was dissolved in 100 mM triethylammonium bicarbonate buffer, reduced with 10 mM tris(2‐carboxyethyl)phosphine at 37°C for 60 min, alkylated with 40 mM iodoacetamide at room temperature in the dark for 40 min, precipitated with six volumes of prechilled acetone at −20°C for 4 h, and centrifuged at 10,000 × *g*. The pellet was redissolved in triethylammonium bicarbonate buffer and digested overnight with trypsin at an enzyme‐to‐protein ratio of 1:50. Peptides were redissolved in 0.1% trifluoroacetic acid, desalted using HLB columns, concentrated, and quantified before mass spectrometric analysis.

Peptides were fractionated by high‐pH reverse‐phase ultra‐performance liquid chromatography, and 20 fractions were collected. Mass spectrometry was performed on a Q Exactive HF‐X instrument coupled to an EASY‐nLC 1200 system. Data‐dependent acquisition (DDA) data were acquired with MS1 resolution set at 70,000 and a scan range of *m/z* 350–1300, then searched against the UniProt *Rattus norvegicus* database using Proteome Discoverer v2.4 with a false discovery rate of <1%. For data‐independent acquisition (DIA), the same instrument was used with MS1 resolution set at 70,000. Data were acquired using a variable window strategy, and indexed retention time standards were added for calibration. DDA data were imported into Spectronaut v17 to generate a spectral library. DIA data were processed to generate a protein quantification matrix. All steps were performed in triplicate under controlled conditions.

Differential protein analysis between the control and DFS groups was performed using the edgeR package in R. Proteins with |log_2_ fold change| > 1 and *p* < 0.05 were considered differentially expressed. Volcano plots were generated using ggplot2. Gene ontology (GO) and Kyoto encyclopedia of genes and genomes (KEGG) enrichment analyses were performed using clusterProfiler. Enriched terms and pathways were visualized using enrichplot. Genes related to sphingolipid metabolism were retrieved from GeneCards and intersected with differentially expressed proteins. The overlap was visualized using ggplot2 and VennDiagram.

### 2.7 Untargeted metabolomics

Liver tissues from the control and DFS groups (*n* = 6/group) were used for untargeted metabolomic profiling. For each sample, 50 mg of tissue was homogenized in methanol/water (4:1, v/v) containing 0.02 mg/mL L‐2‐chlorophenylalanine as an internal standard. Samples were homogenized at low temperature, subjected to ultrasonic extraction, incubated at −20°C for 30 min, and centrifuged at 13,000 × *g* for 15 min at 4°C. Supernatants were transferred for analysis. Equal volumes of all samples were pooled to prepare a quality control (QC) sample. One QC sample was injected every 5–15 samples during analysis.

Metabolomic profiling was performed on a UHPLC‐Q Exactive HF‐X system equipped with an ACQUITY UPLC HSS T3 column. The injection volume was 3 μL. The mobile phases consisted of solvent A (95% water + 5% acetonitrile with 0.1% formic acid) and solvent B (47.5% acetonitrile + 47.5% isopropanol + 5% water with 0.1% formic acid). The column temperature was 40°C, and the flow rate was 0.40 mL/min. Data were acquired in both positive and negative ion modes with a scan range of *m/z* 70–1050, MS1 resolution of 60,000, and MS2 resolution of 7500. Stepped collision energy was set at 20–40–60 V.

Raw data were processed in Progenesis QI v2.3 for baseline correction, peak detection, integration, retention‐time correction, and peak alignment. Metabolites were annotated against the Human Metabolome Database, Metlin, and an in‐house database. Features detected in at least 80% of samples in any group were retained. Remaining missing values were imputed with the minimum value in the original matrix. Peak areas were normalized by total ion current. Features with a relative standard deviation >30% in QC samples were excluded. The processed data were log10‐transformed before statistical analysis.

Principal coordinate analysis was performed for sample clustering. Differential metabolites were defined by variable importance in projection >1 and *p* < 0.05. Pathway enrichment analysis was performed in MetaboAnalyst 6.0.

### 2.8 Clinical sample collection and preparation

Serum samples were collected at the Jinan Central Hospital Affiliated to Shandong First Medical University. Written informed consent was obtained from all participants. The study was approved by the institutional ethics committee and conducted in accordance with the Declaration of Helsinki.

A total of 115 individuals aged 20–70 years were initially screened. Eighteen participants were enrolled for serum ceramide analysis, including 9 nonobese NAFLD patients and 9 nonobese healthy controls.

According to Chinese adult body mass index criteria, nonobesity was defined as a body mass index <24.0 kg/m^2^. NAFLD was diagnosed according to the 2018 guideline criteria^27^ and abdominal ultrasonography after exclusion of alcoholic liver disease and other causes of hepatic steatosis. Exclusion criteria included viral hepatitis, drug‐induced liver injury, autoimmune hepatitis, Wilson's disease, biliary disease, and other chronic liver diseases.

Fasting blood samples were obtained in the morning. Serum biochemical parameters included AST, ALT, TG, TC, and fasting plasma glucose. Group characteristics are summarized in Supporting Information S1: Table S1.

For sample preparation, 40 μL of serum was mixed with 500 μL methanol containing 1 μg/mL Cer(d18:1/17:0) as an internal standard, vortexed for 10 min, centrifuged at 13,000 rpm for 10 min at 4°C, and 2 μL of the supernatant was injected for liquid chromatography–tandem mass spectrometry analysis.

### 2.9 Liquid chromatography–tandem mass spectrometry (LC–MS/MS) analysis

Ceramides in serum, liver tissue, and cultured cells were measured using an LCMS‐8050 system operated in positive ion multiple reaction monitoring mode. Samples (50–100 μL serum, 10–20 mg tissue, or 1 × 10^6^ cells) were extracted with methanol containing Cer(d18:1/17:0) as an internal standard using a modified Bligh–Dyer procedure. The organic phase was collected, dried under nitrogen, and reconstituted for analysis.

Chromatographic separation was performed on an ACQUITY UPLC BEH C18 column (2.1 × 100 mm, 1.7 μm) at 45°C. The mobile phases were ultrapure water containing 20 mM ammonium formate and acetonitrile/isopropanol (1:1, v/v). The flow rate was 0.3 mL/min. Total ceramide and individual ceramide species, including Cer(d18:1/14:0), Cer(d18:1/16:0), Cer(d18:1/18:0), Cer(d18:1/20:0), Cer(d18:1/22:0), and Cer(d18:1/24:0), were monitored.

### 2.10 Isolation and culture of primary rat hepatocytes

Primary hepatocytes were isolated from 6–7‐week‐old male Sprague–Dawley rats by portal vein perfusion with calcium‐ and magnesium‐free Hank's balanced salt solution followed by 0.05% type IV collagenase digestion. Hepatocytes were collected by centrifugation at 50 × *g* for 30 s, washed, and seeded on collagen‐coated dishes. Cell viability was assessed by trypan blue exclusion, and preparations with viability >90% were used.

Cells were cultured in Dulbecco's modified Eagle's medium supplemented with 10% fetal bovine serum, 100 U/mL penicillin, and 100 μg/mL streptomycin at 37°C with 5% CO_2_. When cells reached 60%–70% confluence, they were transduced with sh‐Sptlc2 or sh‐NC lentiviral constructs. The sh‐Sptlc2 sequence is listed in Supporting Information S1: Table S2. Two sh‐Sptlc2 constructs were screened at the protein level, and the construct with higher knockdown efficiency was used for subsequent experiments (Supporting Information S1: Figure S1). After transduction, cells were maintained for 48 h before treatment. For lipotoxicity induction, cells were treated with 0.5 mM palmitic acid (PA) for 0–24 h. For rescue experiments, C2‐ceramide was dissolved in dimethyl sulfoxide and added to serum‐free medium at a final concentration of 50 μM. The final dimethyl sulfoxide concentration did not exceed 0.1% (v/v). The in vitro rescue experiment included four groups: sh‐NC, sh‐NC + PA, sh‐Sptlc2 + PA, and sh‐Sptlc2 + PA + C2‐ceramide. Cells were harvested after 24 h of treatment for subsequent assays.^28^, ^29^

### 2.11 LDH and apoptosis detection

Cells were seeded in 24‐well plates at a density of 2 × 10^5^ cells per well. At the indicated time points, lactate dehydrogenase (LDH) release, propidium iodide (PI) staining, and Annexin V‐FITC staining were used to assess cell viability and apoptosis. For LDH detection, culture supernatants were collected and analyzed using an LDH assay kit (Beyotime). For apoptosis/necrosis detection, cells were stained with a combination of PI and Annexin V‐FITC (ES Science). Fluorescence was analyzed by flow cytometry using a CytoFLEX S flow cytometer (Beckman Coulter).

### 2.12 Terminal deoxynucleotidyl transferase dUTP nick‐end labeling (TUNEL) staining

TUNEL staining was performed on liver sections and cultured hepatocytes using a one‐step apoptosis detection kit. Images were acquired using an Olympus VS120 microscope. For tissue sections, TUNEL‐positive cells were counted in randomly selected nonoverlapping fields under identical magnification. For cell experiments, quantification was performed separately from tissue analysis using the same acquisition settings within each experiment.

### 2.13 Lipid accumulation detection

For cellular lipid staining, hepatocytes were seeded on glass coverslips in 6‐well plates. After treatment, cells were washed with PBS, fixed with 4% paraformaldehyde, stained with BODIPY 493/503 (1 μg/mL in PBS) for 30 min in the dark, and mounted with DAPI‐containing antifade medium. Images were acquired using an Olympus VS120 microscope. Lipid accumulation was quantified as BODIPY‐positive fluorescence normalized to cell number under identical acquisition conditions.

### 2.14 Mitochondrial function

Mitochondrial function was assessed in cultured hepatocytes by measuring the oxygen consumption rate (OCR) and the extracellular acidification rate (ECAR) using a Seahorse XFe analyzer. Hepatocytes were seeded in Seahorse cell culture microplates at 2.5 × 10^4^ cells per well. Four to six technical replicate wells were analyzed for each condition. Before measurement, cells were washed and incubated for 1 h at 37°C in a non‐CO_2_ incubator in Seahorse assay medium supplemented with glucose, glutamine, and pyruvate. OCR and ECAR were recorded at baseline and after sequential injection of oligomycin, carbonyl cyanide‐4‐(trifluoromethoxy)phenylhydrazone, and rotenone/antimycin A. Final concentrations were 1, 0.25, and 1 μM, respectively. OCR and ECAR values were normalized to cell number or protein content, as specified in each experiment.^30^

Following treatment, cells were stained with Fura‐2 AM, 2′,7′‐dichlorodihydrofluorescein diacetate, and tetramethylrhodamine ethyl ester. Intracellular calcium, reactive oxygen species (ROS), and mitochondrial membrane potential were quantified using a microplate reader. Mitochondrial permeability transition pore (mPTP) opening was evaluated using a fluorescence detection kit under confocal microscopy. Detailed instrument information is provided in Supporting Information S1: Table S4.

### 2.15 Transmission electron microscopy

Primary hepatocytes from each treatment group were fixed in 2.5% glutaraldehyde at 4°C, post‐fixed in 1% osmium tetroxide, dehydrated in a graded ethanol series, and embedded in epoxy resin. Ultrathin sections were prepared, stained with uranyl acetate and lead citrate, and examined under a transmission electron microscope. Mitochondrial ultrastructural features, including swelling, cristae disruption, and vacuolar degeneration, were evaluated.

### 2.16 Western blot

Proteins were extracted in radioimmunoprecipitation assay buffer containing protease inhibitors and incubated on ice for 30 min. Lysates were centrifuged, and supernatants were collected. Protein concentrations were determined by BCA assay. Equal amounts of protein were separated by sodium dodecyl sulfate–polyacrylamide gel electrophoresis and transferred to polyvinylidene difluoride membranes. Membranes were incubated with primary antibodies against Sptlc2 (ab307432, Abcam, 1:50), cleaved‐caspase‐3 (9661, Cell Signaling Technology, 1:1000), Bax (2772, Cell Signaling Technology, 1:1000), Bcl‐2 (ab194583, Abcam, 1:1000), voltage‐dependent anion channel 1 (VDAC1) (81538‐1‐RR, Proteintech, 1:2000), and GAPDH (10494‐1‐AP, Proteintech, 1:5000), followed by horseradish peroxidase‐conjugated secondary antibody (7074S, Cell Signaling Technology, 1:2000). Signals were detected using enhanced chemiluminescence and imaged with a Tanon 5200 system.

### 2.17 Quantitative real‐time PCR

Total RNA was extracted using TRIzol reagent. RNA concentration and purity were measured with a NanoDrop spectrophotometer. Reverse transcription was performed using a PrimeScript reverse transcription kit. Quantitative polymerase chain reaction was carried out with SYBR Premix Ex Taq on a LightCycler 480 system. GAPDH was used as the internal control. Primer sequences are listed in Supporting Information S1: Table S3. Relative transcript levels were calculated using the 2^−ΔΔCt^ method.

### 2.18 Statistical analysis

Statistical analysis was performed using GraphPad Prism 10.1.2. Data are presented as mean ± standard deviation. In animal experiments, each rat was treated as one biological replicate. In cell experiments, independent experiments were treated as biological replicates. For image‐based assays, multiple fields were quantified for each sample, averaged to a single value, and then used for group comparison. For Seahorse assays, technical replicate wells were averaged within each independent experiment, and the mean value from each independent experiment was used as one biological replicate for group comparison.

For two‐group comparisons, an unpaired Student's *t* test was used when data met assumptions of normality and homogeneity of variance. For multiple‐group comparisons, one‐way analysis of variance followed by Tukey's post hoc test was used. A two‐sided *p* < 0.05 was considered statistically significant. Detailed instrument information is provided in Supporting Information S1: Table S4.

## 3 RESULTS

### 3.1 Sphingolipid dysregulation and Sptlc2 nomination in the DFS model

**FIGURE 1:**
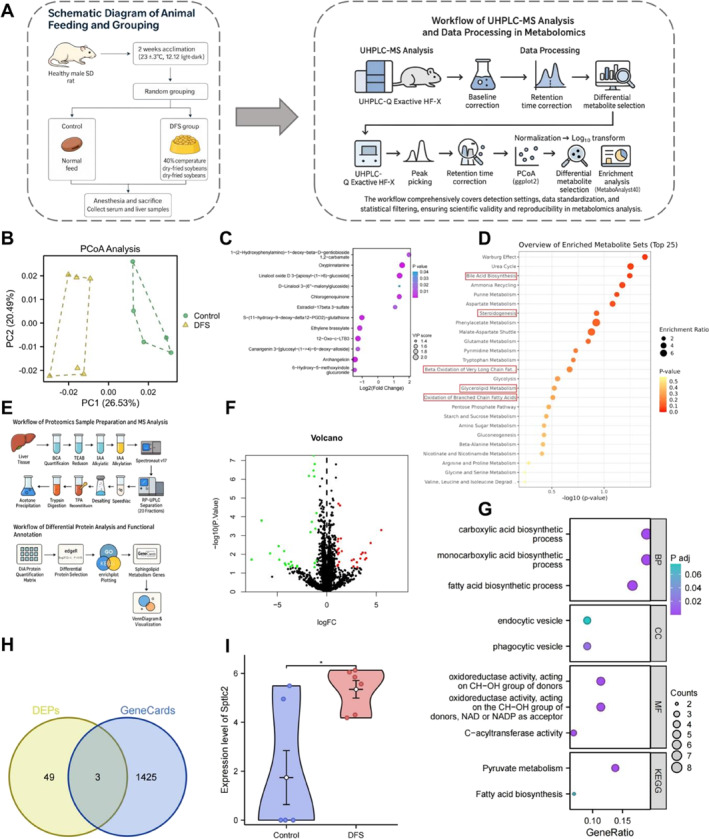
Multi‐omics profiling of the DFS‐induced non‐obese NAFLD model. (A) Workflow of DFS model establishment and sample collection. (B) Principal coordinate analysis of liver metabolomic data. (C) Distribution of differential metabolites between the control and DFS groups. (D) Pathway enrichment analysis of differential metabolites. (E) Workflow of proteomic sample preparation and acquisition. (F) Volcano plot of differentially expressed proteins between the control and DFS groups. (G) GO and KEGG enrichment analyses of differentially expressed proteins. (H) Intersection analysis between differentially expressed proteins and GeneCards‐derived sphingolipid metabolism genes. (I) Quantitative proteomic readout of Sptlc2. Statistical significance is indicated in the panels; *p < 0.05. DFS, dry‐fried soybean; GO, gene ontology; KEGG, Kyoto encyclopedia of genes and genomes; NAFLD, nonalcoholic fatty liver disease; Sptlc2, serine palmitoyltransferase long chain base subunit 2.

Clinical and questionnaire data indicated that a subset of patients with fatty liver disease did not meet body mass index criteria for overweight or obesity, and frequent consumption of high‐temperature processed foods was associated with nonobese NAFLD. A previously established DFS‐induced SD rat model^24^ was therefore used for liver metabolomic profiling in the control and DFS groups (Figure 1A). Principal coordinate analysis showed clear separation of the metabolic profiles between groups (Figure 1B). More than 700 metabolites were detected by LC–MS, and 145 metabolites were differentially abundant in the DFS group (Figure 1C). Pathway enrichment analysis mapped these metabolites to bile acid biosynthesis, steroidogenesis, β‐oxidation of very long chain fatty acids, glycerolipid metabolism, and the oxidation of branched‐chain fatty acids (Figure 1D).

Quantitative proteomics was then performed on liver tissues from the control and DFS groups (Figure 1E). A total of 4497 proteins were identified, of which 52 were differentially expressed in the DFS group, including 26 upregulated and 26 downregulated proteins (Figure 1F). GO and KEGG analyses again highlighted lipid‐related processes, including carboxylic acid and fatty acid biosynthesis (Figure 1G). To specifically focus on sphingolipid metabolism, we searched the GeneCards database for genes associated with sphingolipid metabolism and identified a total of 1428 related genes (Supporting Information S1: Table S5). Intersecting the differentially expressed proteins with a GeneCards‐derived sphingolipid metabolism gene set yielded three candidates, Becn1, Sptlc2, and Pdxk (Figure 1H). Among these, Sptlc2 was significantly increased in the DFS group (*p* < 0.05) (Figure 1I). These analyses positioned Sptlc2 as a candidate for subsequent functional experiments and supported the presence of sphingolipid‐related alterations in the DFS model. These results indicate sphingolipid‐related molecular alterations in the DFS model and place Sptlc2 within the candidate set for subsequent functional analyses.

### 3.2 Long‐chain ceramide changes after Sptlc2 knockdown in vitro and in vivo

**FIGURE 2:**
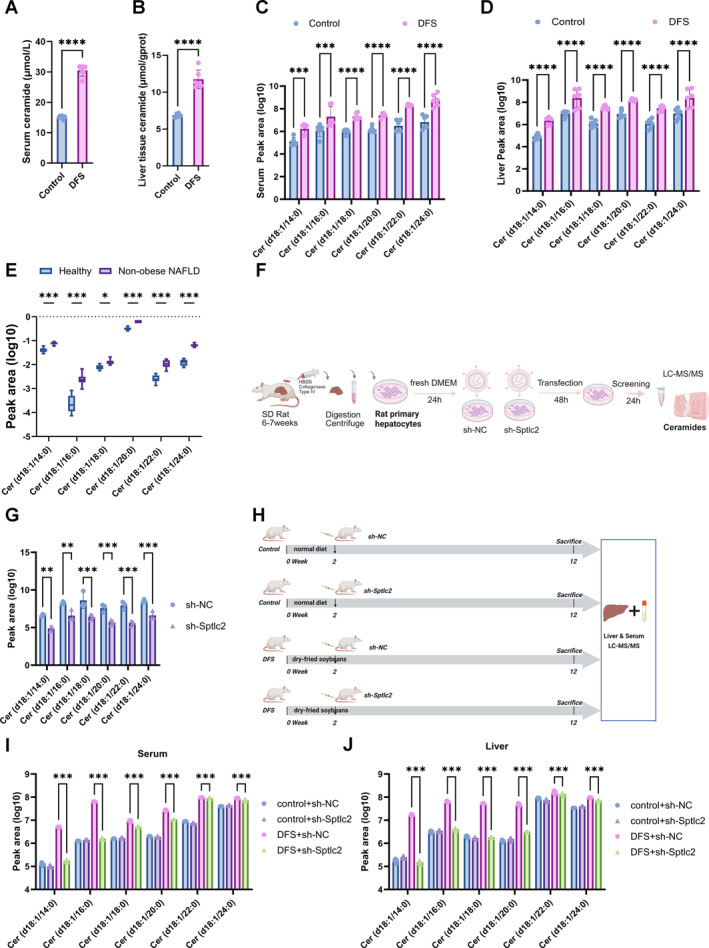
Ceramide profiles after Sptlc2 knockdown in vitro and in vivo. (A, B) LC–MS/MS analysis of total ceramide in serum (A) and liver tissue (B). (C, D) LC–MS/MS analysis of major long‐chain ceramide species in serum (C) and liver tissue (D). (E) Serum long‐chain ceramide species in healthy controls and patients with nonobese NAFLD. (F) Workflow for shRNA‐based manipulation in primary rat hepatocytes. (G) Ceramide species measured in primary hepatocytes. (H) Workflow for liver‐directed Sptlc2 knockdown in vivo. (I, J) LC–MS/MS analysis of long‐chain ceramide species in serum (I) and liver tissue (J) after in vivo knockdown. Cell experiments were repeated three times; animal experiments, *n* = 6. Statistical significance is indicated in the panels; *p < 0.05, **p < 0.01, ***p < 0.001, ****p < 0.0001. LC–MS/MS, Liquid chromatography–tandem mass spectrometry; NAFLD, nonalcoholic fatty liver disease; shRNA, short hairpin RNA; Sptlc2, serine palmitoyltransferase long chain base subunit 2.

Ceramide levels were next assessed in animal and cell samples. LC–MS/MS showed that, compared with the control group, the DFS group had increased total ceramide in both serum and liver tissue (all *p* < 0.0001) (Figure 2A,B), together with elevations in multiple long‐chain ceramide species, including Cer(d18:1/14:0), Cer(d18:1/16:0), Cer(d18:1/18:0), Cer(d18:1/20:0), Cer(d18:1/22:0) and Cer(d18:1/24:0) (all *p* < 0.001) (Figure 2C,D). In the clinical cohort, several long‐chain ceramide species were also increased in serum from patients with nonobese NAFLD (all *p* < 0.05) (Figure 2E). An in vitro sh‐Sptlc2 primary rat hepatocyte model was then established (Figure 2F). LC–MS/MS showed reduced levels of multiple ceramide species in sh‐Sptlc2 cells relative to sh‐NC cells (all *p* < 0.01) (Figure 2G). In the in vivo experiments, control + sh‐NC, control + sh‐Sptlc2, DFS + sh‐NC and DFS + sh‐Sptlc2 groups were generated (Figure 2H). Relative to DFS + sh‐NC, the DFS + sh‐Sptlc2 group showed lower levels of multiple long‐chain ceramide species in both serum and liver tissue, with particularly marked changes in Cer(d18:1/16:0) and Cer(d18:1/18:0) (all *p* < 0.001) (Figure 2I,J). These data support an association between changes in Sptlc2 abundance and alterations in long‐chain ceramide levels.

### 3.3 Hepatocyte‐associated Sptlc2 signal, tissue injury and inflammation after liver‐directed knockdown

**FIGURE 3:**
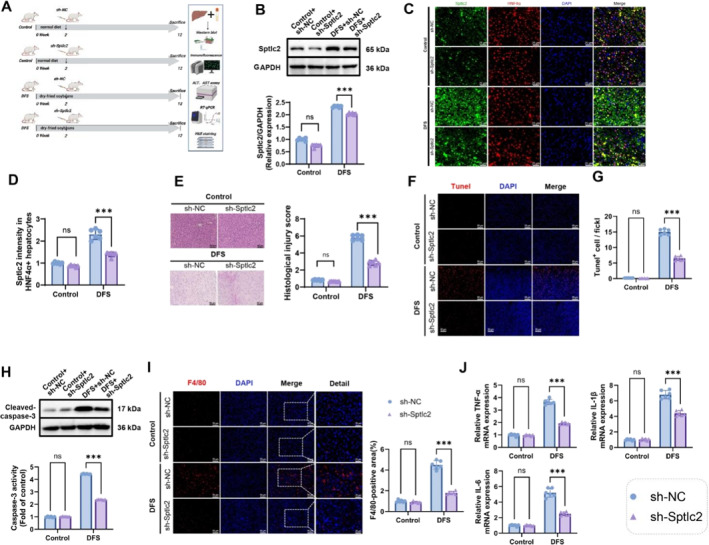
Liver‐directed Sptlc2 knockdown in DFS rats. (A) Schematic overview of the animal experiment. (B) Western blot analysis of hepatic Sptlc2 and corresponding quantification. (C, D) Double immunofluorescence staining of Sptlc2 and HNF4α and corresponding quantification; Sptlc2, green; HNF4α, red; DAPI, blue; quantification indicates Sptlc2 fluorescence intensity in HNF4α‐positive hepatocytes; scale bar, 50 μm. (E) H&E staining and histological injury scoring; scale bar, 50 μm. (F, G) TUNEL staining and corresponding quantification; scale bar, 50 μm. (H) Western blot analysis of cleaved‐caspase‐3 and caspase‐3 activity. (I) F4/80 immunofluorescence staining and quantification of positive area; scale bar, 25 μm. (J) RT‐qPCR analysis of TNF‐α, IL‐1β and IL‐6. Data are mean ± SD, *n* = 6. Statistical significance is indicated in the panels; ns, not significant; ***p < 0.001. DFS, dry‐fried soybean; H&E, hematoxylin and eosin staining; HNF4α, hepatocyte nuclear factor 4 alpha; RT‐qPCR, quantitative real‐time PCR; Sptlc2, serine palmitoyltransferase long chain base subunit 2; TUNEL, terminal deoxynucleotidyl transferase dUTP nick‐end labeling.

Tissue phenotypes after liver‐directed Sptlc2 knockdown were evaluated in the DFS model (Figure 3A). Western blotting showed that, relative to control + sh‐NC, hepatic Sptlc2 protein in control + sh‐Sptlc2 showed a downward trend without a significant difference (Figure 3B), whereas DFS + sh‐NC showed increased Sptlc2 and DFS + sh‐Sptlc2 showed reduced Sptlc2 under DFS conditions (*p* < 0.001) (Figure 3B). Double immunofluorescence showed that the increased Sptlc2 signal in DFS liver was concentrated in HNF4α‐positive hepatocytes and was reduced in DFS + sh‐Sptlc2; quantification showed the same direction of change (*p* < 0.001) (Figure 3C,D).

At week 12, body weight, Lee's index, serum AST and serum ALT were not different across groups (Supporting Information S1: Figure S2A–D). H&E staining showed preserved lobular architecture and limited inflammatory infiltration in control + sh‐NC and control + sh‐Sptlc2, whereas DFS + sh‐NC showed hepatocellular vacuolation, disorganized hepatic cords and increased inflammatory infiltration. These changes were reduced in DFS + sh‐Sptlc2, consistent with the histological injury score (*p* < 0.001) (Figure 3E).

TUNEL staining showed little difference between control groups but increased TUNEL positivity in DFS + sh‐NC, with lower values in DFS + sh‐Sptlc2 (*p* < 0.001) (Figure 3F,G). In parallel, cleaved‐caspase‐3 activity was increased in DFS + sh‐NC and reduced in DFS + sh‐Sptlc2 (*p* < 0.001) (Figure 3H). F4/80 immunofluorescence showed increased macrophage infiltration in DFS + sh‐NC and reduced infiltration in DFS + sh‐Sptlc2 (*p* < 0.001) (Figure 3I). Inflammation‐related transcript levels showed the same direction of change, with increased TNF‐α, IL‐1β and IL‐6 in DFS + sh‐NC and lower levels in DFS + sh‐Sptlc2 (all *p* < 0.001) (Figure 3J). These data link hepatocyte‐associated Sptlc2 signal changes with tissue injury, apoptosis and inflammatory phenotypes.

### 3.4 Hepatic lipotoxicity and oxidative stress after Sptlc2 knockdown

**FIGURE 4:**
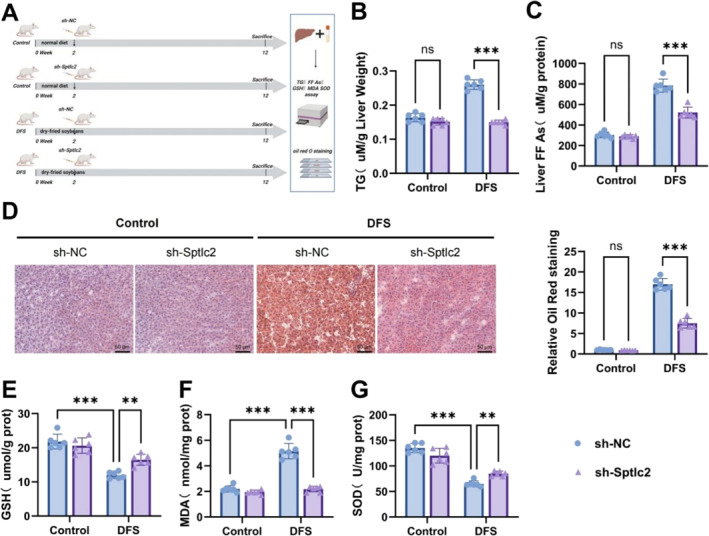
Hepatic lipotoxicity and oxidative stress after Sptlc2 knockdown. (A) Schematic overview of the animal experiment. (B, C) Hepatic TG (B) and FFA (C). (D) Oil Red O staining and corresponding quantification; scale bar, 50 μm. (E–G) Hepatic GSH (E), MDA (F) and SOD (G). Animal experiments, *n* = 6. Statistical significance is indicated in the panels; **p < 0.01, ***p < 0.001. FFA, free fatty acid; GSH, glutathione; MDA, malondialdehyde; SOD, superoxide dismutase; Sptlc2, serine palmitoyltransferase long chain base subunit 2; TG, triglyceride.

Hepatic lipotoxicity‐related phenotypes were next examined after Sptlc2 knockdown (Figure 4A). Supplementary analyses showed no change in serum TG, serum TC, or hepatic TC after Sptlc2 knockdown (Supporting Information S1: Figure S3A–C). In contrast, hepatic TG and FFA were lower in DFS + sh‐Sptlc2 than in DFS + sh‐NC (both *p* < 0.001) (Figure 4B,C). Oil Red O staining showed increased hepatic lipid droplet accumulation in DFS + sh‐NC and reduced lipid deposition in DFS + sh‐Sptlc2 (*p* < 0.001) (Figure 4D).

Oxidative stress‐related measurements showed higher MDA and lower GSH and SOD in DFS + sh‐NC, whereas DFS + sh‐Sptlc2 showed the opposite direction of change (all *p* < 0.01) (Figure 4E–G). These changes were primarily detected at the liver tissue level and were aligned with alterations in intrahepatic lipid droplets and FFAs.

### 3.5 Functional rescue of hepatocyte injury by C2‐ceramide

**FIGURE 5:**
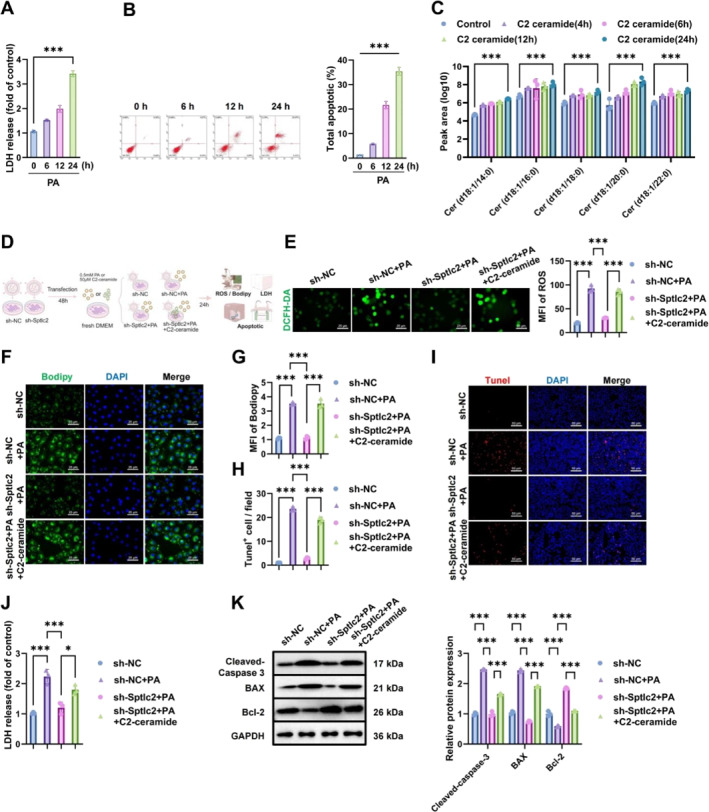
Functional rescue of hepatocyte injury by C2‐ceramide. (A, B) LDH release (A) and flow‐cytometric apoptosis analysis (B) after PA treatment at different time points. (C) Changes associated with long‐chain ceramides after C2‐ceramide treatment. (D) Schematic overview of the cell‐based workflow. (E) DCFH‐DA staining for ROS; scale bar, 25 μm. (F, G) Bodipy staining and corresponding quantification; scale bar, 25 μm. (H, I) TUNEL staining and corresponding quantification; scale bar, 50 μm. (J) LDH assay. (K) Western blot and densitometric quantification of cleaved‐caspase‐3, BAX and Bcl‐2. Cell experiments were repeated three times. Statistical significance is indicated in the panels; *p < 0.05, ***p < 0.001. LDH, lactate dehydrogenase; PA, palmitic acid; ROS, reactive oxygen species; TUNEL, terminal deoxynucleotidyl transferase dUTP nick‐end labeling.

Primary hepatocytes were used to evaluate PA‐induced injury and the effects of C2‐ceramide. After PA treatment, LDH release and apoptosis increased over time (all *p* < 0.001) (Figure 5A,B). Changes associated with intracellular long‐chain ceramides were then assessed after C2‐ceramide exposure (all *p* < 0.001) (Figure 5C), and injury phenotypes were compared across sh‐NC, sh‐NC + PA, sh‐Sptlc2 + PA, and sh‐Sptlc2 + PA + C2‐ceramide groups (Figure 5D).

Relative to sh‐NC, sh‐NC + PA showed increased ROS, lipid droplet accumulation, LDH release and TUNEL positivity. These measures were lower in sh‐Sptlc2 + PA and increased again after C2‐ceramide treatment (all *p* < 0.001) (Figure 5E–J). At the protein level, sh‐NC + PA showed higher cleaved‐caspase‐3 and BAX and lower Bcl‐2; the direction of change was reversed in sh‐Sptlc2 + PA and shifted back after C2‐ceramide treatment (all *p* < 0.001) (Figure 5K). In this setting, C2‐ceramide functioned as a rescue condition aligned with the injury‐related changes observed after Sptlc2 knockdown.

### 3.6 Ceramide‐associated mitochondrial homeostasis and ultrastructural changes

**FIGURE 6:**
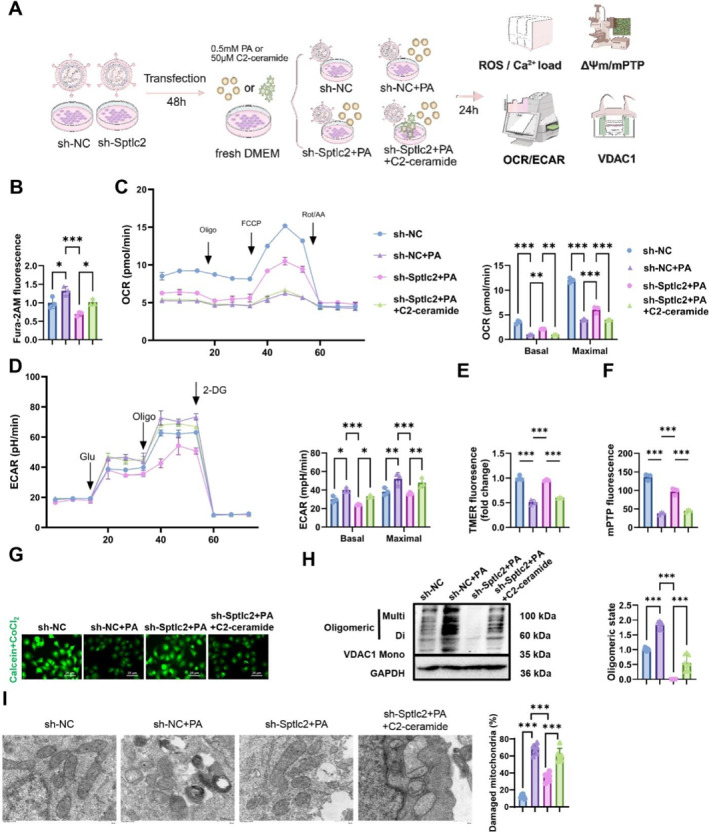
Mitochondrial homeostasis under ceramide‐associated hepatocyte injury. (A) Schematic overview of the cell‐based workflow. (B) Fura‐2 AM‐based measurement of cytosolic Ca^2+^ and corresponding quantification. (C) Seahorse OCR analysis with quantification of basal and maximal respiration. (D) Seahorse ECAR analysis and corresponding quantification. (E) TMRE staining for ΔΨm. (F, G) Calcein‐AM/CoCl_2_ quenching assay for mPTP opening, including representative images and quantification; scale bar, 25 μm. (H) Western blot analysis of VDAC1 oligomerization and densitometric quantification. (I) Transmission electron microscopy of mitochondrial ultrastructure in primary hepatocytes; if included, quantification shows the percentage of damaged mitochondria. Cell experiments were repeated three times. Statistical significance is indicated in the panels; *p < 0.05, **p < 0.01, ***p < 0.001. ECAR, extracellular acidification rate; mPTP, mitochondrial permeability transition pore; OCR, oxygen consumption rate; TMRE, tetramethylrhodamine ethyl ester; VDAC1, voltage‐dependent anion channel 1.

Mitochondrial homeostasis‐related measurements were next assessed in primary hepatocytes (Figure 6A). Relative to sh‐NC, sh‐NC + PA showed increased cytosolic Ca^2+^ and ROS, and both were lower in sh‐Sptlc2 + PA and increased again after C2‐ceramide treatment (all *p* < 0.001) (Figure 6B). Seahorse analysis showed reduced OCR and increased ECAR in sh‐NC + PA, partial recovery in sh‐Sptlc2 + PA, and a shift back toward the sh‐NC + PA profile in sh‐Sptlc2 + PA + C2‐ceramide (all *p* < 0.01) (Figure 6C,D).

Membrane homeostasis‐related measurements showed reduced ΔΨm and increased mPTP opening after PA treatment, with both parameters moving back toward baseline after Sptlc2 knockdown and returning in the opposite direction after C2‐ceramide treatment (all *p* < 0.001) (Figure 6E–G). VDAC1 oligomerization was increased in sh‐NC + PA, reduced in sh‐Sptlc2 + PA, and increased again in sh‐Sptlc2 + PA + C2‐ceramide (all *p* < 0.001) (Figure 6H). Transmission electron microscopy showed intact mitochondrial ultrastructure in sh‐NC, whereas sh‐NC + PA showed mitochondrial swelling, disrupted cristae, and vacuolar degeneration. These alterations were reduced in sh‐Sptlc2 + PA and reappeared in sh‐Sptlc2 + PA + C2‐ceramide (Figure 6I). These findings place mitochondrial functional readouts and ultrastructural changes within the same experimental framework.

### 3.7 In vivo C2‐ceramide rescue of liver injury and lipotoxicity

**FIGURE 7:**
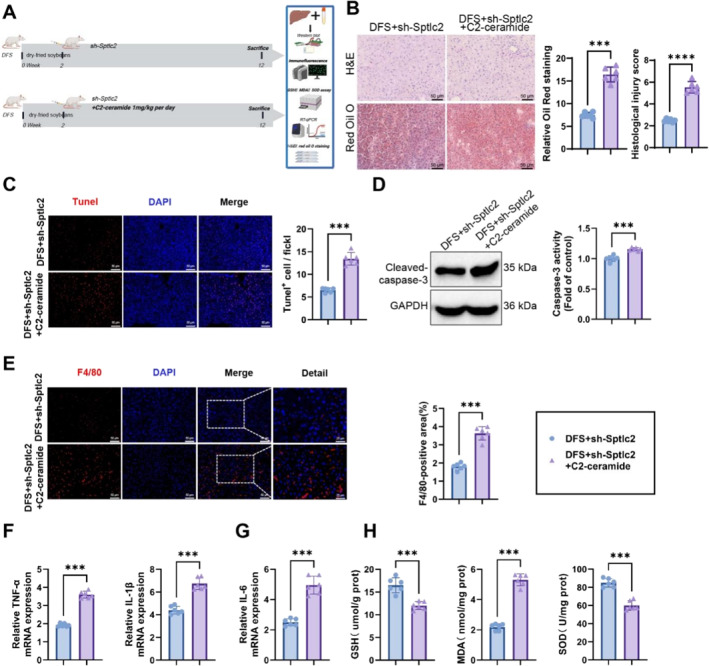
In vivo C2‐ceramide rescue after Sptlc2 knockdown. (A) Schematic overview of DFS feeding, Sptlc2 knockdown and C2‐ceramide treatment in vivo. (B) H&E staining, histological injury scoring, Oil Red O staining and corresponding quantification; scale bar, 50 μm. (C) TUNEL staining; scale bar, 50 μm. (D) Quantification of TUNEL positivity together with caspase‐3 protein detection and activity analysis. (E) F4/80 immunofluorescence staining and quantification of positive area; scale bar, 50 μm. (F, G) RT‐qPCR analysis of TNF‐α, IL‐1β and IL‐6. (H) Hepatic GSH, MDA and SOD levels. Animal experiments, *n* = 6. Statistical significance is indicated in the panels; ***p < 0.001, ****p < 0.0001. DFS, dry‐fried soybean; GSH, glutathione; H&E, hematoxylin and eosin staining; MDA, malondialdehyde; RT‐qPCR, quantitative real‐time PCR; SOD, superoxide dismutase; Sptlc2, serine palmitoyltransferase long chain base subunit 2; TUNEL, terminal deoxynucleotidyl transferase dUTP nick‐end labeling.

The effects of C2‐ceramide were then assessed in vivo after Sptlc2 knockdown (Figure 7A). Relative to DFS + sh‐Sptlc2, the DFS + sh‐Sptlc2 + C2‐ceramide group showed more extensive hepatocellular vacuolation, inflammatory infiltration, and architectural disorganization by H&E staining, together with a higher histological injury score; Oil Red O staining showed increased hepatic lipid droplet accumulation after C2‐ceramide treatment (all *p* < 0.001) (Figure 7B).

TUNEL staining and caspase‐3 activity showed higher hepatocyte apoptosis in DFS + sh‐Sptlc2 + C2‐ceramide than in DFS + sh‐Sptlc2 (all *p* < 0.001) (Figure 7C,D). F4/80 immunofluorescence showed increased macrophage infiltration after C2‐ceramide treatment (*p* < 0.001) (Figure 7E). Inflammation‐related transcript levels showed higher TNF‐α, IL‐1β and IL‐6 in the DFS + sh‐Sptlc2 + C2‐ceramide group (all *p* < 0.001) (Figure 7F,G). In parallel, GSH and SOD were lower and MDA was higher in the C2‐ceramide‐treated group (all *p* < 0.001) (Figure 7H). These in vivo findings were directionally consistent with the cell‐based rescue experiments and support the involvement of a ceramide‐related downstream effect in this phenotype.

## 4 DISCUSSION

NAFLD has attracted increasing attention in Asian populations in recent years, as a substantial proportion of affected individuals do not meet conventional criteria for overweight or obesity.^31^ Clinical studies have shown that this phenotype can still be accompanied by steatosis, inflammation and fibrosis, although disease severity and metabolic background are not uniform across cohorts.^32^, ^33^ In the present study, the DFS model maintained a nonobese phenotype at the level of body weight and Lee's index, yet developed hepatic lipid accumulation, inflammatory changes, and ceramide‐related metabolic abnormalities. Multi‐omics analyses further indicated coordinated disruption of lipid metabolic pathways and identified Sptlc2 as one of a limited number of candidate molecules linked to sphingolipid metabolism. Collectively, these findings support the utility of the DFS model for studying fatty liver under nonobese conditions, while stopping short of defining the observed molecular alterations as specific to the nonobese state.

Ceramides have been widely implicated in lipotoxicity, inflammatory signaling and hepatocellular injury.^34^ In obesity‐associated NAFLD and other metabolic disorders, long‐chain ceramide species have been linked to the severity of steatosis, insulin resistance, and increased apoptosis.^35^, ^36^ Here, both serum and hepatic levels of total ceramides, together with multiple long‐chain ceramide species, were elevated in the DFS model, and exploratory clinical data revealed concordant changes in circulating ceramides. These observations are in line with previous reports associating ceramide accumulation with fatty liver injury,^37^ and suggest that ceramide dysregulation is also present in a nonobese setting. However, the current data do not support the stronger conclusion that such alterations represent a molecular signature unique to nonobese disease.

Intervention experiments further linked Sptlc2 to ceramide metabolism and liver injury. Sptlc2 is an essential component of the serine palmitoyltransferase complex that catalyzes the first step of de novo sphingolipid synthesis.^14^ In the DFS model, Sptlc2 expression was increased, and its knockdown reduced multiple long‐chain ceramide species in both primary hepatocytes and DFS rats, with particularly marked effects on Cer(d18:1/16:0) and Cer(d18:1/18:0). Liver‐directed Sptlc2 knockdown was accompanied by lower histological injury scores, fewer TUNEL‐positive cells, reduced cleaved caspase‐3 activity, decreased F4/80‐positive area, and lower expression of inflammatory transcripts. Hepatic TG and FFA content, as well as Oil Red O‐positive area, were also reduced. In primary hepatocytes, Sptlc2 knockdown attenuated PA‐induced increases in ROS production, LDH release, lipid droplet accumulation, and apoptosis‐related protein changes, whereas these protective effects were shifted back toward injury after C2‐ceramide treatment. Taken together, these findings support a functional association between the Sptlc2–ceramide axis and lipotoxicity, inflammation, and hepatocellular damage.

Mitochondrial dysfunction is widely regarded as a central event in NAFLD progression, linking lipid overload to oxidative stress, impaired energy metabolism, and cell death.^38^ Previous studies have suggested that ceramides may contribute to disruption of mitochondrial membrane homeostasis, amplification of ROS and defective oxidative metabolism.^39^, ^40^ Consistent with this framework, PA treatment increased cytosolic Ca^2+^ and ROS, reduced OCR, increased ECAR, decreased mitochondrial membrane potential, enhanced mPTP opening and promoted VDAC1 oligomerization. Sptlc2 knockdown shifted these parameters toward a less‐injured state, whereas C2‐ceramide treatment moved them back toward injury. TEM observations at the cellular level supported the same overall pattern: PA induced mitochondrial swelling, cristae disruption and vacuolar change, these abnormalities were partially alleviated by Sptlc2 knockdown, and were exacerbated again following C2‐ceramide treatment. Although these data do not define a single linear mitochondrial mechanism, they support a link between ceramide accumulation, loss of mitochondrial homeostasis and hepatocellular injury.

Notably, the relationships observed here between ceramide accumulation, oxidative stress and mitochondrial dysfunction are broadly consistent with findings from obese NAFLD and high‐fat diet models, in which these processes have likewise been associated with liver injury.^41^, ^42^ The advance of the present study is that it places these molecular and cellular relationships in a nonobese experimental context and connects liver‐targeted Sptlc2 knockdown, reduced long‐chain ceramides, and concordant improvement in tissue and cellular phenotypes. At the same time, the data are insufficient to claim that a mechanism specific to nonobese fatty liver has been definitively established, as no obesity‐inducing dietary control was included for direct comparison with obesity‐associated models.

This study also has several limitations. First, the absence of an obesity‐inducing dietary control limits direct assessment of disease specificity. Second, although TEM was added in cell experiments, ultrastructural validation at the liver tissue level remains lacking. Third, although the in vivo knockdown data are supported by liver‐targeted delivery and hepatocyte‐associated changes in Sptlc2 signaling, systematic cell‐type‐specific validation has not yet been completed. Fourth, the in vitro knockdown effect was moderate, and validation was focused primarily at the protein level. Fifth, the clinical cohort was small, serum measurements only were available, and the groups were not balanced for age or sex. Finally, C2‐ceramide served as a functional rescue condition but cannot be regarded as a full physiological surrogate for endogenous long‐chain ceramides. Within these boundaries, the present data support an association between Sptlc2 upregulation, long‐chain ceramide accumulation, and a hepatic phenotype characterized by steatosis, oxidative stress, apoptosis, inflammation, and mitochondrial perturbation.

## 5 CONCLUSION

In summary, integrated proteomic and metabolomic analyses in a DFS‐induced nonobese NAFLD model identified Sptlc2 as a candidate regulator associated with sphingolipid dysregulation. Increased Sptlc2 expression was accompanied by long‐chain ceramide accumulation, hepatic lipotoxic injury, inflammatory activation, and mitochondrial functional impairment. Functional experiments further showed that Sptlc2 knockdown reduced ceramide accumulation, lipid deposition, oxidative stress, inflammatory responses, and hepatocyte injury, whereas C2‐ceramide rescue partially reversed these changes. Together, these findings support a functional role of the Sptlc2–ceramide axis in the pathological changes observed in this model.

This study extends the mechanistic framework of nonobese NAFLD in the DFS model and suggests that the Sptlc2–ceramide pathway may represent a potential target for further investigation. Several limitations should be noted. The present findings were obtained primarily in a rat model and primary rat hepatocytes, and species differences may limit direct extrapolation to human disease. The long‐term safety and biological relevance of exogenous C2‐ceramide exposure also require further evaluation. In addition, because a direct obesogenic‐diet comparator was not included, the relative specificity of this pathway across the obese and obese NAFLD spectrum remains to be established. Future studies incorporating larger clinical cohorts, human liver‐based models, and direct comparisons across disease subtypes will be required to further define the biological and therapeutic relevance of Sptlc2 targeting.

## AUTHOR CONTRIBUTIONS

Li‐Jun Xue, Guang‐Yan Yao, Fei‐Fei Fan, Kai‐Min Li, and Wen‐Ming Wu made significant contributions to this work. Li‐Jun Xue was responsible for conceptualization, and writing the original draft. Guang‐Yan Yao conducted data curation, and formal analysis. Fei‐Fei Fan contributed to investigation and methodology. Kai‐Min Li handled validation, and visualization. Wen‐Ming Wu provided supervision, and contributed to writing review and editing. All authors reviewed and approved the final manuscript.

## CONFLICT OF INTEREST STATEMENT

The authors declare no conflicts of interest.

## ETHICS STATEMENT

This study was approved by the Clinical Ethics Committee of Jinan Central Hospital Affiliated to Shandong First Medical University (No. 20250603001). All animal experiments were approved by the Animal Ethics Committee of Jinan Central Hospital Affiliated to Shandong First Medical University (No. JNCH2024‐059).

## Supporting information

Supporting Information S1

Supporting Information S2

## Data Availability

All data generated or analyzed during this study are included in this article and in Supporting Information S1 and S2. Further inquiries can be directed to the corresponding author.

## References

[1] Zhang, J. , X. Huang , L. Dong , Y. Yang , and D. Kong . 2023. “Epidemiology of Lean/Non‐Obese Nonalcoholic Fatty Liver Disease in China.” Saudi Medical Journal 44(9): 848–863. 10.15537/smj.2023.44.9.20230021. 37717964 PMC10505295

[2] Abbas, Z. , S. Nazir , S. Maqbool , M. Kumar , D. P. Gazder , and S. A. Samejo . 2024. “Characterizing Nonalcoholic Fatty Liver Disease (NAFLD) in Lean Individuals at a Tertiary Care Hospital: A Cross‐Sectional Study.” Euroasian Journal of Hepato‐Gastroenterology 14(2): 198–204. 10.5005/jp-journals-10018-1452. 39802861 PMC11714105

[3] Chan, W.‐K. 2023. “Comparison Between Obese and Non‐Obese Nonalcoholic Fatty Liver Disease.” Clinical and Molecular Hepatology 29(Suppl): S58–S67. 10.3350/cmh.2022.0350. 36472052 PMC10029940

[4] Jang, H. , and W. Kim . 2023. “Non‐Obese or Lean Nonalcoholic Fatty Liver Disease Matters, but Is It Preventable or Inevitable in Light of Its Risk Factors?” Clinical and Molecular Hepatology 29(2): 381–383. 10.3350/cmh.2023.0088. 36891605 PMC10121275

[5] Patel, A. H. , D. Peddu , S. Amin , M. I. Elsaid , C. D. Minacapelli , T.‐M. Chandler , C. Catalano , and V. K. Rustgi . 2022. “Nonalcoholic Fatty Liver Disease in Lean/Nonobese and Obese Individuals: A Comprehensive Review on Prevalence, Pathogenesis, Clinical Outcomes, and Treatment.” Journal of Clinical and Translational Hepatology 11: 502. 10.14218/jcth.2022.00204. 36643037 PMC9817050

[6] Yari, Z. , D. Fotros , and A. Hekmatdoost . 2023. “Comparison of Cardiometabolic Risk Factors Between Obese and Non‐Obese Patients with Nonalcoholic Fatty Liver Disease.” Scientific Reports 13(1): 14531. 10.1038/s41598-023-41893-w. 37666894 PMC10477254

[7] Svegliati‐Baroni, G. , I. Pierantonelli , P. Torquato , R. Marinelli , C. Ferreri , C. Chatgilialoglu , D. Bartolini , and F. Galli . 2019. “Lipidomic Biomarkers and Mechanisms of Lipotoxicity in Non‐Alcoholic Fatty Liver Disease.” Free Radical Biology and Medicine 144: 293–309. 10.1016/j.freeradbiomed.2019.05.029. 31152791

[8] Yu, X.‐D. , and J.‐W. Wang . 2022. “Ceramide De Novo Synthesis in Non‐Alcoholic Fatty Liver Disease: Pathogenic Mechanisms and Therapeutic Perspectives.” Biochemical Pharmacology 202: 115157. 10.1016/j.bcp.2022.115157. 35777449

[9] Ashraf, N. U. , and T. A. Sheikh . 2015. “Endoplasmic Reticulum Stress and Oxidative Stress in the Pathogenesis of Non‐Alcoholic Fatty Liver Disease.” Free Radical Research 49(12): 1405–1418. 10.3109/10715762.2015.1078461. 26223319

[10] Zhang, B. , M. Li , Y. Zou , H. Guo , B. Zhang , C. Xia , H. Zhang , W. Yang , and C. Xu . 2019. “NFκB/Orai1 Facilitates Endoplasmic Reticulum Stress by Oxidative Stress in the Pathogenesis of Non‐Alcoholic Fatty Liver Disease.” Frontiers in Cell and Developmental Biology 7: 202. 10.3389/fcell.2019.00202. 31632961 PMC6783633

[11] Zhang, B. , M. Li , Y. Zou , H. Guo , B. Zhang , C. Xia , H. Zhang , W. Yang , and C. Xu . 2019. “Corrigendum: Nfκb/Orai1 Facilitates Endoplasmic Reticulum Stress by Oxidative Stress in the Pathogenesis of Non‐Alcoholic Fatty Liver Disease.” Frontiers in Cell and Developmental Biology 7: 290. 10.3389/fcell.2019.00290. 31815116 PMC6886368

[12] Tanase, D. M. , E. M. Gosav , D. Petrov , A. E. Jucan , C. M. Lacatusu , M. Floria , C. C. Tarniceriu , C. F. Costea , M. Ciocoiu , and C. Rezus . 2021. “Involvement of Ceramides in Non‐Alcoholic Fatty Liver Disease (NAFLD) Atherosclerosis (ATS) Development: Mechanisms and Therapeutic Targets.” Diagnostics 11: 2053. 10.3390/diagnostics11112053. 34829402 PMC8621166

[13] Chen, J. , Y. Hao , P. Xu , D. Bian , L. Han , X. Wu , Z. Zhuang , J. Wang , and Y. Luo . 2023. “CerS5 Deficiency Promotes Liver Fibrosis Development in Non‐Alcoholic Fatty Liver Disease.” Biochemical and Biophysical Research Communications 667: 120–126. 10.1016/j.bbrc.2023.05.027. 37216827

[14] Zhu, C. , Q. Huai , X. Zhang , H. Dai , X. Li , and H. Wang . 2023. “Insights Into the Roles and Pathomechanisms of Ceramide and Sphigosine‐1‐Phosphate in Nonalcoholic Fatty Liver Disease.” International Journal of Biological Sciences 19(1): 311–330. 10.7150/ijbs.78525. 36594091 PMC9760443

[15] Luukkonen, P. K. , Y. Zhou , S. Sädevirta , M. Leivonen , J. Arola , M. Orešič , T. Hyötyläinen , and H. Yki‐Järvinen . 2016. “Hepatic Ceramides Dissociate Steatosis and Insulin Resistance in Patients with Non‐Alcoholic Fatty Liver Disease.” Journal of Hepatology 64(5): 1167–1175. 10.1016/j.jhep.2016.01.002. 26780287

[16] Chaurasia, B. , V. A. Kaddai , G. I. Lancaster , D. C. Henstridge , S. Sriram , D. L. A. Galam , V. Gopalan , et al. 2016. “Adipocyte Ceramides Regulate Subcutaneous Adipose Browning, Inflammation, and Metabolism.” Cell Metabolism 24(6): 820–834. 10.1016/j.cmet.2016.10.002. 27818258

[17] Teng, W. , Y. Li , M. Du , X. Lei , S. Xie , and F. Ren . 2019. “Sulforaphane Prevents Hepatic Insulin Resistance by Blocking Serine Palmitoyltransferase 3‐Mediated Ceramide Biosynthesis.” Nutrients 11(5): 1185. 10.3390/nu11051185. 31137828 PMC6566605

[18] Lallement, J. , I. Raho , G. Merlen , D. Rainteau , M. Croyal , M. Schiffano , N. Kassis , et al. 2023. “Hepatic Deletion of Serine Palmitoyl Transferase 2 Impairs Ceramide/Sphingomyelin Balance, Bile Acids Homeostasis and Leads to Liver Damage in Mice.” Biochimica et Biophysica Acta (BBA) ‐ Molecular and Cell Biology of Lipids 1868(8): 159333. 10.1016/j.bbalip.2023.159333. 37224999

[19] Roszczyc‐Owsiejczuk, K. , and P. Zabielski . 2021. “Sphingolipids as a Culprit of Mitochondrial Dysfunction in Insulin Resistance and Type 2 Diabetes.” Frontiers in Endocrinology 12: 635175. 10.3389/fendo.2021.635175. 33815291 PMC8013882

[20] Chaurasia, B. , L. Ying , C. L. Talbot , J. A. Maschek , J. Cox , E. H. Schuchman , Y. Hirabayashi , W. L. Holland , and S. A. Summers . 2021. “Ceramides Are Necessary and Sufficient for Diet‐Induced Impairment of Thermogenic Adipocytes.” Molecular Metabolism 45: 101145. 10.1016/j.molmet.2020.101145. 33352310 PMC7807150

[21] Imierska, M. , P. Zabielski , K. Roszczyc‐Owsiejczuk , E. Sokołowska , K. Pogodzińska , I. Kojta , and A. Błachnio‐Zabielska . 2022. “Serine Palmitoyltransferase Gene Silencing Prevents Ceramide Accumulation and Insulin Resistance in Muscles in Mice Fed a High‐Fat Diet.” Cells 11(7): 1123. 10.3390/cells11071123. 35406688 PMC8997855

[22] Tanaka, T. , S. Jiang , H. Hotta , K. Takano , H. Iwanari , K. Sumi , K. Daigo , et al. 2006. “Dysregulated Expression of P1 and P2 Promoter‐Driven Hepatocyte Nuclear Factor‐4α in the Pathogenesis of Human Cancer.” The Journal of Pathology 208(5): 662–672. 10.1002/path.1928. 16400631

[23] Huck, I. , S. Gunewardena , R. Espanol‐Suner , H. Willenbring , and U. Apte . 2019. “Hepatocyte Nuclear Factor 4 Alpha Activation Is Essential for Termination of Liver Regeneration in Mice.” Hepatology 70(2): 666–681. 10.1002/hep.30405. 30520062 PMC6551324

[24] Xue, L.‐J. , J.‐Q. Han , Y.‐C. Zhou , H.‐Y. Peng , T.‐F. Yin , K.‐M. Li , and S.‐K. Yao . 2020. “Untargeted Metabolomics Characteristics of Nonobese Nonalcoholic Fatty Liver Disease Induced by High‐Temperature‐Processed Feed in Sprague‐Dawley Rats.” World Journal of Gastroenterology 26(46): 7299–7311. 10.3748/wjg.v26.i46.7299. 33362385 PMC7739162

[25] Kleiner, D. E. , E. M. Brunt , M. Van Natta , C. Behling , M. J. Contos , O. W. Cummings , L. D. Ferrell , et al. 2005. “Design and Validation of a Histological Scoring System for Nonalcoholic Fatty Liver Disease.” Hepatology 41(6): 1313–1321. 10.1002/hep.20701. 15915461

[26] Brunt, E. M. , D. E. Kleiner , L. A. Wilson , P. Belt , and B. A. Neuschwander‐Tetri . 2011. “Nonalcoholic Fatty Liver Disease (NAFLD) Activity Score and the Histopathologic Diagnosis in NAFLD: Distinct Clinicopathologic Meanings.” Hepatology 53(3): 810–820. 10.1002/hep.24127. 21319198 PMC3079483

[27] Fan, J. G. , L. Wei , and H. Zhuang . 2018. “Guidelines of Prevention and Treatment of Nonalcoholic Fatty Liver Disease (2018, China).” Journal of Digestive Diseases 20(4): 163–173. 10.1111/1751-2980.12685. 30444584

[28] Baral, A. , and P.‐H. Park . 2021. “Leptin Induces Apoptotic and Pyroptotic Cell Death via NLRP3 Inflammasome Activation in Rat Hepatocytes.” International Journal of Molecular Sciences 22: 12589. 10.3390/ijms222212589. 34830465 PMC8622994

[29] Huang, F.‐Q. , H.‐F. Wang , T. Yang , D. Yang , P. Liu , R. N. Alolga , G. Ma , et al. 2025. “Ceramides Increase Mitochondrial Permeabilization to Trigger mtDNA‐Dependent Inflammation in Astrocytes During Brain Ischemia.” Metabolism 166: 156161. 10.1016/j.metabol.2025.156161. 39956315

[30] Heslop, J. A. , C. Rowe , J. Walsh , R. Sison‐Young , R. Jenkins , L. Kamalian , R. Kia , et al. 2016. “Mechanistic Evaluation of Primary Human Hepatocyte Culture Using Global Proteomic Analysis Reveals a Selective Dedifferentiation Profile.” Archives of Toxicology 91(1): 439–452. 10.1007/s00204-016-1694-y. 27039104 PMC5225178

[31] Ito, T. , M. Ishigami , B. Zou , T. Tanaka , H. Takahashi , M. Kurosaki , M. Maeda , et al. 2021. “The Epidemiology of NAFLD and Lean NAFLD in Japan: A Meta‐Analysis with Individual and Forecasting Analysis, 1995–2040.” Hepatology International 15(2): 366–379. 10.1007/s12072-021-10143-4. 33580453

[32] Eslam, M. , F. Chen , and J. George . 2020. “NAFLD in Lean Asians.” Clinical Liver Disease 16(6): 240–243. 10.1002/cld.930. 33489095 PMC7805295

[33] Tang, A. , C. H. Ng , P. H. Phang , K. E. Chan , Y. H. Chin , C. E. Fu , R. W. Zeng , et al. 2023. “Comparative Burden of Metabolic Dysfunction in Lean NAFLD vs Non‐Lean NAFLD – A Systematic Review and Meta‐Analysis.” Clinical Gastroenterology and Hepatology 21(7): 1750–1760.e12. 10.1016/j.cgh.2022.06.029. 35863685

[34] Nikolova‐Karakashian, M. 2018. “Alcoholic and Non‐Alcoholic Fatty Liver Disease: Focus on Ceramide.” Advances in Biological Regulation 70: 40–50. 10.1016/j.jbior.2018.11.004. 30455063

[35] Kasumov, T. , L. Li , M. Li , K. Gulshan , J. P. Kirwan , X. Liu , S. Previs , B. Willard , J. D. Smith , and A. McCullough . 2015. “Ceramide as a Mediator of Non‐Alcoholic Fatty Liver Disease and Associated Atherosclerosis.” PLoS One 10(5): e0126910. 10.1371/journal.pone.0126910. 25993337 PMC4439060

[36] Promrat, K. , L. Longato , J. R. Wands , and S. M. de la Monte . 2011. “Weight Loss Amelioration of Non‐Alcoholic Steatohepatitis Linked to Shifts in Hepatic Ceramide Expression and Serum Ceramide Levels.” Hepatology Research 41(8): 754–762. 10.1111/j.1872-034x.2011.00815.x. 21794038 PMC4550290

[37] Wang, K. , Y. Wei , R. Xu , Y. Li , and C. Mao . 2022. “Manifold Roles of Ceramide Metabolism in Non‐Alcoholic Fatty Liver Disease and Liver Cancer.” Advances in Experimental Medicine and Biology: 157–168. 10.1007/978-981-19-0394-6_11. 35503180

[38] Fromenty, B. , and M. Roden . 2023. “Mitochondrial Alterations in Fatty Liver Diseases.” Journal of Hepatology 78(2): 415–429. 10.1016/j.jhep.2022.09.020. 36209983

[39] Zheng, Y. , S. Wang , J. Wu , and Y. Wang . 2023. “Mitochondrial Metabolic Dysfunction and Non‐Alcoholic Fatty Liver Disease: New Insights from Pathogenic Mechanisms to Clinically Targeted Therapy.” Journal of Translational Medicine 21(1): 510. 10.1186/s12967-023-04367-1. 37507803 PMC10375703

[40] Undamatla, R. , O. G. Fagunloye , J. Chen , L. R. Edmunds , A. Murali , A. Mills , B. Xie , et al. 2023. “Reduced Mitophagy Is an Early Feature of NAFLD and Liver‐Specific PARKIN Knockout Hastens the Onset of Steatosis, Inflammation and Fibrosis.” Scientific Reports 13(1): 7575. 10.1038/s41598-023-34710-x. 37165006 PMC10172344

[41] Kim, G.‐T. , S.‐J. Kim , S.‐H. Park , D. Lee , and T.‐S. Park . 2020. “Hepatic Expression of the Serine Palmitoyltransferase Subunit Sptlc2 Reduces Lipid Droplets in the Liver by Activating VLDL Secretion.” Journal of Lipid and Atherosclerosis 9(2): 291. 10.12997/jla.2020.9.2.291. 32821738 PMC7379091

[42] Zhao, Y. , Y. Zhou , D. Wang , Z. Huang , X. Xiao , Q. Zheng , S. Li , D. Long , and L. Feng . 2023. “Mitochondrial Dysfunction in Metabolic Dysfunction Fatty Liver Disease (MAFLD).” International Journal of Molecular Sciences 24: 17514. 10.3390/ijms242417514. 38139341 PMC10743953

