# Supplementary material for: Ceramide‐mediated mitochondrial dysfunction in nonobese nonalcoholic fatty liver disease: A regulatory role for serine palmitoyltransferase subunit 2
Source: J Cell Commun Signal. 2026 Jun 25;20(2):e70091. doi: 10.1002/ccs3.70091 (PMC13295144; doi:10.1002/ccs3.70091)
Supplement: Supplementary file 1 — Supporting Information S1 [file CCS3-20-e70091-s001.docx]

##
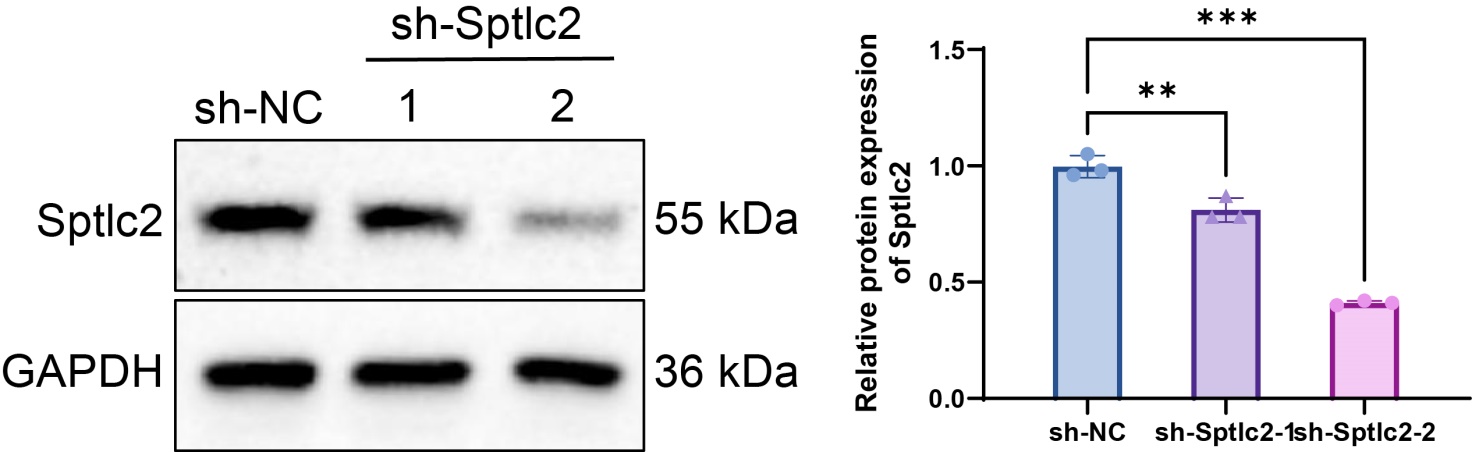


## Figure S1. Screening of sh-Sptlc2 constructs in primary rat hepatocytes

**Note:** Western blot analysis of **Sptlc2** protein expression and corresponding quantification in the **sh-NC, sh-Sptlc2-1** and **sh-Sptlc2-2** groups, used for selection of the construct applied in subsequent in vitro experiments. Data are presented as **mean ± SD**. Statistical significance is indicated in the panels; p < 0.05, *** p < 0.001.

##
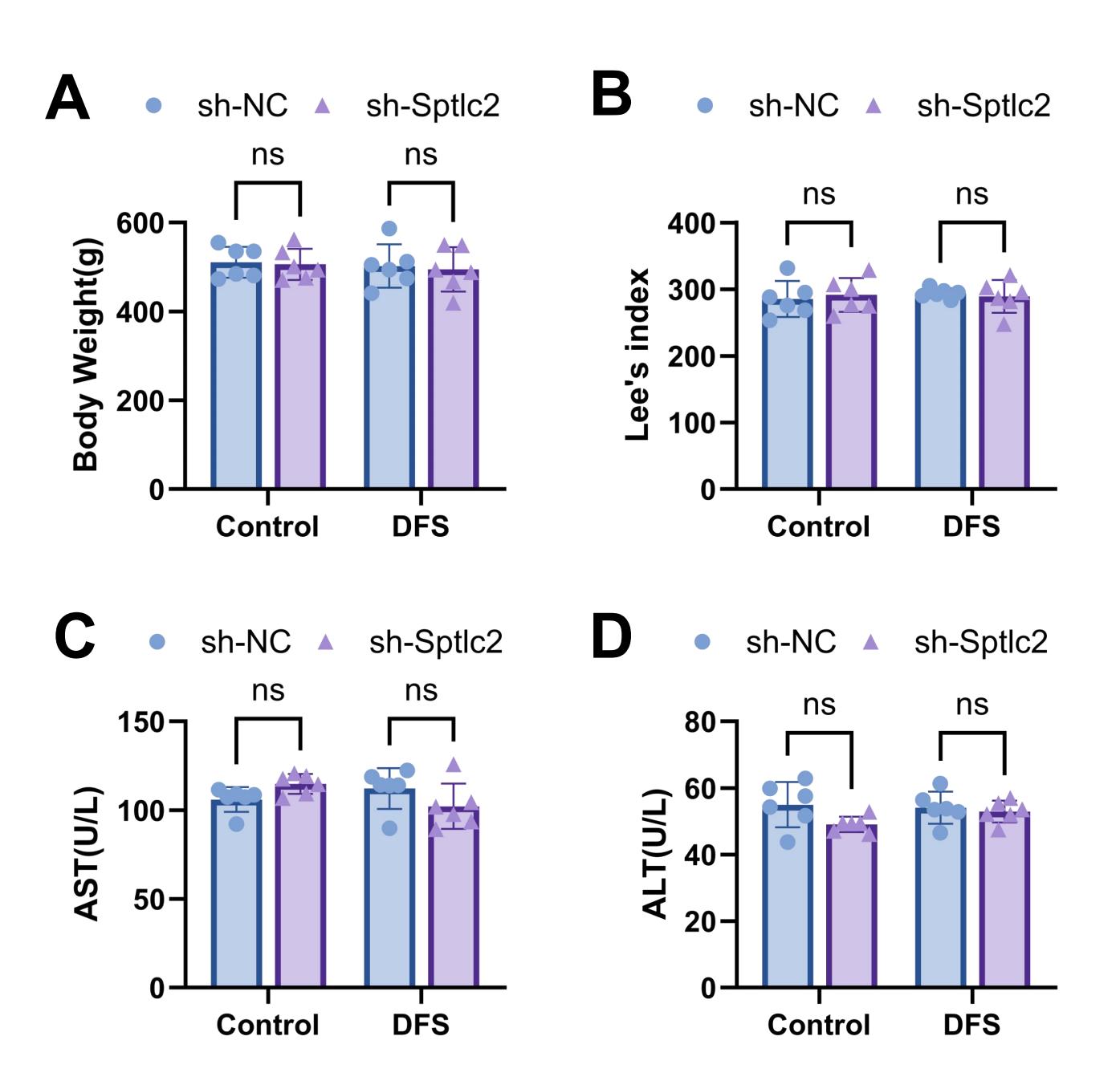


## Figure S2. Body weight, Lee’s index and serum transaminases after liver-directed Sptlc2 knockdown

**Note:** (A, B) Body weight at week 12 (A) and Lee’s index (B) in each group. (C, D) Serum AST (C) and ALT (D) levels in each group. Data are presented as **mean ± SD**, **n = 6**. Statistical significance is indicated in the panels; ns, not significant.

##
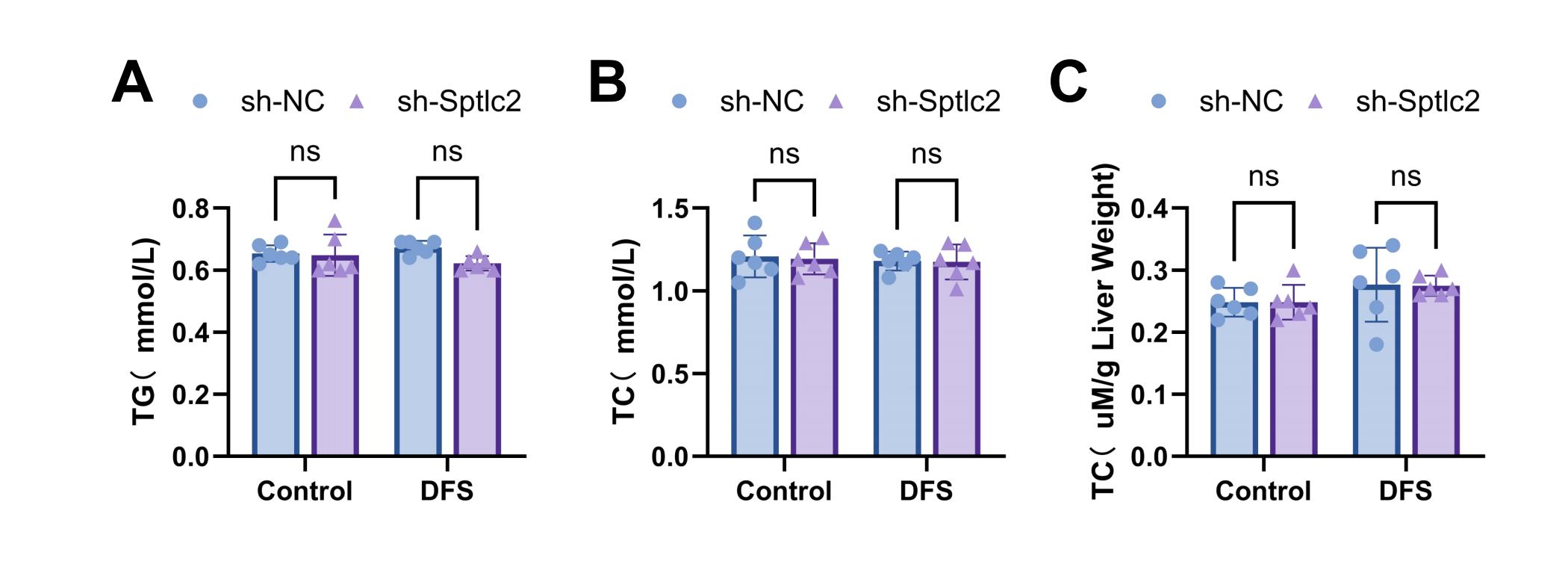


## Figure S3. Serum and hepatic neutral lipid-related indices after Sptlc2 knockdown

**Note:** (A) Serum TG levels in each group. (B) Serum TC levels in each group. (C) Hepatic TC levels in each group. Data are presented as **mean ± SD**, **n = 6**. Statistical significance is indicated in the panels; ns, not significant.

**Table S1. Clinical characteristics of enrolled participants.**

| **Variable** | **Non-obese NAFLD (n=9)** | **Non-obese Healthy controls (n=9)** |
| --- | --- | --- |
| Sex (Male/Female) | 4 / 5 | 1 / 8 |
| Age (years) | 57.22 ± 7.83 | 37.22 ± 4.57 |
| BMI (kg/m²) | 22.57 ± 1.07 | 20.68 ± 1.52 |
| AST (U/L) | 19.34 ± 4.09 | 16.56 ± 2.74 |
| ALT (U/L) | 17.22 ± 2.84 | 13.94 ± 3.68 |
| TG (mmol/L) | 1.02 ± 0.15 | 0.82 ± 0.19 |
| TC (mmol/L) | 4.71 ± 0.82 | 4.45 ± 0.51 |
| FPG (mmol/L) | 4.92 ± 0.21 | 4.69 ± 0.29 |
| Blood sampling | Fasting | Fasting |

**Table S2. Sptlc2 interference sequence.**

| **Targets** | **Sequences** |
| --- | --- |
| sh- Sptlc2-1 (Rat) | GCTGAAGTTCTCAAGACATAT |
| sh- Sptlc2-2 (Rat) | TGCAGCACTCGCCAGGAAATT |
| sh-NC | 5'-CCTAAGGTTAAGTCGCCCTCG-3' |

**Table S3. RT-qPCR** **primer sequence.**

| **Gene ID** | **primer sequence** |
| --- | --- |
| TNF-α-F | GGAGGGAGAACAGCAACTCC |
| TNF-α-R | GCCAGTGTATGAGAGGGACG |
| GAPDH-F | GTACAACTCAGGTTCCGGGG |
| GAPDH-R | TACGGCCAAATCCGTTCACA |
| IL-1β-F | GACTTCACCATGGAACCCGT |
| IL-1β-R | GGAGACTGCCCATTCTCGAC |
| IL-6-F | CACTTCACAAGTCGGAGGCT |
| IL-6-R | TCTGACAGTGCATCATCGCT |

Note: F: Forward; R: Reverse.

**Table S4. Detailed information on instruments used in this study.**

| **Section / Application** | **Instrument** | **Manufacturer** | **Model / Version** | **Purpose** |
| --- | --- | --- | --- | --- |
| Serum and liver biochemistry | Automated biochemical analyzer | Toshiba | *TK-40FR* | Measurement of ALT, AST, TG, and TC |
| Histology / immunofluorescence / TUNEL / BODIPY imaging | Upright slide scanner microscope | Olympus | *VS120* | Acquisition of H&E, immunofluorescence, TUNEL, and BODIPY images |
| Protein quantification for proteomics | Microplate reader | Molecular Devices | *SPECTRAMAX i3x* | Measurement of absorbance at 562 nm in BCA assay |
| High-pH peptide fractionation | Ultra-high-performance liquid chromatography system | Thermo Fisher Scientific | *Vanquish F UHPLC* | High-pH reverse-phase fractionation of peptides |
| Proteomics LC system | Nano-liquid chromatography system | Thermo Fisher Scientific | *EASY-nLC 1200* | Peptide separation before mass spectrometry |
| Proteomics mass spectrometry | Orbitrap mass spectrometer | Thermo Fisher Scientific | *Q Exactive HF-X* | DDA and DIA proteomic data acquisition |
| Peptide concentration | Vacuum concentrator | - | *SpeedVac* | Concentration of desalted peptides |
| Untargeted metabolomics | Ultra-high-performance liquid chromatography-mass spectrometry system | Thermo Fisher Scientific | *UHPLC-Q Exactive HF-X* | Untargeted metabolomic profiling |
| Untargeted metabolomics column | UPLC column | Waters | *ACQUITY UPLC HSS T3, 100 x 2.1 mm, 1.8 um* | Chromatographic separation for untargeted metabolomics |
| Targeted ceramide analysis | Tandem mass spectrometry system | Shimadzu | *LCMS-8050* | LC-MS/MS measurement of ceramides in serum, liver tissue, and cultured cells |
| Targeted ceramide analysis column | UPLC column | Waters | *ACQUITY UPLC BEH C18, 2.1 x 100 mm, 1.7 um* | Chromatographic separation for LC-MS/MS ceramide analysis |
| Tissue homogenization for metabolomics | Cryogenic tissue grinder | Jingxin | - | Low-temperature homogenization of liver tissue samples |
| Cell apoptosis analysis | Flow cytometer | Beckman Coulter | *CytoFLEX S* | Annexin V-FITC / propidium iodide analysis |
| Mitochondrial bioenergetics | Extracellular flux analyzer | Agilent | *Seahorse XFe analyzer* | Measurement of OCR and ECAR |
| mPTP imaging | Confocal microscope | Zeiss | *LSM 880* | Confocal imaging for mitochondrial permeability transition pore assay |
| Transmission electron microscopy | Transmission electron microscope | - | - | Observation of mitochondrial ultrastructure |
| Western blot imaging | Chemiluminescence imaging system | Tanon | *5200* | Detection and imaging of immunoblot signals |
| RNA quantification | UV-visible spectrophotometer | NanoDrop | *ND-1000* | Measurement of RNA concentration and purity |
| RT-qPCR | Real-time PCR system | Roche Diagnostics | *LightCycler 480* | Quantitative real-time PCR |
| Statistical analysis | Statistical software | GraphPad | *Prism 10.1.2* | Statistical analysis |
| Proteomics database search | Software | Thermo Fisher Scientific | *Proteome Discoverer v2.4* | DDA proteomic database search |
| DIA proteomics processing | Software | Biognosys | *Spectronaut v17* | Spectral library generation and DIA data processing |
| Metabolomics data processing | Software | Waters | *Progenesis QI v2.3* | Peak detection, alignment, and metabolite data processing |
| Pathway enrichment / metabolomics statistics | Online platform | MetaboAnalyst | *Version 6.0* | Metabolite pathway enrichment analysis |
| Statistical computing / differential expression | Software environment | R | - | Differential protein analysis and visualization |
| Functional annotation database | Database | UniProt | *Rattus norvegicus dataset* | Proteomics database search |
| Functional annotation database | Database | GeneCards | - | Retrieval of sphingolipid metabolism-related genes |
| Metabolite annotation database | Database | Human Metabolome Database | *HMDB* | Metabolite annotation |
| Metabolite annotation database | Database | Metlin | - | Metabolite annotation |
| Cloud analysis platform | Online platform | Majorbio | - | Statistical analysis of metabolomics data |

**Table S5 Related genes**

| Gene Symbol | Description | Category | Uniprot ID | Gifts | GC Id | Relevance score | GeneCards Link |  |
| --- | --- | --- | --- | --- | --- | --- | --- | --- |
| PSAP | Prosaposin | Protein Coding | P07602 | 61 | GC10M071816 | 101.30884552002 | https://www.genecards.org/cgi-bin/carddisp.pl?gene=PSAP |  |
| INS | Insulin | Protein Coding | P01308 | 61 | GC11M002159 | 42.8761787414551 | https://www.genecards.org/cgi-bin/carddisp.pl?gene=INS |  |
| APOE | Apolipoprotein E | Protein Coding | P02649 | 62 | GC19P152697 | 42.322021484375 | https://www.genecards.org/cgi-bin/carddisp.pl?gene=APOE |  |
| DEGS1 | Delta 4-Desaturase, Sphingolipid 1 | Protein Coding | O15121 | 55 | GC01P224175 | 38.8923797607422 | https://www.genecards.org/cgi-bin/carddisp.pl?gene=DEGS1 |  |
| PPARG | Peroxisome Proliferator Activated Receptor Gamma | Protein Coding | P37231 | 65 | GC03P012287 | 37.1221199035645 | https://www.genecards.org/cgi-bin/carddisp.pl?gene=PPARG |  |
| MTHFR | Methylenetetrahydrofolate Reductase | Protein Coding | P42898 | 61 | GC01M011785 | 36.8882102966309 | https://www.genecards.org/cgi-bin/carddisp.pl?gene=MTHFR |  |
| CBS | Cystathionine Beta-Synthase | Protein Coding | P35520 | 64 | GC21M043053 | 36.4223594665527 | https://www.genecards.org/cgi-bin/carddisp.pl?gene=CBS |  |
| LDLR | Low Density Lipoprotein Receptor | Protein Coding | P01130 | 64 | GC19P151814 | 34.051872253418 | https://www.genecards.org/cgi-bin/carddisp.pl?gene=LDLR |  |
| ADIPOQ | Adiponectin, C1Q And Collagen Domain Containing | Protein Coding | Q15848 | 59 | GC03P186842 | 33.7952575683594 | https://www.genecards.org/cgi-bin/carddisp.pl?gene=ADIPOQ |  |
| SPTLC1 | Serine Palmitoyltransferase Long Chain Base Subunit 1 | Protein Coding | O15269 | 60 | GC09M121881 | 33.7281074523926 | https://www.genecards.org/cgi-bin/carddisp.pl?gene=SPTLC1 |  |
| DEGS2 | Delta 4-Desaturase, Sphingolipid 2 | Protein Coding | Q6QHC5 | 48 | GC14M100143 | 32.6701889038086 | https://www.genecards.org/cgi-bin/carddisp.pl?gene=DEGS2 |  |
| SFTPB | Surfactant Protein B | Protein Coding | P07988 | 56 | GC02M085657 | 31.9300365447998 | https://www.genecards.org/cgi-bin/carddisp.pl?gene=SFTPB |  |
| NPC1 | NPC Intracellular Cholesterol Transporter 1 | Protein Coding | O15118 | 62 | GC18M023506 | 31.3818817138672 | https://www.genecards.org/cgi-bin/carddisp.pl?gene=NPC1 |  |
| ASAH1 | N-Acylsphingosine Amidohydrolase 1 | Protein Coding | Q13510 | 62 | GC08M018055 | 30.9072589874268 | https://www.genecards.org/cgi-bin/carddisp.pl?gene=ASAH1 |  |
| SPTLC2 | Serine Palmitoyltransferase Long Chain Base Subunit 2 | Protein Coding | O15270 | 61 | GC14M077505 | 30.4249248504639 | https://www.genecards.org/cgi-bin/carddisp.pl?gene=SPTLC2 |  |
| SMPD1 | Sphingomyelin Phosphodiesterase 1 | Protein Coding | P17405 | 61 | GC11P006390 | 30.1421432495117 | https://www.genecards.org/cgi-bin/carddisp.pl?gene=SMPD1 |  |
| GBA1 | Glucosylceramidase Beta 1 | Protein Coding | P04062 | 62 | GC01M167453 | 29.2269096374512 | https://www.genecards.org/cgi-bin/carddisp.pl?gene=GBA1 |  |
| APOA1 | Apolipoprotein A1 | Protein Coding | P02647 | 63 | GC11M116835 | 28.7724685668945 | https://www.genecards.org/cgi-bin/carddisp.pl?gene=APOA1 |  |
| LEP | Leptin | Protein Coding | P41159 | 59 | GC07P128241 | 27.8395652770996 | https://www.genecards.org/cgi-bin/carddisp.pl?gene=LEP |  |
| CETP | Cholesteryl Ester Transfer Protein | Protein Coding | P11597 | 60 | GC16P056961 | 27.6368942260742 | https://www.genecards.org/cgi-bin/carddisp.pl?gene=CETP |  |
| LIPC | Lipase C, Hepatic Type | Protein Coding | P11150 | 59 | GC15P058410 | 27.3936595916748 | https://www.genecards.org/cgi-bin/carddisp.pl?gene=LIPC |  |
| CYP1A1 | Cytochrome P450 Family 1 Subfamily A Member 1 | Protein Coding | P04798 | 60 | GC15M074719 | 26.8137149810791 | https://www.genecards.org/cgi-bin/carddisp.pl?gene=CYP1A1 |  |
| INSR | Insulin Receptor | Protein Coding | P06213 | 67 | GC19M007112 | 26.7395210266113 | https://www.genecards.org/cgi-bin/carddisp.pl?gene=INSR |  |
| LPIN1 | Lipin 1 | Protein Coding | Q14693 | 59 | GC02P011677 | 26.406982421875 | https://www.genecards.org/cgi-bin/carddisp.pl?gene=LPIN1 |  |
| ABCA1 | ATP Binding Cassette Subfamily A Member 1 | Protein Coding | O95477 | 62 | GC09M104781 | 26.2072486877441 | https://www.genecards.org/cgi-bin/carddisp.pl?gene=ABCA1 |  |
| GLA | Galactosidase Alpha | Protein Coding | P06280 | 62 | GC0XM101393 | 25.91672707 | https://www.genecards.org/cgi-bin/carddisp.pl?gene=GLA |  |
| ETFA | Electron Transfer Flavoprotein Subunit Alpha | Protein Coding | P13804 | 58 | GC15M162854 | 25.8727989196777 | https://www.genecards.org/cgi-bin/carddisp.pl?gene=ETFA |  |
| HFE | Homeostatic Iron Regulator | Protein Coding | Q30201 | 58 | GC06P026087 | 25.7105865478516 | https://www.genecards.org/cgi-bin/carddisp.pl?gene=HFE |  |
| CYP2B6 | Cytochrome P450 Family 2 Subfamily B Member 6 | Protein Coding | P20813 | 59 | GC19P040991 | 25.6023044586182 | https://www.genecards.org/cgi-bin/carddisp.pl?gene=CYP2B6 |  |
| FA2H | Fatty Acid 2-Hydroxylase | Protein Coding | Q7L5A8 | 58 | GC16M074712 | 25.5301628112793 | https://www.genecards.org/cgi-bin/carddisp.pl?gene=FA2H |  |
| ORMDL3 | ORMDL Sphingolipid Biosynthesis Regulator 3 | Protein Coding | Q8N138 | 49 | GC17M039921 | 25.4341430664063 | https://www.genecards.org/cgi-bin/carddisp.pl?gene=ORMDL3 |  |
| ECHS1 | Enoyl-CoA Hydratase, Short Chain 1 | Protein Coding | P30084 | 59 | GC10M133362 | 24.727367401123 | https://www.genecards.org/cgi-bin/carddisp.pl?gene=ECHS1 |  |
| ACAT1 | Acetyl-CoA Acetyltransferase 1 | Protein Coding | P24752 | 63 | GC11P109833 | 24.6588439941406 | https://www.genecards.org/cgi-bin/carddisp.pl?gene=ACAT1 |  |
| CYP17A1 | Cytochrome P450 Family 17 Subfamily A Member 1 | Protein Coding | P05093 | 62 | GC10M102830 | 24.6445426940918 | https://www.genecards.org/cgi-bin/carddisp.pl?gene=CYP17A1 |  |
| IL6 | Interleukin 6 | Protein Coding | P05231 | 64 | GC07P022725 | 24.2342071533203 | https://www.genecards.org/cgi-bin/carddisp.pl?gene=IL6 |  |
| CYP19A1 | Cytochrome P450 Family 19 Subfamily A Member 1 | Protein Coding | P11511 | 61 | GC15M051208 | 24.0346202850342 | https://www.genecards.org/cgi-bin/carddisp.pl?gene=CYP19A1 |  |
| DDC | Dopa Decarboxylase | Protein Coding | P20711 | 63 | GC07M050458 | 23.85498047 | https://www.genecards.org/cgi-bin/carddisp.pl?gene=DDC |  |
| PPARA | Peroxisome Proliferator Activated Receptor Alpha | Protein Coding | Q07869 | 57 | GC22P046150 | 23.7896366119385 | https://www.genecards.org/cgi-bin/carddisp.pl?gene=PPARA |  |
| TNF | Tumor Necrosis Factor | Protein Coding | P01375 | 65 | GC06P181893 | 23.6547584533691 | https://www.genecards.org/cgi-bin/carddisp.pl?gene=TNF |  |
| PCSK9 | Proprotein Convertase Subtilisin/Kexin Type 9 | Protein Coding | Q8NBP7 | 62 | GC01P055039 | 23.5710525512695 | https://www.genecards.org/cgi-bin/carddisp.pl?gene=PCSK9 |  |
| GLB1 | Galactosidase Beta 1 | Protein Coding | P16278 | 62 | GC03M032963 | 23.4337196350098 | https://www.genecards.org/cgi-bin/carddisp.pl?gene=GLB1 |  |
| ABCG8 | ATP Binding Cassette Subfamily G Member 8 | Protein Coding | Q9H221 | 54 | GC02P045317 | 23.4115428924561 | https://www.genecards.org/cgi-bin/carddisp.pl?gene=ABCG8 |  |
| GALC | Galactosylceramidase | Protein Coding | P54803 | 58 | GC14M087837 | 23.2634563446045 | https://www.genecards.org/cgi-bin/carddisp.pl?gene=GALC |  |
| ASS1 | Argininosuccinate Synthase 1 | Protein Coding | P00966 | 62 | GC09P130444 | 23.1214752197266 | https://www.genecards.org/cgi-bin/carddisp.pl?gene=ASS1 |  |
| CFTR | CF Transmembrane Conductance Regulator | Protein Coding | P13569 | 66 | GC07P117287 | 23.1125679016113 | https://www.genecards.org/cgi-bin/carddisp.pl?gene=CFTR |  |
| ABCG5 | ATP Binding Cassette Subfamily G Member 5 | Protein Coding | Q9H222 | 58 | GC02M043806 | 23.0829830169678 | https://www.genecards.org/cgi-bin/carddisp.pl?gene=ABCG5 |  |
| XDH | Xanthine Dehydrogenase | Protein Coding | P47989 | 60 | GC02M031334 | 22.9091033935547 | https://www.genecards.org/cgi-bin/carddisp.pl?gene=XDH |  |
| ARSA | Arylsulfatase A | Protein Coding | P15289 | 60 | GC22M050622 | 22.836124420166 | https://www.genecards.org/cgi-bin/carddisp.pl?gene=ARSA |  |
| ORMDL1 | ORMDL Sphingolipid Biosynthesis Regulator 1 | Protein Coding | Q9P0S3 | 44 | GC02M189764 | 22.788402557373 | https://www.genecards.org/cgi-bin/carddisp.pl?gene=ORMDL1 |  |
| GM2A | Ganglioside GM2 Activator | Protein Coding | P17900 | 55 | GC05P151212 | 22.3340225219727 | https://www.genecards.org/cgi-bin/carddisp.pl?gene=GM2A |  |
| AKT1 | AKT Serine/Threonine Kinase 1 | Protein Coding | P31749 | 66 | GC14M104769 | 22.2537937164307 | https://www.genecards.org/cgi-bin/carddisp.pl?gene=AKT1 |  |
| AGXT | Alanine--Glyoxylate Aminotransferase | Protein Coding | P21549 | 58 | GC02P240868 | 22.2354297637939 | https://www.genecards.org/cgi-bin/carddisp.pl?gene=AGXT |  |
| GAA | Alpha Glucosidase | Protein Coding | P10253 | 64 | GC17P080101 | 22.2301692962646 | https://www.genecards.org/cgi-bin/carddisp.pl?gene=GAA |  |
| APOA5 | Apolipoprotein A5 | Protein Coding | Q6Q788 | 55 | GC11M116789 | 22.167064666748 | https://www.genecards.org/cgi-bin/carddisp.pl?gene=APOA5 |  |
| G6PD | Glucose-6-Phosphate Dehydrogenase | Protein Coding | P11413 | 62 | GC0XM154625 | 22.1643314361572 | https://www.genecards.org/cgi-bin/carddisp.pl?gene=G6PD |  |
| SGPL1 | Sphingosine-1-Phosphate Lyase 1 | Protein Coding | O95470 | 60 | GC10P070815 | 21.8639221191406 | https://www.genecards.org/cgi-bin/carddisp.pl?gene=SGPL1 |  |
| ORMDL2 | ORMDL Sphingolipid Biosynthesis Regulator 2 | Protein Coding | Q53FV1 | 42 | GC12P075398 | 21.7952098846436 | https://www.genecards.org/cgi-bin/carddisp.pl?gene=ORMDL2 |  |
| CAV1 | Caveolin 1 | Protein Coding | Q03135 | 61 | GC07P116524 | 21.7156524658203 | https://www.genecards.org/cgi-bin/carddisp.pl?gene=CAV1 |  |
| FADS1 | Fatty Acid Desaturase 1 | Protein Coding | O60427 | 55 | GC11M061799 | 21.6700191497803 | https://www.genecards.org/cgi-bin/carddisp.pl?gene=FADS1 |  |
| PTEN | Phosphatase And Tensin Homolog | Protein Coding | P60484 | 65 | GC10P124078 | 21.5328063964844 | https://www.genecards.org/cgi-bin/carddisp.pl?gene=PTEN |  |
| HEXA | Hexosaminidase Subunit Alpha | Protein Coding | P06865 | 60 | GC15M072340 | 20.928581237793 | https://www.genecards.org/cgi-bin/carddisp.pl?gene=HEXA |  |
| TH | Tyrosine Hydroxylase | Protein Coding | P07101 | 63 | GC11M002163 | 20.865650177002 | https://www.genecards.org/cgi-bin/carddisp.pl?gene=TH |  |
| SUMF1 | Sulfatase Modifying Factor 1 | Protein Coding | Q8NBK3 | 54 | GC03M003700 | 20.7950572967529 | https://www.genecards.org/cgi-bin/carddisp.pl?gene=SUMF1 |  |
| PYGM | Glycogen Phosphorylase, Muscle Associated | Protein Coding | P11217 | 60 | GC11M064746 | 20.7846946716309 | https://www.genecards.org/cgi-bin/carddisp.pl?gene=PYGM |  |
| MTOR | Mechanistic Target Of Rapamycin Kinase | Protein Coding | P42345 | 68 | GC01M011106 | 20.7708492279053 | https://www.genecards.org/cgi-bin/carddisp.pl?gene=MTOR |  |
| LCAT | Lecithin-Cholesterol Acyltransferase | Protein Coding | P04180 | 60 | GC16M067939 | 20.749490737915 | https://www.genecards.org/cgi-bin/carddisp.pl?gene=LCAT |  |
| LIPA | Lipase A, Lysosomal Acid Type | Protein Coding | P38571 | 62 | GC10M089213 | 20.6807079315186 | https://www.genecards.org/cgi-bin/carddisp.pl?gene=LIPA |  |
| SOD1 | Superoxide Dismutase 1 | Protein Coding | P00441 | 66 | GC21P031659 | 20.5435981750488 | https://www.genecards.org/cgi-bin/carddisp.pl?gene=SOD1 |  |
| MFSD2B | MFSD2 Lysolipid Transporter B, Sphingolipid | Protein Coding | A6NFX1 | 41 | GC02P024010 | 20.5238800048828 | https://www.genecards.org/cgi-bin/carddisp.pl?gene=MFSD2B |  |
| SPTLC3 | Serine Palmitoyltransferase Long Chain Base Subunit 3 | Protein Coding | Q9NUV7 | 51 | GC20P013008 | 20.4235134124756 | https://www.genecards.org/cgi-bin/carddisp.pl?gene=SPTLC3 |  |
| NPC2 | NPC Intracellular Cholesterol Transporter 2 | Protein Coding | P61916 | 56 | GC14M074476 | 20.1793937683105 | https://www.genecards.org/cgi-bin/carddisp.pl?gene=NPC2 |  |
| PNPO | Pyridoxamine 5'-Phosphate Oxidase | Protein Coding | Q9NVS9 | 59 | GC17P047941 | 20.1302947998047 | https://www.genecards.org/cgi-bin/carddisp.pl?gene=PNPO |  |
| VDR | Vitamin D Receptor | Protein Coding | P11473 | 61 | GC12M047841 | 20.0594234466553 | https://www.genecards.org/cgi-bin/carddisp.pl?gene=VDR |  |
| CRP | C-Reactive Protein | Protein Coding | P02741 | 58 | GC01M167583 | 19.9584579467773 | https://www.genecards.org/cgi-bin/carddisp.pl?gene=CRP |  |
| MTTP | Microsomal Triglyceride Transfer Protein | Protein Coding | P55157 | 57 | GC04P099563 | 19.862247467041 | https://www.genecards.org/cgi-bin/carddisp.pl?gene=MTTP |  |
| ALDH3A2 | Aldehyde Dehydrogenase 3 Family Member A2 | Protein Coding | P51648 | 59 | GC17P019648 | 19.8387908935547 | https://www.genecards.org/cgi-bin/carddisp.pl?gene=ALDH3A2 |  |
| TP53 | Tumor Protein P53 | Protein Coding | P04637 | 66 | GC17M007661 | 19.8309631347656 | https://www.genecards.org/cgi-bin/carddisp.pl?gene=TP53 |  |
| HEXB | Hexosaminidase Subunit Beta | Protein Coding | P07686 | 60 | GC05P074640 | 19.6094970703125 | https://www.genecards.org/cgi-bin/carddisp.pl?gene=HEXB |  |
| BGLAP | Bone Gamma-Carboxyglutamate Protein | Protein Coding | P02818 | 52 | GC01P156242 | 19.5258464813232 | https://www.genecards.org/cgi-bin/carddisp.pl?gene=BGLAP |  |
| SOD2-OT1 | SOD2 Overlapping Transcript 1 | RNA Gene |  | 20 | GC06M159772 | 19.5040302276611 | https://www.genecards.org/cgi-bin/carddisp.pl?gene=SOD2-OT1 |  |
| NAGLU | N-Acetyl-Alpha-Glucosaminidase | Protein Coding | P54802 | 58 | GC17P154848 | 19.4054679870605 | https://www.genecards.org/cgi-bin/carddisp.pl?gene=NAGLU |  |
| CERS3 | Ceramide Synthase 3 | Protein Coding | Q8IU89 | 53 | GC15M163337 | 19.3990211486816 | https://www.genecards.org/cgi-bin/carddisp.pl?gene=CERS3 |  |
| CYP21A2 | Cytochrome P450 Family 21 Subfamily A Member 2 | Protein Coding | P08686 | 55 | GC06P181915 | 19.3105659484863 | https://www.genecards.org/cgi-bin/carddisp.pl?gene=CYP21A2 |  |
| SPHK1 | Sphingosine Kinase 1 | Protein Coding | Q9NYA1 | 58 | GC17P076376 | 19.2671127319336 | https://www.genecards.org/cgi-bin/carddisp.pl?gene=SPHK1 |  |
| BDNF-AS | BDNF Antisense RNA | RNA Gene |  | 31 | GC11P027466 | 19.2016887664795 | https://www.genecards.org/cgi-bin/carddisp.pl?gene=BDNF-AS |  |
| GBA2 | Glucosylceramidase Beta 2 | Protein Coding | Q9HCG7 | 53 | GC09M035736 | 19.1057510375977 | https://www.genecards.org/cgi-bin/carddisp.pl?gene=GBA2 |  |
| MT-TP | Mitochondrially Encoded TRNA-Pro (CCN) | RNA Gene |  | 19 | GCMTM015957 | 19.0572032928467 | https://www.genecards.org/cgi-bin/carddisp.pl?gene=MT-TP |  |
| PNPLA2 | Patatin Like Domain 2, Triacylglycerol Lipase | Protein Coding | Q96AD5 | 57 | GC11P020269 | 19.0230598449707 | https://www.genecards.org/cgi-bin/carddisp.pl?gene=PNPLA2 |  |
| LDHA | Lactate Dehydrogenase A | Protein Coding | P00338 | 64 | GC11P018394 | 18.9320545196533 | https://www.genecards.org/cgi-bin/carddisp.pl?gene=LDHA |  |
| HIF1A | Hypoxia Inducible Factor 1 Subunit Alpha | Protein Coding | Q16665 | 61 | GC14P061695 | 18.9315662384033 | https://www.genecards.org/cgi-bin/carddisp.pl?gene=HIF1A |  |
| TAT | Tyrosine Aminotransferase | Protein Coding | P17735 | 58 | GC16M071565 | 18.8632545471191 | https://www.genecards.org/cgi-bin/carddisp.pl?gene=TAT |  |
| PLIN1 | Perilipin 1 | Protein Coding | O60240 | 60 | GC15M089664 | 18.7371311187744 | https://www.genecards.org/cgi-bin/carddisp.pl?gene=PLIN1 |  |
| MAOA | Monoamine Oxidase A | Protein Coding | P21397 | 62 | GC0XP043654 | 18.728157043457 | https://www.genecards.org/cgi-bin/carddisp.pl?gene=MAOA |  |
| UGCG | UDP-Glucose Ceramide Glucosyltransferase | Protein Coding | Q16739 | 57 | GC09P111896 | 18.5251350402832 | https://www.genecards.org/cgi-bin/carddisp.pl?gene=UGCG |  |
| EPHX2 | Epoxide Hydrolase 2 | Protein Coding | P34913 | 59 | GC08P027490 | 18.5084609985352 | https://www.genecards.org/cgi-bin/carddisp.pl?gene=EPHX2 |  |
| CTSD | Cathepsin D | Protein Coding | P07339 | 65 | GC11M001752 | 18.5027236938477 | https://www.genecards.org/cgi-bin/carddisp.pl?gene=CTSD |  |
| OAT | Ornithine Aminotransferase | Protein Coding | P04181 | 61 | GC10M124397 | 18.4999752044678 | https://www.genecards.org/cgi-bin/carddisp.pl?gene=OAT |  |
| AKT2 | AKT Serine/Threonine Kinase 2 | Protein Coding | P31751 | 67 | GC19M040230 | 18.4281978607178 | https://www.genecards.org/cgi-bin/carddisp.pl?gene=AKT2 |  |
| GCH1 | GTP Cyclohydrolase 1 | Protein Coding | P30793 | 60 | GC14M054842 | 18.3939895629883 | https://www.genecards.org/cgi-bin/carddisp.pl?gene=GCH1 |  |
| GLDC | Glycine Decarboxylase | Protein Coding | P23378 | 60 | GC09M006522 | 18.3862476348877 | https://www.genecards.org/cgi-bin/carddisp.pl?gene=GLDC |  |
| GOT2 | Glutamic-Oxaloacetic Transaminase 2 | Protein Coding | P00505 | 60 | GC16M058707 | 18.2983837127686 | https://www.genecards.org/cgi-bin/carddisp.pl?gene=GOT2 |  |
| PIK3CA | Phosphatidylinositol-4,5-Bisphosphate 3-Kinase Catalytic Subunit Alpha | Protein Coding | P42336 | 65 | GC03P179148 | 18.2776165008545 | https://www.genecards.org/cgi-bin/carddisp.pl?gene=PIK3CA |  |
| IDUA | Alpha-L-Iduronidase | Protein Coding | P35475 | 58 | GC04P000986 | 18.2234115600586 | https://www.genecards.org/cgi-bin/carddisp.pl?gene=IDUA |  |
| CTSA | Cathepsin A | Protein Coding | P10619 | 60 | GC20P045890 | 18.2035217285156 | https://www.genecards.org/cgi-bin/carddisp.pl?gene=CTSA |  |
| TECR | Trans-2,3-Enoyl-CoA Reductase | Protein Coding | Q9NZ01 | 55 | GC19P014517 | 18.1786670684814 | https://www.genecards.org/cgi-bin/carddisp.pl?gene=TECR |  |
| SCP2 | Sterol Carrier Protein 2 | Protein Coding | P22307 | 59 | GC01P052927 | 18.1680965423584 | https://www.genecards.org/cgi-bin/carddisp.pl?gene=SCP2 |  |
| NEU1 | Neuraminidase 1 | Protein Coding | Q99519 | 59 | GC06M031857 | 18.1174163818359 | https://www.genecards.org/cgi-bin/carddisp.pl?gene=NEU1 |  |
| CAT | Catalase | Protein Coding | P04040 | 64 | GC11P034460 | 18.1088600158691 | https://www.genecards.org/cgi-bin/carddisp.pl?gene=CAT |  |
| GPT | Glutamic--Pyruvic Transaminase | Protein Coding | P24298 | 55 | GC08P144502 | 18.0503902435303 | https://www.genecards.org/cgi-bin/carddisp.pl?gene=GPT |  |
| ACER3 | Alkaline Ceramidase 3 | Protein Coding | Q9NUN7 | 50 | GC11P076860 | 18.0351619720459 | https://www.genecards.org/cgi-bin/carddisp.pl?gene=ACER3 |  |
| ASAH2 | N-Acylsphingosine Amidohydrolase 2 | Protein Coding | Q9NR71 | 54 | GC10M050182 | 17.839729309082 | https://www.genecards.org/cgi-bin/carddisp.pl?gene=ASAH2 |  |
| SREBF1 | Sterol Regulatory Element Binding Transcription Factor 1 | Protein Coding | P36956 | 61 | GC17M017810 | 17.5256214141846 | https://www.genecards.org/cgi-bin/carddisp.pl?gene=SREBF1 |  |
| GALNS | Galactosamine (N-Acetyl)-6-Sulfatase | Protein Coding | P34059 | 61 | GC16M088813 | 17.522216796875 | https://www.genecards.org/cgi-bin/carddisp.pl?gene=GALNS |  |
| HMGCR | 3-Hydroxy-3-Methylglutaryl-CoA Reductase | Protein Coding | P04035 | 61 | GC05P075336 | 17.5195713043213 | https://www.genecards.org/cgi-bin/carddisp.pl?gene=HMGCR |  |
| CERS1 | Ceramide Synthase 1 | Protein Coding | P27544 | 55 | GC19M018868 | 17.5037670135498 | https://www.genecards.org/cgi-bin/carddisp.pl?gene=CERS1 |  |
| HNF1A | HNF1 Homeobox A | Protein Coding | P20823 | 60 | GC12P120978 | 17.4924182891846 | https://www.genecards.org/cgi-bin/carddisp.pl?gene=HNF1A |  |
| TYR | Tyrosinase | Protein Coding | P14679 | 61 | GC11P089177 | 17.4698104858398 | https://www.genecards.org/cgi-bin/carddisp.pl?gene=TYR |  |
| CERS2 | Ceramide Synthase 2 | Protein Coding | Q96G23 | 55 | GC01M150960 | 17.4568786621094 | https://www.genecards.org/cgi-bin/carddisp.pl?gene=CERS2 |  |
| PRKAG2 | Protein Kinase AMP-Activated Non-Catalytic Subunit Gamma 2 | Protein Coding | Q9UGJ0 | 62 | GC07M151556 | 17.4146938323975 | https://www.genecards.org/cgi-bin/carddisp.pl?gene=PRKAG2 |  |
| IRS1 | Insulin Receptor Substrate 1 | Protein Coding | P35568 | 61 | GC02M226731 | 17.3515357971191 | https://www.genecards.org/cgi-bin/carddisp.pl?gene=IRS1 |  |
| AGK | Acylglycerol Kinase | Protein Coding | Q53H12 | 53 | GC07P141551 | 17.3011379241943 | https://www.genecards.org/cgi-bin/carddisp.pl?gene=AGK |  |
| CES1 | Carboxylesterase 1 | Protein Coding | P23141 | 61 | GC16M055836 | 17.2906551361084 | https://www.genecards.org/cgi-bin/carddisp.pl?gene=CES1 |  |
| ABCB11 | ATP Binding Cassette Subfamily B Member 11 | Protein Coding | O95342 | 60 | GC02M168922 | 17.1615505218506 | https://www.genecards.org/cgi-bin/carddisp.pl?gene=ABCB11 |  |
| GPT2 | Glutamic--Pyruvic Transaminase 2 | Protein Coding | Q8TD30 | 58 | GC16P121343 | 17.1301708221436 | https://www.genecards.org/cgi-bin/carddisp.pl?gene=GPT2 |  |
| DGAT1 | Diacylglycerol O-Acyltransferase 1 | Protein Coding | O75907 | 60 | GC08M147388 | 17.1297397613525 | https://www.genecards.org/cgi-bin/carddisp.pl?gene=DGAT1 |  |
| PPARD | Peroxisome Proliferator Activated Receptor Delta | Protein Coding | Q03181 | 59 | GC06P182001 | 17.0601902008057 | https://www.genecards.org/cgi-bin/carddisp.pl?gene=PPARD |  |
| FTO | FTO Alpha-Ketoglutarate Dependent Dioxygenase | Protein Coding | Q9C0B1 | 59 | GC16P121535 | 16.9855976104736 | https://www.genecards.org/cgi-bin/carddisp.pl?gene=FTO |  |
| IDS | Iduronate 2-Sulfatase | Protein Coding | P22304 | 60 | GC0XM149476 | 16.9542865753174 | https://www.genecards.org/cgi-bin/carddisp.pl?gene=IDS |  |
| APP | Amyloid Beta Precursor Protein | Protein Coding | P05067 | 63 | GC21M025880 | 16.948127746582 | https://www.genecards.org/cgi-bin/carddisp.pl?gene=APP |  |
| SI | Sucrase-Isomaltase | Protein Coding | P14410 | 58 | GC03M164978 | 16.8018093109131 | https://www.genecards.org/cgi-bin/carddisp.pl?gene=SI |  |
| ARSB | Arylsulfatase B | Protein Coding | P15848 | 60 | GC05M078777 | 16.7890586853027 | https://www.genecards.org/cgi-bin/carddisp.pl?gene=ARSB |  |
| CTH | Cystathionine Gamma-Lyase | Protein Coding | P32929 | 62 | GC01P070411 | 16.7472171783447 | https://www.genecards.org/cgi-bin/carddisp.pl?gene=CTH |  |
| PLA2G6 | Phospholipase A2 Group VI | Protein Coding | O60733 | 61 | GC22M087757 | 16.7291316986084 | https://www.genecards.org/cgi-bin/carddisp.pl?gene=PLA2G6 |  |
| STS | Steroid Sulfatase | Protein Coding | P08842 | 59 | GC0XP007146 | 16.7059288024902 | https://www.genecards.org/cgi-bin/carddisp.pl?gene=STS |  |
| PHGDH | Phosphoglycerate Dehydrogenase | Protein Coding | O43175 | 62 | GC01P119724 | 16.6110439300537 | https://www.genecards.org/cgi-bin/carddisp.pl?gene=PHGDH |  |
| IL10 | Interleukin 10 | Protein Coding | P22301 | 60 | GC01M206767 | 16.406286239624 | https://www.genecards.org/cgi-bin/carddisp.pl?gene=IL10 |  |
| SGPP1 | Sphingosine-1-Phosphate Phosphatase 1 | Protein Coding | Q9BX95 | 45 | GC14M063684 | 16.3975410461426 | https://www.genecards.org/cgi-bin/carddisp.pl?gene=SGPP1 |  |
| SMPD2 | Sphingomyelin Phosphodiesterase 2 | Protein Coding | O60906 | 54 | GC06P109440 | 16.368803024292 | https://www.genecards.org/cgi-bin/carddisp.pl?gene=SMPD2 |  |
| POMC | Proopiomelanocortin | Protein Coding | P01189 | 61 | GC02M025160 | 16.3592643737793 | https://www.genecards.org/cgi-bin/carddisp.pl?gene=POMC |  |
| SDHB | Succinate Dehydrogenase Complex Iron Sulfur Subunit B | Protein Coding | P21912 | 61 | GC01M022674 | 16.3581981658936 | https://www.genecards.org/cgi-bin/carddisp.pl?gene=SDHB |  |
| SIRT1 | Sirtuin 1 | Protein Coding | Q96EB6 | 62 | GC10P067884 | 16.347864151001 | https://www.genecards.org/cgi-bin/carddisp.pl?gene=SIRT1 |  |
| APOC2 | Apolipoprotein C2 | Protein Coding | P02655 | 57 | GC19P152701 | 16.308910369873 | https://www.genecards.org/cgi-bin/carddisp.pl?gene=APOC2 |  |
| ESR1 | Estrogen Receptor 1 | Protein Coding | P03372 | 67 | GC06P151656 | 16.2104053497314 | https://www.genecards.org/cgi-bin/carddisp.pl?gene=ESR1 |  |
| TF | Transferrin | Protein Coding | P02787 | 64 | GC03P143239 | 16.1888637542725 | https://www.genecards.org/cgi-bin/carddisp.pl?gene=TF |  |
| PPT1 | Palmitoyl-Protein Thioesterase 1 | Protein Coding | P50897 | 60 | GC01M040273 | 16.1783275604248 | https://www.genecards.org/cgi-bin/carddisp.pl?gene=PPT1 |  |
| LDLRAP1 | Low Density Lipoprotein Receptor Adaptor Protein 1 | Protein Coding | Q5SW96 | 52 | GC01P025543 | 16.1161060333252 | https://www.genecards.org/cgi-bin/carddisp.pl?gene=LDLRAP1 |  |
| ABCB1 | ATP Binding Cassette Subfamily B Member 1 | Protein Coding | P08183 | 64 | GC07M087504 | 16.0633735656738 | https://www.genecards.org/cgi-bin/carddisp.pl?gene=ABCB1 |  |
| CLN8 | CLN8 Transmembrane ER And ERGIC Protein | Protein Coding | Q9UBY8 | 52 | GC08P001755 | 15.9242572784424 | https://www.genecards.org/cgi-bin/carddisp.pl?gene=CLN8 |  |
| PYGL | Glycogen Phosphorylase L | Protein Coding | P06737 | 59 | GC14M050857 | 15.8209934234619 | https://www.genecards.org/cgi-bin/carddisp.pl?gene=PYGL |  |
| AHR | Aryl Hydrocarbon Receptor | Protein Coding | P35869 | 61 | GC07P016916 | 15.7506790161133 | https://www.genecards.org/cgi-bin/carddisp.pl?gene=AHR |  |
| CLN3 | CLN3 Lysosomal/Endosomal Transmembrane Protein, Battenin | Protein Coding | Q13286 | 56 | GC16M028466 | 15.7038040161133 | https://www.genecards.org/cgi-bin/carddisp.pl?gene=CLN3 |  |
| F2 | Coagulation Factor II, Thrombin | Protein Coding | P00734 | 63 | GC11P049059 | 15.6864280700684 | https://www.genecards.org/cgi-bin/carddisp.pl?gene=F2 |  |
| SGSH | N-Sulfoglucosamine Sulfohydrolase | Protein Coding | P51688 | 60 | GC17M098179 | 15.6849527359009 | https://www.genecards.org/cgi-bin/carddisp.pl?gene=SGSH |  |
| EGF | Epidermal Growth Factor | Protein Coding | P01133 | 64 | GC04P109912 | 15.6590900421143 | https://www.genecards.org/cgi-bin/carddisp.pl?gene=EGF |  |
| TTR | Transthyretin | Protein Coding | P02766 | 61 | GC18P031557 | 15.649585723877 | https://www.genecards.org/cgi-bin/carddisp.pl?gene=TTR |  |
| ACY1 | Aminoacylase 1 | Protein Coding | Q03154 | 57 | GC03P051983 | 15.5703182220459 | https://www.genecards.org/cgi-bin/carddisp.pl?gene=ACY1 |  |
| MIR7-3HG | MIR7-3 Host Gene | RNA Gene | Q8N6C7 | 34 | GC19P151538 | 15.484748840332 | https://www.genecards.org/cgi-bin/carddisp.pl?gene=MIR7-3HG |  |
| KDSR | 3-Ketodihydrosphingosine Reductase | Protein Coding | Q06136 | 53 | GC18M063327 | 15.4546880722046 | https://www.genecards.org/cgi-bin/carddisp.pl?gene=KDSR |  |
| SLC2A4 | Solute Carrier Family 2 Member 4 | Protein Coding | P14672 | 60 | GC17P153733 | 15.4498062133789 | https://www.genecards.org/cgi-bin/carddisp.pl?gene=SLC2A4 |  |
| RYR1 | Ryanodine Receptor 1 | Protein Coding | P21817 | 62 | GC19P152460 | 15.366382598877 | https://www.genecards.org/cgi-bin/carddisp.pl?gene=RYR1 |  |
| GNPTAB | N-Acetylglucosamine-1-Phosphate Transferase Subunits Alpha And Beta | Protein Coding | Q3T906 | 54 | GC12M101745 | 15.351824760437 | https://www.genecards.org/cgi-bin/carddisp.pl?gene=GNPTAB |  |
| TGFB1 | Transforming Growth Factor Beta 1 | Protein Coding | P01137 | 65 | GC19M041301 | 15.2570667266846 | https://www.genecards.org/cgi-bin/carddisp.pl?gene=TGFB1 |  |
| ACSL4 | Acyl-CoA Synthetase Long Chain Family Member 4 | Protein Coding | O60488 | 60 | GC0XM109624 | 15.256462097168 | https://www.genecards.org/cgi-bin/carddisp.pl?gene=ACSL4 |  |
| NOS3 | Nitric Oxide Synthase 3 | Protein Coding | P29474 | 62 | GC07P165997 | 15.2413988113403 | https://www.genecards.org/cgi-bin/carddisp.pl?gene=NOS3 |  |
| GPD1 | Glycerol-3-Phosphate Dehydrogenase 1 | Protein Coding | P21695 | 56 | GC12P075094 | 15.2339878082275 | https://www.genecards.org/cgi-bin/carddisp.pl?gene=GPD1 |  |
| ABAT | 4-Aminobutyrate Aminotransferase | Protein Coding | P80404 | 58 | GC16P008674 | 15.0817165374756 | https://www.genecards.org/cgi-bin/carddisp.pl?gene=ABAT |  |
| ENPP1 | Ectonucleotide Pyrophosphatase/Phosphodiesterase 1 | Protein Coding | P22413 | 62 | GC06P131808 | 15.0681896209717 | https://www.genecards.org/cgi-bin/carddisp.pl?gene=ENPP1 |  |
| PIK3R1 | Phosphoinositide-3-Kinase Regulatory Subunit 1 | Protein Coding | P27986 | 65 | GC05P068215 | 15.0399370193481 | https://www.genecards.org/cgi-bin/carddisp.pl?gene=PIK3R1 |  |
| FDFT1 | Farnesyl-Diphosphate Farnesyltransferase 1 | Protein Coding | P37268 | 58 | GC08P011795 | 15.0300559997559 | https://www.genecards.org/cgi-bin/carddisp.pl?gene=FDFT1 |  |
| NR1I2 | Nuclear Receptor Subfamily 1 Group I Member 2 | Protein Coding | O75469 | 55 | GC03P119780 | 15.0268898010254 | https://www.genecards.org/cgi-bin/carddisp.pl?gene=NR1I2 |  |
| MAPK1 | Mitogen-Activated Protein Kinase 1 | Protein Coding | P28482 | 65 | GC22M021759 | 14.9554538726807 | https://www.genecards.org/cgi-bin/carddisp.pl?gene=MAPK1 |  |
| IL1B | Interleukin 1 Beta | Protein Coding | P01584 | 60 | GC02M112829 | 14.9487705230713 | https://www.genecards.org/cgi-bin/carddisp.pl?gene=IL1B |  |
| PNPLA3 | Patatin Like Domain 3, 1-Acylglycerol-3-Phosphate O-Acyltransferase | Protein Coding | Q9NST1 | 54 | GC22P043923 | 14.9372329711914 | https://www.genecards.org/cgi-bin/carddisp.pl?gene=PNPLA3 |  |
| LAMP2 | Lysosomal Associated Membrane Protein 2 | Protein Coding | P13473 | 57 | GC0XM120426 | 14.9228429794312 | https://www.genecards.org/cgi-bin/carddisp.pl?gene=LAMP2 |  |
| PON1 | Paraoxonase 1 | Protein Coding | P27169 | 60 | GC07M095297 | 14.8890800476074 | https://www.genecards.org/cgi-bin/carddisp.pl?gene=PON1 |  |
| GALNT2 | Polypeptide N-Acetylgalactosaminyltransferase 2 | Protein Coding | Q10471 | 57 | GC01P230057 | 14.8516283035278 | https://www.genecards.org/cgi-bin/carddisp.pl?gene=GALNT2 |  |
| MCOLN1 | Mucolipin TRP Cation Channel 1 | Protein Coding | Q9GZU1 | 56 | GC19P151636 | 14.8360919952393 | https://www.genecards.org/cgi-bin/carddisp.pl?gene=MCOLN1 |  |
| UGT8 | UDP Glycosyltransferase 8 | Protein Coding | Q16880 | 57 | GC04P114598 | 14.7884531021118 | https://www.genecards.org/cgi-bin/carddisp.pl?gene=UGT8 |  |
| ALOX5 | Arachidonate 5-Lipoxygenase | Protein Coding | P09917 | 62 | GC10P045374 | 14.7192697525024 | https://www.genecards.org/cgi-bin/carddisp.pl?gene=ALOX5 |  |
| GNS | Glucosamine (N-Acetyl)-6-Sulfatase | Protein Coding | P15586 | 57 | GC12M064713 | 14.6708879470825 | https://www.genecards.org/cgi-bin/carddisp.pl?gene=GNS |  |
| PTGS2 | Prostaglandin-Endoperoxide Synthase 2 | Protein Coding | P35354 | 62 | GC01M186671 | 14.5477266311646 | https://www.genecards.org/cgi-bin/carddisp.pl?gene=PTGS2 |  |
| FTCD | Formimidoyltransferase Cyclodeaminase | Protein Coding | O95954 | 53 | GC21M054955 | 14.4092483520508 | https://www.genecards.org/cgi-bin/carddisp.pl?gene=FTCD |  |
| ALOX12B | Arachidonate 12-Lipoxygenase, 12R Type | Protein Coding | O75342 | 55 | GC17M096230 | 14.3844976425171 | https://www.genecards.org/cgi-bin/carddisp.pl?gene=ALOX12B |  |
| CLN6 | CLN6 Transmembrane ER Protein | Protein Coding | Q9NWW5 | 50 | GC15M068206 | 14.2593507766724 | https://www.genecards.org/cgi-bin/carddisp.pl?gene=CLN6 |  |
| ABCG2 | ATP Binding Cassette Subfamily G Member 2 (JR Blood Group) | Protein Coding | Q9UNQ0 | 62 | GC04M088090 | 14.1897325515747 | https://www.genecards.org/cgi-bin/carddisp.pl?gene=ABCG2 |  |
| BCAT2 | Branched Chain Amino Acid Transaminase 2 | Protein Coding | O15382 | 57 | GC19M048795 | 14.0811548233032 | https://www.genecards.org/cgi-bin/carddisp.pl?gene=BCAT2 |  |
| MOCOS | Molybdenum Cofactor Sulfurase | Protein Coding | Q96EN8 | 54 | GC18P036187 | 14.0486316680908 | https://www.genecards.org/cgi-bin/carddisp.pl?gene=MOCOS |  |
| NAGA | Alpha-N-Acetylgalactosaminidase | Protein Coding | P17050 | 58 | GC22M042058 | 14.0361394882202 | https://www.genecards.org/cgi-bin/carddisp.pl?gene=NAGA |  |
| SCARB2 | Scavenger Receptor Class B Member 2 | Protein Coding | Q14108 | 58 | GC04M076158 | 13.9917783737183 | https://www.genecards.org/cgi-bin/carddisp.pl?gene=SCARB2 |  |
| SPTSSA | Serine Palmitoyltransferase Small Subunit A | Protein Coding | Q969W0 | 46 | GC14M034432 | 13.9775724411011 | https://www.genecards.org/cgi-bin/carddisp.pl?gene=SPTSSA |  |
| ABCA2 | ATP Binding Cassette Subfamily A Member 2 | Protein Coding | Q9BZC7 | 55 | GC09M137007 | 13.9701452255249 | https://www.genecards.org/cgi-bin/carddisp.pl?gene=ABCA2 |  |
| SERPINE1 | Serpin Family E Member 1 | Protein Coding | P05121 | 63 | GC07P101127 | 13.9217958450317 | https://www.genecards.org/cgi-bin/carddisp.pl?gene=SERPINE1 |  |
| MPO | Myeloperoxidase | Protein Coding | P05164 | 65 | GC17M058269 | 13.9180402755737 | https://www.genecards.org/cgi-bin/carddisp.pl?gene=MPO |  |
| ABCB4 | ATP Binding Cassette Subfamily B Member 4 | Protein Coding | P21439 | 60 | GC07M087365 | 13.8672685623169 | https://www.genecards.org/cgi-bin/carddisp.pl?gene=ABCB4 |  |
| ALG1 | ALG1 Chitobiosyldiphosphodolichol Beta-Mannosyltransferase | Protein Coding | Q9BT22 | 58 | GC16P005033 | 13.8535461425781 | https://www.genecards.org/cgi-bin/carddisp.pl?gene=ALG1 |  |
| TPP1 | Tripeptidyl Peptidase 1 | Protein Coding | O14773 | 59 | GC11M015315 | 13.8534421920776 | https://www.genecards.org/cgi-bin/carddisp.pl?gene=TPP1 |  |
| AIFM1 | Apoptosis Inducing Factor Mitochondria Associated 1 | Protein Coding | O95831 | 63 | GC0XM130129 | 13.8522386550903 | https://www.genecards.org/cgi-bin/carddisp.pl?gene=AIFM1 |  |
| ABCC1 | ATP Binding Cassette Subfamily C Member 1 (ABCC1 Blood Group) | Protein Coding | P33527 | 61 | GC16P015949 | 13.8150777816772 | https://www.genecards.org/cgi-bin/carddisp.pl?gene=ABCC1 |  |
| DPP4 | Dipeptidyl Peptidase 4 | Protein Coding | P27487 | 62 | GC02M161992 | 13.7859954833984 | https://www.genecards.org/cgi-bin/carddisp.pl?gene=DPP4 |  |
| CHKB | Choline Kinase Beta | Protein Coding | Q9Y259 | 58 | GC22M050578 | 13.6695575714111 | https://www.genecards.org/cgi-bin/carddisp.pl?gene=CHKB |  |
| PRDX1 | Peroxiredoxin 1 | Protein Coding | Q06830 | 62 | GC01M045906 | 13.5754346847534 | https://www.genecards.org/cgi-bin/carddisp.pl?gene=PRDX1 |  |
| CEL | Carboxyl Ester Lipase | Protein Coding | P19835 | 58 | GC09P133061 | 13.5642585754395 | https://www.genecards.org/cgi-bin/carddisp.pl?gene=CEL |  |
| NSDHL | NAD(P) Dependent 3-Beta-Hydroxysteroid Dehydrogenase NSDHL | Protein Coding | Q15738 | 57 | GC0XP152830 | 13.441463470459 | https://www.genecards.org/cgi-bin/carddisp.pl?gene=NSDHL |  |
| APOA2 | Apolipoprotein A2 | Protein Coding | P02652 | 58 | GC01M161222 | 13.4309825897217 | https://www.genecards.org/cgi-bin/carddisp.pl?gene=APOA2 |  |
| PLA2G7 | Phospholipase A2 Group VII | Protein Coding | Q13093 | 63 | GC06M046704 | 13.4131383895874 | https://www.genecards.org/cgi-bin/carddisp.pl?gene=PLA2G7 |  |
| GOT1 | Glutamic-Oxaloacetic Transaminase 1 | Protein Coding | P17174 | 58 | GC10M099396 | 13.3890419006348 | https://www.genecards.org/cgi-bin/carddisp.pl?gene=GOT1 |  |
| PTPN11 | Protein Tyrosine Phosphatase Non-Receptor Type 11 | Protein Coding | Q06124 | 67 | GC12P112418 | 13.3722372055054 | https://www.genecards.org/cgi-bin/carddisp.pl?gene=PTPN11 |  |
| NPPA | Natriuretic Peptide A | Protein Coding | P01160 | 57 | GC01M022465 | 13.3592710494995 | https://www.genecards.org/cgi-bin/carddisp.pl?gene=NPPA |  |
| FUT2 | Fucosyltransferase 2 (H Blood Group) | Protein Coding | Q10981 | 58 | GC19P048695 | 13.3093156814575 | https://www.genecards.org/cgi-bin/carddisp.pl?gene=FUT2 |  |
| BDNF | Brain Derived Neurotrophic Factor | Protein Coding | P23560 | 62 | GC11M027654 | 13.2682647705078 | https://www.genecards.org/cgi-bin/carddisp.pl?gene=BDNF |  |
| ALAS2 | 5'-Aminolevulinate Synthase 2 | Protein Coding | P22557 | 59 | GC0XM055009 | 13.2656135559082 | https://www.genecards.org/cgi-bin/carddisp.pl?gene=ALAS2 |  |
| FADS2 | Fatty Acid Desaturase 2 | Protein Coding | O95864 | 54 | GC11P061792 | 13.2129917144775 | https://www.genecards.org/cgi-bin/carddisp.pl?gene=FADS2 |  |
| SPHK2 | Sphingosine Kinase 2 | Protein Coding | Q9NRA0 | 57 | GC19P048619 | 13.1955852508545 | https://www.genecards.org/cgi-bin/carddisp.pl?gene=SPHK2 |  |
| FGF21 | Fibroblast Growth Factor 21 | Protein Coding | Q9NSA1 | 52 | GC19P152833 | 13.1939668655396 | https://www.genecards.org/cgi-bin/carddisp.pl?gene=FGF21 |  |
| DHTKD1 | Dehydrogenase E1 And Transketolase Domain Containing 1 | Protein Coding | Q96HY7 | 53 | GC10P012068 | 13.1839532852173 | https://www.genecards.org/cgi-bin/carddisp.pl?gene=DHTKD1 |  |
| DNMT1 | DNA Methyltransferase 1 | Protein Coding | P26358 | 66 | GC19M010133 | 13.1645555496216 | https://www.genecards.org/cgi-bin/carddisp.pl?gene=DNMT1 |  |
| LRP1 | LDL Receptor Related Protein 1 | Protein Coding | Q07954 | 61 | GC12P057128 | 13.1452407836914 | https://www.genecards.org/cgi-bin/carddisp.pl?gene=LRP1 |  |
| NFS1 | NFS1 Cysteine Desulfurase | Protein Coding | Q9Y697 | 58 | GC20M035668 | 13.0159072875977 | https://www.genecards.org/cgi-bin/carddisp.pl?gene=NFS1 |  |
| PSAT1 | Phosphoserine Aminotransferase 1 | Protein Coding | Q9Y617 | 59 | GC09P078297 | 13.0121040344238 | https://www.genecards.org/cgi-bin/carddisp.pl?gene=PSAT1 |  |
| SLC25A3 | Solute Carrier Family 25 Member 3 | Protein Coding | Q00325 | 58 | GC12P098593 | 13.0055704116821 | https://www.genecards.org/cgi-bin/carddisp.pl?gene=SLC25A3 |  |
| LPIN2 | Lipin 2 | Protein Coding | Q92539 | 52 | GC18M004017 | 13.0039978027344 | https://www.genecards.org/cgi-bin/carddisp.pl?gene=LPIN2 |  |
| CYCS | Cytochrome C, Somatic | Protein Coding | P99999 | 61 | GC07M025118 | 12.9813108444214 | https://www.genecards.org/cgi-bin/carddisp.pl?gene=CYCS |  |
| SNCA | Synuclein Alpha | Protein Coding | P37840 | 65 | GC04M089724 | 12.9627294540405 | https://www.genecards.org/cgi-bin/carddisp.pl?gene=SNCA |  |
| KYNU | Kynureninase | Protein Coding | Q16719 | 61 | GC02P142877 | 12.9569120407104 | https://www.genecards.org/cgi-bin/carddisp.pl?gene=KYNU |  |
| CNR1 | Cannabinoid Receptor 1 | Protein Coding | P21554 | 59 | GC06M088139 | 12.9374551773071 | https://www.genecards.org/cgi-bin/carddisp.pl?gene=CNR1 |  |
| KRAS | KRAS Proto-Oncogene, GTPase | Protein Coding | P01116 | 66 | GC12M035956 | 12.9271936416626 | https://www.genecards.org/cgi-bin/carddisp.pl?gene=KRAS |  |
| CYB5R3 | Cytochrome B5 Reductase 3 | Protein Coding | P00387 | 57 | GC22M086164 | 12.9238243103027 | https://www.genecards.org/cgi-bin/carddisp.pl?gene=CYB5R3 |  |
| SEPSECS | Sep (O-Phosphoserine) TRNA:Sec (Selenocysteine) TRNA Synthase | Protein Coding | Q9HD40 | 56 | GC04M025121 | 12.9041376113892 | https://www.genecards.org/cgi-bin/carddisp.pl?gene=SEPSECS |  |
| TCF7L2 | Transcription Factor 7 Like 2 | Protein Coding | Q9NQB0 | 59 | GC10P112950 | 12.891921043396 | https://www.genecards.org/cgi-bin/carddisp.pl?gene=TCF7L2 |  |
| PRKN | Parkin RBR E3 Ubiquitin Protein Ligase | Protein Coding | O60260 | 62 | GC06M161348 | 12.8094682693481 | https://www.genecards.org/cgi-bin/carddisp.pl?gene=PRKN |  |
| CERT1 | Ceramide Transporter 1 | Protein Coding | Q9Y5P4 | 52 | GC05M075356 | 12.7917413711548 | https://www.genecards.org/cgi-bin/carddisp.pl?gene=CERT1 |  |
| CERS4 | Ceramide Synthase 4 | Protein Coding | Q9HA82 | 50 | GC19P008206 | 12.728006362915 | https://www.genecards.org/cgi-bin/carddisp.pl?gene=CERS4 |  |
| ELOVL4 | ELOVL Fatty Acid Elongase 4 | Protein Coding | Q9GZR5 | 58 | GC06M079914 | 12.6682939529419 | https://www.genecards.org/cgi-bin/carddisp.pl?gene=ELOVL4 |  |
| SIRT3 | Sirtuin 3 | Protein Coding | Q9NTG7 | 60 | GC11M000215 | 12.6665868759155 | https://www.genecards.org/cgi-bin/carddisp.pl?gene=SIRT3 |  |
| CERS6 | Ceramide Synthase 6 | Protein Coding | Q6ZMG9 | 51 | GC02P168491 | 12.6489162445068 | https://www.genecards.org/cgi-bin/carddisp.pl?gene=CERS6 |  |
| LCT | Lactase | Protein Coding | P09848 | 57 | GC02M135787 | 12.6488914489746 | https://www.genecards.org/cgi-bin/carddisp.pl?gene=LCT |  |
| CLN5 | CLN5 Intracellular Trafficking Protein | Protein Coding | O75503 | 53 | GC13P076990 | 12.647518157959 | https://www.genecards.org/cgi-bin/carddisp.pl?gene=CLN5 |  |
| GAD1 | Glutamate Decarboxylase 1 | Protein Coding | Q99259 | 62 | GC02P170813 | 12.6008920669556 | https://www.genecards.org/cgi-bin/carddisp.pl?gene=GAD1 |  |
| ST3GAL5 | ST3 Beta-Galactoside Alpha-2,3-Sialyltransferase 5 | Protein Coding | Q9UNP4 | 60 | GC02M085911 | 12.5915679931641 | https://www.genecards.org/cgi-bin/carddisp.pl?gene=ST3GAL5 |  |
| ATP13A2 | ATPase Cation Transporting 13A2 | Protein Coding | Q9NQ11 | 57 | GC01M016985 | 12.5911502838135 | https://www.genecards.org/cgi-bin/carddisp.pl?gene=ATP13A2 |  |
| SLC30A10 | Solute Carrier Family 30 Member 10 | Protein Coding | Q6XR72 | 54 | GC01M219685 | 12.5763931274414 | https://www.genecards.org/cgi-bin/carddisp.pl?gene=SLC30A10 |  |
| SMPD3 | Sphingomyelin Phosphodiesterase 3 | Protein Coding | Q9NY59 | 54 | GC16M068358 | 12.5721273422241 | https://www.genecards.org/cgi-bin/carddisp.pl?gene=SMPD3 |  |
| GCKR | Glucokinase Regulator | Protein Coding | Q14397 | 52 | GC02P027496 | 12.5702714920044 | https://www.genecards.org/cgi-bin/carddisp.pl?gene=GCKR |  |
| HTR1A | 5-Hydroxytryptamine Receptor 1A | Protein Coding | P08908 | 59 | GC05M063960 | 12.5571947097778 | https://www.genecards.org/cgi-bin/carddisp.pl?gene=HTR1A |  |
| MYC | MYC Proto-Oncogene, BHLH Transcription Factor | Protein Coding | P01106 | 65 | GC08P127735 | 12.5472097396851 | https://www.genecards.org/cgi-bin/carddisp.pl?gene=MYC |  |
| H19 | H19 Imprinted Maternally Expressed Transcript | RNA Gene |  | 37 | GC11M001995 | 12.4344215393066 | https://www.genecards.org/cgi-bin/carddisp.pl?gene=H19 |  |
| IGF1R | Insulin Like Growth Factor 1 Receptor | Protein Coding | P08069 | 68 | GC15P098648 | 12.4270515441895 | https://www.genecards.org/cgi-bin/carddisp.pl?gene=IGF1R |  |
| NFE2L2 | NFE2 Like BZIP Transcription Factor 2 | Protein Coding | Q16236 | 64 | GC02M177227 | 12.3889904022217 | https://www.genecards.org/cgi-bin/carddisp.pl?gene=NFE2L2 |  |
| ATP8B1 | ATPase Phospholipid Transporting 8B1 | Protein Coding | O43520 | 54 | GC18M057646 | 12.3777379989624 | https://www.genecards.org/cgi-bin/carddisp.pl?gene=ATP8B1 |  |
| ATP5F1E | ATP Synthase F1 Subunit Epsilon | Protein Coding | P56381 | 54 | GC20M059026 | 12.3531551361084 | https://www.genecards.org/cgi-bin/carddisp.pl?gene=ATP5F1E |  |
| SCARB1 | Scavenger Receptor Class B Member 1 | Protein Coding | Q8WTV0 | 60 | GC12M124776 | 12.3406744003296 | https://www.genecards.org/cgi-bin/carddisp.pl?gene=SCARB1 |  |
| SGMS1 | Sphingomyelin Synthase 1 | Protein Coding | Q86VZ5 | 50 | GC10M050305 | 12.3341436386108 | https://www.genecards.org/cgi-bin/carddisp.pl?gene=SGMS1 |  |
| SREBF2 | Sterol Regulatory Element Binding Transcription Factor 2 | Protein Coding | Q12772 | 55 | GC22P041833 | 12.3226890563965 | https://www.genecards.org/cgi-bin/carddisp.pl?gene=SREBF2 |  |
| POMGNT2 | Protein O-Linked Mannose N-Acetylglucosaminyltransferase 2 (Beta 1,4-) | Protein Coding | Q8NAT1 | 51 | GC03M043121 | 12.3049230575562 | https://www.genecards.org/cgi-bin/carddisp.pl?gene=POMGNT2 |  |
| PIK3CG | Phosphatidylinositol-4,5-Bisphosphate 3-Kinase Catalytic Subunit Gamma | Protein Coding | P48736 | 62 | GC07P106865 | 12.2965316772461 | https://www.genecards.org/cgi-bin/carddisp.pl?gene=PIK3CG |  |
| FADS3 | Fatty Acid Desaturase 3 | Protein Coding | Q9Y5Q0 | 48 | GC11M061873 | 12.2641448974609 | https://www.genecards.org/cgi-bin/carddisp.pl?gene=FADS3 |  |
| CERNA3 | Competing Endogenous LncRNA 3 For MiR-645 | RNA Gene |  | 21 | GC08P056444 | 12.2495050430298 | https://www.genecards.org/cgi-bin/carddisp.pl?gene=CERNA3 |  |
| PCYT1A | Phosphate Cytidylyltransferase 1A, Choline | Protein Coding | P49585 | 59 | GC03M196214 | 12.2485609054565 | https://www.genecards.org/cgi-bin/carddisp.pl?gene=PCYT1A |  |
| IFNG | Interferon Gamma | Protein Coding | P01579 | 63 | GC12M068154 | 12.2278823852539 | https://www.genecards.org/cgi-bin/carddisp.pl?gene=IFNG |  |
| MLXIPL | MLX Interacting Protein Like | Protein Coding | Q9NP71 | 55 | GC07M073593 | 12.2068290710449 | https://www.genecards.org/cgi-bin/carddisp.pl?gene=MLXIPL |  |
| CAVIN1 | Caveolae Associated Protein 1 | Protein Coding | Q6NZI2 | 54 | GC17M097212 | 12.182599067688 | https://www.genecards.org/cgi-bin/carddisp.pl?gene=CAVIN1 |  |
| CTNNB1 | Catenin Beta 1 | Protein Coding | P35222 | 67 | GC03P041194 | 12.1721849441528 | https://www.genecards.org/cgi-bin/carddisp.pl?gene=CTNNB1 |  |
| DNM2 | Dynamin 2 | Protein Coding | P50570 | 63 | GC19P010718 | 12.1617336273193 | https://www.genecards.org/cgi-bin/carddisp.pl?gene=DNM2 |  |
| FABP4 | Fatty Acid Binding Protein 4 | Protein Coding | P15090 | 56 | GC08M081478 | 12.1031684875488 | https://www.genecards.org/cgi-bin/carddisp.pl?gene=FABP4 |  |
| RAF1 | Raf-1 Proto-Oncogene, Serine/Threonine Kinase | Protein Coding | P04049 | 67 | GC03M012583 | 12.0494785308838 | https://www.genecards.org/cgi-bin/carddisp.pl?gene=RAF1 |  |
| SLC33A1 | Solute Carrier Family 33 Member 1 | Protein Coding | O00400 | 55 | GC03M155821 | 12.0247917175293 | https://www.genecards.org/cgi-bin/carddisp.pl?gene=SLC33A1 |  |
| PINK1 | PTEN Induced Kinase 1 | Protein Coding | Q9BXM7 | 60 | GC01P076060 | 11.9728574752808 | https://www.genecards.org/cgi-bin/carddisp.pl?gene=PINK1 |  |
| NPC1L1 | NPC1 Like Intracellular Cholesterol Transporter 1 | Protein Coding | Q9UHC9 | 56 | GC07M044512 | 11.9395017623901 | https://www.genecards.org/cgi-bin/carddisp.pl?gene=NPC1L1 |  |
| ALG14 | ALG14 UDP-N-Acetylglucosaminyltransferase Subunit | Protein Coding | Q96F25 | 52 | GC01M094974 | 11.9256820678711 | https://www.genecards.org/cgi-bin/carddisp.pl?gene=ALG14 |  |
| B4GALT1 | Beta-1,4-Galactosyltransferase 1 | Protein Coding | P15291 | 59 | GC09M033100 | 11.9202785491943 | https://www.genecards.org/cgi-bin/carddisp.pl?gene=B4GALT1 |  |
| DGKE | Diacylglycerol Kinase Epsilon | Protein Coding | P52429 | 58 | GC17P056834 | 11.9148502349854 | https://www.genecards.org/cgi-bin/carddisp.pl?gene=DGKE |  |
| CCL2 | C-C Motif Chemokine Ligand 2 | Protein Coding | P13500 | 62 | GC17P034255 | 11.894341468811 | https://www.genecards.org/cgi-bin/carddisp.pl?gene=CCL2 |  |
| TFRC | Transferrin Receptor | Protein Coding | P02786 | 64 | GC03M196703 | 11.8803005218506 | https://www.genecards.org/cgi-bin/carddisp.pl?gene=TFRC |  |
| SAMHD1 | SAM And HD Domain Containing Deoxynucleoside Triphosphate Triphosphohydrolase 1 | Protein Coding | Q9Y3Z3 | 55 | GC20M036890 | 11.8671789169312 | https://www.genecards.org/cgi-bin/carddisp.pl?gene=SAMHD1 |  |
| CTSK | Cathepsin K | Protein Coding | P43235 | 62 | GC01M167274 | 11.8639707565308 | https://www.genecards.org/cgi-bin/carddisp.pl?gene=CTSK |  |
| CERK | Ceramide Kinase | Protein Coding | Q8TCT0 | 52 | GC22M046684 | 11.7969131469727 | https://www.genecards.org/cgi-bin/carddisp.pl?gene=CERK |  |
| ST3GAL3 | ST3 Beta-Galactoside Alpha-2,3-Sialyltransferase 3 | Protein Coding | Q11203 | 58 | GC01P043705 | 11.792049407959 | https://www.genecards.org/cgi-bin/carddisp.pl?gene=ST3GAL3 |  |
| GNAS | GNAS Complex Locus | Protein Coding | P84996 | 63 | GC20P058839 | 11.7674236297607 | https://www.genecards.org/cgi-bin/carddisp.pl?gene=GNAS |  |
| SPNS2 | SPNS Lysolipid Transporter 2, Sphingosine-1-Phosphate | Protein Coding | Q8IVW8 | 44 | GC17P004498 | 11.7640762329102 | https://www.genecards.org/cgi-bin/carddisp.pl?gene=SPNS2 |  |
| APOA4 | Apolipoprotein A4 | Protein Coding | P06727 | 55 | GC11M116820 | 11.7320404052734 | https://www.genecards.org/cgi-bin/carddisp.pl?gene=APOA4 |  |
| S1PR1 | Sphingosine-1-Phosphate Receptor 1 | Protein Coding | P21453 | 59 | GC01P101236 | 11.7293939590454 | https://www.genecards.org/cgi-bin/carddisp.pl?gene=S1PR1 |  |
| PLA2G4A | Phospholipase A2 Group IVA | Protein Coding | P47712 | 62 | GC01P186798 | 11.7237272262573 | https://www.genecards.org/cgi-bin/carddisp.pl?gene=PLA2G4A |  |
| BCL2 | BCL2 Apoptosis Regulator | Protein Coding | P10415 | 64 | GC18M063123 | 11.718225479126 | https://www.genecards.org/cgi-bin/carddisp.pl?gene=BCL2 |  |
| ARV1 | ARV1 Homolog, Fatty Acid Homeostasis Modulator | Protein Coding | Q9H2C2 | 47 | GC01P230978 | 11.7113952636719 | https://www.genecards.org/cgi-bin/carddisp.pl?gene=ARV1 |  |
| ACER2 | Alkaline Ceramidase 2 | Protein Coding | Q5QJU3 | 46 | GC09P019409 | 11.7013483047485 | https://www.genecards.org/cgi-bin/carddisp.pl?gene=ACER2 |  |
| ELOVL1 | ELOVL Fatty Acid Elongase 1 | Protein Coding | Q9BW60 | 52 | GC01M043363 | 11.6690826416016 | https://www.genecards.org/cgi-bin/carddisp.pl?gene=ELOVL1 |  |
| STAT3 | Signal Transducer And Activator Of Transcription 3 | Protein Coding | P40763 | 67 | GC17M042313 | 11.6434669494629 | https://www.genecards.org/cgi-bin/carddisp.pl?gene=STAT3 |  |
| MBOAT7 | Membrane Bound Acylglycerophosphatidylinositol O-Acyltransferase MBOAT7 | Protein Coding | Q96N66 | 51 | GC19M054173 | 11.6198253631592 | https://www.genecards.org/cgi-bin/carddisp.pl?gene=MBOAT7 |  |
| HCCS | Holocytochrome C Synthase | Protein Coding | P53701 | 55 | GC0XP011111 | 11.6066093444824 | https://www.genecards.org/cgi-bin/carddisp.pl?gene=HCCS |  |
| MIR126 | MicroRNA 126 | RNA Gene |  | 31 | GC09P136670 | 11.5941724777222 | https://www.genecards.org/cgi-bin/carddisp.pl?gene=MIR126 |  |
| SHMT1 | Serine Hydroxymethyltransferase 1 | Protein Coding | P34896 | 57 | GC17M096499 | 11.5788831710815 | https://www.genecards.org/cgi-bin/carddisp.pl?gene=SHMT1 |  |
| B4GALT6 | Beta-1,4-Galactosyltransferase 6 | Protein Coding | Q9UBX8 | 50 | GC18M031622 | 11.5750331878662 | https://www.genecards.org/cgi-bin/carddisp.pl?gene=B4GALT6 |  |
| ATM | ATM Serine/Threonine Kinase | Protein Coding | Q13315 | 66 | GC11P108222 | 11.5534696578979 | https://www.genecards.org/cgi-bin/carddisp.pl?gene=ATM |  |
| GFAP | Glial Fibrillary Acidic Protein | Protein Coding | P14136 | 61 | GC17M097317 | 11.5410032272339 | https://www.genecards.org/cgi-bin/carddisp.pl?gene=GFAP |  |
| MT-TK | Mitochondrially Encoded TRNA-Lys (AAA/G) | RNA Gene |  | 22 | GCMTP008297 | 11.5228204727173 | https://www.genecards.org/cgi-bin/carddisp.pl?gene=MT-TK |  |
| CERS5 | Ceramide Synthase 5 | Protein Coding | Q8N5B7 | 48 | GC12M050129 | 11.4826469421387 | https://www.genecards.org/cgi-bin/carddisp.pl?gene=CERS5 |  |
| SLC27A4 | Solute Carrier Family 27 Member 4 | Protein Coding | Q6P1M0 | 56 | GC09P128340 | 11.3171625137329 | https://www.genecards.org/cgi-bin/carddisp.pl?gene=SLC27A4 |  |
| DNAH8 | Dynein Axonemal Heavy Chain 8 | Protein Coding | Q96JB1 | 50 | GC06P182037 | 11.314510345459 | https://www.genecards.org/cgi-bin/carddisp.pl?gene=DNAH8 |  |
| TNFSF11 | TNF Superfamily Member 11 | Protein Coding | O14788 | 62 | GC13P042562 | 11.2762746810913 | https://www.genecards.org/cgi-bin/carddisp.pl?gene=TNFSF11 |  |
| TLR4 | Toll Like Receptor 4 | Protein Coding | O00206 | 64 | GC09P117704 | 11.261043548584 | https://www.genecards.org/cgi-bin/carddisp.pl?gene=TLR4 |  |
| ARSG | Arylsulfatase G | Protein Coding | Q96EG1 | 54 | GC17P068259 | 11.2544565200806 | https://www.genecards.org/cgi-bin/carddisp.pl?gene=ARSG |  |
| PIKFYVE | Phosphoinositide Kinase, FYVE-Type Zinc Finger Containing | Protein Coding | Q9Y2I7 | 63 | GC02P208266 | 11.2518835067749 | https://www.genecards.org/cgi-bin/carddisp.pl?gene=PIKFYVE |  |
| CDH23 | Cadherin Related 23 | Protein Coding | Q9H251 | 57 | GC10P071396 | 11.2504844665527 | https://www.genecards.org/cgi-bin/carddisp.pl?gene=CDH23 |  |
| FAS | Fas Cell Surface Death Receptor | Protein Coding | P25445 | 64 | GC10P124091 | 11.1310520172119 | https://www.genecards.org/cgi-bin/carddisp.pl?gene=FAS |  |
| CHKA | Choline Kinase Alpha | Protein Coding | P35790 | 57 | GC11M068052 | 11.0806474685669 | https://www.genecards.org/cgi-bin/carddisp.pl?gene=CHKA |  |
| TRPM6 | Transient Receptor Potential Cation Channel Subfamily M Member 6 | Protein Coding | Q9BX84 | 57 | GC09M074725 | 11.0392818450928 | https://www.genecards.org/cgi-bin/carddisp.pl?gene=TRPM6 |  |
| MAPK3 | Mitogen-Activated Protein Kinase 3 | Protein Coding | P27361 | 62 | GC16M051076 | 10.9573097229004 | https://www.genecards.org/cgi-bin/carddisp.pl?gene=MAPK3 |  |
| PLCB1 | Phospholipase C Beta 1 | Protein Coding | Q9NQ66 | 61 | GC20P008077 | 10.9153642654419 | https://www.genecards.org/cgi-bin/carddisp.pl?gene=PLCB1 |  |
| CERKL | CERK Like Autophagy Regulator | Protein Coding | Q49MI3 | 51 | GC02M181536 | 10.9076366424561 | https://www.genecards.org/cgi-bin/carddisp.pl?gene=CERKL |  |
| ODC1 | Ornithine Decarboxylase 1 | Protein Coding | P11926 | 61 | GC02M010432 | 10.8686800003052 | https://www.genecards.org/cgi-bin/carddisp.pl?gene=ODC1 |  |
| EIF2AK3 | Eukaryotic Translation Initiation Factor 2 Alpha Kinase 3 | Protein Coding | Q9NZJ5 | 63 | GC02M088556 | 10.8442153930664 | https://www.genecards.org/cgi-bin/carddisp.pl?gene=EIF2AK3 |  |
| ADRB2 | Adrenoceptor Beta 2 | Protein Coding | P07550 | 62 | GC05P159202 | 10.8344058990479 | https://www.genecards.org/cgi-bin/carddisp.pl?gene=ADRB2 |  |
| MIR33B | MicroRNA 33b | RNA Gene |  | 27 | GC17M017813 | 10.8323659896851 | https://www.genecards.org/cgi-bin/carddisp.pl?gene=MIR33B |  |
| LINC02605 | Long Intergenic Non-Protein Coding RNA 2605 | RNA Gene |  | 21 | GC08P078838 | 10.8162260055542 | https://www.genecards.org/cgi-bin/carddisp.pl?gene=LINC02605 |  |
| IDH1 | Isocitrate Dehydrogenase (NADP(+)) 1 | Protein Coding | O75874 | 65 | GC02M208236 | 10.8078575134277 | https://www.genecards.org/cgi-bin/carddisp.pl?gene=IDH1 |  |
| NR5A1 | Nuclear Receptor Subfamily 5 Group A Member 1 | Protein Coding | Q13285 | 62 | GC09M124481 | 10.794135093689 | https://www.genecards.org/cgi-bin/carddisp.pl?gene=NR5A1 |  |
| PRL | Prolactin | Protein Coding | P01236 | 56 | GC06M022287 | 10.776294708252 | https://www.genecards.org/cgi-bin/carddisp.pl?gene=PRL |  |
| MIR17 | MicroRNA 17 | RNA Gene |  | 28 | GC13P091350 | 10.7029190063477 | https://www.genecards.org/cgi-bin/carddisp.pl?gene=MIR17 |  |
| FIG4 | FIG4 Phosphoinositide 5-Phosphatase | Protein Coding | Q92562 | 56 | GC06P182745 | 10.6876993179321 | https://www.genecards.org/cgi-bin/carddisp.pl?gene=FIG4 |  |
| IGFBP3 | Insulin Like Growth Factor Binding Protein 3 | Protein Coding | P17936 | 59 | GC07M045912 | 10.6308364868164 | https://www.genecards.org/cgi-bin/carddisp.pl?gene=IGFBP3 |  |
| ADIPOR1 | Adiponectin Receptor 1 | Protein Coding | Q96A54 | 55 | GC01M202940 | 10.6158771514893 | https://www.genecards.org/cgi-bin/carddisp.pl?gene=ADIPOR1 |  |
| FOXO1 | Forkhead Box O1 | Protein Coding | Q12778 | 64 | GC13M040555 | 10.6138792037964 | https://www.genecards.org/cgi-bin/carddisp.pl?gene=FOXO1 |  |
| COL1A1 | Collagen Type I Alpha 1 Chain | Protein Coding | P02452 | 62 | GC17M097561 | 10.6088123321533 | https://www.genecards.org/cgi-bin/carddisp.pl?gene=COL1A1 |  |
| SHMT2 | Serine Hydroxymethyltransferase 2 | Protein Coding | P34897 | 58 | GC12P057229 | 10.6001710891724 | https://www.genecards.org/cgi-bin/carddisp.pl?gene=SHMT2 |  |
| CASP3 | Caspase 3 | Protein Coding | P42574 | 62 | GC04M184627 | 10.5900764465332 | https://www.genecards.org/cgi-bin/carddisp.pl?gene=CASP3 |  |
| CHIT1 | Chitinase 1 | Protein Coding | Q13231 | 57 | GC01M203213 | 10.5731582641602 | https://www.genecards.org/cgi-bin/carddisp.pl?gene=CHIT1 |  |
| TSHR | Thyroid Stimulating Hormone Receptor | Protein Coding | P16473 | 61 | GC14P080954 | 10.5427436828613 | https://www.genecards.org/cgi-bin/carddisp.pl?gene=TSHR |  |
| MAPT | Microtubule Associated Protein Tau | Protein Coding | P10636 | 62 | GC17P045894 | 10.5151386260986 | https://www.genecards.org/cgi-bin/carddisp.pl?gene=MAPT |  |
| ALPP | Alkaline Phosphatase, Placental | Protein Coding | P05187 | 57 | GC02P232378 | 10.4962005615234 | https://www.genecards.org/cgi-bin/carddisp.pl?gene=ALPP |  |
| MAP2K1 | Mitogen-Activated Protein Kinase Kinase 1 | Protein Coding | Q02750 | 67 | GC15P066386 | 10.4642915725708 | https://www.genecards.org/cgi-bin/carddisp.pl?gene=MAP2K1 |  |
| ARSL | Arylsulfatase L | Protein Coding | P51690 | 52 | GC0XM002934 | 10.4485120773315 | https://www.genecards.org/cgi-bin/carddisp.pl?gene=ARSL |  |
| FBN1 | Fibrillin 1 | Protein Coding | P35555 | 60 | GC15M048408 | 10.4243898391724 | https://www.genecards.org/cgi-bin/carddisp.pl?gene=FBN1 |  |
| SLC35A1 | Solute Carrier Family 35 Member A1 | Protein Coding | P78382 | 52 | GC06P087470 | 10.3998556137085 | https://www.genecards.org/cgi-bin/carddisp.pl?gene=SLC35A1 |  |
| SOD2 | Superoxide Dismutase 2 | Protein Coding | P04179 | 61 | GC06M159669 | 10.3852300643921 | https://www.genecards.org/cgi-bin/carddisp.pl?gene=SOD2 |  |
| MAPK8 | Mitogen-Activated Protein Kinase 8 | Protein Coding | P45983 | 62 | GC10P048306 | 10.3567361831665 | https://www.genecards.org/cgi-bin/carddisp.pl?gene=MAPK8 |  |
| AKR1C3 | Aldo-Keto Reductase Family 1 Member C3 | Protein Coding | P42330 | 57 | GC10P005035 | 10.3395490646362 | https://www.genecards.org/cgi-bin/carddisp.pl?gene=AKR1C3 |  |
| SGPP2 | Sphingosine-1-Phosphate Phosphatase 2 | Protein Coding | Q8IWX5 | 42 | GC02P222424 | 10.337366104126 | https://www.genecards.org/cgi-bin/carddisp.pl?gene=SGPP2 |  |
| KCNH2 | Potassium Voltage-Gated Channel Subfamily H Member 2 | Protein Coding | Q12809 | 63 | GC07M150944 | 10.3220252990723 | https://www.genecards.org/cgi-bin/carddisp.pl?gene=KCNH2 |  |
| PLA2G2A | Phospholipase A2 Group IIA | Protein Coding | P14555 | 60 | GC01M019975 | 10.3191013336182 | https://www.genecards.org/cgi-bin/carddisp.pl?gene=PLA2G2A |  |
| ALOXE3 | Arachidonate Epidermal Lipoxygenase 3 | Protein Coding | Q9BYJ1 | 53 | GC17M096228 | 10.2975397109985 | https://www.genecards.org/cgi-bin/carddisp.pl?gene=ALOXE3 |  |
| KCNQ1 | Potassium Voltage-Gated Channel Subfamily Q Member 1 | Protein Coding | P51787 | 62 | GC11P002444 | 10.2230548858643 | https://www.genecards.org/cgi-bin/carddisp.pl?gene=KCNQ1 |  |
| PLCB4 | Phospholipase C Beta 4 | Protein Coding | Q15147 | 58 | GC20P009067 | 10.2203636169434 | https://www.genecards.org/cgi-bin/carddisp.pl?gene=PLCB4 |  |
| S1PR2 | Sphingosine-1-Phosphate Receptor 2 | Protein Coding | O95136 | 58 | GC19M108105 | 10.208610534668 | https://www.genecards.org/cgi-bin/carddisp.pl?gene=S1PR2 |  |
| AGXT2 | Alanine--Glyoxylate Aminotransferase 2 | Protein Coding | Q9BYV1 | 52 | GC05M034998 | 10.1977968215942 | https://www.genecards.org/cgi-bin/carddisp.pl?gene=AGXT2 |  |
| PLTP | Phospholipid Transfer Protein | Protein Coding | P55058 | 57 | GC20M045898 | 10.167423248291 | https://www.genecards.org/cgi-bin/carddisp.pl?gene=PLTP |  |
| CHAT | Choline O-Acetyltransferase | Protein Coding | P28329 | 61 | GC10P049609 | 10.1623487472534 | https://www.genecards.org/cgi-bin/carddisp.pl?gene=CHAT |  |
| EP300 | E1A Binding Protein P300 | Protein Coding | Q09472 | 65 | GC22P092249 | 10.0983180999756 | https://www.genecards.org/cgi-bin/carddisp.pl?gene=EP300 |  |
| VPS33B | VPS33B Late Endosome And Lysosome Associated | Protein Coding | Q9H267 | 54 | GC15M090998 | 10.0779733657837 | https://www.genecards.org/cgi-bin/carddisp.pl?gene=VPS33B |  |
| PLPP1 | Phospholipid Phosphatase 1 | Protein Coding | O14494 | 49 | GC05M055425 | 9.9929370880127 | https://www.genecards.org/cgi-bin/carddisp.pl?gene=PLPP1 |  |
| ACER1 | Alkaline Ceramidase 1 | Protein Coding | Q8TDN7 | 44 | GC19M006306 | 9.97197151184082 | https://www.genecards.org/cgi-bin/carddisp.pl?gene=ACER1 |  |
| ATP6V1E1 | ATPase H+ Transporting V1 Subunit E1 | Protein Coding | P36543 | 58 | GC22M017592 | 9.95415592193604 | https://www.genecards.org/cgi-bin/carddisp.pl?gene=ATP6V1E1 |  |
| ALDH3B1 | Aldehyde Dehydrogenase 3 Family Member B1 | Protein Coding | P43353 | 52 | GC11P108019 | 9.93862915039063 | https://www.genecards.org/cgi-bin/carddisp.pl?gene=ALDH3B1 |  |
| ELOVL6 | ELOVL Fatty Acid Elongase 6 | Protein Coding | Q9H5J4 | 52 | GC04M110045 | 9.93360805511475 | https://www.genecards.org/cgi-bin/carddisp.pl?gene=ELOVL6 |  |
| PARP1 | Poly(ADP-Ribose) Polymerase 1 | Protein Coding | P09874 | 64 | GC01M226360 | 9.93285846710205 | https://www.genecards.org/cgi-bin/carddisp.pl?gene=PARP1 |  |
| GAL3ST1 | Galactose-3-O-Sulfotransferase 1 | Protein Coding | Q99999 | 51 | GC22M030554 | 9.92560005187988 | https://www.genecards.org/cgi-bin/carddisp.pl?gene=GAL3ST1 |  |
| GSK3B | Glycogen Synthase Kinase 3 Beta | Protein Coding | P49841 | 64 | GC03M119821 | 9.92119693756104 | https://www.genecards.org/cgi-bin/carddisp.pl?gene=GSK3B |  |
| SRC | SRC Proto-Oncogene, Non-Receptor Tyrosine Kinase | Protein Coding | P12931 | 64 | GC20P037344 | 9.90342330932617 | https://www.genecards.org/cgi-bin/carddisp.pl?gene=SRC |  |
| PIK3C2A | Phosphatidylinositol-4-Phosphate 3-Kinase Catalytic Subunit Type 2 Alpha | Protein Coding | O00443 | 60 | GC11M018151 | 9.89215087890625 | https://www.genecards.org/cgi-bin/carddisp.pl?gene=PIK3C2A |  |
| PLPP3 | Phospholipid Phosphatase 3 | Protein Coding | O14495 | 51 | GC01M056495 | 9.89151382446289 | https://www.genecards.org/cgi-bin/carddisp.pl?gene=PLPP3 |  |
| RARS1 | Arginyl-TRNA Synthetase 1 | Protein Coding | P54136 | 57 | GC05P168487 | 9.82716941833496 | https://www.genecards.org/cgi-bin/carddisp.pl?gene=RARS1 |  |
| PLP1 | Proteolipid Protein 1 | Protein Coding | P60201 | 56 | GC0XP103773 | 9.82619953155518 | https://www.genecards.org/cgi-bin/carddisp.pl?gene=PLP1 |  |
| PTPN1 | Protein Tyrosine Phosphatase Non-Receptor Type 1 | Protein Coding | P18031 | 62 | GC20P050510 | 9.7638111114502 | https://www.genecards.org/cgi-bin/carddisp.pl?gene=PTPN1 |  |
| ARSH | Arylsulfatase Family Member H | Protein Coding | Q5FYA8 | 44 | GC0XP003006 | 9.76364994049072 | https://www.genecards.org/cgi-bin/carddisp.pl?gene=ARSH |  |
| NOS2 | Nitric Oxide Synthase 2 | Protein Coding | P35228 | 62 | GC17M027756 | 9.74985504150391 | https://www.genecards.org/cgi-bin/carddisp.pl?gene=NOS2 |  |
| CD40LG | CD40 Ligand | Protein Coding | P29965 | 62 | GC0XP136649 | 9.74207019805908 | https://www.genecards.org/cgi-bin/carddisp.pl?gene=CD40LG |  |
| ABCG1 | ATP Binding Cassette Subfamily G Member 1 | Protein Coding | P45844 | 55 | GC21P042199 | 9.71543312072754 | https://www.genecards.org/cgi-bin/carddisp.pl?gene=ABCG1 |  |
| HSPG2 | Heparan Sulfate Proteoglycan 2 | Protein Coding | P98160 | 61 | GC01M021822 | 9.71184730529785 | https://www.genecards.org/cgi-bin/carddisp.pl?gene=HSPG2 |  |
| KCNJ5 | Potassium Inwardly Rectifying Channel Subfamily J Member 5 | Protein Coding | P48544 | 61 | GC11P128891 | 9.68618583679199 | https://www.genecards.org/cgi-bin/carddisp.pl?gene=KCNJ5 |  |
| SMAD3 | SMAD Family Member 3 | Protein Coding | P84022 | 66 | GC15P067063 | 9.67448806762695 | https://www.genecards.org/cgi-bin/carddisp.pl?gene=SMAD3 |  |
| PSEN1 | Presenilin 1 | Protein Coding | P49768 | 66 | GC14P073136 | 9.66738319396973 | https://www.genecards.org/cgi-bin/carddisp.pl?gene=PSEN1 |  |
| CXCL8 | C-X-C Motif Chemokine Ligand 8 | Protein Coding | P10145 | 57 | GC04P073740 | 9.66447830200195 | https://www.genecards.org/cgi-bin/carddisp.pl?gene=CXCL8 |  |
| SRD5A1 | Steroid 5 Alpha-Reductase 1 | Protein Coding | P18405 | 56 | GC05P006633 | 9.59130573272705 | https://www.genecards.org/cgi-bin/carddisp.pl?gene=SRD5A1 |  |
| FTH1 | Ferritin Heavy Chain 1 | Protein Coding | P02794 | 63 | GC11M061959 | 9.56385040283203 | https://www.genecards.org/cgi-bin/carddisp.pl?gene=FTH1 |  |
| SGMS2 | Sphingomyelin Synthase 2 | Protein Coding | Q8NHU3 | 48 | GC04P107824 | 9.54227447509766 | https://www.genecards.org/cgi-bin/carddisp.pl?gene=SGMS2 |  |
| ELOVL3 | ELOVL Fatty Acid Elongase 3 | Protein Coding | Q9HB03 | 46 | GC10P124469 | 9.52657508850098 | https://www.genecards.org/cgi-bin/carddisp.pl?gene=ELOVL3 |  |
| MAPK14 | Mitogen-Activated Protein Kinase 14 | Protein Coding | Q16539 | 64 | GC06P182012 | 9.50404071807861 | https://www.genecards.org/cgi-bin/carddisp.pl?gene=MAPK14 |  |
| PEMT | Phosphatidylethanolamine N-Methyltransferase | Protein Coding | Q9UBM1 | 51 | GC17M096443 | 9.49709129333496 | https://www.genecards.org/cgi-bin/carddisp.pl?gene=PEMT |  |
| EGFR | Epidermal Growth Factor Receptor | Protein Coding | P00533 | 68 | GC07P055019 | 9.4857006072998 | https://www.genecards.org/cgi-bin/carddisp.pl?gene=EGFR |  |
| IL18 | Interleukin 18 | Protein Coding | Q14116 | 57 | GC11M112143 | 9.48509407043457 | https://www.genecards.org/cgi-bin/carddisp.pl?gene=IL18 |  |
| GPAM | Glycerol-3-Phosphate Acyltransferase, Mitochondrial | Protein Coding | Q9HCL2 | 53 | GC10M112148 | 9.48250102996826 | https://www.genecards.org/cgi-bin/carddisp.pl?gene=GPAM |  |
| B4GALNT1 | Beta-1,4-N-Acetyl-Galactosaminyltransferase 1 | Protein Coding | Q00973 | 58 | GC12M057623 | 9.47717952728271 | https://www.genecards.org/cgi-bin/carddisp.pl?gene=B4GALNT1 |  |
| HTT | Huntingtin | Protein Coding | P42858 | 57 | GC04P003041 | 9.45859146118164 | https://www.genecards.org/cgi-bin/carddisp.pl?gene=HTT |  |
| STT3A | STT3 Oligosaccharyltransferase Complex Catalytic Subunit A | Protein Coding | P46977 | 58 | GC11P126001 | 9.45835876464844 | https://www.genecards.org/cgi-bin/carddisp.pl?gene=STT3A |  |
| CAV3 | Caveolin 3 | Protein Coding | P56539 | 57 | GC03P008733 | 9.44949340820313 | https://www.genecards.org/cgi-bin/carddisp.pl?gene=CAV3 |  |
| HSP90AA1 | Heat Shock Protein 90 Alpha Family Class A Member 1 | Protein Coding | P07900 | 64 | GC14M102080 | 9.35416126251221 | https://www.genecards.org/cgi-bin/carddisp.pl?gene=HSP90AA1 |  |
| B3GALNT1 | Beta-1,3-N-Acetylgalactosaminyltransferase 1 (Globoside Blood Group) | Protein Coding | O75752 | 50 | GC03M161083 | 9.35385131835938 | https://www.genecards.org/cgi-bin/carddisp.pl?gene=B3GALNT1 |  |
| TREM2 | Triggering Receptor Expressed On Myeloid Cells 2 | Protein Coding | Q9NZC2 | 57 | GC06M106680 | 9.35221385955811 | https://www.genecards.org/cgi-bin/carddisp.pl?gene=TREM2 |  |
| LPA | Lipoprotein(A) | Protein Coding | P08519 | 55 | GC06M160531 | 9.35166454315186 | https://www.genecards.org/cgi-bin/carddisp.pl?gene=LPA |  |
| ELOVL2 | ELOVL Fatty Acid Elongase 2 | Protein Coding | Q9NXB9 | 50 | GC06M010980 | 9.34968948364258 | https://www.genecards.org/cgi-bin/carddisp.pl?gene=ELOVL2 |  |
| NLRP3 | NLR Family Pyrin Domain Containing 3 | Protein Coding | Q96P20 | 62 | GC01P247632 | 9.32767581939697 | https://www.genecards.org/cgi-bin/carddisp.pl?gene=NLRP3 |  |
| VDAC1 | Voltage Dependent Anion Channel 1 | Protein Coding | P21796 | 59 | GC05M133975 | 9.31198978424072 | https://www.genecards.org/cgi-bin/carddisp.pl?gene=VDAC1 |  |
| GRN | Granulin Precursor | Protein Coding | P28799 | 61 | GC17P044345 | 9.2985668182373 | https://www.genecards.org/cgi-bin/carddisp.pl?gene=GRN |  |
| SCARNA5 | Small Cajal Body-Specific RNA 5 | RNA Gene |  | 28 | GC02P233275 | 9.28636360168457 | https://www.genecards.org/cgi-bin/carddisp.pl?gene=SCARNA5 |  |
| GATA6 | GATA Binding Protein 6 | Protein Coding | Q92908 | 60 | GC18P022169 | 9.2637825012207 | https://www.genecards.org/cgi-bin/carddisp.pl?gene=GATA6 |  |
| VPS33A | VPS33A Core Subunit Of CORVET And HOPS Complexes | Protein Coding | Q96AX1 | 52 | GC12M122229 | 9.26324939727783 | https://www.genecards.org/cgi-bin/carddisp.pl?gene=VPS33A |  |
| HLA-B | Major Histocompatibility Complex, Class I, B | Protein Coding | P01889 | 59 | GC06M106436 | 9.25704193115234 | https://www.genecards.org/cgi-bin/carddisp.pl?gene=HLA-B |  |
| PTPRC | Protein Tyrosine Phosphatase Receptor Type C | Protein Coding | P08575 | 65 | GC01P198607 | 9.24702167510986 | https://www.genecards.org/cgi-bin/carddisp.pl?gene=PTPRC |  |
| PDXK | Pyridoxal Kinase | Protein Coding | O00764 | 59 | GC21P043719 | 9.2298059463501 | https://www.genecards.org/cgi-bin/carddisp.pl?gene=PDXK |  |
| ESR2 | Estrogen Receptor 2 | Protein Coding | Q92731 | 61 | GC14M064084 | 9.22674083709717 | https://www.genecards.org/cgi-bin/carddisp.pl?gene=ESR2 |  |
| ALDH3A1 | Aldehyde Dehydrogenase 3 Family Member A1 | Protein Coding | P30838 | 58 | GC17M019737 | 9.20467662811279 | https://www.genecards.org/cgi-bin/carddisp.pl?gene=ALDH3A1 |  |
| KYAT1 | Kynurenine Aminotransferase 1 | Protein Coding | Q16773 | 51 | GC09M131882 | 9.19111347198486 | https://www.genecards.org/cgi-bin/carddisp.pl?gene=KYAT1 |  |
| ABCC6 | ATP Binding Cassette Subfamily C Member 6 | Protein Coding | O95255 | 60 | GC16M050398 | 9.18122386932373 | https://www.genecards.org/cgi-bin/carddisp.pl?gene=ABCC6 |  |
| MIRLET7C | MicroRNA Let-7c | RNA Gene |  | 31 | GC21P021313 | 9.11688995361328 | https://www.genecards.org/cgi-bin/carddisp.pl?gene=MIRLET7C |  |
| IDO1 | Indoleamine 2,3-Dioxygenase 1 | Protein Coding | P14902 | 59 | GC08P044830 | 9.08991527557373 | https://www.genecards.org/cgi-bin/carddisp.pl?gene=IDO1 |  |
| SCAP | SREBF Chaperone | Protein Coding | Q12770 | 53 | GC03M047413 | 9.06334972381592 | https://www.genecards.org/cgi-bin/carddisp.pl?gene=SCAP |  |
| PRKD1 | Protein Kinase D1 | Protein Coding | Q15139 | 62 | GC14M029576 | 9.06326103210449 | https://www.genecards.org/cgi-bin/carddisp.pl?gene=PRKD1 |  |
| SPTSSB | Serine Palmitoyltransferase Small Subunit B | Protein Coding | Q8NFR3 | 43 | GC03M161344 | 9.01174354553223 | https://www.genecards.org/cgi-bin/carddisp.pl?gene=SPTSSB |  |
| SUMF2 | Sulfatase Modifying Factor 2 | Protein Coding | Q8NBJ7 | 47 | GC07P061675 | 9.00605297088623 | https://www.genecards.org/cgi-bin/carddisp.pl?gene=SUMF2 |  |
| SPP1 | Secreted Phosphoprotein 1 | Protein Coding | P10451 | 57 | GC04P087975 | 9.0026969909668 | https://www.genecards.org/cgi-bin/carddisp.pl?gene=SPP1 |  |
| PLCD1 | Phospholipase C Delta 1 | Protein Coding | P51178 | 61 | GC03M038008 | 8.98055839538574 | https://www.genecards.org/cgi-bin/carddisp.pl?gene=PLCD1 |  |
| OSBP | Oxysterol Binding Protein | Protein Coding | P22059 | 52 | GC11M139539 | 8.96688079833984 | https://www.genecards.org/cgi-bin/carddisp.pl?gene=OSBP |  |
| PLPBP | Pyridoxal Phosphate Binding Protein | Protein Coding | O94903 | 52 | GC08P044751 | 8.95893478393555 | https://www.genecards.org/cgi-bin/carddisp.pl?gene=PLPBP |  |
| MIR29C | MicroRNA 29c | RNA Gene |  | 27 | GC01M207838 | 8.94196319580078 | https://www.genecards.org/cgi-bin/carddisp.pl?gene=MIR29C |  |
| ADCY10 | Adenylate Cyclase 10 | Protein Coding | Q96PN6 | 56 | GC01M167809 | 8.92827224731445 | https://www.genecards.org/cgi-bin/carddisp.pl?gene=ADCY10 |  |
| ACSL1 | Acyl-CoA Synthetase Long Chain Family Member 1 | Protein Coding | P33121 | 59 | GC04M184755 | 8.92731285095215 | https://www.genecards.org/cgi-bin/carddisp.pl?gene=ACSL1 |  |
| HSPB1 | Heat Shock Protein Family B (Small) Member 1 | Protein Coding | P04792 | 65 | GC07P076302 | 8.9252872467041 | https://www.genecards.org/cgi-bin/carddisp.pl?gene=HSPB1 |  |
| TSPO | Translocator Protein | Protein Coding | B1AH88 | 58 | GC22P043151 | 8.91769504547119 | https://www.genecards.org/cgi-bin/carddisp.pl?gene=TSPO |  |
| GDAP1 | Ganglioside Induced Differentiation Associated Protein 1 | Protein Coding | Q8TB36 | 55 | GC08P074315 | 8.89756202697754 | https://www.genecards.org/cgi-bin/carddisp.pl?gene=GDAP1 |  |
| MMP2 | Matrix Metallopeptidase 2 | Protein Coding | P08253 | 66 | GC16P121565 | 8.86943244934082 | https://www.genecards.org/cgi-bin/carddisp.pl?gene=MMP2 |  |
| GATA4 | GATA Binding Protein 4 | Protein Coding | P43694 | 62 | GC08P011676 | 8.85859489440918 | https://www.genecards.org/cgi-bin/carddisp.pl?gene=GATA4 |  |
| TSC1 | TSC Complex Subunit 1 | Protein Coding | Q92574 | 61 | GC09M132891 | 8.84578132629395 | https://www.genecards.org/cgi-bin/carddisp.pl?gene=TSC1 |  |
| VEGFA | Vascular Endothelial Growth Factor A | Protein Coding | P15692 | 61 | GC06P043770 | 8.83834552764893 | https://www.genecards.org/cgi-bin/carddisp.pl?gene=VEGFA |  |
| PGR-AS1 | PGR Antisense RNA 1 | RNA Gene |  | 20 | GC11P108894 | 8.82142066955566 | https://www.genecards.org/cgi-bin/carddisp.pl?gene=PGR-AS1 |  |
| KNG1 | Kininogen 1 | Protein Coding | P01042 | 61 | GC03P186717 | 8.82059764862061 | https://www.genecards.org/cgi-bin/carddisp.pl?gene=KNG1 |  |
| JUN | Jun Proto-Oncogene, AP-1 Transcription Factor Subunit | Protein Coding | P05412 | 62 | GC01M058780 | 8.80540466308594 | https://www.genecards.org/cgi-bin/carddisp.pl?gene=JUN |  |
| EDN1 | Endothelin 1 | Protein Coding | P05305 | 61 | GC06P012910 | 8.75050354003906 | https://www.genecards.org/cgi-bin/carddisp.pl?gene=EDN1 |  |
| TERT | Telomerase Reverse Transcriptase | Protein Coding | O14746 | 64 | GC05M001253 | 8.73500823974609 | https://www.genecards.org/cgi-bin/carddisp.pl?gene=TERT |  |
| SLC26A1 | Solute Carrier Family 26 Member 1 | Protein Coding | Q9H2B4 | 51 | GC04M006254 | 8.70674705505371 | https://www.genecards.org/cgi-bin/carddisp.pl?gene=SLC26A1 |  |
| SP1 | Sp1 Transcription Factor | Protein Coding | P08047 | 60 | GC12P053380 | 8.70346450805664 | https://www.genecards.org/cgi-bin/carddisp.pl?gene=SP1 |  |
| NFKB1 | Nuclear Factor Kappa B Subunit 1 | Protein Coding | P19838 | 66 | GC04P102501 | 8.68879318237305 | https://www.genecards.org/cgi-bin/carddisp.pl?gene=NFKB1 |  |
| LRRK2 | Leucine Rich Repeat Kinase 2 | Protein Coding | Q5S007 | 62 | GC12P040196 | 8.6709566116333 | https://www.genecards.org/cgi-bin/carddisp.pl?gene=LRRK2 |  |
| MAP2K2 | Mitogen-Activated Protein Kinase Kinase 2 | Protein Coding | P36507 | 67 | GC19M004090 | 8.65905094146729 | https://www.genecards.org/cgi-bin/carddisp.pl?gene=MAP2K2 |  |
| ABCA7 | ATP Binding Cassette Subfamily A Member 7 | Protein Coding | Q8IZY2 | 58 | GC19P151373 | 8.63604736328125 | https://www.genecards.org/cgi-bin/carddisp.pl?gene=ABCA7 |  |
| GPX4 | Glutathione Peroxidase 4 | Protein Coding | P36969 | 60 | GC19P001103 | 8.58975219726563 | https://www.genecards.org/cgi-bin/carddisp.pl?gene=GPX4 |  |
| SORT1 | Sortilin 1 | Protein Coding | Q99523 | 58 | GC01M109310 | 8.55669593811035 | https://www.genecards.org/cgi-bin/carddisp.pl?gene=SORT1 |  |
| FOS | Fos Proto-Oncogene, AP-1 Transcription Factor Subunit | Protein Coding | P01100 | 64 | GC14P075278 | 8.55155849456787 | https://www.genecards.org/cgi-bin/carddisp.pl?gene=FOS |  |
| TFEB | Transcription Factor EB | Protein Coding | P19484 | 55 | GC06M106693 | 8.54526805877686 | https://www.genecards.org/cgi-bin/carddisp.pl?gene=TFEB |  |
| CBL | Cbl Proto-Oncogene | Protein Coding | P22681 | 64 | GC11P119206 | 8.54396820068359 | https://www.genecards.org/cgi-bin/carddisp.pl?gene=CBL |  |
| BECN1 | Beclin 1 | Protein Coding | Q14457 | 59 | GC17M042810 | 8.53634262084961 | https://www.genecards.org/cgi-bin/carddisp.pl?gene=BECN1 |  |
| IL2 | Interleukin 2 | Protein Coding | P60568 | 58 | GC04M122451 | 8.53532218933105 | https://www.genecards.org/cgi-bin/carddisp.pl?gene=IL2 |  |
| ICAM1 | Intercellular Adhesion Molecule 1 | Protein Coding | P05362 | 63 | GC19P151796 | 8.52854919433594 | https://www.genecards.org/cgi-bin/carddisp.pl?gene=ICAM1 |  |
| PDGFRB | Platelet Derived Growth Factor Receptor Beta | Protein Coding | P09619 | 67 | GC05M150113 | 8.48571586608887 | https://www.genecards.org/cgi-bin/carddisp.pl?gene=PDGFRB |  |
| ATP2A2 | ATPase Sarcoplasmic/Endoplasmic Reticulum Ca2+ Transporting 2 | Protein Coding | P16615 | 64 | GC12P110280 | 8.42782115936279 | https://www.genecards.org/cgi-bin/carddisp.pl?gene=ATP2A2 |  |
| B4GALT5 | Beta-1,4-Galactosyltransferase 5 | Protein Coding | O43286 | 48 | GC20M049632 | 8.41281700134277 | https://www.genecards.org/cgi-bin/carddisp.pl?gene=B4GALT5 |  |
| HACL1 | 2-Hydroxyacyl-CoA Lyase 1 | Protein Coding | Q9UJ83 | 50 | GC03M028707 | 8.40820789337158 | https://www.genecards.org/cgi-bin/carddisp.pl?gene=HACL1 |  |
| ACE2 | Angiotensin Converting Enzyme 2 | Protein Coding | Q9BYF1 | 62 | GC0XM015494 | 8.40458297729492 | https://www.genecards.org/cgi-bin/carddisp.pl?gene=ACE2 |  |
| MAPK10 | Mitogen-Activated Protein Kinase 10 | Protein Coding | P53779 | 60 | GC04M085990 | 8.40121173858643 | https://www.genecards.org/cgi-bin/carddisp.pl?gene=MAPK10 |  |
| VWF | Von Willebrand Factor | Protein Coding | P04275 | 61 | GC12M035631 | 8.4007625579834 | https://www.genecards.org/cgi-bin/carddisp.pl?gene=VWF |  |
| TXN | Thioredoxin | Protein Coding | P10599 | 59 | GC09M110243 | 8.36367893218994 | https://www.genecards.org/cgi-bin/carddisp.pl?gene=TXN |  |
| AGPAT1 | 1-Acylglycerol-3-Phosphate O-Acyltransferase 1 | Protein Coding | Q99943 | 51 | GC06M032168 | 8.35713386535645 | https://www.genecards.org/cgi-bin/carddisp.pl?gene=AGPAT1 |  |
| VAPB | VAMP Associated Protein B And C | Protein Coding | O95292 | 57 | GC20P058389 | 8.34055709838867 | https://www.genecards.org/cgi-bin/carddisp.pl?gene=VAPB |  |
| ENTPD1 | Ectonucleoside Triphosphate Diphosphohydrolase 1 | Protein Coding | P49961 | 62 | GC10P124203 | 8.3105297088623 | https://www.genecards.org/cgi-bin/carddisp.pl?gene=ENTPD1 |  |
| FOXP3 | Forkhead Box P3 | Protein Coding | Q9BZS1 | 59 | GC0XM049250 | 8.30551910400391 | https://www.genecards.org/cgi-bin/carddisp.pl?gene=FOXP3 |  |
| PARK7 | Parkinsonism Associated Deglycase | Protein Coding | Q99497 | 60 | GC01P075179 | 8.28668308258057 | https://www.genecards.org/cgi-bin/carddisp.pl?gene=PARK7 |  |
| SMAD2 | SMAD Family Member 2 | Protein Coding | Q15796 | 65 | GC18M047809 | 8.28504371643066 | https://www.genecards.org/cgi-bin/carddisp.pl?gene=SMAD2 |  |
| SELE | Selectin E | Protein Coding | P16581 | 57 | GC01M169722 | 8.27939510345459 | https://www.genecards.org/cgi-bin/carddisp.pl?gene=SELE |  |
| SLC10A2 | Solute Carrier Family 10 Member 2 | Protein Coding | Q12908 | 54 | GC13M103043 | 8.26270961761475 | https://www.genecards.org/cgi-bin/carddisp.pl?gene=SLC10A2 |  |
| PLIN2 | Perilipin 2 | Protein Coding | Q99541 | 54 | GC09M019319 | 8.25816249847412 | https://www.genecards.org/cgi-bin/carddisp.pl?gene=PLIN2 |  |
| ST8SIA1 | ST8 Alpha-N-Acetyl-Neuraminide Alpha-2,8-Sialyltransferase 1 | Protein Coding | Q92185 | 53 | GC12M022063 | 8.24389839172363 | https://www.genecards.org/cgi-bin/carddisp.pl?gene=ST8SIA1 |  |
| SELP | Selectin P | Protein Coding | P16109 | 59 | GC01M169558 | 8.22111225128174 | https://www.genecards.org/cgi-bin/carddisp.pl?gene=SELP |  |
| ATP1A1 | ATPase Na+/K+ Transporting Subunit Alpha 1 | Protein Coding | P05023 | 62 | GC01P116372 | 8.21884346008301 | https://www.genecards.org/cgi-bin/carddisp.pl?gene=ATP1A1 |  |
| TRA-TGC7-1 | TRNA-Ala (Anticodon TGC) 7-1 | RNA Gene |  | 14 | GC06M106311 | 8.20896339416504 | https://www.genecards.org/cgi-bin/carddisp.pl?gene=TRA-TGC7-1 |  |
| PRKCA | Protein Kinase C Alpha | Protein Coding | P17252 | 64 | GC17P066302 | 8.1629695892334 | https://www.genecards.org/cgi-bin/carddisp.pl?gene=PRKCA |  |
| CXCR4 | C-X-C Motif Chemokine Receptor 4 | Protein Coding | P61073 | 65 | GC02M136114 | 8.15825748443604 | https://www.genecards.org/cgi-bin/carddisp.pl?gene=CXCR4 |  |
| MYBPC3 | Myosin Binding Protein C3 | Protein Coding | Q14896 | 60 | GC11M139452 | 8.15364933013916 | https://www.genecards.org/cgi-bin/carddisp.pl?gene=MYBPC3 |  |
| HMGA1 | High Mobility Group AT-Hook 1 | Protein Coding | P17096 | 59 | GC06P181988 | 8.15316104888916 | https://www.genecards.org/cgi-bin/carddisp.pl?gene=HMGA1 |  |
| TFAP2A | Transcription Factor AP-2 Alpha | Protein Coding | P05549 | 60 | GC06M010393 | 8.13943767547607 | https://www.genecards.org/cgi-bin/carddisp.pl?gene=TFAP2A |  |
| AARS1 | Alanyl-TRNA Synthetase 1 | Protein Coding | P49588 | 56 | GC16M075123 | 8.1095438 | https://www.genecards.org/cgi-bin/carddisp.pl?gene=AARS1 |  |
| TNFRSF11A | TNF Receptor Superfamily Member 11a | Protein Coding | Q9Y6Q6 | 59 | GC18P062325 | 8.10344886779785 | https://www.genecards.org/cgi-bin/carddisp.pl?gene=TNFRSF11A |  |
| PLPP2 | Phospholipid Phosphatase 2 | Protein Coding | O43688 | 50 | GC19M107685 | 8.08695697784424 | https://www.genecards.org/cgi-bin/carddisp.pl?gene=PLPP2 |  |
| SAMD8 | Sterile Alpha Motif Domain Containing 8 | Protein Coding | Q96LT4 | 46 | GC10P075099 | 8.07254695892334 | https://www.genecards.org/cgi-bin/carddisp.pl?gene=SAMD8 |  |
| ATP2A1 | ATPase Sarcoplasmic/Endoplasmic Reticulum Ca2+ Transporting 1 | Protein Coding | O14983 | 62 | GC16P120907 | 8.07070159912109 | https://www.genecards.org/cgi-bin/carddisp.pl?gene=ATP2A1 |  |
| PRNP | Prion Protein (Kanno Blood Group) | Protein Coding | F7VJQ1 | 61 | GC20P004686 | 8.05377769470215 | https://www.genecards.org/cgi-bin/carddisp.pl?gene=PRNP |  |
| PYGB | Glycogen Phosphorylase B | Protein Coding | P11216 | 55 | GC20P025248 | 8.04841423034668 | https://www.genecards.org/cgi-bin/carddisp.pl?gene=PYGB |  |
| ELOVL5 | ELOVL Fatty Acid Elongase 5 | Protein Coding | Q9NYP7 | 55 | GC06M053267 | 8.0246639251709 | https://www.genecards.org/cgi-bin/carddisp.pl?gene=ELOVL5 |  |
| PLEK | Pleckstrin | Protein Coding | P08567 | 52 | GC02P068365 | 8.01635456085205 | https://www.genecards.org/cgi-bin/carddisp.pl?gene=PLEK |  |
| SERINC1 | Serine Incorporator 1 | Protein Coding | Q9NRX5 | 51 | GC06M122443 | 8.00067710876465 | https://www.genecards.org/cgi-bin/carddisp.pl?gene=SERINC1 |  |
| APOH | Apolipoprotein H | Protein Coding | P02749 | 58 | GC17M066212 | 7.99563074111938 | https://www.genecards.org/cgi-bin/carddisp.pl?gene=APOH |  |
| YAP1 | Yes1 Associated Transcriptional Regulator | Protein Coding | P46937 | 61 | GC11P102110 | 7.97304630279541 | https://www.genecards.org/cgi-bin/carddisp.pl?gene=YAP1 |  |
| RPS6KB1 | Ribosomal Protein S6 Kinase B1 | Protein Coding | P23443 | 63 | GC17P059893 | 7.96817064285278 | https://www.genecards.org/cgi-bin/carddisp.pl?gene=RPS6KB1 |  |
| BCR | BCR Activator Of RhoGEF And GTPase | Protein Coding | P11274 | 65 | GC22P023179 | 7.94665908813477 | https://www.genecards.org/cgi-bin/carddisp.pl?gene=BCR |  |
| ARF1 | ARF GTPase 1 | Protein Coding | P84077 | 58 | GC01P228082 | 7.92578792572021 | https://www.genecards.org/cgi-bin/carddisp.pl?gene=ARF1 |  |
| CD4 | CD4 Molecule | Protein Coding | P01730 | 63 | GC12P006786 | 7.87767219543457 | https://www.genecards.org/cgi-bin/carddisp.pl?gene=CD4 |  |
| IL4 | Interleukin 4 | Protein Coding | P05112 | 59 | GC05P132673 | 7.83316802978516 | https://www.genecards.org/cgi-bin/carddisp.pl?gene=IL4 |  |
| LACC1 | Laccase Domain Containing 1 | Protein Coding | Q8IV20 | 47 | GC13P043879 | 7.82590961456299 | https://www.genecards.org/cgi-bin/carddisp.pl?gene=LACC1 |  |
| HDAC1 | Histone Deacetylase 1 | Protein Coding | Q13547 | 63 | GC01P032292 | 7.80965280532837 | https://www.genecards.org/cgi-bin/carddisp.pl?gene=HDAC1 |  |
| PCYT2 | Phosphate Cytidylyltransferase 2, Ethanolamine | Protein Coding | Q99447 | 56 | GC17M081900 | 7.76682710647583 | https://www.genecards.org/cgi-bin/carddisp.pl?gene=PCYT2 |  |
| M6PR | Mannose-6-Phosphate Receptor, Cation Dependent | Protein Coding | P20645 | 56 | GC12M035768 | 7.7274751663208 | https://www.genecards.org/cgi-bin/carddisp.pl?gene=M6PR |  |
| MGAM | Maltase-Glucoamylase | Protein Coding | O43451 | 57 | GC07P167658 | 7.70661544799805 | https://www.genecards.org/cgi-bin/carddisp.pl?gene=MGAM |  |
| CSNK2A1 | Casein Kinase 2 Alpha 1 | Protein Coding | P68400 | 63 | GC20M000472 | 7.69749164581299 | https://www.genecards.org/cgi-bin/carddisp.pl?gene=CSNK2A1 |  |
| SNORD15A | Small Nucleolar RNA, C/D Box 15A | RNA Gene |  | 25 | GC11P075400 | 7.67657423019409 | https://www.genecards.org/cgi-bin/carddisp.pl?gene=SNORD15A |  |
| CREB1 | CAMP Responsive Element Binding Protein 1 | Protein Coding | P16220 | 62 | GC02P207529 | 7.66406440734863 | https://www.genecards.org/cgi-bin/carddisp.pl?gene=CREB1 |  |
| EIF2AK2 | Eukaryotic Translation Initiation Factor 2 Alpha Kinase 2 | Protein Coding | P19525 | 62 | GC02M037099 | 7.65567588806152 | https://www.genecards.org/cgi-bin/carddisp.pl?gene=EIF2AK2 |  |
| GJB2 | Gap Junction Protein Beta 2 | Protein Coding | P29033 | 58 | GC13M020187 | 7.64370107650757 | https://www.genecards.org/cgi-bin/carddisp.pl?gene=GJB2 |  |
| CD44 | CD44 Molecule (IN Blood Group) | Protein Coding | P16070 | 61 | GC11P035139 | 7.63008689880371 | https://www.genecards.org/cgi-bin/carddisp.pl?gene=CD44 |  |
| PPP2CA | Protein Phosphatase 2 Catalytic Subunit Alpha | Protein Coding | P67775 | 63 | GC05M134194 | 7.61768627166748 | https://www.genecards.org/cgi-bin/carddisp.pl?gene=PPP2CA |  |
| TLR2 | Toll Like Receptor 2 | Protein Coding | O60603 | 65 | GC04P153684 | 7.59816837310791 | https://www.genecards.org/cgi-bin/carddisp.pl?gene=TLR2 |  |
| TXNRD1 | Thioredoxin Reductase 1 | Protein Coding | Q16881 | 57 | GC12P104215 | 7.59801912307739 | https://www.genecards.org/cgi-bin/carddisp.pl?gene=TXNRD1 |  |
| SLC27A2 | Solute Carrier Family 27 Member 2 | Protein Coding | O14975 | 57 | GC15P050182 | 7.57395362854004 | https://www.genecards.org/cgi-bin/carddisp.pl?gene=SLC27A2 |  |
| DNM1 | Dynamin 1 | Protein Coding | Q05193 | 63 | GC09P128191 | 7.50454998016357 | https://www.genecards.org/cgi-bin/carddisp.pl?gene=DNM1 |  |
| ELOVL7 | ELOVL Fatty Acid Elongase 7 | Protein Coding | A1L3X0 | 48 | GC05M060751 | 7.50139236450195 | https://www.genecards.org/cgi-bin/carddisp.pl?gene=ELOVL7 |  |
| BTK | Bruton Tyrosine Kinase | Protein Coding | Q06187 | 67 | GC0XM101349 | 7.48802518844604 | https://www.genecards.org/cgi-bin/carddisp.pl?gene=BTK |  |
| TIMP1 | TIMP Metallopeptidase Inhibitor 1 | Protein Coding | P01033 | 57 | GC0XP060812 | 7.47803449630737 | https://www.genecards.org/cgi-bin/carddisp.pl?gene=TIMP1 |  |
| SERPINF1 | Serpin Family F Member 1 | Protein Coding | P36955 | 58 | GC17P153476 | 7.47124862670898 | https://www.genecards.org/cgi-bin/carddisp.pl?gene=SERPINF1 |  |
| SLC7A5 | Solute Carrier Family 7 Member 5 | Protein Coding | Q01650 | 57 | GC16M087830 | 7.47069454193115 | https://www.genecards.org/cgi-bin/carddisp.pl?gene=SLC7A5 |  |
| DBI | Diazepam Binding Inhibitor, Acyl-CoA Binding Protein | Protein Coding | P07108 | 58 | GC02P119366 | 7.45210313796997 | https://www.genecards.org/cgi-bin/carddisp.pl?gene=DBI |  |
| CDK2 | Cyclin Dependent Kinase 2 | Protein Coding | P24941 | 64 | GC12P055966 | 7.44895315170288 | https://www.genecards.org/cgi-bin/carddisp.pl?gene=CDK2 |  |
| HSP90B1 | Heat Shock Protein 90 Beta Family Member 1 | Protein Coding | P14625 | 60 | GC12P103930 | 7.42919206619263 | https://www.genecards.org/cgi-bin/carddisp.pl?gene=HSP90B1 |  |
| CD38 | CD38 Molecule | Protein Coding | P28907 | 59 | GC04P030063 | 7.42140913009644 | https://www.genecards.org/cgi-bin/carddisp.pl?gene=CD38 |  |
| IL17A | Interleukin 17A | Protein Coding | Q16552 | 55 | GC06P052186 | 7.3773627281189 | https://www.genecards.org/cgi-bin/carddisp.pl?gene=IL17A |  |
| RAC1 | Rac Family Small GTPase 1 | Protein Coding | P63000 | 62 | GC07P019033 | 7.37478685379028 | https://www.genecards.org/cgi-bin/carddisp.pl?gene=RAC1 |  |
| NTRK1 | Neurotrophic Receptor Tyrosine Kinase 1 | Protein Coding | P04629 | 62 | GC01P156815 | 7.35980844497681 | https://www.genecards.org/cgi-bin/carddisp.pl?gene=NTRK1 |  |
| HNRNPU | Heterogeneous Nuclear Ribonucleoprotein U | Protein Coding | Q00839 | 55 | GC01M244853 | 7.3406023979187 | https://www.genecards.org/cgi-bin/carddisp.pl?gene=HNRNPU |  |
| APOL1 | Apolipoprotein L1 | Protein Coding | O14791 | 56 | GC22P036253 | 7.28312397003174 | https://www.genecards.org/cgi-bin/carddisp.pl?gene=APOL1 |  |
| USF1 | Upstream Transcription Factor 1 | Protein Coding | P22415 | 55 | GC01M161039 | 7.27892065048218 | https://www.genecards.org/cgi-bin/carddisp.pl?gene=USF1 |  |
| VCAM1 | Vascular Cell Adhesion Molecule 1 | Protein Coding | P19320 | 58 | GC01P100719 | 7.26880741119385 | https://www.genecards.org/cgi-bin/carddisp.pl?gene=VCAM1 |  |
| S1PR3 | Sphingosine-1-Phosphate Receptor 3 | Protein Coding | Q99500 | 57 | GC09P106821 | 7.2576150894165 | https://www.genecards.org/cgi-bin/carddisp.pl?gene=S1PR3 |  |
| ZAP70 | Zeta Chain Of T Cell Receptor Associated Protein Kinase 70 | Protein Coding | P43403 | 64 | GC02P100091 | 7.25469875335693 | https://www.genecards.org/cgi-bin/carddisp.pl?gene=ZAP70 |  |
| UBE3A | Ubiquitin Protein Ligase E3A | Protein Coding | Q05086 | 61 | GC15M025333 | 7.24241065979004 | https://www.genecards.org/cgi-bin/carddisp.pl?gene=UBE3A |  |
| HSD17B12 | Hydroxysteroid 17-Beta Dehydrogenase 12 | Protein Coding | Q53GQ0 | 50 | GC11P043652 | 7.23290205001831 | https://www.genecards.org/cgi-bin/carddisp.pl?gene=HSD17B12 |  |
| CD40 | CD40 Molecule | Protein Coding | P25942 | 63 | GC20P046118 | 7.2295708656311 | https://www.genecards.org/cgi-bin/carddisp.pl?gene=CD40 |  |
| NOTCH1 | Notch Receptor 1 | Protein Coding | P46531 | 66 | GC09M139264 | 7.21805906295776 | https://www.genecards.org/cgi-bin/carddisp.pl?gene=NOTCH1 |  |
| ARCN1 | Archain 1 | Protein Coding | P48444 | 55 | GC11P118572 | 7.18420553207397 | https://www.genecards.org/cgi-bin/carddisp.pl?gene=ARCN1 |  |
| ITPR1 | Inositol 1,4,5-Trisphosphate Receptor Type 1 | Protein Coding | Q14643 | 62 | GC03P004496 | 7.18150329589844 | https://www.genecards.org/cgi-bin/carddisp.pl?gene=ITPR1 |  |
| LCK | LCK Proto-Oncogene, Src Family Tyrosine Kinase | Protein Coding | P06239 | 65 | GC01P032251 | 7.17691087722778 | https://www.genecards.org/cgi-bin/carddisp.pl?gene=LCK |  |
| B3GALT4 | Beta-1,3-Galactosyltransferase 4 | Protein Coding | O96024 | 52 | GC06P033277 | 7.16604566574097 | https://www.genecards.org/cgi-bin/carddisp.pl?gene=B3GALT4 |  |
| BAX | BCL2 Associated X, Apoptosis Regulator | Protein Coding | Q07812 | 64 | GC19P048954 | 7.15789842605591 | https://www.genecards.org/cgi-bin/carddisp.pl?gene=BAX |  |
| MIR145 | MicroRNA 145 | RNA Gene |  | 32 | GC05P149430 | 7.1526951789856 | https://www.genecards.org/cgi-bin/carddisp.pl?gene=MIR145 |  |
| ENPP7 | Ectonucleotide Pyrophosphatase/Phosphodiesterase 7 | Protein Coding | Q6UWV6 | 51 | GC17P079730 | 7.14707088470459 | https://www.genecards.org/cgi-bin/carddisp.pl?gene=ENPP7 |  |
| HLA-DQB1 | Major Histocompatibility Complex, Class II, DQ Beta 1 | Protein Coding | P01920 | 57 | GC06M106540 | 7.13553953170776 | https://www.genecards.org/cgi-bin/carddisp.pl?gene=HLA-DQB1 |  |
| IGF2R | Insulin Like Growth Factor 2 Receptor | Protein Coding | P11717 | 60 | GC06P159969 | 7.13099718093872 | https://www.genecards.org/cgi-bin/carddisp.pl?gene=IGF2R |  |
| FOXH1 | Forkhead Box H1 | Protein Coding | O75593 | 52 | GC08M144473 | 7.0859203338623 | https://www.genecards.org/cgi-bin/carddisp.pl?gene=FOXH1 |  |
| TNFRSF1A | TNF Receptor Superfamily Member 1A | Protein Coding | P19438 | 62 | GC12M006328 | 7.06713438034058 | https://www.genecards.org/cgi-bin/carddisp.pl?gene=TNFRSF1A |  |
| ADPRH | ADP-Ribosylarginine Hydrolase | Protein Coding | P54922 | 48 | GC03P119579 | 7.03470849990845 | https://www.genecards.org/cgi-bin/carddisp.pl?gene=ADPRH |  |
| TRC-GCA24-1 | TRNA-Cys (GCA) 24-1 | RNA Gene |  | 14 | GC17M097073 | 7.02857971191406 | https://www.genecards.org/cgi-bin/carddisp.pl?gene=TRC-GCA24-1 |  |
| HACD1 | 3-Hydroxyacyl-CoA Dehydratase 1 | Protein Coding | B0YJ81 | 52 | GC10M017589 | 7.02432918548584 | https://www.genecards.org/cgi-bin/carddisp.pl?gene=HACD1 |  |
| ERN1 | Endoplasmic Reticulum To Nucleus Signaling 1 | Protein Coding | O75460 | 60 | GC17M064039 | 7.02399444580078 | https://www.genecards.org/cgi-bin/carddisp.pl?gene=ERN1 |  |
| ACHE | Acetylcholinesterase (Yt Blood Group) | Protein Coding | P22303 | 61 | GC07M100889 | 7.02294778823853 | https://www.genecards.org/cgi-bin/carddisp.pl?gene=ACHE |  |
| VPS53 | VPS53 Subunit Of GARP Complex | Protein Coding | Q5VIR6 | 53 | GC17M000508 | 7.00952625274658 | https://www.genecards.org/cgi-bin/carddisp.pl?gene=VPS53 |  |
| ARSK | Arylsulfatase Family Member K | Protein Coding | Q6UWY0 | 48 | GC05P095555 | 7.00616025924683 | https://www.genecards.org/cgi-bin/carddisp.pl?gene=ARSK |  |
| MIR133B | MicroRNA 133b | RNA Gene |  | 31 | GC06P052148 | 7.00608587265015 | https://www.genecards.org/cgi-bin/carddisp.pl?gene=MIR133B |  |
| VAMP2 | Vesicle Associated Membrane Protein 2 | Protein Coding | P63027 | 58 | GC17M096233 | 7.00198411941528 | https://www.genecards.org/cgi-bin/carddisp.pl?gene=VAMP2 |  |
| MBTPS1 | Membrane Bound Transcription Factor Peptidase, Site 1 | Protein Coding | Q14703 | 55 | GC16M084053 | 6.9670467376709 | https://www.genecards.org/cgi-bin/carddisp.pl?gene=MBTPS1 |  |
| COL1A2 | Collagen Type I Alpha 2 Chain | Protein Coding | P08123 | 61 | GC07P094394 | 6.95170116424561 | https://www.genecards.org/cgi-bin/carddisp.pl?gene=COL1A2 |  |
| TFPT | TCF3 Fusion Partner | Protein Coding | P0C1Z6 | 45 | GC19M054107 | 6.94404792785645 | https://www.genecards.org/cgi-bin/carddisp.pl?gene=TFPT |  |
| CDIPT | CDP-Diacylglycerol--Inositol 3-Phosphatidyltransferase | Protein Coding | O14735 | 53 | GC16M051044 | 6.89886236190796 | https://www.genecards.org/cgi-bin/carddisp.pl?gene=CDIPT |  |
| DLG4 | Discs Large MAGUK Scaffold Protein 4 | Protein Coding | P78352 | 61 | GC17M096179 | 6.89665555953979 | https://www.genecards.org/cgi-bin/carddisp.pl?gene=DLG4 |  |
| NR2F2 | Nuclear Receptor Subfamily 2 Group F Member 2 | Protein Coding | P24468 | 61 | GC15P096325 | 6.89184188842773 | https://www.genecards.org/cgi-bin/carddisp.pl?gene=NR2F2 |  |
| CDK4 | Cyclin Dependent Kinase 4 | Protein Coding | P11802 | 67 | GC12M060483 | 6.88200092315674 | https://www.genecards.org/cgi-bin/carddisp.pl?gene=CDK4 |  |
| ST3GAL2 | ST3 Beta-Galactoside Alpha-2,3-Sialyltransferase 2 | Protein Coding | Q16842 | 52 | GC16M070375 | 6.8721718788147 | https://www.genecards.org/cgi-bin/carddisp.pl?gene=ST3GAL2 |  |
| ACSL5 | Acyl-CoA Synthetase Long Chain Family Member 5 | Protein Coding | Q9ULC5 | 54 | GC10P112374 | 6.86633110046387 | https://www.genecards.org/cgi-bin/carddisp.pl?gene=ACSL5 |  |
| A4GALT | Alpha 1,4-Galactosyltransferase (P1PK Blood Group) | Protein Coding | Q9NPC4 | 54 | GC22M042692 | 6.86565065383911 | https://www.genecards.org/cgi-bin/carddisp.pl?gene=A4GALT |  |
| ELANE | Elastase, Neutrophil Expressed | Protein Coding | P08246 | 64 | GC19P151362 | 6.84025573730469 | https://www.genecards.org/cgi-bin/carddisp.pl?gene=ELANE |  |
| PVT1 | Pvt1 Oncogene | RNA Gene |  | 33 | GC08P128394 | 6.83320140838623 | https://www.genecards.org/cgi-bin/carddisp.pl?gene=PVT1 |  |
| DES | Desmin | Protein Coding | P17661 | 61 | GC02P219418 | 6.83215236663818 | https://www.genecards.org/cgi-bin/carddisp.pl?gene=DES |  |
| HNRNPH2 | Heterogeneous Nuclear Ribonucleoprotein H2 | Protein Coding | P55795 | 53 | GC0XP101408 | 6.80670785903931 | https://www.genecards.org/cgi-bin/carddisp.pl?gene=HNRNPH2 |  |
| MMP1 | Matrix Metallopeptidase 1 | Protein Coding | P03956 | 62 | GC11M140392 | 6.80567455291748 | https://www.genecards.org/cgi-bin/carddisp.pl?gene=MMP1 |  |
| TEX2 | Testis Expressed 2 | Protein Coding | Q8IWB9 | 44 | GC17M064147 | 6.79559326171875 | https://www.genecards.org/cgi-bin/carddisp.pl?gene=TEX2 |  |
| THBS1 | Thrombospondin 1 | Protein Coding | P07996 | 59 | GC15P039581 | 6.76885223388672 | https://www.genecards.org/cgi-bin/carddisp.pl?gene=THBS1 |  |
| HSPA4 | Heat Shock Protein Family A (Hsp70) Member 4 | Protein Coding | P34932 | 55 | GC05P133132 | 6.75734376907349 | https://www.genecards.org/cgi-bin/carddisp.pl?gene=HSPA4 |  |
| PLG | Plasminogen | Protein Coding | P00747 | 62 | GC06P160702 | 6.74469995498657 | https://www.genecards.org/cgi-bin/carddisp.pl?gene=PLG |  |
| HDC | Histidine Decarboxylase | Protein Coding | P19113 | 58 | GC15M050241 | 6.74416637420654 | https://www.genecards.org/cgi-bin/carddisp.pl?gene=HDC |  |
| APOM | Apolipoprotein M | Protein Coding | O95445 | 51 | GC06P181900 | 6.73912668228149 | https://www.genecards.org/cgi-bin/carddisp.pl?gene=APOM |  |
| PPP1CA | Protein Phosphatase 1 Catalytic Subunit Alpha | Protein Coding | P62136 | 61 | GC11M139864 | 6.72976064682007 | https://www.genecards.org/cgi-bin/carddisp.pl?gene=PPP1CA |  |
| RAPSN | Receptor Associated Protein Of The Synapse | Protein Coding | Q13702 | 54 | GC11M139455 | 6.72600841522217 | https://www.genecards.org/cgi-bin/carddisp.pl?gene=RAPSN |  |
| CDC42 | Cell Division Cycle 42 | Protein Coding | P60953 | 63 | GC01P022052 | 6.68914794921875 | https://www.genecards.org/cgi-bin/carddisp.pl?gene=CDC42 |  |
| FN1 | Fibronectin 1 | Protein Coding | P02751 | 63 | GC02M215360 | 6.68334293365479 | https://www.genecards.org/cgi-bin/carddisp.pl?gene=FN1 |  |
| DOCK7 | Dedicator Of Cytokinesis 7 | Protein Coding | Q96N67 | 54 | GC01M062454 | 6.67553186416626 | https://www.genecards.org/cgi-bin/carddisp.pl?gene=DOCK7 |  |
| NGF | Nerve Growth Factor | Protein Coding | P01138 | 64 | GC01M115285 | 6.66247844696045 | https://www.genecards.org/cgi-bin/carddisp.pl?gene=NGF |  |
| TRP-AGG2-5 | TRNA-Pro (Anticodon AGG) 2-5 | RNA Gene |  | 18 | GC14M028662 | 6.66225147247314 | https://www.genecards.org/cgi-bin/carddisp.pl?gene=TRP-AGG2-5 |  |
| CYB5B | Cytochrome B5 Type B | Protein Coding | O43169 | 54 | GC16P122007 | 6.65317153930664 | https://www.genecards.org/cgi-bin/carddisp.pl?gene=CYB5B |  |
| TUG1 | Taurine Up-Regulated 1 | Protein Coding | A0A6I8PU40 | 34 | GC22P030969 | 6.63638067245483 | https://www.genecards.org/cgi-bin/carddisp.pl?gene=TUG1 |  |
| HLA-DRB1 | Major Histocompatibility Complex, Class II, DR Beta 1 | Protein Coding | P01911 | 60 | GC06M106528 | 6.63474798202515 | https://www.genecards.org/cgi-bin/carddisp.pl?gene=HLA-DRB1 |  |
| TRPM3 | Transient Receptor Potential Cation Channel Subfamily M Member 3 | Protein Coding | Q9HCF6 | 54 | GC09M070529 | 6.6326060295105 | https://www.genecards.org/cgi-bin/carddisp.pl?gene=TRPM3 |  |
| NCOA2 | Nuclear Receptor Coactivator 2 | Protein Coding | Q15596 | 57 | GC08M070109 | 6.61099910736084 | https://www.genecards.org/cgi-bin/carddisp.pl?gene=NCOA2 |  |
| SMPD4 | Sphingomyelin Phosphodiesterase 4 | Protein Coding | Q9NXE4 | 52 | GC02M130151 | 6.5727219581604 | https://www.genecards.org/cgi-bin/carddisp.pl?gene=SMPD4 |  |
| RHOA | Ras Homolog Family Member A | Protein Coding | P61586 | 62 | GC03M049359 | 6.57055187225342 | https://www.genecards.org/cgi-bin/carddisp.pl?gene=RHOA |  |
| YWHAG | Tyrosine 3-Monooxygenase/Tryptophan 5-Monooxygenase Activation Protein Gamma | Protein Coding | P61981 | 61 | GC07M081119 | 6.56973028182983 | https://www.genecards.org/cgi-bin/carddisp.pl?gene=YWHAG |  |
| EZR | Ezrin | Protein Coding | P15311 | 59 | GC06M158765 | 6.54175519943237 | https://www.genecards.org/cgi-bin/carddisp.pl?gene=EZR |  |
| PDGFB | Platelet Derived Growth Factor Subunit B | Protein Coding | P01127 | 64 | GC22M086976 | 6.53567123413086 | https://www.genecards.org/cgi-bin/carddisp.pl?gene=PDGFB |  |
| RAB7A | RAB7A, Member RAS Oncogene Family | Protein Coding | P51149 | 62 | GC03P143091 | 6.50567436218262 | https://www.genecards.org/cgi-bin/carddisp.pl?gene=RAB7A |  |
| PTCH1 | Patched 1 | Protein Coding | Q13635 | 64 | GC09M095442 | 6.50122165679932 | https://www.genecards.org/cgi-bin/carddisp.pl?gene=PTCH1 |  |
| KEAP1 | Kelch Like ECH Associated Protein 1 | Protein Coding | Q14145 | 61 | GC19M010486 | 6.49948215484619 | https://www.genecards.org/cgi-bin/carddisp.pl?gene=KEAP1 |  |
| DDIT3 | DNA Damage Inducible Transcript 3 | Protein Coding | P35638 | 59 | GC12M057516 | 6.49125289916992 | https://www.genecards.org/cgi-bin/carddisp.pl?gene=DDIT3 |  |
| DLG2 | Discs Large MAGUK Scaffold Protein 2 | Protein Coding | Q15700 | 55 | GC11M083455 | 6.48932695388794 | https://www.genecards.org/cgi-bin/carddisp.pl?gene=DLG2 |  |
| PLA2G10 | Phospholipase A2 Group X | Protein Coding | O15496 | 55 | GC16M014672 | 6.48753547668457 | https://www.genecards.org/cgi-bin/carddisp.pl?gene=PLA2G10 |  |
| P4HB | Prolyl 4-Hydroxylase Subunit Beta | Protein Coding | P07237 | 62 | GC17M081843 | 6.48584985733032 | https://www.genecards.org/cgi-bin/carddisp.pl?gene=P4HB |  |
| ZPR1 | ZPR1 Zinc Finger | Protein Coding | O75312 | 51 | GC11M140583 | 6.48089694976807 | https://www.genecards.org/cgi-bin/carddisp.pl?gene=ZPR1 |  |
| CIITA | Class II Major Histocompatibility Complex Transactivator | Protein Coding | P33076 | 56 | GC16P120364 | 6.4745831489563 | https://www.genecards.org/cgi-bin/carddisp.pl?gene=CIITA |  |
| SGK1 | Serum/Glucocorticoid Regulated Kinase 1 | Protein Coding | O00141 | 62 | GC06M134169 | 6.46704959869385 | https://www.genecards.org/cgi-bin/carddisp.pl?gene=SGK1 |  |
| RAB11A | RAB11A, Member RAS Oncogene Family | Protein Coding | P62491 | 58 | GC15P193373 | 6.45188760757446 | https://www.genecards.org/cgi-bin/carddisp.pl?gene=RAB11A |  |
| SLC11A1 | Solute Carrier Family 11 Member 1 | Protein Coding | P49279 | 59 | GC02P218382 | 6.44352197647095 | https://www.genecards.org/cgi-bin/carddisp.pl?gene=SLC11A1 |  |
| RPL36A-HNRNPH2 | RPL36A-HNRNPH2 Readthrough | Protein Coding |  | 25 | GC0XP101423 | 6.42672157287598 | https://www.genecards.org/cgi-bin/carddisp.pl?gene=RPL36A-HNRNPH2 |  |
| ATF6 | Activating Transcription Factor 6 | Protein Coding | P18850 | 61 | GC01P161766 | 6.42424583435059 | https://www.genecards.org/cgi-bin/carddisp.pl?gene=ATF6 |  |
| ACSL6 | Acyl-CoA Synthetase Long Chain Family Member 6 | Protein Coding | Q9UKU0 | 52 | GC05M131949 | 6.37670421600342 | https://www.genecards.org/cgi-bin/carddisp.pl?gene=ACSL6 |  |
| ABO | ABO, Alpha 1-3-N-Acetylgalactosaminyltransferase And Alpha 1-3-Galactosyltransferase | Protein Coding | P16442 | 49 | GC09M133250 | 6.37386655807495 | https://www.genecards.org/cgi-bin/carddisp.pl?gene=ABO |  |
| PLCG1 | Phospholipase C Gamma 1 | Protein Coding | P19174 | 62 | GC20P041136 | 6.34261178970337 | https://www.genecards.org/cgi-bin/carddisp.pl?gene=PLCG1 |  |
| FADD | Fas Associated Via Death Domain | Protein Coding | Q13158 | 60 | GC11P070203 | 6.33859920501709 | https://www.genecards.org/cgi-bin/carddisp.pl?gene=FADD |  |
| TRPV4 | Transient Receptor Potential Cation Channel Subfamily V Member 4 | Protein Coding | Q9HBA0 | 61 | GC12M109783 | 6.33753538131714 | https://www.genecards.org/cgi-bin/carddisp.pl?gene=TRPV4 |  |
| ACTG1 | Actin Gamma 1 | Protein Coding | P63261 | 60 | GC17M081509 | 6.33502626419067 | https://www.genecards.org/cgi-bin/carddisp.pl?gene=ACTG1 |  |
| CNP | 2',3'-Cyclic Nucleotide 3' Phosphodiesterase | Protein Coding | P09543 | 56 | GC17P041966 | 6.32032585144043 | https://www.genecards.org/cgi-bin/carddisp.pl?gene=CNP |  |
| ACSL3 | Acyl-CoA Synthetase Long Chain Family Member 3 | Protein Coding | O95573 | 54 | GC02P222860 | 6.30841445922852 | https://www.genecards.org/cgi-bin/carddisp.pl?gene=ACSL3 |  |
| PISD | Phosphatidylserine Decarboxylase | Protein Coding | Q9UG56 | 57 | GC22M086014 | 6.29156589508057 | https://www.genecards.org/cgi-bin/carddisp.pl?gene=PISD |  |
| CLTC | Clathrin Heavy Chain | Protein Coding | Q00610 | 60 | GC17P059619 | 6.25890684127808 | https://www.genecards.org/cgi-bin/carddisp.pl?gene=CLTC |  |
| SPNS1 | SPNS Lysolipid Transporter 1, Lysophospholipid | Protein Coding | Q9H2V7 | 44 | GC16P028974 | 6.25576400756836 | https://www.genecards.org/cgi-bin/carddisp.pl?gene=SPNS1 |  |
| PRKCB | Protein Kinase C Beta | Protein Coding | P05771 | 62 | GC16P120735 | 6.25536394119263 | https://www.genecards.org/cgi-bin/carddisp.pl?gene=PRKCB |  |
| CALR | Calreticulin | Protein Coding | P27797 | 64 | GC19P012938 | 6.24833488464355 | https://www.genecards.org/cgi-bin/carddisp.pl?gene=CALR |  |
| SPTA1 | Spectrin Alpha, Erythrocytic 1 | Protein Coding | P02549 | 57 | GC01M158610 | 6.24795627593994 | https://www.genecards.org/cgi-bin/carddisp.pl?gene=SPTA1 |  |
| GAD2 | Glutamate Decarboxylase 2 | Protein Coding | Q05329 | 57 | GC10P026216 | 6.24494552612305 | https://www.genecards.org/cgi-bin/carddisp.pl?gene=GAD2 |  |
| APOD | Apolipoprotein D | Protein Coding | P05090 | 55 | GC03M195568 | 6.22780132293701 | https://www.genecards.org/cgi-bin/carddisp.pl?gene=APOD |  |
| VAPA | VAMP Associated Protein A | Protein Coding | Q9P0L0 | 55 | GC18P009938 | 6.22635173797607 | https://www.genecards.org/cgi-bin/carddisp.pl?gene=VAPA |  |
| SPNS3 | SPNS Lysolipid Transporter 3, Sphingosine-1-Phosphate (Putative) | Protein Coding | Q6ZMD2 | 39 | GC17P004433 | 6.21275615692139 | https://www.genecards.org/cgi-bin/carddisp.pl?gene=SPNS3 |  |
| FUT1 | Fucosyltransferase 1 (H Blood Group) | Protein Coding | P19526 | 52 | GC19M048748 | 6.21210670471191 | https://www.genecards.org/cgi-bin/carddisp.pl?gene=FUT1 |  |
| AGRN | Agrin | Protein Coding | O00468 | 58 | GC01P001020 | 6.20103788375854 | https://www.genecards.org/cgi-bin/carddisp.pl?gene=AGRN |  |
| TNFRSF1B | TNF Receptor Superfamily Member 1B | Protein Coding | P20333 | 61 | GC01P075466 | 6.18099021911621 | https://www.genecards.org/cgi-bin/carddisp.pl?gene=TNFRSF1B |  |
| MIRLET7E | MicroRNA Let-7e | RNA Gene |  | 28 | GC19P152977 | 6.16644716262817 | https://www.genecards.org/cgi-bin/carddisp.pl?gene=MIRLET7E |  |
| CD247 | CD247 Molecule | Protein Coding | P20963 | 62 | GC01M167425 | 6.14713191986084 | https://www.genecards.org/cgi-bin/carddisp.pl?gene=CD247 |  |
| SRR | Serine Racemase | Protein Coding | Q9GZT4 | 54 | GC17P002303 | 6.14390277862549 | https://www.genecards.org/cgi-bin/carddisp.pl?gene=SRR |  |
| RBFOX1 | RNA Binding Fox-1 Homolog 1 | Protein Coding | Q9NWB1 | 52 | GC16P120267 | 6.13197660446167 | https://www.genecards.org/cgi-bin/carddisp.pl?gene=RBFOX1 |  |
| LAMP1 | Lysosomal Associated Membrane Protein 1 | Protein Coding | P11279 | 57 | GC13P113297 | 6.12942314147949 | https://www.genecards.org/cgi-bin/carddisp.pl?gene=LAMP1 |  |
| ST3GAL1 | ST3 Beta-Galactoside Alpha-2,3-Sialyltransferase 1 | Protein Coding | Q11201 | 55 | GC08M133454 | 6.12172698974609 | https://www.genecards.org/cgi-bin/carddisp.pl?gene=ST3GAL1 |  |
| COPB2 | COPI Coat Complex Subunit Beta 2 | Protein Coding | P35606 | 55 | GC03M139355 | 6.11441564559937 | https://www.genecards.org/cgi-bin/carddisp.pl?gene=COPB2 |  |
| CASP8 | Caspase 8 | Protein Coding | Q14790 | 65 | GC02P201233 | 6.11072015762329 | https://www.genecards.org/cgi-bin/carddisp.pl?gene=CASP8 |  |
| CDKN3 | Cyclin Dependent Kinase Inhibitor 3 | Protein Coding | Q16667 | 54 | GC14P058243 | 6.10367870330811 | https://www.genecards.org/cgi-bin/carddisp.pl?gene=CDKN3 |  |
| CSNK1G2 | Casein Kinase 1 Gamma 2 | Protein Coding | P78368 | 52 | GC19P001941 | 6.10220766067505 | https://www.genecards.org/cgi-bin/carddisp.pl?gene=CSNK1G2 |  |
| IL13 | Interleukin 13 | Protein Coding | P35225 | 57 | GC05P132656 | 6.09674263000488 | https://www.genecards.org/cgi-bin/carddisp.pl?gene=IL13 |  |
| BACE1 | Beta-Secretase 1 | Protein Coding | P56817 | 60 | GC11M117285 | 6.095871925 | https://www.genecards.org/cgi-bin/carddisp.pl?gene=BACE1 |  |
| PLIN3 | Perilipin 3 | Protein Coding | O60664 | 54 | GC19M107908 | 6.09456777572632 | https://www.genecards.org/cgi-bin/carddisp.pl?gene=PLIN3 |  |
| LYN | LYN Proto-Oncogene, Src Family Tyrosine Kinase | Protein Coding | P07948 | 62 | GC08P055879 | 6.09284067153931 | https://www.genecards.org/cgi-bin/carddisp.pl?gene=LYN |  |
| ABCC3 | ATP Binding Cassette Subfamily C Member 3 | Protein Coding | O15438 | 58 | GC17P050634 | 6.08808994293213 | https://www.genecards.org/cgi-bin/carddisp.pl?gene=ABCC3 |  |
| DGKZ | Diacylglycerol Kinase Zeta | Protein Coding | Q13574 | 56 | GC11P046332 | 6.08445262908936 | https://www.genecards.org/cgi-bin/carddisp.pl?gene=DGKZ |  |
| NCOA3 | Nuclear Receptor Coactivator 3 | Protein Coding | Q9Y6Q9 | 60 | GC20P047501 | 6.07480430603027 | https://www.genecards.org/cgi-bin/carddisp.pl?gene=NCOA3 |  |
| BCAT1 | Branched Chain Amino Acid Transaminase 1 | Protein Coding | P54687 | 57 | GC12M024810 | 6.07239484786987 | https://www.genecards.org/cgi-bin/carddisp.pl?gene=BCAT1 |  |
| MIR221 | MicroRNA 221 | RNA Gene |  | 29 | GC0XM045746 | 6.06597185134888 | https://www.genecards.org/cgi-bin/carddisp.pl?gene=MIR221 |  |
| GBA3 | Glucosylceramidase Beta 3 (Gene/Pseudogene) | Protein Coding | Q9H227 | 44 | GC04P030165 | 6.04411602020264 | https://www.genecards.org/cgi-bin/carddisp.pl?gene=GBA3 |  |
| PNPLA1 | Patatin Like Domain 1, Omega-Hydroxyceramide Transacylase | Protein Coding | Q8N8W4 | 47 | GC06P182015 | 6.02234554290771 | https://www.genecards.org/cgi-bin/carddisp.pl?gene=PNPLA1 |  |
| CCR5 | C-C Motif Chemokine Receptor 5 | Protein Coding | P51681 | 60 | GC03P064850 | 6.01708793640137 | https://www.genecards.org/cgi-bin/carddisp.pl?gene=CCR5 |  |
| GJA1 | Gap Junction Protein Alpha 1 | Protein Coding | P17302 | 64 | GC06P182951 | 5.99256324768066 | https://www.genecards.org/cgi-bin/carddisp.pl?gene=GJA1 |  |
| TRAF6 | TNF Receptor Associated Factor 6 | Protein Coding | Q9Y4K3 | 59 | GC11M036467 | 5.98906755447388 | https://www.genecards.org/cgi-bin/carddisp.pl?gene=TRAF6 |  |
| RB1 | RB Transcriptional Corepressor 1 | Protein Coding | P06400 | 61 | GC13P048303 | 5.98751926422119 | https://www.genecards.org/cgi-bin/carddisp.pl?gene=RB1 |  |
| GNAQ | G Protein Subunit Alpha Q | Protein Coding | P50148 | 61 | GC09M077716 | 5.97889709472656 | https://www.genecards.org/cgi-bin/carddisp.pl?gene=GNAQ |  |
| LGALS3 | Galectin 3 | Protein Coding | P17931 | 57 | GC14P055124 | 5.94248962402344 | https://www.genecards.org/cgi-bin/carddisp.pl?gene=LGALS3 |  |
| ERLIN2 | ER Lipid Raft Associated 2 | Protein Coding | O94905 | 55 | GC08P037736 | 5.93935012817383 | https://www.genecards.org/cgi-bin/carddisp.pl?gene=ERLIN2 |  |
| GLTP | Glycolipid Transfer Protein | Protein Coding | Q9NZD2 | 47 | GC12M109850 | 5.91869640350342 | https://www.genecards.org/cgi-bin/carddisp.pl?gene=GLTP |  |
| RTN4 | Reticulon 4 | Protein Coding | Q9NQC3 | 57 | GC02M054934 | 5.90566635131836 | https://www.genecards.org/cgi-bin/carddisp.pl?gene=RTN4 |  |
| MYH9 | Myosin Heavy Chain 9 | Protein Coding | P35579 | 60 | GC22M036281 | 5.88338136672974 | https://www.genecards.org/cgi-bin/carddisp.pl?gene=MYH9 |  |
| HDAC9 | Histone Deacetylase 9 | Protein Coding | Q9UKV0 | 63 | GC07P018086 | 5.88019895553589 | https://www.genecards.org/cgi-bin/carddisp.pl?gene=HDAC9 |  |
| CXCL10 | C-X-C Motif Chemokine Ligand 10 | Protein Coding | P02778 | 57 | GC04M076021 | 5.87353277206421 | https://www.genecards.org/cgi-bin/carddisp.pl?gene=CXCL10 |  |
| C9orf72 | C9orf72-SMCR8 Complex Subunit | Protein Coding | Q96LT7 | 54 | GC09M030157 | 5.87198114395142 | https://www.genecards.org/cgi-bin/carddisp.pl?gene=C9orf72 |  |
| CAV2 | Caveolin 2 | Protein Coding | P51636 | 55 | GC07P116287 | 5.87024974822998 | https://www.genecards.org/cgi-bin/carddisp.pl?gene=CAV2 |  |
| IL6R | Interleukin 6 Receptor | Protein Coding | P08887 | 63 | GC01P154405 | 5.84269142150879 | https://www.genecards.org/cgi-bin/carddisp.pl?gene=IL6R |  |
| ST6GALNAC6 | ST6 N-Acetylgalactosaminide Alpha-2,6-Sialyltransferase 6 | Protein Coding | Q969X2 | 44 | GC09M131763 | 5.83987045288086 | https://www.genecards.org/cgi-bin/carddisp.pl?gene=ST6GALNAC6 |  |
| LTA | Lymphotoxin Alpha | Protein Coding | P01374 | 55 | GC06P181891 | 5.83169221878052 | https://www.genecards.org/cgi-bin/carddisp.pl?gene=LTA |  |
| TSPAN7 | Tetraspanin 7 | Protein Coding | P41732 | 57 | GC0XP038561 | 5.82282066345215 | https://www.genecards.org/cgi-bin/carddisp.pl?gene=TSPAN7 |  |
| ABCA12 | ATP Binding Cassette Subfamily A Member 12 | Protein Coding | Q86UK0 | 52 | GC02M214931 | 5.81978225708008 | https://www.genecards.org/cgi-bin/carddisp.pl?gene=ABCA12 |  |
| CCN2 | Cellular Communication Network Factor 2 | Protein Coding | P29279 | 58 | GC06M131948 | 5.81038808822632 | https://www.genecards.org/cgi-bin/carddisp.pl?gene=CCN2 |  |
| NEU3 | Neuraminidase 3 | Protein Coding | Q9UQ49 | 50 | GC11P108302 | 5.78081226348877 | https://www.genecards.org/cgi-bin/carddisp.pl?gene=NEU3 |  |
| P2RY12 | Purinergic Receptor P2Y12 | Protein Coding | Q9H244 | 61 | GC03M151336 | 5.77614593505859 | https://www.genecards.org/cgi-bin/carddisp.pl?gene=P2RY12 |  |
| GRM1 | Glutamate Metabotropic Receptor 1 | Protein Coding | Q13255 | 63 | GC06P183496 | 5.76238822937012 | https://www.genecards.org/cgi-bin/carddisp.pl?gene=GRM1 |  |
| KCNA3 | Potassium Voltage-Gated Channel Subfamily A Member 3 | Protein Coding | P22001 | 57 | GC01M110654 | 5.76229238510132 | https://www.genecards.org/cgi-bin/carddisp.pl?gene=KCNA3 |  |
| STARD7 | StAR Related Lipid Transfer Domain Containing 7 | Protein Coding | Q9NQZ5 | 50 | GC02M096184 | 5.76051378250122 | https://www.genecards.org/cgi-bin/carddisp.pl?gene=STARD7 |  |
| KCNE2 | Potassium Voltage-Gated Channel Subfamily E Regulatory Subunit 2 | Protein Coding | Q9Y6J6 | 55 | GC21P034316 | 5.75753498077393 | https://www.genecards.org/cgi-bin/carddisp.pl?gene=KCNE2 |  |
| LOC106627981 | GBA Recombination Region | Functional Element |  | 11 | GC01P155233 | 5.75218439102173 | https://www.genecards.org/cgi-bin/carddisp.pl?gene=LOC106627981 |  |
| NGFR | Nerve Growth Factor Receptor | Protein Coding | P08138 | 60 | GC17P049495 | 5.74795627593994 | https://www.genecards.org/cgi-bin/carddisp.pl?gene=NGFR |  |
| APC | APC Regulator Of WNT Signaling Pathway | Protein Coding | P25054 | 62 | GC05P112707 | 5.74291944503784 | https://www.genecards.org/cgi-bin/carddisp.pl?gene=APC |  |
| HFE-AS1 | HFE Antisense RNA 1 | RNA Gene |  | 15 | GC06M106104 | 5.74051189422607 | https://www.genecards.org/cgi-bin/carddisp.pl?gene=HFE-AS1 |  |
| FFAR1 | Free Fatty Acid Receptor 1 | Protein Coding | O14842 | 51 | GC19P153609 | 5.73320770263672 | https://www.genecards.org/cgi-bin/carddisp.pl?gene=FFAR1 |  |
| GABBR1 | Gamma-Aminobutyric Acid Type B Receptor Subunit 1 | Protein Coding | Q9UBS5 | 61 | GC06M029555 | 5.72974395751953 | https://www.genecards.org/cgi-bin/carddisp.pl?gene=GABBR1 |  |
| GSTA2 | Glutathione S-Transferase Alpha 2 | Protein Coding | P09210 | 48 | GC06M052750 | 5.72331190109253 | https://www.genecards.org/cgi-bin/carddisp.pl?gene=GSTA2 |  |
| CNR2 | Cannabinoid Receptor 2 | Protein Coding | P34972 | 57 | GC01M023870 | 5.71614265441895 | https://www.genecards.org/cgi-bin/carddisp.pl?gene=CNR2 |  |
| HULC | Hepatocellular Carcinoma Up-Regulated Long Non-Coding RNA | RNA Gene |  | 29 | GC06P009612 | 5.70324897766113 | https://www.genecards.org/cgi-bin/carddisp.pl?gene=HULC |  |
| VAC14 | VAC14 Component Of PIKFYVE Complex | Protein Coding | Q08AM6 | 55 | GC16M075143 | 5.70087051391602 | https://www.genecards.org/cgi-bin/carddisp.pl?gene=VAC14 |  |
| KDR | Kinase Insert Domain Receptor | Protein Coding | P35968 | 67 | GC04M055078 | 5.68371963500977 | https://www.genecards.org/cgi-bin/carddisp.pl?gene=KDR |  |
| MIR95 | MicroRNA 95 | RNA Gene |  | 27 | GC04M008029 | 5.68184185028076 | https://www.genecards.org/cgi-bin/carddisp.pl?gene=MIR95 |  |
| E2F1 | E2F Transcription Factor 1 | Protein Coding | Q01094 | 57 | GC20M033675 | 5.67510795593262 | https://www.genecards.org/cgi-bin/carddisp.pl?gene=E2F1 |  |
| EIF2S1 | Eukaryotic Translation Initiation Factor 2 Subunit Alpha | Protein Coding | P05198 | 58 | GC14P067359 | 5.67418670654297 | https://www.genecards.org/cgi-bin/carddisp.pl?gene=EIF2S1 |  |
| AP2S1 | Adaptor Related Protein Complex 2 Subunit Sigma 1 | Protein Coding | P53680 | 55 | GC19M046838 | 5.67263984680176 | https://www.genecards.org/cgi-bin/carddisp.pl?gene=AP2S1 |  |
| ECE1 | Endothelin Converting Enzyme 1 | Protein Coding | P42892 | 61 | GC01M021217 | 5.66693162918091 | https://www.genecards.org/cgi-bin/carddisp.pl?gene=ECE1 |  |
| HSPA1A | Heat Shock Protein Family A (Hsp70) Member 1A | Protein Coding | P0DMV8 | 60 | GC06P181910 | 5.64666986465454 | https://www.genecards.org/cgi-bin/carddisp.pl?gene=HSPA1A |  |
| ANXA5 | Annexin A5 | Protein Coding | P08758 | 60 | GC04M121667 | 5.64466524124146 | https://www.genecards.org/cgi-bin/carddisp.pl?gene=ANXA5 |  |
| OPRM1 | Opioid Receptor Mu 1 | Protein Coding | P35372 | 61 | GC06P183705 | 5.63039112091064 | https://www.genecards.org/cgi-bin/carddisp.pl?gene=OPRM1 |  |
| MSX2 | Msh Homeobox 2 | Protein Coding | P35548 | 57 | GC05P174724 | 5.62617492675781 | https://www.genecards.org/cgi-bin/carddisp.pl?gene=MSX2 |  |
| HLA-A | Major Histocompatibility Complex, Class I, A | Protein Coding | P04439 | 59 | GC06P181854 | 5.60854387283325 | https://www.genecards.org/cgi-bin/carddisp.pl?gene=HLA-A |  |
| CASP9 | Caspase 9 | Protein Coding | P55211 | 61 | GC01M022575 | 5.60229110717773 | https://www.genecards.org/cgi-bin/carddisp.pl?gene=CASP9 |  |
| AP2M1 | Adaptor Related Protein Complex 2 Subunit Mu 1 | Protein Coding | Q96CW1 | 60 | GC03P184174 | 5.58455657958984 | https://www.genecards.org/cgi-bin/carddisp.pl?gene=AP2M1 |  |
| DNM3 | Dynamin 3 | Protein Coding | Q9UQ16 | 56 | GC01P177364 | 5.58363771438599 | https://www.genecards.org/cgi-bin/carddisp.pl?gene=DNM3 |  |
| TRPV6 | Transient Receptor Potential Cation Channel Subfamily V Member 6 | Protein Coding | Q9H1D0 | 60 | GC07M142871 | 5.58277940750122 | https://www.genecards.org/cgi-bin/carddisp.pl?gene=TRPV6 |  |
| CD59 | CD59 Molecule (CD59 Blood Group) | Protein Coding | P13987 | 59 | GC11M033737 | 5.58068609237671 | https://www.genecards.org/cgi-bin/carddisp.pl?gene=CD59 |  |
| HTR3A | 5-Hydroxytryptamine Receptor 3A | Protein Coding | P46098 | 58 | GC11P113975 | 5.55643033981323 | https://www.genecards.org/cgi-bin/carddisp.pl?gene=HTR3A |  |
| CDKN1A | Cyclin Dependent Kinase Inhibitor 1A | Protein Coding | P38936 | 63 | GC06P182020 | 5.54633140563965 | https://www.genecards.org/cgi-bin/carddisp.pl?gene=CDKN1A |  |
| MBP | Myelin Basic Protein | Protein Coding | P02686 | 58 | GC18M076978 | 5.53693866729736 | https://www.genecards.org/cgi-bin/carddisp.pl?gene=MBP |  |
| NAAA | N-Acylethanolamine Acid Amidase | Protein Coding | Q02083 | 56 | GC04M075910 | 5.51849412918091 | https://www.genecards.org/cgi-bin/carddisp.pl?gene=NAAA |  |
| DHRS9 | Dehydrogenase/Reductase 9 | Protein Coding | Q9BPW9 | 51 | GC02P169064 | 5.48943901062012 | https://www.genecards.org/cgi-bin/carddisp.pl?gene=DHRS9 |  |
| CSF3 | Colony Stimulating Factor 3 | Protein Coding | P09919 | 55 | GC17P040015 | 5.48688507080078 | https://www.genecards.org/cgi-bin/carddisp.pl?gene=CSF3 |  |
| AGGF1 | Angiogenic Factor With G-Patch And FHA Domains 1 | Protein Coding | Q8N302 | 52 | GC05P077029 | 5.47834491729736 | https://www.genecards.org/cgi-bin/carddisp.pl?gene=AGGF1 |  |
| ADRB1 | Adrenoceptor Beta 1 | Protein Coding | P08588 | 62 | GC10P125703 | 5.47323513031006 | https://www.genecards.org/cgi-bin/carddisp.pl?gene=ADRB1 |  |
| KCNA5 | Potassium Voltage-Gated Channel Subfamily A Member 5 | Protein Coding | P22460 | 58 | GC12P005043 | 5.46339702606201 | https://www.genecards.org/cgi-bin/carddisp.pl?gene=KCNA5 |  |
| COPB1 | COPI Coat Complex Subunit Beta 1 | Protein Coding | P53618 | 52 | GC11M015482 | 5.44600200653076 | https://www.genecards.org/cgi-bin/carddisp.pl?gene=COPB1 |  |
| ITGB3 | Integrin Subunit Beta 3 | Protein Coding | P05106 | 65 | GC17P155043 | 5.41993141174316 | https://www.genecards.org/cgi-bin/carddisp.pl?gene=ITGB3 |  |
| NEU2 | Neuraminidase 2 | Protein Coding | Q9Y3R4 | 53 | GC02P233032 | 5.41748714447021 | https://www.genecards.org/cgi-bin/carddisp.pl?gene=NEU2 |  |
| IL3 | Interleukin 3 | Protein Coding | P08700 | 57 | GC05P132060 | 5.41714382171631 | https://www.genecards.org/cgi-bin/carddisp.pl?gene=IL3 |  |
| CSF2 | Colony Stimulating Factor 2 | Protein Coding | P04141 | 58 | GC05P132073 | 5.37729072570801 | https://www.genecards.org/cgi-bin/carddisp.pl?gene=CSF2 |  |
| DRD1 | Dopamine Receptor D1 | Protein Coding | P21728 | 58 | GC05M175440 | 5.37626314163208 | https://www.genecards.org/cgi-bin/carddisp.pl?gene=DRD1 |  |
| ACSBG1 | Acyl-CoA Synthetase Bubblegum Family Member 1 | Protein Coding | Q96GR2 | 51 | GC15M078167 | 5.3701000213623 | https://www.genecards.org/cgi-bin/carddisp.pl?gene=ACSBG1 |  |
| ITGB2 | Integrin Subunit Beta 2 | Protein Coding | P05107 | 63 | GC21M044885 | 5.36868906021118 | https://www.genecards.org/cgi-bin/carddisp.pl?gene=ITGB2 |  |
| NR2F1 | Nuclear Receptor Subfamily 2 Group F Member 1 | Protein Coding | P10589 | 57 | GC05P093583 | 5.36473369598389 | https://www.genecards.org/cgi-bin/carddisp.pl?gene=NR2F1 |  |
| THRSP | Thyroid Hormone Responsive | Protein Coding | Q92748 | 45 | GC11P078063 | 5.32992267608643 | https://www.genecards.org/cgi-bin/carddisp.pl?gene=THRSP |  |
| PAK1 | P21 (RAC1) Activated Kinase 1 | Protein Coding | Q13153 | 64 | GC11M140142 | 5.32787275314331 | https://www.genecards.org/cgi-bin/carddisp.pl?gene=PAK1 |  |
| CCL5 | C-C Motif Chemokine Ligand 5 | Protein Coding | P13501 | 56 | GC17M035871 | 5.31235599517822 | https://www.genecards.org/cgi-bin/carddisp.pl?gene=CCL5 |  |
| ARSD | Arylsulfatase D | Protein Coding | P51689 | 48 | GC0XM002903 | 5.30536413192749 | https://www.genecards.org/cgi-bin/carddisp.pl?gene=ARSD |  |
| ELAVL1 | ELAV Like RNA Binding Protein 1 | Protein Coding | Q15717 | 54 | GC19M007958 | 5.30440330505371 | https://www.genecards.org/cgi-bin/carddisp.pl?gene=ELAVL1 |  |
| KRIT1 | KRIT1 Ankyrin Repeat Containing | Protein Coding | O00522 | 54 | GC07M092198 | 5.29054737091064 | https://www.genecards.org/cgi-bin/carddisp.pl?gene=KRIT1 |  |
| DGKQ | Diacylglycerol Kinase Theta | Protein Coding | P52824 | 54 | GC04M000958 | 5.2871150970459 | https://www.genecards.org/cgi-bin/carddisp.pl?gene=DGKQ |  |
| OGA | O-GlcNAcase | Protein Coding | O60502 | 53 | GC10M101785 | 5.27763414382935 | https://www.genecards.org/cgi-bin/carddisp.pl?gene=OGA |  |
| DGKB | Diacylglycerol Kinase Beta | Protein Coding | Q9Y6T7 | 52 | GC07M014145 | 5.2670955657959 | https://www.genecards.org/cgi-bin/carddisp.pl?gene=DGKB |  |
| PAPSS1 | 3'-Phosphoadenosine 5'-Phosphosulfate Synthase 1 | Protein Coding | O43252 | 52 | GC04M107590 | 5.26068067550659 | https://www.genecards.org/cgi-bin/carddisp.pl?gene=PAPSS1 |  |
| SDS | Serine Dehydratase | Protein Coding | P20132 | 51 | GC12M113392 | 5.23843669891357 | https://www.genecards.org/cgi-bin/carddisp.pl?gene=SDS |  |
| LOC129936056 | ATAC-STARR-Seq Lymphoblastoid Silent Region 14016 | Functional Element |  | 7 | GC03P004489 | 5.23109865188599 | https://www.genecards.org/cgi-bin/carddisp.pl?gene=LOC129936056 |  |
| IL23R | Interleukin 23 Receptor | Protein Coding | Q5VWK5 | 57 | GC01P067138 | 5.22144460678101 | https://www.genecards.org/cgi-bin/carddisp.pl?gene=IL23R |  |
| RARA | Retinoic Acid Receptor Alpha | Protein Coding | P10276 | 62 | GC17P040309 | 5.21922492980957 | https://www.genecards.org/cgi-bin/carddisp.pl?gene=RARA |  |
| H3-3A | H3.3 Histone A | Protein Coding | P84243 | 57 | GC01P226062 | 5.2093710899353 | https://www.genecards.org/cgi-bin/carddisp.pl?gene=H3-3A |  |
| ITIH4 | Inter-Alpha-Trypsin Inhibitor Heavy Chain 4 | Protein Coding | Q14624 | 55 | GC03M052812 | 5.20194387435913 | https://www.genecards.org/cgi-bin/carddisp.pl?gene=ITIH4 |  |
| AADAT | Aminoadipate Aminotransferase | Protein Coding | Q8N5Z0 | 52 | GC04M170060 | 5.19202899932861 | https://www.genecards.org/cgi-bin/carddisp.pl?gene=AADAT |  |
| SP3 | Sp3 Transcription Factor | Protein Coding | Q02447 | 55 | GC02M173882 | 5.1858491897583 | https://www.genecards.org/cgi-bin/carddisp.pl?gene=SP3 |  |
| VPS35 | VPS35 Retromer Complex Component | Protein Coding | Q96QK1 | 55 | GC16M051451 | 5.17011022567749 | https://www.genecards.org/cgi-bin/carddisp.pl?gene=VPS35 |  |
| ENPP2 | Ectonucleotide Pyrophosphatase/Phosphodiesterase 2 | Protein Coding | Q13822 | 58 | GC08M119556 | 5.16152715682983 | https://www.genecards.org/cgi-bin/carddisp.pl?gene=ENPP2 |  |
| GNAO1 | G Protein Subunit Alpha O1 | Protein Coding | P09471 | 60 | GC16P121581 | 5.16115140914917 | https://www.genecards.org/cgi-bin/carddisp.pl?gene=GNAO1 |  |
| CDK1 | Cyclin Dependent Kinase 1 | Protein Coding | P06493 | 60 | GC10P060772 | 5.15730714797974 | https://www.genecards.org/cgi-bin/carddisp.pl?gene=CDK1 |  |
| ULK1 | Unc-51 Like Autophagy Activating Kinase 1 | Protein Coding | O75385 | 57 | GC12P131894 | 5.13646364212036 | https://www.genecards.org/cgi-bin/carddisp.pl?gene=ULK1 |  |
| PLEKHA8 | Pleckstrin Homology Domain Containing A8 | Protein Coding | Q96JA3 | 48 | GC07P030027 | 5.12525081634521 | https://www.genecards.org/cgi-bin/carddisp.pl?gene=PLEKHA8 |  |
| ALAS1 | 5'-Aminolevulinate Synthase 1 | Protein Coding | P13196 | 55 | GC03P052198 | 5.1169319152832 | https://www.genecards.org/cgi-bin/carddisp.pl?gene=ALAS1 |  |
| PDXDC1 | Pyridoxal Dependent Decarboxylase Domain Containing 1 | Protein Coding | Q6P996 | 48 | GC16P014974 | 5.10591793060303 | https://www.genecards.org/cgi-bin/carddisp.pl?gene=PDXDC1 |  |
| LMAN1 | Lectin, Mannose Binding 1 | Protein Coding | P49257 | 58 | GC18M059327 | 5.09252071380615 | https://www.genecards.org/cgi-bin/carddisp.pl?gene=LMAN1 |  |
| YWHAB | Tyrosine 3-Monooxygenase/Tryptophan 5-Monooxygenase Activation Protein Beta | Protein Coding | P31946 | 60 | GC20P044885 | 5.08901405334473 | https://www.genecards.org/cgi-bin/carddisp.pl?gene=YWHAB |  |
| MAG | Myelin Associated Glycoprotein | Protein Coding | P20916 | 60 | GC19P035292 | 5.08289909362793 | https://www.genecards.org/cgi-bin/carddisp.pl?gene=MAG |  |
| PSAPL1 | Prosaposin Like 1 | Protein Coding | Q6NUJ1 | 44 | GC04M007434 | 5.06900310516357 | https://www.genecards.org/cgi-bin/carddisp.pl?gene=PSAPL1 |  |
| ALDH3B2 | Aldehyde Dehydrogenase 3 Family Member B2 | Protein Coding | P48448 | 50 | GC11M067662 | 5.06871604919434 | https://www.genecards.org/cgi-bin/carddisp.pl?gene=ALDH3B2 |  |
| CLU | Clusterin | Protein Coding | P10909 | 60 | GC08M027596 | 5.06704616546631 | https://www.genecards.org/cgi-bin/carddisp.pl?gene=CLU |  |
| NEU4 | Neuraminidase 4 | Protein Coding | Q8WWR8 | 47 | GC02P242392 | 5.03485822677612 | https://www.genecards.org/cgi-bin/carddisp.pl?gene=NEU4 |  |
| CEBPB | CCAAT Enhancer Binding Protein Beta | Protein Coding | P17676 | 58 | GC20P050190 | 5.03252077102661 | https://www.genecards.org/cgi-bin/carddisp.pl?gene=CEBPB |  |
| PKD2 | Polycystin 2, Transient Receptor Potential Cation Channel | Protein Coding | Q13563 | 61 | GC04P088007 | 5.031813622 | https://www.genecards.org/cgi-bin/carddisp.pl?gene=PKD2 |  |
| ESRRB | Estrogen Related Receptor Beta | Protein Coding | O95718 | 61 | GC14P076310 | 5.03150939941406 | https://www.genecards.org/cgi-bin/carddisp.pl?gene=ESRRB |  |
| IL17F | Interleukin 17F | Protein Coding | Q96PD4 | 54 | GC06M106847 | 5.03103065490723 | https://www.genecards.org/cgi-bin/carddisp.pl?gene=IL17F |  |
| HLMR1 | Hepatic LncRNA Metabolic Regulator 1 | RNA Gene |  | 12 | GC03P143422 | 5.02753829956055 | https://www.genecards.org/cgi-bin/carddisp.pl?gene=HLMR1 |  |
| CASP1 | Caspase 1 | Protein Coding | P29466 | 61 | GC11M105025 | 5.02345371246338 | https://www.genecards.org/cgi-bin/carddisp.pl?gene=CASP1 |  |
| ST8SIA5 | ST8 Alpha-N-Acetyl-Neuraminide Alpha-2,8-Sialyltransferase 5 | Protein Coding | O15466 | 44 | GC18M046667 | 5.01013565063477 | https://www.genecards.org/cgi-bin/carddisp.pl?gene=ST8SIA5 |  |
| EPAS1 | Endothelial PAS Domain Protein 1 | Protein Coding | Q99814 | 64 | GC02P046293 | 5.00514650344849 | https://www.genecards.org/cgi-bin/carddisp.pl?gene=EPAS1 |  |
| S1PR5 | Sphingosine-1-Phosphate Receptor 5 | Protein Coding | Q9H228 | 55 | GC19M010512 | 5.00513410568237 | https://www.genecards.org/cgi-bin/carddisp.pl?gene=S1PR5 |  |
| CNGA1 | Cyclic Nucleotide Gated Channel Subunit Alpha 1 | Protein Coding | P29973 | 57 | GC04M047935 | 4.9871301651001 | https://www.genecards.org/cgi-bin/carddisp.pl?gene=CNGA1 |  |
| IL1R1 | Interleukin 1 Receptor Type 1 | Protein Coding | P14778 | 60 | GC02P102136 | 4.98544216156006 | https://www.genecards.org/cgi-bin/carddisp.pl?gene=IL1R1 |  |
| HDAC2 | Histone Deacetylase 2 | Protein Coding | Q92769 | 65 | GC06M113933 | 4.98536348342896 | https://www.genecards.org/cgi-bin/carddisp.pl?gene=HDAC2 |  |
| CDH1 | Cadherin 1 | Protein Coding | P12830 | 62 | GC16P068737 | 4.98049545288086 | https://www.genecards.org/cgi-bin/carddisp.pl?gene=CDH1 |  |
| FURIN | Furin, Paired Basic Amino Acid Cleaving Enzyme | Protein Coding | P09958 | 61 | GC15P090868 | 4.94292402267456 | https://www.genecards.org/cgi-bin/carddisp.pl?gene=FURIN |  |
| CCR1 | C-C Motif Chemokine Receptor 1 | Protein Coding | P32246 | 57 | GC03M046218 | 4.9397873878479 | https://www.genecards.org/cgi-bin/carddisp.pl?gene=CCR1 |  |
| TMEM258 | Transmembrane Protein 258 | Protein Coding | P61165 | 42 | GC11M061768 | 4.93439149856567 | https://www.genecards.org/cgi-bin/carddisp.pl?gene=TMEM258 |  |
| HACD3 | 3-Hydroxyacyl-CoA Dehydratase 3 | Protein Coding | Q9P035 | 43 | GC15P065530 | 4.93359041213989 | https://www.genecards.org/cgi-bin/carddisp.pl?gene=HACD3 |  |
| FAM20C | FAM20C Golgi Associated Secretory Pathway Kinase | Protein Coding | Q8IXL6 | 55 | GC07P000192 | 4.91840887069702 | https://www.genecards.org/cgi-bin/carddisp.pl?gene=FAM20C |  |
| NKX2-1 | NK2 Homeobox 1 | Protein Coding | P43699 | 61 | GC14M036516 | 4.91137361526489 | https://www.genecards.org/cgi-bin/carddisp.pl?gene=NKX2-1 |  |
| KCNV2 | Potassium Voltage-Gated Channel Modifier Subfamily V Member 2 | Protein Coding | Q8TDN2 | 54 | GC09P002717 | 4.91058111190796 | https://www.genecards.org/cgi-bin/carddisp.pl?gene=KCNV2 |  |
| RICTOR | RPTOR Independent Companion Of MTOR Complex 2 | Protein Coding | Q6R327 | 56 | GC05M038937 | 4.90583610534668 | https://www.genecards.org/cgi-bin/carddisp.pl?gene=RICTOR |  |
| FASLG | Fas Ligand | Protein Coding | P48023 | 61 | GC01P172659 | 4.90250682830811 | https://www.genecards.org/cgi-bin/carddisp.pl?gene=FASLG |  |
| ITGB1 | Integrin Subunit Beta 1 | Protein Coding | P05556 | 62 | GC10M037195 | 4.89477300643921 | https://www.genecards.org/cgi-bin/carddisp.pl?gene=ITGB1 |  |
| FAS-AS1 | FAS Antisense RNA 1 | RNA Gene |  | 21 | GC10M088991 | 4.8685188293457 | https://www.genecards.org/cgi-bin/carddisp.pl?gene=FAS-AS1 |  |
| MIR320A | MicroRNA 320a | RNA Gene |  | 31 | GC08M022455 | 4.86481332778931 | https://www.genecards.org/cgi-bin/carddisp.pl?gene=MIR320A |  |
| FLT3 | Fms Related Receptor Tyrosine Kinase 3 | Protein Coding | P36888 | 66 | GC13M028003 | 4.85240077972412 | https://www.genecards.org/cgi-bin/carddisp.pl?gene=FLT3 |  |
| VIP | Vasoactive Intestinal Peptide | Protein Coding | P01282 | 57 | GC06P152750 | 4.82796573638916 | https://www.genecards.org/cgi-bin/carddisp.pl?gene=VIP |  |
| DYNC2LI1 | Dynein Cytoplasmic 2 Light Intermediate Chain 1 | Protein Coding | Q8TCX1 | 51 | GC02P045313 | 4.82618856430054 | https://www.genecards.org/cgi-bin/carddisp.pl?gene=DYNC2LI1 |  |
| KCNE3 | Potassium Voltage-Gated Channel Subfamily E Regulatory Subunit 3 | Protein Coding | Q9Y6H6 | 57 | GC11M074454 | 4.82568407058716 | https://www.genecards.org/cgi-bin/carddisp.pl?gene=KCNE3 |  |
| KLF4 | KLF Transcription Factor 4 | Protein Coding | O43474 | 60 | GC09M107484 | 4.82441997528076 | https://www.genecards.org/cgi-bin/carddisp.pl?gene=KLF4 |  |
| FUT9 | Fucosyltransferase 9 | Protein Coding | Q9Y231 | 52 | GC06P096015 | 4.82338714599609 | https://www.genecards.org/cgi-bin/carddisp.pl?gene=FUT9 |  |
| PRKCD | Protein Kinase C Delta | Protein Coding | Q05655 | 65 | GC03P053156 | 4.81065368652344 | https://www.genecards.org/cgi-bin/carddisp.pl?gene=PRKCD |  |
| VDAC2 | Voltage Dependent Anion Channel 2 | Protein Coding | P45880 | 56 | GC10P075210 | 4.80665302276611 | https://www.genecards.org/cgi-bin/carddisp.pl?gene=VDAC2 |  |
| BCL2L1 | BCL2 Like 1 | Protein Coding | Q07817 | 62 | GC20M031664 | 4.80233907699585 | https://www.genecards.org/cgi-bin/carddisp.pl?gene=BCL2L1 |  |
| VIM | Vimentin | Protein Coding | P08670 | 64 | GC10P017227 | 4.7965784072876 | https://www.genecards.org/cgi-bin/carddisp.pl?gene=VIM |  |
| PPM1L | Protein Phosphatase, Mg2+/Mn2+ Dependent 1L | Protein Coding | Q5SGD2 | 47 | GC03P160755 | 4.76394891738892 | https://www.genecards.org/cgi-bin/carddisp.pl?gene=PPM1L |  |
| PTK2B | Protein Tyrosine Kinase 2 Beta | Protein Coding | Q14289 | 62 | GC08P027311 | 4.75787544250488 | https://www.genecards.org/cgi-bin/carddisp.pl?gene=PTK2B |  |
| ARSI | Arylsulfatase Family Member I | Protein Coding | Q5FYB1 | 47 | GC05M150296 | 4.75452423095703 | https://www.genecards.org/cgi-bin/carddisp.pl?gene=ARSI |  |
| TACR1 | Tachykinin Receptor 1 | Protein Coding | P25103 | 59 | GC02M075860 | 4.75323724746704 | https://www.genecards.org/cgi-bin/carddisp.pl?gene=TACR1 |  |
| PDPK1 | 3-Phosphoinositide Dependent Protein Kinase 1 | Protein Coding | O15530 | 61 | GC16P002537 | 4.75170135498047 | https://www.genecards.org/cgi-bin/carddisp.pl?gene=PDPK1 |  |
| ANXA2 | Annexin A2 | Protein Coding | P07355 | 60 | GC15M060347 | 4.74954414367676 | https://www.genecards.org/cgi-bin/carddisp.pl?gene=ANXA2 |  |
| CLCN7 | Chloride Voltage-Gated Channel 7 | Protein Coding | P51798 | 58 | GC16M001444 | 4.74919462203979 | https://www.genecards.org/cgi-bin/carddisp.pl?gene=CLCN7 |  |
| NEDD4 | NEDD4 E3 Ubiquitin Protein Ligase | Protein Coding | P46934 | 60 | GC15M055826 | 4.73894453048706 | https://www.genecards.org/cgi-bin/carddisp.pl?gene=NEDD4 |  |
| CHPT1 | Choline Phosphotransferase 1 | Protein Coding | Q8WUD6 | 48 | GC12P101696 | 4.72379732131958 | https://www.genecards.org/cgi-bin/carddisp.pl?gene=CHPT1 |  |
| PHB2 | Prohibitin 2 | Protein Coding | Q99623 | 56 | GC12M006965 | 4.72019386291504 | https://www.genecards.org/cgi-bin/carddisp.pl?gene=PHB2 |  |
| PRKD2 | Protein Kinase D2 | Protein Coding | Q9BZL6 | 57 | GC19M046674 | 4.71472263336182 | https://www.genecards.org/cgi-bin/carddisp.pl?gene=PRKD2 |  |
| BRCC3 | BRCA1/BRCA2-Containing Complex Subunit 3 | Protein Coding | P46736 | 55 | GC0XP155071 | 4.7131028175354 | https://www.genecards.org/cgi-bin/carddisp.pl?gene=BRCC3 |  |
| DPEP1 | Dipeptidase 1 | Protein Coding | P16444 | 57 | GC16P089613 | 4.69410228729248 | https://www.genecards.org/cgi-bin/carddisp.pl?gene=DPEP1 |  |
| ACTA2 | Actin Alpha 2, Smooth Muscle | Protein Coding | P62736 | 61 | GC10M088935 | 4.68551301956177 | https://www.genecards.org/cgi-bin/carddisp.pl?gene=ACTA2 |  |
| ITGAX | Integrin Subunit Alpha X | Protein Coding | P20702 | 59 | GC16P121102 | 4.67900371551514 | https://www.genecards.org/cgi-bin/carddisp.pl?gene=ITGAX |  |
| RIPK1 | Receptor Interacting Serine/Threonine Kinase 1 | Protein Coding | Q13546 | 64 | GC06P009353 | 4.67620086669922 | https://www.genecards.org/cgi-bin/carddisp.pl?gene=RIPK1 |  |
| NOD2 | Nucleotide Binding Oligomerization Domain Containing 2 | Protein Coding | Q9HC29 | 61 | GC16P050693 | 4.66657161712646 | https://www.genecards.org/cgi-bin/carddisp.pl?gene=NOD2 |  |
| PTDSS1 | Phosphatidylserine Synthase 1 | Protein Coding | P48651 | 56 | GC08P096261 | 4.66653823852539 | https://www.genecards.org/cgi-bin/carddisp.pl?gene=PTDSS1 |  |
| RAB14 | RAB14, Member RAS Oncogene Family | Protein Coding | P61106 | 50 | GC09M121178 | 4.66380596160889 | https://www.genecards.org/cgi-bin/carddisp.pl?gene=RAB14 |  |
| SHC1 | SHC Adaptor Protein 1 | Protein Coding | P29353 | 57 | GC01M154962 | 4.6503119468689 | https://www.genecards.org/cgi-bin/carddisp.pl?gene=SHC1 |  |
| SERINC5 | Serine Incorporator 5 | Protein Coding | Q86VE9 | 45 | GC05M080111 | 4.6496376991272 | https://www.genecards.org/cgi-bin/carddisp.pl?gene=SERINC5 |  |
| ABHD3 | Abhydrolase Domain Containing 3, Phospholipase | Protein Coding | Q8WU67 | 46 | GC18M021650 | 4.63767099380493 | https://www.genecards.org/cgi-bin/carddisp.pl?gene=ABHD3 |  |
| ADIPOQ-AS1 | ADIPOQ Antisense RNA 1 | RNA Gene |  | 18 | GC03M186851 | 4.61245203018188 | https://www.genecards.org/cgi-bin/carddisp.pl?gene=ADIPOQ-AS1 |  |
| LOC106660625 | Adiponectin Enhancer Region | Functional Element |  | 4 | GC03P188261 | 4.56469821929932 | https://www.genecards.org/cgi-bin/carddisp.pl?gene=LOC106660625 |  |
| PLD2 | Phospholipase D2 | Protein Coding | O14939 | 58 | GC17P153629 | 4.55140113830566 | https://www.genecards.org/cgi-bin/carddisp.pl?gene=PLD2 |  |
| PRKD3 | Protein Kinase D3 | Protein Coding | O94806 | 57 | GC02M037251 | 4.537278175 | https://www.genecards.org/cgi-bin/carddisp.pl?gene=PRKD3 |  |
| HLA-DQA1 | Major Histocompatibility Complex, Class II, DQ Alpha 1 | Protein Coding | P01909 | 55 | GC06P181921 | 4.53433275222778 | https://www.genecards.org/cgi-bin/carddisp.pl?gene=HLA-DQA1 |  |
| HACD2 | 3-Hydroxyacyl-CoA Dehydratase 2 | Protein Coding | Q6Y1H2 | 42 | GC03M123490 | 4.52270078659058 | https://www.genecards.org/cgi-bin/carddisp.pl?gene=HACD2 |  |
| ST6GALNAC5 | ST6 N-Acetylgalactosaminide Alpha-2,6-Sialyltransferase 5 | Protein Coding | Q9BVH7 | 44 | GC01P076867 | 4.51771259307861 | https://www.genecards.org/cgi-bin/carddisp.pl?gene=ST6GALNAC5 |  |
| IL5 | Interleukin 5 | Protein Coding | P05113 | 58 | GC05M132541 | 4.47998571395874 | https://www.genecards.org/cgi-bin/carddisp.pl?gene=IL5 |  |
| GOLM1 | Golgi Membrane Protein 1 | Protein Coding | Q8NBJ4 | 52 | GC09M086026 | 4.47623682022095 | https://www.genecards.org/cgi-bin/carddisp.pl?gene=GOLM1 |  |
| ZDHHC9 | Zinc Finger DHHC-Type Palmitoyltransferase 9 | Protein Coding | Q9Y397 | 54 | GC0XM129803 | 4.4630069732666 | https://www.genecards.org/cgi-bin/carddisp.pl?gene=ZDHHC9 |  |
| APOC4 | Apolipoprotein C4 | Protein Coding | P55056 | 45 | GC19P152700 | 4.46288251876831 | https://www.genecards.org/cgi-bin/carddisp.pl?gene=APOC4 |  |
| CLIP1 | CAP-Gly Domain Containing Linker Protein 1 | Protein Coding | P30622 | 57 | GC12M122271 | 4.4601879119873 | https://www.genecards.org/cgi-bin/carddisp.pl?gene=CLIP1 |  |
| STAP1 | Signal Transducing Adaptor Family Member 1 | Protein Coding | Q9ULZ2 | 50 | GC04P067558 | 4.45248031616211 | https://www.genecards.org/cgi-bin/carddisp.pl?gene=STAP1 |  |
| STARD3 | StAR Related Lipid Transfer Domain Containing 3 | Protein Coding | Q14849 | 51 | GC17P039637 | 4.44469213485718 | https://www.genecards.org/cgi-bin/carddisp.pl?gene=STARD3 |  |
| MIR494 | MicroRNA 494 | RNA Gene |  | 26 | GC14P121384 | 4.44278717041016 | https://www.genecards.org/cgi-bin/carddisp.pl?gene=MIR494 |  |
| ATP10D | ATPase Phospholipid Transporting 10D (Putative) | Protein Coding | Q9P241 | 48 | GC04P047549 | 4.44083976745605 | https://www.genecards.org/cgi-bin/carddisp.pl?gene=ATP10D |  |
| CD79A | CD79a Molecule | Protein Coding | P11912 | 61 | GC19P041877 | 4.4378662109375 | https://www.genecards.org/cgi-bin/carddisp.pl?gene=CD79A |  |
| RAB5A | RAB5A, Member RAS Oncogene Family | Protein Coding | P20339 | 58 | GC03P027238 | 4.43312454223633 | https://www.genecards.org/cgi-bin/carddisp.pl?gene=RAB5A |  |
| NDN | Necdin, MAGE Family Member | Protein Coding | Q99608 | 55 | GC15M047478 | 4.42848300933838 | https://www.genecards.org/cgi-bin/carddisp.pl?gene=NDN |  |
| SIGMAR1 | Sigma Non-Opioid Intracellular Receptor 1 | Protein Coding | Q99720 | 59 | GC09M034634 | 4.42445755004883 | https://www.genecards.org/cgi-bin/carddisp.pl?gene=SIGMAR1 |  |
| RAB6A | RAB6A, Member RAS Oncogene Family | Protein Coding | P20340 | 53 | GC11M140017 | 4.41862773895264 | https://www.genecards.org/cgi-bin/carddisp.pl?gene=RAB6A |  |
| COPA | COPI Coat Complex Subunit Alpha | Protein Coding | P53621 | 55 | GC01M160288 | 4.41571426391602 | https://www.genecards.org/cgi-bin/carddisp.pl?gene=COPA |  |
| MAT2B | Methionine Adenosyltransferase 2 Non-Catalytic Beta Subunit | Protein Coding | Q9NZL9 | 51 | GC05P163504 | 4.41433572769165 | https://www.genecards.org/cgi-bin/carddisp.pl?gene=MAT2B |  |
| ST6GAL1 | ST6 Beta-Galactoside Alpha-2,6-Sialyltransferase 1 | Protein Coding | P15907 | 57 | GC03P186930 | 4.41116619110107 | https://www.genecards.org/cgi-bin/carddisp.pl?gene=ST6GAL1 |  |
| CSNK2A2 | Casein Kinase 2 Alpha 2 | Protein Coding | P19784 | 58 | GC16M058157 | 4.40475511550903 | https://www.genecards.org/cgi-bin/carddisp.pl?gene=CSNK2A2 |  |
| FCGR2A | Fc Gamma Receptor IIa | Protein Coding | P12318 | 61 | GC01P161505 | 4.40440702438354 | https://www.genecards.org/cgi-bin/carddisp.pl?gene=FCGR2A |  |
| KCNE1 | Potassium Voltage-Gated Channel Subfamily E Regulatory Subunit 1 | Protein Coding | P15382 | 58 | GC21M034446 | 4.40106058120728 | https://www.genecards.org/cgi-bin/carddisp.pl?gene=KCNE1 |  |
| MPZ | Myelin Protein Zero | Protein Coding | P25189 | 55 | GC01M167641 | 4.39854907989502 | https://www.genecards.org/cgi-bin/carddisp.pl?gene=MPZ |  |
| MBL2 | Mannose Binding Lectin 2 | Protein Coding | P11226 | 60 | GC10M052760 | 4.39802074432373 | https://www.genecards.org/cgi-bin/carddisp.pl?gene=MBL2 |  |
| ADCYAP1 | Adenylate Cyclase Activating Polypeptide 1 | Protein Coding | P18509 | 55 | GC18P000895 | 4.37998390197754 | https://www.genecards.org/cgi-bin/carddisp.pl?gene=ADCYAP1 |  |
| SYK | Spleen Associated Tyrosine Kinase | Protein Coding | P43405 | 64 | GC09P106847 | 4.37773323059082 | https://www.genecards.org/cgi-bin/carddisp.pl?gene=SYK |  |
| PLAU | Plasminogen Activator, Urokinase | Protein Coding | P00749 | 64 | GC10P073909 | 4.37330627441406 | https://www.genecards.org/cgi-bin/carddisp.pl?gene=PLAU |  |
| MUSK | Muscle Associated Receptor Tyrosine Kinase | Protein Coding | O15146 | 61 | GC09P110668 | 4.37130212783813 | https://www.genecards.org/cgi-bin/carddisp.pl?gene=MUSK |  |
| GLB1L2 | Galactosidase Beta 1 Like 2 | Protein Coding | Q8IW92 | 44 | GC11P134331 | 4.36725473403931 | https://www.genecards.org/cgi-bin/carddisp.pl?gene=GLB1L2 |  |
| RALA | RAS Like Proto-Oncogene A | Protein Coding | P11233 | 60 | GC07P039622 | 4.36501455307007 | https://www.genecards.org/cgi-bin/carddisp.pl?gene=RALA |  |
| ACP1 | Acid Phosphatase 1 | Protein Coding | P24666 | 55 | GC02P001239 | 4.3535270690918 | https://www.genecards.org/cgi-bin/carddisp.pl?gene=ACP1 |  |
| KPNB1 | Karyopherin Subunit Beta 1 | Protein Coding | Q14974 | 57 | GC17P047649 | 4.34983921051025 | https://www.genecards.org/cgi-bin/carddisp.pl?gene=KPNB1 |  |
| GOT1L1 | Glutamic-Oxaloacetic Transaminase 1 Like 1 | Protein Coding | Q8NHS2 | 44 | GC08M038831 | 4.34802150726318 | https://www.genecards.org/cgi-bin/carddisp.pl?gene=GOT1L1 |  |
| GDF1 | Growth Differentiation Factor 1 | Protein Coding | P27539 | 49 | GC19M108411 | 4.30449247360229 | https://www.genecards.org/cgi-bin/carddisp.pl?gene=GDF1 |  |
| ARF6 | ARF GTPase 6 | Protein Coding | P62330 | 56 | GC14P058041 | 4.29653692245483 | https://www.genecards.org/cgi-bin/carddisp.pl?gene=ARF6 |  |
| XRCC5 | X-Ray Repair Cross Complementing 5 | Protein Coding | P13010 | 57 | GC02P216107 | 4.28513383865356 | https://www.genecards.org/cgi-bin/carddisp.pl?gene=XRCC5 |  |
| CEPT1 | Choline/Ethanolamine Phosphotransferase 1 | Protein Coding | Q9Y6K0 | 51 | GC01P111139 | 4.28510904312134 | https://www.genecards.org/cgi-bin/carddisp.pl?gene=CEPT1 |  |
| TP63 | Tumor Protein P63 | Protein Coding | Q9H3D4 | 58 | GC03P189598 | 4.27708625793457 | https://www.genecards.org/cgi-bin/carddisp.pl?gene=TP63 |  |
| PPIA | Peptidylprolyl Isomerase A | Protein Coding | P62937 | 58 | GC07P045440 | 4.27465963363647 | https://www.genecards.org/cgi-bin/carddisp.pl?gene=PPIA |  |
| CIB2 | Calcium And Integrin Binding Family Member 2 | Protein Coding | O75838 | 51 | GC15M078104 | 4.27271938323975 | https://www.genecards.org/cgi-bin/carddisp.pl?gene=CIB2 |  |
| SYNE2 | Spectrin Repeat Containing Nuclear Envelope Protein 2 | Protein Coding | Q8WXH0 | 52 | GC14P063761 | 4.26733207702637 | https://www.genecards.org/cgi-bin/carddisp.pl?gene=SYNE2 |  |
| DGKA | Diacylglycerol Kinase Alpha | Protein Coding | P23743 | 55 | GC12P055927 | 4.26720857620239 | https://www.genecards.org/cgi-bin/carddisp.pl?gene=DGKA |  |
| PNLIPRP2 | Pancreatic Lipase Related Protein 2 (Gene/Pseudogene) | Protein Coding | P54317 | 41 | GC10P116620 | 4.26613616943359 | https://www.genecards.org/cgi-bin/carddisp.pl?gene=PNLIPRP2 |  |
| STARD4 | StAR Related Lipid Transfer Domain Containing 4 | Protein Coding | Q96DR4 | 47 | GC05M111496 | 4.26611423492432 | https://www.genecards.org/cgi-bin/carddisp.pl?gene=STARD4 |  |
| FYN | FYN Proto-Oncogene, Src Family Tyrosine Kinase | Protein Coding | P06241 | 60 | GC06M111660 | 4.25366926193237 | https://www.genecards.org/cgi-bin/carddisp.pl?gene=FYN |  |
| ST8SIA3 | ST8 Alpha-N-Acetyl-Neuraminide Alpha-2,8-Sialyltransferase 3 | Protein Coding | O43173 | 42 | GC18P057350 | 4.24698209762573 | https://www.genecards.org/cgi-bin/carddisp.pl?gene=ST8SIA3 |  |
| LGALS1 | Galectin 1 | Protein Coding | P09382 | 56 | GC22P037675 | 4.24312162399292 | https://www.genecards.org/cgi-bin/carddisp.pl?gene=LGALS1 |  |
| GSN | Gelsolin | Protein Coding | P06396 | 62 | GC09P121201 | 4.23696994781494 | https://www.genecards.org/cgi-bin/carddisp.pl?gene=GSN |  |
| MIR758 | MicroRNA 758 | RNA Gene |  | 21 | GC14P121395 | 4.2350492477417 | https://www.genecards.org/cgi-bin/carddisp.pl?gene=MIR758 |  |
| ST6GALNAC3 | ST6 N-Acetylgalactosaminide Alpha-2,6-Sialyltransferase 3 | Protein Coding | Q8NDV1 | 44 | GC01P076074 | 4.23369836807251 | https://www.genecards.org/cgi-bin/carddisp.pl?gene=ST6GALNAC3 |  |
| RASA2 | RAS P21 Protein Activator 2 | Protein Coding | Q15283 | 51 | GC03P141487 | 4.22462368011475 | https://www.genecards.org/cgi-bin/carddisp.pl?gene=RASA2 |  |
| IL12RB2 | Interleukin 12 Receptor Subunit Beta 2 | Protein Coding | Q99665 | 57 | GC01P067307 | 4.22446060180664 | https://www.genecards.org/cgi-bin/carddisp.pl?gene=IL12RB2 |  |
| LPIN3 | Lipin 3 | Protein Coding | Q9BQK8 | 44 | GC20P041340 | 4.22281169891357 | https://www.genecards.org/cgi-bin/carddisp.pl?gene=LPIN3 |  |
| GLB1L3 | Galactosidase Beta 1 Like 3 | Protein Coding | Q8NCI6 | 45 | GC11P134274 | 4.2202672958374 | https://www.genecards.org/cgi-bin/carddisp.pl?gene=GLB1L3 |  |
| ITPK1 | Inositol-Tetrakisphosphate 1-Kinase | Protein Coding | Q13572 | 51 | GC14M092936 | 4.21277618408203 | https://www.genecards.org/cgi-bin/carddisp.pl?gene=ITPK1 |  |
| PSMD2 | Proteasome 26S Subunit Ubiquitin Receptor, Non-ATPase 2 | Protein Coding | Q13200 | 55 | GC03P185761 | 4.19511890411377 | https://www.genecards.org/cgi-bin/carddisp.pl?gene=PSMD2 |  |
| XIAP | X-Linked Inhibitor Of Apoptosis | Protein Coding | P98170 | 64 | GC0XP123859 | 4.16250944137573 | https://www.genecards.org/cgi-bin/carddisp.pl?gene=XIAP |  |
| CTNND2 | Catenin Delta 2 | Protein Coding | Q9UQB3 | 55 | GC05M010971 | 4.16249418258667 | https://www.genecards.org/cgi-bin/carddisp.pl?gene=CTNND2 |  |
| SBF1 | SET Binding Factor 1 | Protein Coding | O95248 | 54 | GC22M086278 | 4.16097545623779 | https://www.genecards.org/cgi-bin/carddisp.pl?gene=SBF1 |  |
| TRAF2 | TNF Receptor Associated Factor 2 | Protein Coding | Q12933 | 58 | GC09P136881 | 4.15112972259521 | https://www.genecards.org/cgi-bin/carddisp.pl?gene=TRAF2 |  |
| PCDH7 | Protocadherin 7 | Protein Coding | O60245 | 50 | GC04P030725 | 4.15034341812134 | https://www.genecards.org/cgi-bin/carddisp.pl?gene=PCDH7 |  |
| ABL1 | ABL Proto-Oncogene 1, Non-Receptor Tyrosine Kinase | Protein Coding | P00519 | 65 | GC09P130713 | 4.14732456207275 | https://www.genecards.org/cgi-bin/carddisp.pl?gene=ABL1 |  |
| GRB2 | Growth Factor Receptor Bound Protein 2 | Protein Coding | P62993 | 60 | GC17M075318 | 4.14721918106079 | https://www.genecards.org/cgi-bin/carddisp.pl?gene=GRB2 |  |
| AZIN2 | Antizyme Inhibitor 2 | Protein Coding | Q96A70 | 52 | GC01P033081 | 4.14564085006714 | https://www.genecards.org/cgi-bin/carddisp.pl?gene=AZIN2 |  |
| BSG | Basigin (Ok Blood Group) | Protein Coding | P35613 | 57 | GC19P000571 | 4.14479446411133 | https://www.genecards.org/cgi-bin/carddisp.pl?gene=BSG |  |
| IL18R1 | Interleukin 18 Receptor 1 | Protein Coding | Q13478 | 56 | GC02P102311 | 4.14235877990723 | https://www.genecards.org/cgi-bin/carddisp.pl?gene=IL18R1 |  |
| PLD3 | Phospholipase D Family Member 3 | Protein Coding | Q8IV08 | 54 | GC19P040348 | 4.14195108413696 | https://www.genecards.org/cgi-bin/carddisp.pl?gene=PLD3 |  |
| GLB1L | Galactosidase Beta 1 Like | Protein Coding | Q6UWU2 | 45 | GC02M219237 | 4.13128900527954 | https://www.genecards.org/cgi-bin/carddisp.pl?gene=GLB1L |  |
| BEST1 | Bestrophin 1 | Protein Coding | O76090 | 56 | GC11P061949 | 4.1277642250061 | https://www.genecards.org/cgi-bin/carddisp.pl?gene=BEST1 |  |
| DCC | DCC Netrin 1 Receptor | Protein Coding | P43146 | 60 | GC18P052340 | 4.12418842315674 | https://www.genecards.org/cgi-bin/carddisp.pl?gene=DCC |  |
| HLA-C | Major Histocompatibility Complex, Class I, C | Protein Coding | P10321 | 57 | GC06M106435 | 4.10329723358154 | https://www.genecards.org/cgi-bin/carddisp.pl?gene=HLA-C |  |
| TRPA1 | Transient Receptor Potential Cation Channel Subfamily A Member 1 | Protein Coding | O75762 | 60 | GC08M072019 | 4.10126829147339 | https://www.genecards.org/cgi-bin/carddisp.pl?gene=TRPA1 |  |
| MIR365A | MicroRNA 365a | RNA Gene |  | 27 | GC16P014309 | 4.1011700630188 | https://www.genecards.org/cgi-bin/carddisp.pl?gene=MIR365A |  |
| CD19 | CD19 Molecule | Protein Coding | P15391 | 64 | GC16P120912 | 4.08544588088989 | https://www.genecards.org/cgi-bin/carddisp.pl?gene=CD19 |  |
| B3GNT5 | UDP-GlcNAc:BetaGal Beta-1,3-N-Acetylglucosaminyltransferase 5 | Protein Coding | Q9BYG0 | 50 | GC03P183253 | 4.08459377288818 | https://www.genecards.org/cgi-bin/carddisp.pl?gene=B3GNT5 |  |
| MCL1 | MCL1 Apoptosis Regulator, BCL2 Family Member | Protein Coding | Q07820 | 62 | GC01M167251 | 4.06372261047363 | https://www.genecards.org/cgi-bin/carddisp.pl?gene=MCL1 |  |
| AIF1 | Allograft Inflammatory Factor 1 | Protein Coding | P55008 | 51 | GC06P181892 | 4.05443286895752 | https://www.genecards.org/cgi-bin/carddisp.pl?gene=AIF1 |  |
| TM4SF5 | Transmembrane 4 L Six Family Member 5 | Protein Coding | O14894 | 42 | GC17P004771 | 4.05404329299927 | https://www.genecards.org/cgi-bin/carddisp.pl?gene=TM4SF5 |  |
| AP1G1 | Adaptor Related Protein Complex 1 Subunit Gamma 1 | Protein Coding | O43747 | 52 | GC16M071729 | 4.05159616470337 | https://www.genecards.org/cgi-bin/carddisp.pl?gene=AP1G1 |  |
| AMFR | Autocrine Motility Factor Receptor | Protein Coding | Q9UKV5 | 57 | GC16M056361 | 4.04594135284424 | https://www.genecards.org/cgi-bin/carddisp.pl?gene=AMFR |  |
| BAD | BCL2 Associated Agonist Of Cell Death | Protein Coding | Q92934 | 57 | GC11M139722 | 4.03979730606079 | https://www.genecards.org/cgi-bin/carddisp.pl?gene=BAD |  |
| FCGR2B | Fc Gamma Receptor IIb | Protein Coding | P31994 | 61 | GC01P177026 | 4.0303692817688 | https://www.genecards.org/cgi-bin/carddisp.pl?gene=FCGR2B |  |
| DUSP19 | Dual Specificity Phosphatase 19 | Protein Coding | Q8WTR2 | 49 | GC02P183078 | 4.03022813796997 | https://www.genecards.org/cgi-bin/carddisp.pl?gene=DUSP19 |  |
| LOC129999940 | ATAC-STARR-Seq Lymphoblastoid Silent Region 18966 | Functional Element |  | 7 | GC08P018108 | 4.01991128921509 | https://www.genecards.org/cgi-bin/carddisp.pl?gene=LOC129999940 |  |
| CNTNAP4 | Contactin Associated Protein Family Member 4 | Protein Coding | Q9C0A0 | 48 | GC16P122197 | 4.01518392562866 | https://www.genecards.org/cgi-bin/carddisp.pl?gene=CNTNAP4 |  |
| PWAR4 | Prader Willi/Angelman Region RNA 4 | RNA Gene |  | 17 | GC15P025211 | 4.00536966323853 | https://www.genecards.org/cgi-bin/carddisp.pl?gene=PWAR4 |  |
| CDH2 | Cadherin 2 | Protein Coding | P19022 | 64 | GC18M038314 | 4.00527429580688 | https://www.genecards.org/cgi-bin/carddisp.pl?gene=CDH2 |  |
| KCNE5 | Potassium Voltage-Gated Channel Subfamily E Regulatory Subunit 5 | Protein Coding | Q9UJ90 | 45 | GC0XM109623 | 4.00198745727539 | https://www.genecards.org/cgi-bin/carddisp.pl?gene=KCNE5 |  |
| H2AZ1 | H2A.Z Variant Histone 1 | Protein Coding | P0C0S5 | 53 | GC04M099949 | 3.9965763092041 | https://www.genecards.org/cgi-bin/carddisp.pl?gene=H2AZ1 |  |
| APCS | Amyloid P Component, Serum | Protein Coding | P02743 | 55 | GC01P159587 | 3.995032787 | https://www.genecards.org/cgi-bin/carddisp.pl?gene=APCS |  |
| DLEU1 | Deleted In Lymphocytic Leukemia 1 | RNA Gene | O43261 | 37 | GC13P051743 | 3.99097084999084 | https://www.genecards.org/cgi-bin/carddisp.pl?gene=DLEU1 |  |
| PWAR1 | Prader Willi/Angelman Region RNA 1 | RNA Gene |  | 22 | GC15P025135 | 3.98990297317505 | https://www.genecards.org/cgi-bin/carddisp.pl?gene=PWAR1 |  |
| FBN3 | Fibrillin 3 | Protein Coding | Q75N90 | 50 | GC19M008065 | 3.98976469039917 | https://www.genecards.org/cgi-bin/carddisp.pl?gene=FBN3 |  |
| CDH11 | Cadherin 11 | Protein Coding | P55287 | 59 | GC16M064943 | 3.96647882461548 | https://www.genecards.org/cgi-bin/carddisp.pl?gene=CDH11 |  |
| MSN | Moesin | Protein Coding | P26038 | 60 | GC0XP065588 | 3.92233180999756 | https://www.genecards.org/cgi-bin/carddisp.pl?gene=MSN |  |
| ADM | Adrenomedullin | Protein Coding | P35318 | 57 | GC11P021130 | 3.91065692901611 | https://www.genecards.org/cgi-bin/carddisp.pl?gene=ADM |  |
| GADL1 | Glutamate Decarboxylase Like 1 | Protein Coding | Q6ZQY3 | 46 | GC03M030743 | 3.90803980827332 | https://www.genecards.org/cgi-bin/carddisp.pl?gene=GADL1 |  |
| LNPEP | Leucyl And Cystinyl Aminopeptidase | Protein Coding | Q9UIQ6 | 58 | GC05P096935 | 3.90610456466675 | https://www.genecards.org/cgi-bin/carddisp.pl?gene=LNPEP |  |
| FLG | Filaggrin | Protein Coding | P20930 | 54 | GC01M167330 | 3.88944816589355 | https://www.genecards.org/cgi-bin/carddisp.pl?gene=FLG |  |
| TRPV5 | Transient Receptor Potential Cation Channel Subfamily V Member 5 | Protein Coding | Q9NQA5 | 54 | GC07M142908 | 3.88122177124023 | https://www.genecards.org/cgi-bin/carddisp.pl?gene=TRPV5 |  |
| ATG5 | Autophagy Related 5 | Protein Coding | Q9H1Y0 | 58 | GC06M106045 | 3.86756634712219 | https://www.genecards.org/cgi-bin/carddisp.pl?gene=ATG5 |  |
| GPER1 | G Protein-Coupled Estrogen Receptor 1 | Protein Coding | Q99527 | 53 | GC07P018825 | 3.86465692520142 | https://www.genecards.org/cgi-bin/carddisp.pl?gene=GPER1 |  |
| BIRC5 | Baculoviral IAP Repeat Containing 5 | Protein Coding | O15392 | 58 | GC17P078214 | 3.85505414009094 | https://www.genecards.org/cgi-bin/carddisp.pl?gene=BIRC5 |  |
| B3GALT1 | Beta-1,3-Galactosyltransferase 1 | Protein Coding | Q9Y5Z6 | 42 | GC02P167306 | 3.84742259979248 | https://www.genecards.org/cgi-bin/carddisp.pl?gene=B3GALT1 |  |
| PTK2 | Protein Tyrosine Kinase 2 | Protein Coding | Q05397 | 61 | GC08M140657 | 3.82450175285339 | https://www.genecards.org/cgi-bin/carddisp.pl?gene=PTK2 |  |
| B3GALT5 | Beta-1,3-Galactosyltransferase 5 | Protein Coding | Q9Y2C3 | 51 | GC21P039556 | 3.81725525856018 | https://www.genecards.org/cgi-bin/carddisp.pl?gene=B3GALT5 |  |
| PLA1A | Phospholipase A1 Member A | Protein Coding | Q53H76 | 52 | GC03P119597 | 3.80745124816895 | https://www.genecards.org/cgi-bin/carddisp.pl?gene=PLA1A |  |
| CD14 | CD14 Molecule | Protein Coding | P08571 | 58 | GC05M140631 | 3.80615091323853 | https://www.genecards.org/cgi-bin/carddisp.pl?gene=CD14 |  |
| CAMP | Cathelicidin Antimicrobial Peptide | Protein Coding | P49913 | 53 | GC03P064957 | 3.79783248901367 | https://www.genecards.org/cgi-bin/carddisp.pl?gene=CAMP |  |
| RHO | Rhodopsin | Protein Coding | P08100 | 60 | GC03P143117 | 3.78907155990601 | https://www.genecards.org/cgi-bin/carddisp.pl?gene=RHO |  |
| HNF1A-AS1 | HNF1A Antisense RNA 1 | RNA Gene |  | 28 | GC12M124665 | 3.77640080451965 | https://www.genecards.org/cgi-bin/carddisp.pl?gene=HNF1A-AS1 |  |
| RASA1 | RAS P21 Protein Activator 1 | Protein Coding | P20936 | 60 | GC05P087267 | 3.77587556838989 | https://www.genecards.org/cgi-bin/carddisp.pl?gene=RASA1 |  |
| PBX1 | PBX Homeobox 1 | Protein Coding | P40424 | 60 | GC01P177083 | 3.76130247116089 | https://www.genecards.org/cgi-bin/carddisp.pl?gene=PBX1 |  |
| PTPA | Protein Phosphatase 2 Phosphatase Activator | Protein Coding | Q15257 | 58 | GC09P152908 | 3.73326563835144 | https://www.genecards.org/cgi-bin/carddisp.pl?gene=PTPA |  |
| PITPNB | Phosphatidylinositol Transfer Protein Beta | Protein Coding | P48739 | 51 | GC22M027851 | 3.72960114479065 | https://www.genecards.org/cgi-bin/carddisp.pl?gene=PITPNB |  |
| NTF3 | Neurotrophin 3 | Protein Coding | P20783 | 57 | GC12P074017 | 3.71280837059021 | https://www.genecards.org/cgi-bin/carddisp.pl?gene=NTF3 |  |
| MITF | Melanocyte Inducing Transcription Factor | Protein Coding | O75030 | 61 | GC03P069740 | 3.69678854942322 | https://www.genecards.org/cgi-bin/carddisp.pl?gene=MITF |  |
| PDIA3 | Protein Disulfide Isomerase Family A Member 3 | Protein Coding | P30101 | 57 | GC15P043746 | 3.69268226623535 | https://www.genecards.org/cgi-bin/carddisp.pl?gene=PDIA3 |  |
| MMP14 | Matrix Metallopeptidase 14 | Protein Coding | P50281 | 64 | GC14P057548 | 3.68734216690063 | https://www.genecards.org/cgi-bin/carddisp.pl?gene=MMP14 |  |
| TRPC1 | Transient Receptor Potential Cation Channel Subfamily C Member 1 | Protein Coding | P48995 | 52 | GC03P142724 | 3.68625807762146 | https://www.genecards.org/cgi-bin/carddisp.pl?gene=TRPC1 |  |
| NCSTN | Nicastrin | Protein Coding | Q92542 | 62 | GC01P160343 | 3.68609428405762 | https://www.genecards.org/cgi-bin/carddisp.pl?gene=NCSTN |  |
| ERLIN1 | ER Lipid Raft Associated 1 | Protein Coding | O75477 | 54 | GC10M100150 | 3.67284178733826 | https://www.genecards.org/cgi-bin/carddisp.pl?gene=ERLIN1 |  |
| SMPD5 | Sphingomyelin Phosphodiesterase 5 (Pseudogene) | Pseudogene |  | 12 | GC08P148698 | 3.65867567062378 | https://www.genecards.org/cgi-bin/carddisp.pl?gene=SMPD5 |  |
| PDXP | Pyridoxal Phosphatase | Protein Coding | Q96GD0 | 50 | GC22P037658 | 3.65386915206909 | https://www.genecards.org/cgi-bin/carddisp.pl?gene=PDXP |  |
| CSAD | Cysteine Sulfinic Acid Decarboxylase | Protein Coding | Q9Y600 | 50 | GC12M053160 | 3.64209270477295 | https://www.genecards.org/cgi-bin/carddisp.pl?gene=CSAD |  |
| PHB1 | Prohibitin 1 | Protein Coding | P35232 | 58 | GC17M097550 | 3.62558460235596 | https://www.genecards.org/cgi-bin/carddisp.pl?gene=PHB1 |  |
| DGKG | Diacylglycerol Kinase Gamma | Protein Coding | P49619 | 52 | GC03M186105 | 3.62515020370483 | https://www.genecards.org/cgi-bin/carddisp.pl?gene=DGKG |  |
| RAB8A | RAB8A, Member RAS Oncogene Family | Protein Coding | P61006 | 52 | GC19P016111 | 3.62256288528442 | https://www.genecards.org/cgi-bin/carddisp.pl?gene=RAB8A |  |
| MLH1 | MutL Homolog 1 | Protein Coding | P40692 | 62 | GC03P036993 | 3.62237691879272 | https://www.genecards.org/cgi-bin/carddisp.pl?gene=MLH1 |  |
| PGRMC1 | Progesterone Receptor Membrane Component 1 | Protein Coding | O00264 | 57 | GC0XP119236 | 3.61935234069824 | https://www.genecards.org/cgi-bin/carddisp.pl?gene=PGRMC1 |  |
| MSBP1 | Minisatellite Binding Protein 1 | Protein Coding |  | 10 | GC00U990213 | 3.59434509277344 | https://www.genecards.org/cgi-bin/carddisp.pl?gene=MSBP1 |  |
| OSBPL9 | Oxysterol Binding Protein Like 9 | Protein Coding | Q96SU4 | 47 | GC01P078322 | 3.59217381477356 | https://www.genecards.org/cgi-bin/carddisp.pl?gene=OSBPL9 |  |
| SKAP2 | Src Kinase Associated Phosphoprotein 2 | Protein Coding | O75563 | 49 | GC07M026654 | 3.59207963943481 | https://www.genecards.org/cgi-bin/carddisp.pl?gene=SKAP2 |  |
| TRPC3 | Transient Receptor Potential Cation Channel Subfamily C Member 3 | Protein Coding | Q13507 | 58 | GC04M121879 | 3.58678007125854 | https://www.genecards.org/cgi-bin/carddisp.pl?gene=TRPC3 |  |
| ZNF512 | Zinc Finger Protein 512 | Protein Coding | Q96ME7 | 40 | GC02P027582 | 3.57882499694824 | https://www.genecards.org/cgi-bin/carddisp.pl?gene=ZNF512 |  |
| PSENEN | Presenilin Enhancer, Gamma-Secretase Subunit | Protein Coding | Q9NZ42 | 57 | GC19P152399 | 3.5483021736145 | https://www.genecards.org/cgi-bin/carddisp.pl?gene=PSENEN |  |
| BNIP3 | BCL2 Interacting Protein 3 | Protein Coding | Q12983 | 54 | GC10M131966 | 3.54820156097412 | https://www.genecards.org/cgi-bin/carddisp.pl?gene=BNIP3 |  |
| TLR9 | Toll Like Receptor 9 | Protein Coding | Q9NR96 | 58 | GC03M054885 | 3.52476119995117 | https://www.genecards.org/cgi-bin/carddisp.pl?gene=TLR9 |  |
| FCGR3B | Fc Gamma Receptor IIIb | Protein Coding | O75015 | 55 | GC01M161623 | 3.51515674591064 | https://www.genecards.org/cgi-bin/carddisp.pl?gene=FCGR3B |  |
| ST6GALNAC4 | ST6 N-Acetylgalactosaminide Alpha-2,6-Sialyltransferase 4 | Protein Coding | Q9H4F1 | 47 | GC09M127907 | 3.51433515548706 | https://www.genecards.org/cgi-bin/carddisp.pl?gene=ST6GALNAC4 |  |
| PLA2G4C | Phospholipase A2 Group IVC | Protein Coding | Q9UP65 | 46 | GC19M048047 | 3.51341676712036 | https://www.genecards.org/cgi-bin/carddisp.pl?gene=PLA2G4C |  |
| CHRNA1 | Cholinergic Receptor Nicotinic Alpha 1 Subunit | Protein Coding | P02708 | 59 | GC02M174747 | 3.50070667266846 | https://www.genecards.org/cgi-bin/carddisp.pl?gene=CHRNA1 |  |
| CPTP | Ceramide-1-Phosphate Transfer Protein | Protein Coding | Q5TA50 | 44 | GC01P074925 | 3.49889516830444 | https://www.genecards.org/cgi-bin/carddisp.pl?gene=CPTP |  |
| PCSK2 | Proprotein Convertase Subtilisin/Kexin Type 2 | Protein Coding | P16519 | 57 | GC20P017226 | 3.49286842346191 | https://www.genecards.org/cgi-bin/carddisp.pl?gene=PCSK2 |  |
| C5AR1 | Complement C5a Receptor 1 | Protein Coding | P21730 | 58 | GC19P047290 | 3.48717308044434 | https://www.genecards.org/cgi-bin/carddisp.pl?gene=C5AR1 |  |
| PRKCE | Protein Kinase C Epsilon | Protein Coding | Q02156 | 61 | GC02P045651 | 3.47340726852417 | https://www.genecards.org/cgi-bin/carddisp.pl?gene=PRKCE |  |
| ARSJ | Arylsulfatase Family Member J | Protein Coding | Q5FYB0 | 44 | GC04M113901 | 3.47314739227295 | https://www.genecards.org/cgi-bin/carddisp.pl?gene=ARSJ |  |
| H2AC20 | H2A Clustered Histone 20 | Protein Coding | Q16777 | 49 | GC01P176455 | 3.47270679473877 | https://www.genecards.org/cgi-bin/carddisp.pl?gene=H2AC20 |  |
| PSMC6 | Proteasome 26S Subunit, ATPase 6 | Protein Coding | P62333 | 52 | GC14P052707 | 3.46677875518799 | https://www.genecards.org/cgi-bin/carddisp.pl?gene=PSMC6 |  |
| RAB4A | RAB4A, Member RAS Oncogene Family | Protein Coding | P20338 | 54 | GC01P229271 | 3.44721412658691 | https://www.genecards.org/cgi-bin/carddisp.pl?gene=RAB4A |  |
| CCL3 | C-C Motif Chemokine Ligand 3 | Protein Coding | P10147 | 54 | GC17M036088 | 3.4471378326416 | https://www.genecards.org/cgi-bin/carddisp.pl?gene=CCL3 |  |
| ZNF101 | Zinc Finger Protein 101 | Protein Coding | Q8IZC7 | 38 | GC19P152116 | 3.44606256484985 | https://www.genecards.org/cgi-bin/carddisp.pl?gene=ZNF101 |  |
| FKBP4 | FKBP Prolyl Isomerase 4 | Protein Coding | Q02790 | 57 | GC12P073950 | 3.44555592536926 | https://www.genecards.org/cgi-bin/carddisp.pl?gene=FKBP4 |  |
| ST6GAL2 | ST6 Beta-Galactoside Alpha-2,6-Sialyltransferase 2 | Protein Coding | Q96JF0 | 47 | GC02M106801 | 3.43711996078491 | https://www.genecards.org/cgi-bin/carddisp.pl?gene=ST6GAL2 |  |
| NTAN1 | N-Terminal Asparagine Amidase | Protein Coding | Q96AB6 | 45 | GC16M015037 | 3.43220138549805 | https://www.genecards.org/cgi-bin/carddisp.pl?gene=NTAN1 |  |
| AP2A1 | Adaptor Related Protein Complex 2 Subunit Alpha 1 | Protein Coding | O95782 | 51 | GC19P152884 | 3.42948532104492 | https://www.genecards.org/cgi-bin/carddisp.pl?gene=AP2A1 |  |
| SCLY | Selenocysteine Lyase | Protein Coding | Q96I15 | 46 | GC02P238077 | 3.42642140388489 | https://www.genecards.org/cgi-bin/carddisp.pl?gene=SCLY |  |
| STARD5 | StAR Related Lipid Transfer Domain Containing 5 | Protein Coding | Q9NSY2 | 47 | GC15M081309 | 3.41995525360107 | https://www.genecards.org/cgi-bin/carddisp.pl?gene=STARD5 |  |
| APOC1P1 | Apolipoprotein C1 Pseudogene 1 | Pseudogene |  | 14 | GC19P044926 | 3.41438841819763 | https://www.genecards.org/cgi-bin/carddisp.pl?gene=APOC1P1 |  |
| PLAUR | Plasminogen Activator, Urokinase Receptor | Protein Coding | Q03405 | 57 | GC19M043646 | 3.40511703491211 | https://www.genecards.org/cgi-bin/carddisp.pl?gene=PLAUR |  |
| CD1D | CD1d Molecule | Protein Coding | P15813 | 55 | GC01P158178 | 3.40081524848938 | https://www.genecards.org/cgi-bin/carddisp.pl?gene=CD1D |  |
| RNU1-1 | RNA, U1 Small Nuclear 1 | RNA Gene |  | 22 | GC01M016514 | 3.3966851234436 | https://www.genecards.org/cgi-bin/carddisp.pl?gene=RNU1-1 |  |
| SACM1L | SAC1 Like Phosphatidylinositide Phosphatase | Protein Coding | Q9NTJ5 | 43 | GC03P064833 | 3.3919506072998 | https://www.genecards.org/cgi-bin/carddisp.pl?gene=SACM1L |  |
| CTSH | Cathepsin H | Protein Coding | P09668 | 58 | GC15M162922 | 3.39079880714417 | https://www.genecards.org/cgi-bin/carddisp.pl?gene=CTSH |  |
| RHD | Rh Blood Group D Antigen | Protein Coding | Q02161 | 54 | GC01P025272 | 3.37900304794312 | https://www.genecards.org/cgi-bin/carddisp.pl?gene=RHD |  |
| CD81 | CD81 Molecule | Protein Coding | P60033 | 59 | GC11P020330 | 3.37556052207947 | https://www.genecards.org/cgi-bin/carddisp.pl?gene=CD81 |  |
| CDKN1B | Cyclin Dependent Kinase Inhibitor 1B | Protein Coding | P46527 | 61 | GC12P074277 | 3.37423515319824 | https://www.genecards.org/cgi-bin/carddisp.pl?gene=CDKN1B |  |
| CAMK2G | Calcium/Calmodulin Dependent Protein Kinase II Gamma | Protein Coding | Q13555 | 62 | GC10M073812 | 3.36184740066528 | https://www.genecards.org/cgi-bin/carddisp.pl?gene=CAMK2G |  |
| DGKH | Diacylglycerol Kinase Eta | Protein Coding | Q86XP1 | 49 | GC13P042040 | 3.35828638076782 | https://www.genecards.org/cgi-bin/carddisp.pl?gene=DGKH |  |
| CXCL12 | C-X-C Motif Chemokine Ligand 12 | Protein Coding | P48061 | 59 | GC10M044370 | 3.34271168708801 | https://www.genecards.org/cgi-bin/carddisp.pl?gene=CXCL12 |  |
| CASP10 | Caspase 10 | Protein Coding | Q92851 | 60 | GC02P201182 | 3.33129549026489 | https://www.genecards.org/cgi-bin/carddisp.pl?gene=CASP10 |  |
| AQP1 | Aquaporin 1 (Colton Blood Group) | Protein Coding | P29972 | 60 | GC07P030911 | 3.3304762840271 | https://www.genecards.org/cgi-bin/carddisp.pl?gene=AQP1 |  |
| RAB3IL1 | RAB3A Interacting Protein Like 1 | Protein Coding | Q8TBN0 | 48 | GC11M061897 | 3.32269406318665 | https://www.genecards.org/cgi-bin/carddisp.pl?gene=RAB3IL1 |  |
| CRK | CRK Proto-Oncogene, Adaptor Protein | Protein Coding | P46108 | 57 | GC17M001420 | 3.31271815299988 | https://www.genecards.org/cgi-bin/carddisp.pl?gene=CRK |  |
| MRC1 | Mannose Receptor C-Type 1 | Protein Coding | P22897 | 51 | GC10P017809 | 3.3126847743988 | https://www.genecards.org/cgi-bin/carddisp.pl?gene=MRC1 |  |
| COPG1 | COPI Coat Complex Subunit Gamma 1 | Protein Coding | Q9Y678 | 52 | GC03P129249 | 3.30031514167786 | https://www.genecards.org/cgi-bin/carddisp.pl?gene=COPG1 |  |
| CNBP | CCHC-Type Zinc Finger Nucleic Acid Binding Protein | Protein Coding | P62633 | 55 | GC03M129167 | 3.29581594467163 | https://www.genecards.org/cgi-bin/carddisp.pl?gene=CNBP |  |
| PKMYT1 | Protein Kinase, Membrane Associated Tyrosine/Threonine 1 | Protein Coding | Q99640 | 53 | GC16M049939 | 3.28850245475769 | https://www.genecards.org/cgi-bin/carddisp.pl?gene=PKMYT1 |  |
| MAP2K4 | Mitogen-Activated Protein Kinase Kinase 4 | Protein Coding | P45985 | 62 | GC17P012020 | 3.28841114044189 | https://www.genecards.org/cgi-bin/carddisp.pl?gene=MAP2K4 |  |
| PKD2L1 | Polycystin 2 Like 1, Transient Receptor Potential Cation Channel | Protein Coding | Q9P0L9 | 55 | GC10M100288 | 3.28572225570679 | https://www.genecards.org/cgi-bin/carddisp.pl?gene=PKD2L1 |  |
| CCL4 | C-C Motif Chemokine Ligand 4 | Protein Coding | P13236 | 52 | GC17P036103 | 3.27883529663086 | https://www.genecards.org/cgi-bin/carddisp.pl?gene=CCL4 |  |
| SRF | Serum Response Factor | Protein Coding | P11831 | 56 | GC06P043171 | 3.27794599533081 | https://www.genecards.org/cgi-bin/carddisp.pl?gene=SRF |  |
| PLA2G15 | Phospholipase A2 Group XV | Protein Coding | Q8NCC3 | 49 | GC16P068245 | 3.25023555755615 | https://www.genecards.org/cgi-bin/carddisp.pl?gene=PLA2G15 |  |
| PPID | Peptidylprolyl Isomerase D | Protein Coding | Q08752 | 57 | GC04M158709 | 3.24945783615112 | https://www.genecards.org/cgi-bin/carddisp.pl?gene=PPID |  |
| KCNN1 | Potassium Calcium-Activated Channel Subfamily N Member 1 | Protein Coding | Q92952 | 50 | GC19P152096 | 3.24457597732544 | https://www.genecards.org/cgi-bin/carddisp.pl?gene=KCNN1 |  |
| LNC-LBCS | LncRNA Bladder And Prostate Cancer Suppressor, HnRNPK Interacting | RNA Gene |  | 19 | GC06M019306 | 3.24137735366821 | https://www.genecards.org/cgi-bin/carddisp.pl?gene=LNC-LBCS |  |
| IL12RB1 | Interleukin 12 Receptor Subunit Beta 1 | Protein Coding | P42701 | 58 | GC19M018058 | 3.24136257171631 | https://www.genecards.org/cgi-bin/carddisp.pl?gene=IL12RB1 |  |
| BCYRN1 | Brain Cytoplasmic RNA 1 | RNA Gene |  | 28 | GC02P047253 | 3.23664593696594 | https://www.genecards.org/cgi-bin/carddisp.pl?gene=BCYRN1 |  |
| B4GALT2 | Beta-1,4-Galactosyltransferase 2 | Protein Coding | O60909 | 49 | GC01P043978 | 3.23015427589417 | https://www.genecards.org/cgi-bin/carddisp.pl?gene=B4GALT2 |  |
| RAB10 | RAB10, Member RAS Oncogene Family | Protein Coding | P61026 | 54 | GC02P026033 | 3.22593283653259 | https://www.genecards.org/cgi-bin/carddisp.pl?gene=RAB10 |  |
| AGFG1 | ArfGAP With FG Repeats 1 | Protein Coding | P52594 | 52 | GC02P227473 | 3.21910715103149 | https://www.genecards.org/cgi-bin/carddisp.pl?gene=AGFG1 |  |
| OSBPL3 | Oxysterol Binding Protein Like 3 | Protein Coding | Q9H4L5 | 46 | GC07M024836 | 3.20594787597656 | https://www.genecards.org/cgi-bin/carddisp.pl?gene=OSBPL3 |  |
| MAX | MYC Associated Factor X | Protein Coding | P61244 | 59 | GC14M065425 | 3.19651031494141 | https://www.genecards.org/cgi-bin/carddisp.pl?gene=MAX |  |
| RPL28P4 | Ribosomal Protein L28 Pseudogene 4 | Pseudogene |  | 6 | GC15P058200 | 3.19325590133667 | https://www.genecards.org/cgi-bin/carddisp.pl?gene=RPL28P4 |  |
| LPAR1 | Lysophosphatidic Acid Receptor 1 | Protein Coding | Q92633 | 59 | GC09M110873 | 3.19203591346741 | https://www.genecards.org/cgi-bin/carddisp.pl?gene=LPAR1 |  |
| RDX | Radixin | Protein Coding | P35241 | 61 | GC11M109864 | 3.18936777114868 | https://www.genecards.org/cgi-bin/carddisp.pl?gene=RDX |  |
| PPBP | Pro-Platelet Basic Protein | Protein Coding | P02775 | 55 | GC04M073986 | 3.17647075653076 | https://www.genecards.org/cgi-bin/carddisp.pl?gene=PPBP |  |
| PIDD1 | P53-Induced Death Domain Protein 1 | Protein Coding | Q9HB75 | 50 | GC11M015067 | 3.16489505767822 | https://www.genecards.org/cgi-bin/carddisp.pl?gene=PIDD1 |  |
| KLF14 | KLF Transcription Factor 14 | Protein Coding | Q8TD94 | 43 | GC07M130731 | 3.16366291046143 | https://www.genecards.org/cgi-bin/carddisp.pl?gene=KLF14 |  |
| NHERF1 | NHERF Family PDZ Scaffold Protein 1 | Protein Coding | O14745 | 57 | GC17P155557 | 3.15811061859131 | https://www.genecards.org/cgi-bin/carddisp.pl?gene=NHERF1 |  |
| HACD4 | 3-Hydroxyacyl-CoA Dehydratase 4 | Protein Coding | Q5VWC8 | 40 | GC09M020999 | 3.15159845352173 | https://www.genecards.org/cgi-bin/carddisp.pl?gene=HACD4 |  |
| CD80 | CD80 Molecule | Protein Coding | P33681 | 55 | GC03M119524 | 3.15070748329163 | https://www.genecards.org/cgi-bin/carddisp.pl?gene=CD80 |  |
| FLOT2 | Flotillin 2 | Protein Coding | Q14254 | 51 | GC17M096702 | 3.15007472038269 | https://www.genecards.org/cgi-bin/carddisp.pl?gene=FLOT2 |  |
| IGHMBP2 | Immunoglobulin Mu DNA Binding Protein 2 | Protein Coding | P38935 | 54 | GC11P068903 | 3.14509391784668 | https://www.genecards.org/cgi-bin/carddisp.pl?gene=IGHMBP2 |  |
| LPAR3 | Lysophosphatidic Acid Receptor 3 | Protein Coding | Q9UBY5 | 56 | GC01M084811 | 3.14374041557312 | https://www.genecards.org/cgi-bin/carddisp.pl?gene=LPAR3 |  |
| GCNT1 | Glucosaminyl (N-Acetyl) Transferase 1 | Protein Coding | Q02742 | 51 | GC09P077647 | 3.14337825775146 | https://www.genecards.org/cgi-bin/carddisp.pl?gene=GCNT1 |  |
| LMAN2 | Lectin, Mannose Binding 2 | Protein Coding | Q12907 | 54 | GC05M183282 | 3.14234805107117 | https://www.genecards.org/cgi-bin/carddisp.pl?gene=LMAN2 |  |
| ILKAP | ILK Associated Serine/Threonine Phosphatase | Protein Coding | Q9H0C8 | 48 | GC02M238170 | 3.13383388519287 | https://www.genecards.org/cgi-bin/carddisp.pl?gene=ILKAP |  |
| CNKSR1 | Connector Enhancer Of Kinase Suppressor Of Ras 1 | Protein Coding | Q969H4 | 50 | GC01P076401 | 3.13298749923706 | https://www.genecards.org/cgi-bin/carddisp.pl?gene=CNKSR1 |  |
| ETNK2 | Ethanolamine Kinase 2 | Protein Coding | Q9NVF9 | 50 | GC01M204100 | 3.13266611099243 | https://www.genecards.org/cgi-bin/carddisp.pl?gene=ETNK2 |  |
| RECK | Reversion Inducing Cysteine Rich Protein With Kazal Motifs | Protein Coding | O95980 | 52 | GC09P036036 | 3.13220882415771 | https://www.genecards.org/cgi-bin/carddisp.pl?gene=RECK |  |
| CDK17 | Cyclin Dependent Kinase 17 | Protein Coding | Q00537 | 49 | GC12M096278 | 3.1255476474762 | https://www.genecards.org/cgi-bin/carddisp.pl?gene=CDK17 |  |
| NRP1 | Neuropilin 1 | Protein Coding | O14786 | 61 | GC10M033177 | 3.12409567832947 | https://www.genecards.org/cgi-bin/carddisp.pl?gene=NRP1 |  |
| ARSF | Arylsulfatase F | Protein Coding | P54793 | 48 | GC0XP003040 | 3.12074279785156 | https://www.genecards.org/cgi-bin/carddisp.pl?gene=ARSF |  |
| MIR19A | MicroRNA 19a | RNA Gene |  | 28 | GC13P091748 | 3.12030792236328 | https://www.genecards.org/cgi-bin/carddisp.pl?gene=MIR19A |  |
| GJB1 | Gap Junction Protein Beta 1 | Protein Coding | P08034 | 60 | GC0XP071212 | 3.11739158630371 | https://www.genecards.org/cgi-bin/carddisp.pl?gene=GJB1 |  |
| POLR2E | RNA Polymerase II, I And III Subunit E | Protein Coding | P19388 | 52 | GC19M001086 | 3.11727929115295 | https://www.genecards.org/cgi-bin/carddisp.pl?gene=POLR2E |  |
| MAPK9 | Mitogen-Activated Protein Kinase 9 | Protein Coding | P45984 | 61 | GC05M183397 | 3.11707401275635 | https://www.genecards.org/cgi-bin/carddisp.pl?gene=MAPK9 |  |
| ST6GALNAC1 | ST6 N-Acetylgalactosaminide Alpha-2,6-Sialyltransferase 1 | Protein Coding | Q9NSC7 | 52 | GC17M098087 | 3.11197757720947 | https://www.genecards.org/cgi-bin/carddisp.pl?gene=ST6GALNAC1 |  |
| MPP2 | MAGUK P55 Scaffold Protein 2 | Protein Coding | Q14168 | 47 | GC17M043875 | 3.11108350753784 | https://www.genecards.org/cgi-bin/carddisp.pl?gene=MPP2 |  |
| MAPK11 | Mitogen-Activated Protein Kinase 11 | Protein Coding | Q15759 | 60 | GC22M050263 | 3.08517456054688 | https://www.genecards.org/cgi-bin/carddisp.pl?gene=MAPK11 |  |
| B3GALT2 | Beta-1,3-Galactosyltransferase 2 | Protein Coding | O43825 | 44 | GC01M193147 | 3.08267760276794 | https://www.genecards.org/cgi-bin/carddisp.pl?gene=B3GALT2 |  |
| KCNH7 | Potassium Voltage-Gated Channel Subfamily H Member 7 | Protein Coding | Q9NS40 | 54 | GC02M162371 | 3.07433271408081 | https://www.genecards.org/cgi-bin/carddisp.pl?gene=KCNH7 |  |
| TUBA1B | Tubulin Alpha 1b | Protein Coding | P68363 | 57 | GC12M049127 | 3.05180168151855 | https://www.genecards.org/cgi-bin/carddisp.pl?gene=TUBA1B |  |
| ST6GALNAC2 | ST6 N-Acetylgalactosaminide Alpha-2,6-Sialyltransferase 2 | Protein Coding | Q9UJ37 | 46 | GC17M076565 | 3.05158424377441 | https://www.genecards.org/cgi-bin/carddisp.pl?gene=ST6GALNAC2 |  |
| DIABLO | Diablo IAP-Binding Mitochondrial Protein | Protein Coding | Q9NR28 | 60 | GC12M124699 | 3.0505039691925 | https://www.genecards.org/cgi-bin/carddisp.pl?gene=DIABLO |  |
| CD63 | CD63 Molecule | Protein Coding | P08962 | 56 | GC12M060362 | 3.03976821899414 | https://www.genecards.org/cgi-bin/carddisp.pl?gene=CD63 |  |
| LAPTM4B | Lysosomal Protein Transmembrane 4 Beta | Protein Coding | Q86VI4 | 46 | GC08P097775 | 3.0392918586731 | https://www.genecards.org/cgi-bin/carddisp.pl?gene=LAPTM4B |  |
| RIPK2 | Receptor Interacting Serine/Threonine Kinase 2 | Protein Coding | O43353 | 58 | GC08P090055 | 3.00406408309937 | https://www.genecards.org/cgi-bin/carddisp.pl?gene=RIPK2 |  |
| RPP38 | Ribonuclease P/MRP Subunit P38 | Protein Coding | P78345 | 44 | GC10P015097 | 2.99723672866821 | https://www.genecards.org/cgi-bin/carddisp.pl?gene=RPP38 |  |
| VAMP3 | Vesicle Associated Membrane Protein 3 | Protein Coding | Q15836 | 52 | GC01P075167 | 2.99661874771118 | https://www.genecards.org/cgi-bin/carddisp.pl?gene=VAMP3 |  |
| GP2 | Glycoprotein 2 | Protein Coding | P55259 | 52 | GC16M020309 | 2.99493217468262 | https://www.genecards.org/cgi-bin/carddisp.pl?gene=GP2 |  |
| BAIAP2L1 | BAR/IMD Domain Containing Adaptor Protein 2 Like 1 | Protein Coding | Q9UHR4 | 50 | GC07M098322 | 2.98510789871216 | https://www.genecards.org/cgi-bin/carddisp.pl?gene=BAIAP2L1 |  |
| ETNPPL | Ethanolamine-Phosphate Phospho-Lyase | Protein Coding | Q8TBG4 | 44 | GC04M108742 | 2.97965860366821 | https://www.genecards.org/cgi-bin/carddisp.pl?gene=ETNPPL |  |
| SERINC4 | Serine Incorporator 4 | Protein Coding | A6NH21 | 38 | GC15M043794 | 2.97428607940674 | https://www.genecards.org/cgi-bin/carddisp.pl?gene=SERINC4 |  |
| CCL18 | C-C Motif Chemokine Ligand 18 | Protein Coding | P55774 | 49 | GC17P036064 | 2.9694287776947 | https://www.genecards.org/cgi-bin/carddisp.pl?gene=CCL18 |  |
| CTTN | Cortactin | Protein Coding | Q14247 | 57 | GC11P070398 | 2.95896625518799 | https://www.genecards.org/cgi-bin/carddisp.pl?gene=CTTN |  |
| MSH6 | MutS Homolog 6 | Protein Coding | P52701 | 61 | GC02P047695 | 2.95282459259033 | https://www.genecards.org/cgi-bin/carddisp.pl?gene=MSH6 |  |
| CASP6 | Caspase 6 | Protein Coding | P55212 | 61 | GC04M109688 | 2.94987559318542 | https://www.genecards.org/cgi-bin/carddisp.pl?gene=CASP6 |  |
| ETNK1 | Ethanolamine Kinase 1 | Protein Coding | Q9HBU6 | 51 | GC12P022625 | 2.94871139526367 | https://www.genecards.org/cgi-bin/carddisp.pl?gene=ETNK1 |  |
| PLA2G2D | Phospholipase A2 Group IID | Protein Coding | Q9UNK4 | 47 | GC01M020111 | 2.94610786437988 | https://www.genecards.org/cgi-bin/carddisp.pl?gene=PLA2G2D |  |
| FLOT1 | Flotillin 1 | Protein Coding | O75955 | 54 | GC06M106410 | 2.9454174041748 | https://www.genecards.org/cgi-bin/carddisp.pl?gene=FLOT1 |  |
| SYT9 | Synaptotagmin 9 | Protein Coding | Q86SS6 | 43 | GC11P007238 | 2.9445116519928 | https://www.genecards.org/cgi-bin/carddisp.pl?gene=SYT9 |  |
| EGFL8 | EGF Like Domain Multiple 8 | Protein Coding | Q99944 | 42 | GC06P181917 | 2.9392261505127 | https://www.genecards.org/cgi-bin/carddisp.pl?gene=EGFL8 |  |
| KLF5 | KLF Transcription Factor 5 | Protein Coding | Q13887 | 58 | GC13P073054 | 2.92810130119324 | https://www.genecards.org/cgi-bin/carddisp.pl?gene=KLF5 |  |
| GNRHR | Gonadotropin Releasing Hormone Receptor | Protein Coding | P30968 | 59 | GC04M067737 | 2.92808198928833 | https://www.genecards.org/cgi-bin/carddisp.pl?gene=GNRHR |  |
| MSH2 | MutS Homolog 2 | Protein Coding | P43246 | 61 | GC02P047403 | 2.92758893966675 | https://www.genecards.org/cgi-bin/carddisp.pl?gene=MSH2 |  |
| PTGER4 | Prostaglandin E Receptor 4 | Protein Coding | P35408 | 58 | GC05P040679 | 2.92583751678467 | https://www.genecards.org/cgi-bin/carddisp.pl?gene=PTGER4 |  |
| FHL2 | Four And A Half LIM Domains 2 | Protein Coding | Q14192 | 58 | GC02M105357 | 2.92390108108521 | https://www.genecards.org/cgi-bin/carddisp.pl?gene=FHL2 |  |
| BAK1 | BCL2 Antagonist/Killer 1 | Protein Coding | Q16611 | 57 | GC06M033572 | 2.90907192230225 | https://www.genecards.org/cgi-bin/carddisp.pl?gene=BAK1 |  |
| TNFSF10 | TNF Superfamily Member 10 | Protein Coding | P50591 | 58 | GC03M172505 | 2.89955353736877 | https://www.genecards.org/cgi-bin/carddisp.pl?gene=TNFSF10 |  |
| PARD3 | Par-3 Family Cell Polarity Regulator | Protein Coding | Q8TEW0 | 54 | GC10M037208 | 2.8920521736145 | https://www.genecards.org/cgi-bin/carddisp.pl?gene=PARD3 |  |
| BCL2L11 | BCL2 Like 11 | Protein Coding | O43521 | 58 | GC02P111119 | 2.88929128646851 | https://www.genecards.org/cgi-bin/carddisp.pl?gene=BCL2L11 |  |
| TPCN2 | Two Pore Segment Channel 2 | Protein Coding | Q8NHX9 | 52 | GC11P108053 | 2.8884162902832 | https://www.genecards.org/cgi-bin/carddisp.pl?gene=TPCN2 |  |
| CHM | CHM Rab Escort Protein | Protein Coding | P24386 | 53 | GC0XM085861 | 2.87886667251587 | https://www.genecards.org/cgi-bin/carddisp.pl?gene=CHM |  |
| IFNGR1 | Interferon Gamma Receptor 1 | Protein Coding | P15260 | 64 | GC06M137197 | 2.86614656448364 | https://www.genecards.org/cgi-bin/carddisp.pl?gene=IFNGR1 |  |
| EGR1 | Early Growth Response 1 | Protein Coding | P18146 | 57 | GC05P138465 | 2.84122800827026 | https://www.genecards.org/cgi-bin/carddisp.pl?gene=EGR1 |  |
| A3GALT2 | Alpha 1,3-Galactosyltransferase 2 | Protein Coding | U3KPV4 | 31 | GC01M033306 | 2.83664917945862 | https://www.genecards.org/cgi-bin/carddisp.pl?gene=A3GALT2 |  |
| RAB43 | RAB43, Member RAS Oncogene Family | Protein Coding | Q86YS6 | 45 | GC03M132460 | 2.8343563079834 | https://www.genecards.org/cgi-bin/carddisp.pl?gene=RAB43 |  |
| LASP1 | LIM And SH3 Protein 1 | Protein Coding | Q14847 | 53 | GC17P038869 | 2.8336706161499 | https://www.genecards.org/cgi-bin/carddisp.pl?gene=LASP1 |  |
| MR1 | Major Histocompatibility Complex, Class I-Related | Protein Coding | Q95460 | 52 | GC01P181033 | 2.82640480995178 | https://www.genecards.org/cgi-bin/carddisp.pl?gene=MR1 |  |
| PLEKHA3 | Pleckstrin Homology Domain Containing A3 | Protein Coding | Q9HB20 | 47 | GC02P178480 | 2.81641674041748 | https://www.genecards.org/cgi-bin/carddisp.pl?gene=PLEKHA3 |  |
| PXN | Paxillin | Protein Coding | P49023 | 58 | GC12M120210 | 2.81075692176819 | https://www.genecards.org/cgi-bin/carddisp.pl?gene=PXN |  |
| PCYT1B | Phosphate Cytidylyltransferase 1B, Choline | Protein Coding | Q9Y5K3 | 53 | GC0XM024558 | 2.80687737464905 | https://www.genecards.org/cgi-bin/carddisp.pl?gene=PCYT1B |  |
| S1PR4 | Sphingosine-1-Phosphate Receptor 4 | Protein Coding | O95977 | 51 | GC19P151478 | 2.80535697937012 | https://www.genecards.org/cgi-bin/carddisp.pl?gene=S1PR4 |  |
| KYAT3 | Kynurenine Aminotransferase 3 | Protein Coding | Q6YP21 | 45 | GC01M088921 | 2.8027331829071 | https://www.genecards.org/cgi-bin/carddisp.pl?gene=KYAT3 |  |
| ITGA9 | Integrin Subunit Alpha 9 | Protein Coding | Q13797 | 55 | GC03P037468 | 2.80110740661621 | https://www.genecards.org/cgi-bin/carddisp.pl?gene=ITGA9 |  |
| EEA1 | Early Endosome Antigen 1 | Protein Coding | Q15075 | 55 | GC12M092770 | 2.7971351146698 | https://www.genecards.org/cgi-bin/carddisp.pl?gene=EEA1 |  |
| ATP5MGL | ATP Synthase Membrane Subunit G Like | Protein Coding | Q7Z4Y8 | 36 | GC22M086165 | 2.79466724395752 | https://www.genecards.org/cgi-bin/carddisp.pl?gene=ATP5MGL |  |
| C12orf43 | Chromosome 12 Open Reading Frame 43 | Protein Coding | Q96C57 | 44 | GC12M121000 | 2.79403448104858 | https://www.genecards.org/cgi-bin/carddisp.pl?gene=C12orf43 |  |
| COPE | COPI Coat Complex Subunit Epsilon | Protein Coding | O14579 | 51 | GC19M018899 | 2.78909969329834 | https://www.genecards.org/cgi-bin/carddisp.pl?gene=COPE |  |
| SLC13A1 | Solute Carrier Family 13 Member 1 | Protein Coding | Q9BZW2 | 48 | GC07M123113 | 2.77474689483643 | https://www.genecards.org/cgi-bin/carddisp.pl?gene=SLC13A1 |  |
| SNAP23 | Synaptosome Associated Protein 23 | Protein Coding | O00161 | 55 | GC15P042491 | 2.77215266227722 | https://www.genecards.org/cgi-bin/carddisp.pl?gene=SNAP23 |  |
| GALNT16 | Polypeptide N-Acetylgalactosaminyltransferase 16 | Protein Coding | Q8N428 | 49 | GC14P069259 | 2.76712536811829 | https://www.genecards.org/cgi-bin/carddisp.pl?gene=GALNT16 |  |
| LGALS4 | Galectin 4 | Protein Coding | P56470 | 51 | GC19M108718 | 2.7578911781311 | https://www.genecards.org/cgi-bin/carddisp.pl?gene=LGALS4 |  |
| ERP44 | Endoplasmic Reticulum Protein 44 | Protein Coding | Q9BS26 | 51 | GC09M099979 | 2.75503492355347 | https://www.genecards.org/cgi-bin/carddisp.pl?gene=ERP44 |  |
| BASP1 | Brain Abundant Membrane Attached Signal Protein 1 | Protein Coding | P80723 | 48 | GC05P017065 | 2.75213670730591 | https://www.genecards.org/cgi-bin/carddisp.pl?gene=BASP1 |  |
| STOM | Stomatin | Protein Coding | P27105 | 54 | GC09M121338 | 2.74651598930359 | https://www.genecards.org/cgi-bin/carddisp.pl?gene=STOM |  |
| SET | SET Nuclear Proto-Oncogene | Protein Coding | Q01105 | 56 | GC09P152894 | 2.74550342559814 | https://www.genecards.org/cgi-bin/carddisp.pl?gene=SET |  |
| ACYP1 | Acylphosphatase 1 | Protein Coding | P07311 | 49 | GC14M075053 | 2.74479627609253 | https://www.genecards.org/cgi-bin/carddisp.pl?gene=ACYP1 |  |
| DUSP1 | Dual Specificity Phosphatase 1 | Protein Coding | P28562 | 59 | GC05M172768 | 2.74448585510254 | https://www.genecards.org/cgi-bin/carddisp.pl?gene=DUSP1 |  |
| MIR379 | MicroRNA 379 | RNA Gene |  | 27 | GC14P121370 | 2.73970556259155 | https://www.genecards.org/cgi-bin/carddisp.pl?gene=MIR379 |  |
| PIK3C2B | Phosphatidylinositol-4-Phosphate 3-Kinase Catalytic Subunit Type 2 Beta | Protein Coding | O00750 | 58 | GC01M204422 | 2.73767471313477 | https://www.genecards.org/cgi-bin/carddisp.pl?gene=PIK3C2B |  |
| TENT4A | Terminal Nucleotidyltransferase 4A | Protein Coding | Q5XG87 | 47 | GC05P006713 | 2.73636627197266 | https://www.genecards.org/cgi-bin/carddisp.pl?gene=TENT4A |  |
| S100A6 | S100 Calcium Binding Protein A6 | Protein Coding | P06703 | 54 | GC01M167362 | 2.73213243484497 | https://www.genecards.org/cgi-bin/carddisp.pl?gene=S100A6 |  |
| MAPK13 | Mitogen-Activated Protein Kinase 13 | Protein Coding | O15264 | 60 | GC06P182013 | 2.72467160224915 | https://www.genecards.org/cgi-bin/carddisp.pl?gene=MAPK13 |  |
| G0S2 | G0/G1 Switch 2 | Protein Coding | P27469 | 42 | GC01P209675 | 2.7155065536499 | https://www.genecards.org/cgi-bin/carddisp.pl?gene=G0S2 |  |
| TIMP3 | TIMP Metallopeptidase Inhibitor 3 | Protein Coding | P35625 | 57 | GC22P092006 | 2.71503400802612 | https://www.genecards.org/cgi-bin/carddisp.pl?gene=TIMP3 |  |
| SELPLG | Selectin P Ligand | Protein Coding | Q14242 | 56 | GC12M108621 | 2.71366143226624 | https://www.genecards.org/cgi-bin/carddisp.pl?gene=SELPLG |  |
| NHLRC2 | NHL Repeat Containing 2 | Protein Coding | Q8NBF2 | 47 | GC10P113854 | 2.71330547332764 | https://www.genecards.org/cgi-bin/carddisp.pl?gene=NHLRC2 |  |
| RAB9A | RAB9A, Member RAS Oncogene Family | Protein Coding | P51151 | 49 | GC0XP013930 | 2.70586681365967 | https://www.genecards.org/cgi-bin/carddisp.pl?gene=RAB9A |  |
| STARD3NL | STARD3 N-Terminal Like | Protein Coding | O95772 | 42 | GC07P038179 | 2.70370244979858 | https://www.genecards.org/cgi-bin/carddisp.pl?gene=STARD3NL |  |
| ILVBL | IlvB Acetolactate Synthase Like | Protein Coding | A1L0T0 | 47 | GC19M108287 | 2.69366049766541 | https://www.genecards.org/cgi-bin/carddisp.pl?gene=ILVBL |  |
| CASP7 | Caspase 7 | Protein Coding | P55210 | 62 | GC10P113679 | 2.69029331207275 | https://www.genecards.org/cgi-bin/carddisp.pl?gene=CASP7 |  |
| AP2A2 | Adaptor Related Protein Complex 2 Subunit Alpha 2 | Protein Coding | O94973 | 50 | GC11P000924 | 2.68921136856079 | https://www.genecards.org/cgi-bin/carddisp.pl?gene=AP2A2 |  |
| MUC5AC | Mucin 5AC, Oligomeric Mucus/Gel-Forming | Protein Coding | P98088 | 51 | GC11P020281 | 2.67589974403381 | https://www.genecards.org/cgi-bin/carddisp.pl?gene=MUC5AC |  |
| MAPK7 | Mitogen-Activated Protein Kinase 7 | Protein Coding | Q13164 | 61 | GC17P154121 | 2.67485737800598 | https://www.genecards.org/cgi-bin/carddisp.pl?gene=MAPK7 |  |
| TLCD3B | TLC Domain Containing 3B | Protein Coding | Q71RH2 | 45 | GC16M051071 | 2.67202925682068 | https://www.genecards.org/cgi-bin/carddisp.pl?gene=TLCD3B |  |
| THY1 | Thy-1 Cell Surface Antigen | Protein Coding | P04216 | 56 | GC11M140671 | 2.66840100288391 | https://www.genecards.org/cgi-bin/carddisp.pl?gene=THY1 |  |
| AP2B1 | Adaptor Related Protein Complex 2 Subunit Beta 1 | Protein Coding | P63010 | 54 | GC17P035578 | 2.66364145278931 | https://www.genecards.org/cgi-bin/carddisp.pl?gene=AP2B1 |  |
| PTDSS2 | Phosphatidylserine Synthase 2 | Protein Coding | Q9BVG9 | 47 | GC11P000448 | 2.66020250320435 | https://www.genecards.org/cgi-bin/carddisp.pl?gene=PTDSS2 |  |
| OPRD1 | Opioid Receptor Delta 1 | Protein Coding | P41143 | 55 | GC01P028812 | 2.65782451629639 | https://www.genecards.org/cgi-bin/carddisp.pl?gene=OPRD1 |  |
| NOD1 | Nucleotide Binding Oligomerization Domain Containing 1 | Protein Coding | Q9Y239 | 55 | GC07M030424 | 2.65485692024231 | https://www.genecards.org/cgi-bin/carddisp.pl?gene=NOD1 |  |
| OSM | Oncostatin M | Protein Coding | P13725 | 55 | GC22M030262 | 2.65354299545288 | https://www.genecards.org/cgi-bin/carddisp.pl?gene=OSM |  |
| MPIG6B | Megakaryocyte And Platelet Inhibitory Receptor G6b | Protein Coding | O95866 | 51 | GC06P189252 | 2.6506814956665 | https://www.genecards.org/cgi-bin/carddisp.pl?gene=MPIG6B |  |
| PA2G4P2 | Proliferation-Associated 2G4 Pseudogene 2 | Pseudogene |  | 10 | GC20P012380 | 2.64243268966675 | https://www.genecards.org/cgi-bin/carddisp.pl?gene=PA2G4P2 |  |
| APOA1-AS | APOA1 Antisense RNA | RNA Gene |  | 23 | GC11P116836 | 2.63207530975342 | https://www.genecards.org/cgi-bin/carddisp.pl?gene=APOA1-AS |  |
| TIMELESS | Timeless Circadian Regulator | Protein Coding | Q9UNS1 | 52 | GC12M056416 | 2.6227343082428 | https://www.genecards.org/cgi-bin/carddisp.pl?gene=TIMELESS |  |
| SDSL | Serine Dehydratase Like | Protein Coding | Q96GA7 | 48 | GC12P113422 | 2.60552763938904 | https://www.genecards.org/cgi-bin/carddisp.pl?gene=SDSL |  |
| FCGR1A | Fc Gamma Receptor Ia | Protein Coding | P12314 | 55 | GC01P176446 | 2.59173679351807 | https://www.genecards.org/cgi-bin/carddisp.pl?gene=FCGR1A |  |
| SH3TC2 | SH3 Domain And Tetratricopeptide Repeats 2 | Protein Coding | Q8TF17 | 50 | GC05M148923 | 2.59069323539734 | https://www.genecards.org/cgi-bin/carddisp.pl?gene=SH3TC2 |  |
| TRPM1 | Transient Receptor Potential Cation Channel Subfamily M Member 1 | Protein Coding | Q7Z4N2 | 56 | GC15M031001 | 2.59046673774719 | https://www.genecards.org/cgi-bin/carddisp.pl?gene=TRPM1 |  |
| PNPLA5 | Patatin Like Domain 5, Triacylglycerol Lipase | Protein Coding | Q7Z6Z6 | 44 | GC22M043879 | 2.57858538627625 | https://www.genecards.org/cgi-bin/carddisp.pl?gene=PNPLA5 |  |
| CCNB1 | Cyclin B1 | Protein Coding | P14635 | 60 | GC05P069167 | 2.54704904556274 | https://www.genecards.org/cgi-bin/carddisp.pl?gene=CCNB1 |  |
| TLR7 | Toll Like Receptor 7 | Protein Coding | Q9NYK1 | 61 | GC0XP012867 | 2.54319286346436 | https://www.genecards.org/cgi-bin/carddisp.pl?gene=TLR7 |  |
| ANXA6 | Annexin A6 | Protein Coding | P08133 | 55 | GC05M151100 | 2.53814744949341 | https://www.genecards.org/cgi-bin/carddisp.pl?gene=ANXA6 |  |
| PTGER2 | Prostaglandin E Receptor 2 | Protein Coding | P43116 | 61 | GC14P052314 | 2.53557300567627 | https://www.genecards.org/cgi-bin/carddisp.pl?gene=PTGER2 |  |
| MAP2K5 | Mitogen-Activated Protein Kinase Kinase 5 | Protein Coding | Q13163 | 57 | GC15P193378 | 2.52924585342407 | https://www.genecards.org/cgi-bin/carddisp.pl?gene=MAP2K5 |  |
| USP1 | Ubiquitin Specific Peptidase 1 | Protein Coding | O94782 | 57 | GC01P062436 | 2.52110505104065 | https://www.genecards.org/cgi-bin/carddisp.pl?gene=USP1 |  |
| MAPK12 | Mitogen-Activated Protein Kinase 12 | Protein Coding | P53778 | 60 | GC22M086274 | 2.51363468170166 | https://www.genecards.org/cgi-bin/carddisp.pl?gene=MAPK12 |  |
| RAB32 | RAB32, Member RAS Oncogene Family | Protein Coding | Q13637 | 49 | GC06P146543 | 2.49762487411499 | https://www.genecards.org/cgi-bin/carddisp.pl?gene=RAB32 |  |
| ABCC10 | ATP Binding Cassette Subfamily C Member 10 | Protein Coding | Q5T3U5 | 51 | GC06P043427 | 2.49233746528625 | https://www.genecards.org/cgi-bin/carddisp.pl?gene=ABCC10 |  |
| CD79B | CD79b Molecule | Protein Coding | P40259 | 59 | GC17M063928 | 2.4844765663147 | https://www.genecards.org/cgi-bin/carddisp.pl?gene=CD79B |  |
| CD86 | CD86 Molecule | Protein Coding | P42081 | 57 | GC03P122055 | 2.48328304290771 | https://www.genecards.org/cgi-bin/carddisp.pl?gene=CD86 |  |
| PRRC2A | Proline Rich Coiled-Coil 2A | Protein Coding | P48634 | 45 | GC06P181895 | 2.4697368144989 | https://www.genecards.org/cgi-bin/carddisp.pl?gene=PRRC2A |  |
| P2RY1 | Purinergic Receptor P2Y1 | Protein Coding | P47900 | 59 | GC03P152835 | 2.45783853530884 | https://www.genecards.org/cgi-bin/carddisp.pl?gene=P2RY1 |  |
| MSH5 | MutS Homolog 5 | Protein Coding | O43196 | 54 | GC06P181906 | 2.45674228668213 | https://www.genecards.org/cgi-bin/carddisp.pl?gene=MSH5 |  |
| IVL | Involucrin | Protein Coding | P07476 | 51 | GC01P176624 | 2.45172381401062 | https://www.genecards.org/cgi-bin/carddisp.pl?gene=IVL |  |
| APOL6 | Apolipoprotein L6 | Protein Coding | Q9BWW8 | 42 | GC22P035648 | 2.45104455947876 | https://www.genecards.org/cgi-bin/carddisp.pl?gene=APOL6 |  |
| COPZ1 | COPI Coat Complex Subunit Zeta 1 | Protein Coding | P61923 | 47 | GC12P054301 | 2.44944930076599 | https://www.genecards.org/cgi-bin/carddisp.pl?gene=COPZ1 |  |
| NSMAF | Neutral Sphingomyelinase Activation Associated Factor | Protein Coding | Q92636 | 51 | GC08M058569 | 2.44773626327515 | https://www.genecards.org/cgi-bin/carddisp.pl?gene=NSMAF |  |
| TRPM2 | Transient Receptor Potential Cation Channel Subfamily M Member 2 | Protein Coding | O94759 | 53 | GC21P044350 | 2.43352890014648 | https://www.genecards.org/cgi-bin/carddisp.pl?gene=TRPM2 |  |
| SNHG14 | Small Nucleolar RNA Host Gene 14 | RNA Gene |  | 25 | GC15P192315 | 2.43140435218811 | https://www.genecards.org/cgi-bin/carddisp.pl?gene=SNHG14 |  |
| HLA-G | Major Histocompatibility Complex, Class I, G | Protein Coding | P17693 | 57 | GC06P181845 | 2.42660236358643 | https://www.genecards.org/cgi-bin/carddisp.pl?gene=HLA-G |  |
| POM121 | POM121 Transmembrane Nucleoporin | Protein Coding | Q96HA1 | 46 | GC07P072879 | 2.41796731948853 | https://www.genecards.org/cgi-bin/carddisp.pl?gene=POM121 |  |
| TOLLIP | Toll Interacting Protein | Protein Coding | Q9H0E2 | 56 | GC11M001274 | 2.41569232940674 | https://www.genecards.org/cgi-bin/carddisp.pl?gene=TOLLIP |  |
| PMP22 | Peripheral Myelin Protein 22 | Protein Coding | Q01453 | 56 | GC17M015229 | 2.41214561462402 | https://www.genecards.org/cgi-bin/carddisp.pl?gene=PMP22 |  |
| PHYKPL | 5-Phosphohydroxy-L-Lysine Phospho-Lyase | Protein Coding | Q8IUZ5 | 48 | GC05M178207 | 2.40726208686829 | https://www.genecards.org/cgi-bin/carddisp.pl?gene=PHYKPL |  |
| CD1A | CD1a Molecule | Protein Coding | P06126 | 52 | GC01P176906 | 2.39897179603577 | https://www.genecards.org/cgi-bin/carddisp.pl?gene=CD1A |  |
| TMEM229B | Transmembrane Protein 229B | Protein Coding | Q8NBD8 | 37 | GC14M067447 | 2.398029089 | https://www.genecards.org/cgi-bin/carddisp.pl?gene=TMEM229B |  |
| LORICRIN | Loricrin Cornified Envelope Precursor Protein | Protein Coding | P23490 | 47 | GC01P176636 | 2.39472484588623 | https://www.genecards.org/cgi-bin/carddisp.pl?gene=LORICRIN |  |
| KLF12 | KLF Transcription Factor 12 | Protein Coding | Q9Y4X4 | 48 | GC13M073686 | 2.38068294525146 | https://www.genecards.org/cgi-bin/carddisp.pl?gene=KLF12 |  |
| GPRC6A | G Protein-Coupled Receptor Class C Group 6 Member A | Protein Coding | Q5T6X5 | 48 | GC06M116793 | 2.36574745178223 | https://www.genecards.org/cgi-bin/carddisp.pl?gene=GPRC6A |  |
| NOTCH4 | Notch Receptor 4 | Protein Coding | Q99466 | 60 | GC06M106508 | 2.36383938789368 | https://www.genecards.org/cgi-bin/carddisp.pl?gene=NOTCH4 |  |
| LPAR2 | Lysophosphatidic Acid Receptor 2 | Protein Coding | Q9HBW0 | 54 | GC19M108446 | 2.35855865478516 | https://www.genecards.org/cgi-bin/carddisp.pl?gene=LPAR2 |  |
| POLDIP3 | DNA Polymerase Delta Interacting Protein 3 | Protein Coding | Q9BY77 | 50 | GC22M042583 | 2.35816049575806 | https://www.genecards.org/cgi-bin/carddisp.pl?gene=POLDIP3 |  |
| CORIN | Corin, Serine Peptidase | Protein Coding | Q9Y5Q5 | 57 | GC04M047596 | 2.35751438140869 | https://www.genecards.org/cgi-bin/carddisp.pl?gene=CORIN |  |
| LOC130063376 | ATAC-STARR-Seq Lymphoblastoid Silent Region 9986 | Functional Element |  | 7 | GC19P158095 | 2.35011029243469 | https://www.genecards.org/cgi-bin/carddisp.pl?gene=LOC130063376 |  |
| MIR495 | MicroRNA 495 | RNA Gene |  | 25 | GC14P121385 | 2.34533023834229 | https://www.genecards.org/cgi-bin/carddisp.pl?gene=MIR495 |  |
| CD209 | CD209 Molecule | Protein Coding | Q9NNX6 | 56 | GC19M007739 | 2.33265542984009 | https://www.genecards.org/cgi-bin/carddisp.pl?gene=CD209 |  |
| COPG2 | COPI Coat Complex Subunit Gamma 2 | Protein Coding | Q9UBF2 | 49 | GC07M130506 | 2.33112812042236 | https://www.genecards.org/cgi-bin/carddisp.pl?gene=COPG2 |  |
| LSR | Lipolysis Stimulated Lipoprotein Receptor | Protein Coding | Q86X29 | 51 | GC19P152384 | 2.31776809692383 | https://www.genecards.org/cgi-bin/carddisp.pl?gene=LSR |  |
| CDH8 | Cadherin 8 | Protein Coding | P55286 | 55 | GC16M061647 | 2.31031107902527 | https://www.genecards.org/cgi-bin/carddisp.pl?gene=CDH8 |  |
| SERINC2 | Serine Incorporator 2 | Protein Coding | Q96SA4 | 45 | GC01P031409 | 2.30567860603333 | https://www.genecards.org/cgi-bin/carddisp.pl?gene=SERINC2 |  |
| MAP2K3 | Mitogen-Activated Protein Kinase Kinase 3 | Protein Coding | P46734 | 61 | GC17P154189 | 2.3008668422699 | https://www.genecards.org/cgi-bin/carddisp.pl?gene=MAP2K3 |  |
| APOL2 | Apolipoprotein L2 | Protein Coding | Q9BQE5 | 49 | GC22M036226 | 2.25629806518555 | https://www.genecards.org/cgi-bin/carddisp.pl?gene=APOL2 |  |
| NPEPPS | Aminopeptidase Puromycin Sensitive | Protein Coding | P55786 | 55 | GC17P047522 | 2.25347328186035 | https://www.genecards.org/cgi-bin/carddisp.pl?gene=NPEPPS |  |
| EIF2S2P1 | Eukaryotic Translation Initiation Factor 2 Subunit 2 Beta Pseudogene 1 | Pseudogene |  | 9 | GC14P063734 | 2.239177942 | https://www.genecards.org/cgi-bin/carddisp.pl?gene=EIF2S2P1 |  |
| RRP7A | Ribosomal RNA Processing 7 Homolog A | Protein Coding | Q9Y3A4 | 50 | GC22M086162 | 2.23803973197937 | https://www.genecards.org/cgi-bin/carddisp.pl?gene=RRP7A |  |
| PAQR4 | Progestin And AdipoQ Receptor Family Member 4 | Protein Coding | Q8N4S7 | 43 | GC16P002969 | 2.23246192932129 | https://www.genecards.org/cgi-bin/carddisp.pl?gene=PAQR4 |  |
| ZMYND8 | Zinc Finger MYND-Type Containing 8 | Protein Coding | Q9ULU4 | 52 | GC20M047209 | 2.23166441917419 | https://www.genecards.org/cgi-bin/carddisp.pl?gene=ZMYND8 |  |
| APOL4 | Apolipoprotein L4 | Protein Coding | Q9BPW4 | 44 | GC22M086096 | 2.23123836517334 | https://www.genecards.org/cgi-bin/carddisp.pl?gene=APOL4 |  |
| RAB30 | RAB30, Member RAS Oncogene Family | Protein Coding | Q15771 | 42 | GC11M082973 | 2.22505235671997 | https://www.genecards.org/cgi-bin/carddisp.pl?gene=RAB30 |  |
| NFASC | Neurofascin | Protein Coding | O94856 | 56 | GC01P204828 | 2.22357654571533 | https://www.genecards.org/cgi-bin/carddisp.pl?gene=NFASC |  |
| NTRK3 | Neurotrophic Receptor Tyrosine Kinase 3 | Protein Coding | Q16288 | 65 | GC15M087859 | 2.22104907035828 | https://www.genecards.org/cgi-bin/carddisp.pl?gene=NTRK3 |  |
| DAB2 | DAB Adaptor Protein 2 | Protein Coding | P98082 | 55 | GC05M039371 | 2.21189141273499 | https://www.genecards.org/cgi-bin/carddisp.pl?gene=DAB2 |  |
| APOC4-APOC2 | APOC4-APOC2 Readthrough (NMD Candidate) | RNA Gene |  | 20 | GC19P044942 | 2.20645070075989 | https://www.genecards.org/cgi-bin/carddisp.pl?gene=APOC4-APOC2 |  |
| GCAT | Glycine C-Acetyltransferase | Protein Coding | O75600 | 50 | GC22P037807 | 2.18585634231567 | https://www.genecards.org/cgi-bin/carddisp.pl?gene=GCAT |  |
| ERG | ETS Transcription Factor ERG | Protein Coding | P11308 | 59 | GC21M038367 | 2.18528413772583 | https://www.genecards.org/cgi-bin/carddisp.pl?gene=ERG |  |
| PRX | Periaxin | Protein Coding | Q9BXM0 | 54 | GC19M040393 | 2.17917442321777 | https://www.genecards.org/cgi-bin/carddisp.pl?gene=PRX |  |
| OR8I2 | Olfactory Receptor Family 8 Subfamily I Member 2 | Protein Coding | Q8N0Y5 | 37 | GC11P056093 | 2.17536330223083 | https://www.genecards.org/cgi-bin/carddisp.pl?gene=OR8I2 |  |
| ZNF600 | Zinc Finger Protein 600 | Protein Coding | Q6ZNG1 | 35 | GC19M052749 | 2.17536330223083 | https://www.genecards.org/cgi-bin/carddisp.pl?gene=ZNF600 |  |
| PATJ | PATJ Crumbs Cell Polarity Complex Component | Protein Coding | Q8NI35 | 47 | GC01P078703 | 2.15142607688904 | https://www.genecards.org/cgi-bin/carddisp.pl?gene=PATJ |  |
| ATP10A | ATPase Phospholipid Transporting 10A (Putative) | Protein Coding | O60312 | 54 | GC15M047499 | 2.15025949478149 | https://www.genecards.org/cgi-bin/carddisp.pl?gene=ATP10A |  |
| BTNL2 | Butyrophilin Like 2 | Protein Coding | Q9UIR0 | 52 | GC06M032393 | 2.14744734764099 | https://www.genecards.org/cgi-bin/carddisp.pl?gene=BTNL2 |  |
| STOML2 | Stomatin Like 2 | Protein Coding | Q9UJZ1 | 49 | GC09M035099 | 2.12746000289917 | https://www.genecards.org/cgi-bin/carddisp.pl?gene=STOML2 |  |
| VPS54 | VPS54 Subunit Of GARP Complex | Protein Coding | Q9P1Q0 | 49 | GC02M063892 | 2.12720441818237 | https://www.genecards.org/cgi-bin/carddisp.pl?gene=VPS54 |  |
| CDC20 | Cell Division Cycle 20 | Protein Coding | Q12834 | 58 | GC01P043358 | 2.12695860862732 | https://www.genecards.org/cgi-bin/carddisp.pl?gene=CDC20 |  |
| KRT1 | Keratin 1 | Protein Coding | P04264 | 60 | GC12M052674 | 2.1266942024231 | https://www.genecards.org/cgi-bin/carddisp.pl?gene=KRT1 |  |
| TPCN1 | Two Pore Segment Channel 1 | Protein Coding | Q9ULQ1 | 46 | GC12P113221 | 2.12399435043335 | https://www.genecards.org/cgi-bin/carddisp.pl?gene=TPCN1 |  |
| CYSLTR2 | Cysteinyl Leukotriene Receptor 2 | Protein Coding | Q9NS75 | 58 | GC13P048653 | 2.12335157394409 | https://www.genecards.org/cgi-bin/carddisp.pl?gene=CYSLTR2 |  |
| CLTA | Clathrin Light Chain A | Protein Coding | P09496 | 48 | GC09P036190 | 2.12277793884277 | https://www.genecards.org/cgi-bin/carddisp.pl?gene=CLTA |  |
| ADAT1 | Adenosine Deaminase TRNA Specific 1 | Protein Coding | Q9BUB4 | 51 | GC16M075596 | 2.12260842323303 | https://www.genecards.org/cgi-bin/carddisp.pl?gene=ADAT1 |  |
| F11 | Coagulation Factor XI | Protein Coding | P03951 | 60 | GC04P186447 | 2.12103223800659 | https://www.genecards.org/cgi-bin/carddisp.pl?gene=F11 |  |
| ITGAL | Integrin Subunit Alpha L | Protein Coding | P20701 | 60 | GC16P030472 | 2.11636853218079 | https://www.genecards.org/cgi-bin/carddisp.pl?gene=ITGAL |  |
| RAB4B | RAB4B, Member RAS Oncogene Family | Protein Coding | P61018 | 42 | GC19P152544 | 2.11257433891296 | https://www.genecards.org/cgi-bin/carddisp.pl?gene=RAB4B |  |
| ZPBP2 | Zona Pellucida Binding Protein 2 | Protein Coding | Q6X784 | 43 | GC17P154755 | 2.11254549026489 | https://www.genecards.org/cgi-bin/carddisp.pl?gene=ZPBP2 |  |
| MIR369 | MicroRNA 369 | RNA Gene |  | 28 | GC14P121364 | 2.10597991943359 | https://www.genecards.org/cgi-bin/carddisp.pl?gene=MIR369 |  |
| MYO1C | Myosin IC | Protein Coding | O00159 | 53 | GC17M001464 | 2.10518264770508 | https://www.genecards.org/cgi-bin/carddisp.pl?gene=MYO1C |  |
| TLR1 | Toll Like Receptor 1 | Protein Coding | Q15399 | 61 | GC04M038793 | 2.10056352615356 | https://www.genecards.org/cgi-bin/carddisp.pl?gene=TLR1 |  |
| SEC14L1 | SEC14 Like Lipid Binding 1 | Protein Coding | Q92503 | 47 | GC17P155648 | 2.09406137466431 | https://www.genecards.org/cgi-bin/carddisp.pl?gene=SEC14L1 |  |
| HOTAIRM1 | HOXA Transcript Antisense RNA, Myeloid-Specific 1 | RNA Gene |  | 28 | GC07P027095 | 2.09229516983032 | https://www.genecards.org/cgi-bin/carddisp.pl?gene=HOTAIRM1 |  |
| PAWR | Pro-Apoptotic WT1 Regulator | Protein Coding | Q96IZ0 | 54 | GC12M079574 | 2.08964824676514 | https://www.genecards.org/cgi-bin/carddisp.pl?gene=PAWR |  |
| SAG | S-Antigen Visual Arrestin | Protein Coding | P10523 | 58 | GC02P235069 | 2.07863259315491 | https://www.genecards.org/cgi-bin/carddisp.pl?gene=SAG |  |
| APH1A | Aph-1 Homolog A, Gamma-Secretase Subunit | Protein Coding | Q96BI3 | 55 | GC01M150265 | 2.07224559783936 | https://www.genecards.org/cgi-bin/carddisp.pl?gene=APH1A |  |
| CASP2 | Caspase 2 | Protein Coding | P42575 | 61 | GC07P165863 | 2.0575098991394 | https://www.genecards.org/cgi-bin/carddisp.pl?gene=CASP2 |  |
| OSBPL11 | Oxysterol Binding Protein Like 11 | Protein Coding | Q9BXB4 | 46 | GC03M125529 | 2.04385495185852 | https://www.genecards.org/cgi-bin/carddisp.pl?gene=OSBPL11 |  |
| HTR7 | 5-Hydroxytryptamine Receptor 7 | Protein Coding | P34969 | 58 | GC10M090740 | 2.03624320030212 | https://www.genecards.org/cgi-bin/carddisp.pl?gene=HTR7 |  |
| H4C8 | H4 Clustered Histone 8 | Protein Coding | P62805 | 44 | GC06M109209 | 2.03307247161865 | https://www.genecards.org/cgi-bin/carddisp.pl?gene=H4C8 |  |
| AOAH | Acyloxyacyl Hydrolase | Protein Coding | P28039 | 51 | GC07M036519 | 2.0261504650116 | https://www.genecards.org/cgi-bin/carddisp.pl?gene=AOAH |  |
| COPZ2 | COPI Coat Complex Subunit Zeta 2 | Protein Coding | Q9P299 | 41 | GC17M048026 | 2.01845455169678 | https://www.genecards.org/cgi-bin/carddisp.pl?gene=COPZ2 |  |
| APOL5 | Apolipoprotein L5 | Protein Coding | Q9BWW9 | 38 | GC22P092047 | 2.00053691864014 | https://www.genecards.org/cgi-bin/carddisp.pl?gene=APOL5 |  |
| SV2B | Synaptic Vesicle Glycoprotein 2B | Protein Coding | Q7L1I2 | 52 | GC15P091099 | 1.997234583 | https://www.genecards.org/cgi-bin/carddisp.pl?gene=SV2B |  |
| GJD2 | Gap Junction Protein Delta 2 | Protein Coding | Q9UKL4 | 53 | GC15M034751 | 1.99702155590057 | https://www.genecards.org/cgi-bin/carddisp.pl?gene=GJD2 |  |
| NTN1 | Netrin 1 | Protein Coding | O95631 | 58 | GC17P153828 | 1.98621129989624 | https://www.genecards.org/cgi-bin/carddisp.pl?gene=NTN1 |  |
| S100A10 | S100 Calcium Binding Protein A10 | Protein Coding | P60903 | 55 | GC01M167322 | 1.98456490039825 | https://www.genecards.org/cgi-bin/carddisp.pl?gene=S100A10 |  |
| RNU1-4 | RNA, U1 Small Nuclear 4 | RNA Gene |  | 14 | GC01P016740 | 1.98399031162262 | https://www.genecards.org/cgi-bin/carddisp.pl?gene=RNU1-4 |  |
| KRT17 | Keratin 17 | Protein Coding | Q04695 | 58 | GC17M041619 | 1.97407972812653 | https://www.genecards.org/cgi-bin/carddisp.pl?gene=KRT17 |  |
| DAOA-AS1 | DAOA Antisense RNA 1 | RNA Gene |  | 24 | GC13M105467 | 1.97266066074371 | https://www.genecards.org/cgi-bin/carddisp.pl?gene=DAOA-AS1 |  |
| KRT5 | Keratin 5 | Protein Coding | P13647 | 58 | GC12M052514 | 1.96933352947235 | https://www.genecards.org/cgi-bin/carddisp.pl?gene=KRT5 |  |
| TRAM1 | Translocation Associated Membrane Protein 1 | Protein Coding | Q15629 | 48 | GC08M070573 | 1.96813535690308 | https://www.genecards.org/cgi-bin/carddisp.pl?gene=TRAM1 |  |
| MIR301B | MicroRNA 301b | RNA Gene |  | 18 | GC22P091622 | 1.96532022953033 | https://www.genecards.org/cgi-bin/carddisp.pl?gene=MIR301B |  |
| MCOLN2 | Mucolipin TRP Cation Channel 2 | Protein Coding | Q8IZK6 | 47 | GC01M084925 | 1.96102118492126 | https://www.genecards.org/cgi-bin/carddisp.pl?gene=MCOLN2 |  |
| CNRIP1 | Cannabinoid Receptor Interacting Protein 1 | Protein Coding | Q96F85 | 46 | GC02M068284 | 1.94997096061707 | https://www.genecards.org/cgi-bin/carddisp.pl?gene=CNRIP1 |  |
| SMPDL3B | Sphingomyelin Phosphodiesterase Acid Like 3B | Protein Coding | Q92485 | 48 | GC01P076530 | 1.94844734668732 | https://www.genecards.org/cgi-bin/carddisp.pl?gene=SMPDL3B |  |
| HCK | HCK Proto-Oncogene, Src Family Tyrosine Kinase | Protein Coding | P08631 | 62 | GC20P032052 | 1.94662356376648 | https://www.genecards.org/cgi-bin/carddisp.pl?gene=HCK |  |
| LRRC32 | Leucine Rich Repeat Containing 32 | Protein Coding | Q14392 | 51 | GC11M076657 | 1.9417051076889 | https://www.genecards.org/cgi-bin/carddisp.pl?gene=LRRC32 |  |
| PTPN3 | Protein Tyrosine Phosphatase Non-Receptor Type 3 | Protein Coding | P26045 | 55 | GC09M109375 | 1.9388028383255 | https://www.genecards.org/cgi-bin/carddisp.pl?gene=PTPN3 |  |
| PDXDC2P | Pyridoxal Dependent Decarboxylase Domain Containing 2, Pseudogene | Pseudogene | Q6P474 | 24 | GC16M075095 | 1.93588745594025 | https://www.genecards.org/cgi-bin/carddisp.pl?gene=PDXDC2P |  |
| STX4 | Syntaxin 4 | Protein Coding | Q12846 | 55 | GC16P121076 | 1.93226301670074 | https://www.genecards.org/cgi-bin/carddisp.pl?gene=STX4 |  |
| ISM1 | Isthmin 1 | Protein Coding | B1AKI9 | 41 | GC20P013221 | 1.93083667755127 | https://www.genecards.org/cgi-bin/carddisp.pl?gene=ISM1 |  |
| ANP32A | Acidic Nuclear Phosphoprotein 32 Family Member A | Protein Coding | P39687 | 56 | GC15M068778 | 1.93052697181702 | https://www.genecards.org/cgi-bin/carddisp.pl?gene=ANP32A |  |
| GP9 | Glycoprotein IX Platelet | Protein Coding | P14770 | 58 | GC03P143109 | 1.92791342735291 | https://www.genecards.org/cgi-bin/carddisp.pl?gene=GP9 |  |
| USF2 | Upstream Transcription Factor 2, C-Fos Interacting | Protein Coding | Q15853 | 51 | GC19P035268 | 1.91999852657318 | https://www.genecards.org/cgi-bin/carddisp.pl?gene=USF2 |  |
| ADAMTS17 | ADAM Metallopeptidase With Thrombospondin Type 1 Motif 17 | Protein Coding | Q8TE56 | 51 | GC15M099971 | 1.90848851203918 | https://www.genecards.org/cgi-bin/carddisp.pl?gene=ADAMTS17 |  |
| SGK2 | Serum/Glucocorticoid Regulated Kinase 2 | Protein Coding | Q9HBY8 | 54 | GC20P043558 | 1.90050387382507 | https://www.genecards.org/cgi-bin/carddisp.pl?gene=SGK2 |  |
| KCNA4 | Potassium Voltage-Gated Channel Subfamily A Member 4 | Protein Coding | P22459 | 58 | GC11M030009 | 1.87481272220612 | https://www.genecards.org/cgi-bin/carddisp.pl?gene=KCNA4 |  |
| PLPPR5 | Phospholipid Phosphatase Related 5 | Protein Coding | Q32ZL2 | 41 | GC01M098891 | 1.87220108509064 | https://www.genecards.org/cgi-bin/carddisp.pl?gene=PLPPR5 |  |
| PGAP6 | Post-GPI Attachment To Proteins 6 | Protein Coding | Q9HCN3 | 41 | GC16M000370 | 1.86838400363922 | https://www.genecards.org/cgi-bin/carddisp.pl?gene=PGAP6 |  |
| CCR3 | C-C Motif Chemokine Receptor 3 | Protein Coding | P51677 | 59 | GC03P064845 | 1.86763274669647 | https://www.genecards.org/cgi-bin/carddisp.pl?gene=CCR3 |  |
| RBSN | Rabenosyn, RAB Effector | Protein Coding | Q9H1K0 | 47 | GC03M015070 | 1.86370170116425 | https://www.genecards.org/cgi-bin/carddisp.pl?gene=RBSN |  |
| MAPK6 | Mitogen-Activated Protein Kinase 6 | Protein Coding | Q16659 | 53 | GC15P051952 | 1.84711050987244 | https://www.genecards.org/cgi-bin/carddisp.pl?gene=MAPK6 |  |
| VAV1 | Vav Guanine Nucleotide Exchange Factor 1 | Protein Coding | P15498 | 58 | GC19P006772 | 1.84700298309326 | https://www.genecards.org/cgi-bin/carddisp.pl?gene=VAV1 |  |
| TSPAN32 | Tetraspanin 32 | Protein Coding | Q96QS1 | 45 | GC11P020317 | 1.8433963060379 | https://www.genecards.org/cgi-bin/carddisp.pl?gene=TSPAN32 |  |
| CRMA | Cardiomyocyte Maturation Associated LncRNA | RNA Gene |  | 22 | GC20M062909 | 1.83713614940643 | https://www.genecards.org/cgi-bin/carddisp.pl?gene=CRMA |  |
| CYP21A1P | Cytochrome P450 Family 21 Subfamily A Member 1, Pseudogene | Pseudogene |  | 23 | GC06P032005 | 1.81995606422424 | https://www.genecards.org/cgi-bin/carddisp.pl?gene=CYP21A1P |  |
| KRR1 | KRR1 Small Subunit Processome Component Homolog | Protein Coding | Q13601 | 47 | GC12M075490 | 1.81713855266571 | https://www.genecards.org/cgi-bin/carddisp.pl?gene=KRR1 |  |
| OPRK1 | Opioid Receptor Kappa 1 | Protein Coding | P41145 | 54 | GC08M053227 | 1.81110978126526 | https://www.genecards.org/cgi-bin/carddisp.pl?gene=OPRK1 |  |
| MIR181B1 | MicroRNA 181b-1 | RNA Gene |  | 28 | GC01M198858 | 1.80788993835449 | https://www.genecards.org/cgi-bin/carddisp.pl?gene=MIR181B1 |  |
| PLEKHH1 | Pleckstrin Homology, MyTH4 And FERM Domain Containing H1 | Protein Coding | Q9ULM0 | 39 | GC14P067533 | 1.80191743373871 | https://www.genecards.org/cgi-bin/carddisp.pl?gene=PLEKHH1 |  |
| SLC37A3 | Solute Carrier Family 37 Member 3 | Protein Coding | Q8NCC5 | 44 | GC07M140293 | 1.79654443264008 | https://www.genecards.org/cgi-bin/carddisp.pl?gene=SLC37A3 |  |
| PEPC | Peptidase C | Protein Coding |  | 7 | GC01U990121 | 1.78928422927856 | https://www.genecards.org/cgi-bin/carddisp.pl?gene=PEPC |  |
| CASP14 | Caspase 14 | Protein Coding | P31944 | 58 | GC19P015049 | 1.7880152463913 | https://www.genecards.org/cgi-bin/carddisp.pl?gene=CASP14 |  |
| TLR6 | Toll Like Receptor 6 | Protein Coding | Q9Y2C9 | 57 | GC04M038828 | 1.78265571594238 | https://www.genecards.org/cgi-bin/carddisp.pl?gene=TLR6 |  |
| LMCD1 | LIM And Cysteine Rich Domains 1 | Protein Coding | Q9NZU5 | 48 | GC03P008910 | 1.76584327220917 | https://www.genecards.org/cgi-bin/carddisp.pl?gene=LMCD1 |  |
| MIR542 | MicroRNA 542 | RNA Gene |  | 26 | GC0XM135415 | 1.76382148265839 | https://www.genecards.org/cgi-bin/carddisp.pl?gene=MIR542 |  |
| MCOLN3 | Mucolipin TRP Cation Channel 3 | Protein Coding | Q8TDD5 | 50 | GC01M085019 | 1.76062655448914 | https://www.genecards.org/cgi-bin/carddisp.pl?gene=MCOLN3 |  |
| CSK | C-Terminal Src Kinase | Protein Coding | P41240 | 59 | GC15P074782 | 1.76049423217773 | https://www.genecards.org/cgi-bin/carddisp.pl?gene=CSK |  |
| TXK | TXK Tyrosine Kinase | Protein Coding | P42681 | 56 | GC04M048353 | 1.75662124156952 | https://www.genecards.org/cgi-bin/carddisp.pl?gene=TXK |  |
| EPS15 | Epidermal Growth Factor Receptor Pathway Substrate 15 | Protein Coding | P42566 | 56 | GC01M051354 | 1.75594985485077 | https://www.genecards.org/cgi-bin/carddisp.pl?gene=EPS15 |  |
| BCLAF1 | BCL2 Associated Transcription Factor 1 | Protein Coding | Q9NYF8 | 51 | GC06M136256 | 1.74879550933838 | https://www.genecards.org/cgi-bin/carddisp.pl?gene=BCLAF1 |  |
| MIR543 | MicroRNA 543 | RNA Gene |  | 20 | GC14P120390 | 1.7436854839325 | https://www.genecards.org/cgi-bin/carddisp.pl?gene=MIR543 |  |
| MYOG | Myogenin | Protein Coding | P15173 | 52 | GC01M203083 | 1.74339878559113 | https://www.genecards.org/cgi-bin/carddisp.pl?gene=MYOG |  |
| PAG1 | Phosphoprotein Membrane Anchor With Glycosphingolipid Microdomains 1 | Protein Coding | Q9NWQ8 | 50 | GC08M080967 | 1.73785543441772 | https://www.genecards.org/cgi-bin/carddisp.pl?gene=PAG1 |  |
| APOL3 | Apolipoprotein L3 | Protein Coding | O95236 | 44 | GC22M086094 | 1.72913146018982 | https://www.genecards.org/cgi-bin/carddisp.pl?gene=APOL3 |  |
| CPD | Carboxypeptidase D | Protein Coding | O75976 | 52 | GC17P030378 | 1.72738242149353 | https://www.genecards.org/cgi-bin/carddisp.pl?gene=CPD |  |
| ST14 | ST14 Transmembrane Serine Protease Matriptase | Protein Coding | Q9Y5Y6 | 60 | GC11P130159 | 1.72176086902618 | https://www.genecards.org/cgi-bin/carddisp.pl?gene=ST14 |  |
| PITPNA | Phosphatidylinositol Transfer Protein Alpha | Protein Coding | Q00169 | 50 | GC17M095907 | 1.72011160850525 | https://www.genecards.org/cgi-bin/carddisp.pl?gene=PITPNA |  |
| CD1B | CD1b Molecule | Protein Coding | P29016 | 50 | GC01M167560 | 1.70712637901306 | https://www.genecards.org/cgi-bin/carddisp.pl?gene=CD1B |  |
| TMEM127 | Transmembrane Protein 127 | Protein Coding | O75204 | 50 | GC02M096248 | 1.70676338672638 | https://www.genecards.org/cgi-bin/carddisp.pl?gene=TMEM127 |  |
| GJA8 | Gap Junction Protein Alpha 8 | Protein Coding | P48165 | 57 | GC01P147902 | 1.70301961898804 | https://www.genecards.org/cgi-bin/carddisp.pl?gene=GJA8 |  |
| STX2 | Syntaxin 2 | Protein Coding | P32856 | 50 | GC12M130789 | 1.70237576961517 | https://www.genecards.org/cgi-bin/carddisp.pl?gene=STX2 |  |
| ATG4C | Autophagy Related 4C Cysteine Peptidase | Protein Coding | Q96DT6 | 45 | GC01P062784 | 1.69595122337341 | https://www.genecards.org/cgi-bin/carddisp.pl?gene=ATG4C |  |
| CTNND1 | Catenin Delta 1 | Protein Coding | O60716 | 58 | GC11P059474 | 1.68067622184753 | https://www.genecards.org/cgi-bin/carddisp.pl?gene=CTNND1 |  |
| MIR101-1 | MicroRNA 101-1 | RNA Gene |  | 28 | GC01M065058 | 1.67331397533417 | https://www.genecards.org/cgi-bin/carddisp.pl?gene=MIR101-1 |  |
| IL20RB | Interleukin 20 Receptor Subunit Beta | Protein Coding | Q6UXL0 | 46 | GC03P136946 | 1.63911259174347 | https://www.genecards.org/cgi-bin/carddisp.pl?gene=IL20RB |  |
| PPT2-EGFL8 | PPT2-EGFL8 Readthrough (NMD Candidate) | RNA Gene |  | 14 | GC06P181916 | 1.60152268409729 | https://www.genecards.org/cgi-bin/carddisp.pl?gene=PPT2-EGFL8 |  |
| GNA12 | G Protein Subunit Alpha 12 | Protein Coding | Q03113 | 54 | GC07M004006 | 1.59384036064148 | https://www.genecards.org/cgi-bin/carddisp.pl?gene=GNA12 |  |
| TMEM163 | Transmembrane Protein 163 | Protein Coding | Q8TC26 | 46 | GC02M134455 | 1.58778309822083 | https://www.genecards.org/cgi-bin/carddisp.pl?gene=TMEM163 |  |
| FCN2 | Ficolin 2 | Protein Coding | Q15485 | 57 | GC09P134864 | 1.58630704879761 | https://www.genecards.org/cgi-bin/carddisp.pl?gene=FCN2 |  |
| PAQR9 | Progestin And AdipoQ Receptor Family Member 9 | Protein Coding | Q6ZVX9 | 38 | GC03M142949 | 1.58333814144135 | https://www.genecards.org/cgi-bin/carddisp.pl?gene=PAQR9 |  |
| MAL | Mal, T Cell Differentiation Protein | Protein Coding | P21145 | 48 | GC02P095025 | 1.58328342437744 | https://www.genecards.org/cgi-bin/carddisp.pl?gene=MAL |  |
| MIR708 | MicroRNA 708 | RNA Gene |  | 28 | GC11M079402 | 1.57717132568359 | https://www.genecards.org/cgi-bin/carddisp.pl?gene=MIR708 |  |
| PRSS3 | Serine Protease 3 | Protein Coding | P35030 | 55 | GC09P033750 | 1.57078731060028 | https://www.genecards.org/cgi-bin/carddisp.pl?gene=PRSS3 |  |
| LAG3 | Lymphocyte Activating 3 | Protein Coding | P18627 | 53 | GC12P074069 | 1.56633841991425 | https://www.genecards.org/cgi-bin/carddisp.pl?gene=LAG3 |  |
| NPDC1 | Neural Proliferation, Differentiation And Control 1 | Protein Coding | Q9NQX5 | 46 | GC09M137039 | 1.55806541442871 | https://www.genecards.org/cgi-bin/carddisp.pl?gene=NPDC1 |  |
| EPN3 | Epsin 3 | Protein Coding | Q9H201 | 45 | GC17P050532 | 1.55777609348297 | https://www.genecards.org/cgi-bin/carddisp.pl?gene=EPN3 |  |
| TNFSF15 | TNF Superfamily Member 15 | Protein Coding | O95150 | 55 | GC09M114784 | 1.55734801292419 | https://www.genecards.org/cgi-bin/carddisp.pl?gene=TNFSF15 |  |
| CLTB | Clathrin Light Chain B | Protein Coding | P09497 | 50 | GC05M176392 | 1.54681539535522 | https://www.genecards.org/cgi-bin/carddisp.pl?gene=CLTB |  |
| MEIS1 | Meis Homeobox 1 | Protein Coding | O00470 | 54 | GC02P066433 | 1.54633319377899 | https://www.genecards.org/cgi-bin/carddisp.pl?gene=MEIS1 |  |
| C1QL2 | Complement C1q Like 2 | Protein Coding | Q7Z5L3 | 41 | GC02M119156 | 1.53907811641693 | https://www.genecards.org/cgi-bin/carddisp.pl?gene=C1QL2 |  |
| PCDH20 | Protocadherin 20 | Protein Coding | Q8N6Y1 | 44 | GC13M061409 | 1.53821420669556 | https://www.genecards.org/cgi-bin/carddisp.pl?gene=PCDH20 |  |
| LINC00347 | Long Intergenic Non-Protein Coding RNA 347 | RNA Gene |  | 15 | GC13P074423 | 1.53821420669556 | https://www.genecards.org/cgi-bin/carddisp.pl?gene=LINC00347 |  |
| RPL21P108 | Ribosomal Protein L21 Pseudogene 108 | Pseudogene |  | 10 | GC13M074377 | 1.53821420669556 | https://www.genecards.org/cgi-bin/carddisp.pl?gene=RPL21P108 |  |
| RPL8P3 | Ribosomal Protein L8 Pseudogene 3 | Pseudogene |  | 9 | GC03M142827 | 1.53821420669556 | https://www.genecards.org/cgi-bin/carddisp.pl?gene=RPL8P3 |  |
| EIF4A1P6 | Eukaryotic Translation Initiation Factor 4A1 Pseudogene 6 | Pseudogene |  | 8 | GC13M060636 | 1.53821420669556 | https://www.genecards.org/cgi-bin/carddisp.pl?gene=EIF4A1P6 |  |
| NPM1P3 | Nucleophosmin 1 Pseudogene 3 | Pseudogene |  | 8 | GC16P120268 | 1.53821420669556 | https://www.genecards.org/cgi-bin/carddisp.pl?gene=NPM1P3 |  |
| RPS27P27 | Ribosomal Protein S27 Pseudogene 27 | Pseudogene |  | 8 | GC16P121719 | 1.53821420669556 | https://www.genecards.org/cgi-bin/carddisp.pl?gene=RPS27P27 |  |
| SLC26A11 | Solute Carrier Family 26 Member 11 | Protein Coding | Q86WA9 | 47 | GC17P080219 | 1.53684628009796 | https://www.genecards.org/cgi-bin/carddisp.pl?gene=SLC26A11 |  |
| PLSCR1 | Phospholipid Scramblase 1 | Protein Coding | O15162 | 54 | GC03M146515 | 1.53413593769073 | https://www.genecards.org/cgi-bin/carddisp.pl?gene=PLSCR1 |  |
| MIR135B | MicroRNA 135b | RNA Gene |  | 30 | GC01M205448 | 1.52533411979675 | https://www.genecards.org/cgi-bin/carddisp.pl?gene=MIR135B |  |
| GSDMB | Gasdermin B | Protein Coding | Q8TAX9 | 44 | GC17M039904 | 1.5243079662323 | https://www.genecards.org/cgi-bin/carddisp.pl?gene=GSDMB |  |
| MYO10 | Myosin X | Protein Coding | Q9HD67 | 48 | GC05M016661 | 1.52425193786621 | https://www.genecards.org/cgi-bin/carddisp.pl?gene=MYO10 |  |
| PTCH2 | Patched 2 | Protein Coding | Q9Y6C5 | 57 | GC01M044819 | 1.51761841773987 | https://www.genecards.org/cgi-bin/carddisp.pl?gene=PTCH2 |  |
| WBP2 | WW Domain Binding Protein 2 | Protein Coding | Q969T9 | 51 | GC17M075845 | 1.51466345787048 | https://www.genecards.org/cgi-bin/carddisp.pl?gene=WBP2 |  |
| APBB2 | Amyloid Beta Precursor Protein Binding Family B Member 2 | Protein Coding | Q92870 | 51 | GC04M040812 | 1.49528336524963 | https://www.genecards.org/cgi-bin/carddisp.pl?gene=APBB2 |  |
| FOXC1 | Forkhead Box C1 | Protein Coding | Q12948 | 55 | GC06P002084 | 1.49493300914764 | https://www.genecards.org/cgi-bin/carddisp.pl?gene=FOXC1 |  |
| MAP2K6 | Mitogen-Activated Protein Kinase Kinase 6 | Protein Coding | P52564 | 60 | GC17P069414 | 1.49418663978577 | https://www.genecards.org/cgi-bin/carddisp.pl?gene=MAP2K6 |  |
| EPN2 | Epsin 2 | Protein Coding | O95208 | 50 | GC17P019215 | 1.47638487815857 | https://www.genecards.org/cgi-bin/carddisp.pl?gene=EPN2 |  |
| GSDMA | Gasdermin A | Protein Coding | Q96QA5 | 47 | GC17P154759 | 1.46254217624664 | https://www.genecards.org/cgi-bin/carddisp.pl?gene=GSDMA |  |
| TNFRSF10B | TNF Receptor Superfamily Member 10b | Protein Coding | O14763 | 62 | GC08M023006 | 1.45885026454926 | https://www.genecards.org/cgi-bin/carddisp.pl?gene=TNFRSF10B |  |
| RAB11FIP2 | RAB11 Family Interacting Protein 2 | Protein Coding | Q7L804 | 50 | GC10M118004 | 1.44908285140991 | https://www.genecards.org/cgi-bin/carddisp.pl?gene=RAB11FIP2 |  |
| TXNRD3 | Thioredoxin Reductase 3 | Protein Coding | Q86VQ6 | 49 | GC03M126577 | 1.44500041007996 | https://www.genecards.org/cgi-bin/carddisp.pl?gene=TXNRD3 |  |
| LAT2 | Linker For Activation Of T Cells Family Member 2 | Protein Coding | Q9GZY6 | 50 | GC07P074199 | 1.44296658039093 | https://www.genecards.org/cgi-bin/carddisp.pl?gene=LAT2 |  |
| ZDHHC8 | Zinc Finger DHHC-Type Palmitoyltransferase 8 | Protein Coding | Q9ULC8 | 51 | GC22P020129 | 1.43736600875854 | https://www.genecards.org/cgi-bin/carddisp.pl?gene=ZDHHC8 |  |
| TM9SF2 | Transmembrane 9 Superfamily Member 2 | Protein Coding | Q99805 | 43 | GC13P099446 | 1.43636429309845 | https://www.genecards.org/cgi-bin/carddisp.pl?gene=TM9SF2 |  |
| PACRG | Parkin Coregulated | Protein Coding | Q96M98 | 51 | GC06P162727 | 1.42684316635132 | https://www.genecards.org/cgi-bin/carddisp.pl?gene=PACRG |  |
| ZDHHC20 | Zinc Finger DHHC-Type Palmitoyltransferase 20 | Protein Coding | Q5W0Z9 | 42 | GC13M024853 | 1.42378890514374 | https://www.genecards.org/cgi-bin/carddisp.pl?gene=ZDHHC20 |  |
| GTF3A | General Transcription Factor IIIA | Protein Coding | Q92664 | 45 | GC13P028953 | 1.41931021213531 | https://www.genecards.org/cgi-bin/carddisp.pl?gene=GTF3A |  |
| UNC5C | Unc-5 Netrin Receptor C | Protein Coding | O95185 | 52 | GC04M095162 | 1.40906035900116 | https://www.genecards.org/cgi-bin/carddisp.pl?gene=UNC5C |  |
| BEX3 | Brain Expressed X-Linked 3 | Protein Coding | Q00994 | 40 | GC0XP104044 | 1.38125276565552 | https://www.genecards.org/cgi-bin/carddisp.pl?gene=BEX3 |  |
| SGK3 | Serum/Glucocorticoid Regulated Kinase Family Member 3 | Protein Coding | Q96BR1 | 51 | GC08P066712 | 1.38022971153259 | https://www.genecards.org/cgi-bin/carddisp.pl?gene=SGK3 |  |
| RHOD | Ras Homolog Family Member D | Protein Coding | O00212 | 50 | GC11P107960 | 1.352050781 | https://www.genecards.org/cgi-bin/carddisp.pl?gene=RHOD |  |
| DNAJB4 | DnaJ Heat Shock Protein Family (Hsp40) Member B4 | Protein Coding | Q9UDY4 | 51 | GC01P077979 | 1.34911000728607 | https://www.genecards.org/cgi-bin/carddisp.pl?gene=DNAJB4 |  |
| FCER1A | Fc Epsilon Receptor Ia | Protein Coding | P12319 | 54 | GC01P159283 | 1.34133636951447 | https://www.genecards.org/cgi-bin/carddisp.pl?gene=FCER1A |  |
| GNLY | Granulysin | Protein Coding | P22749 | 50 | GC02P085685 | 1.33447027206421 | https://www.genecards.org/cgi-bin/carddisp.pl?gene=GNLY |  |
| EHD3 | EH Domain Containing 3 | Protein Coding | Q9NZN3 | 46 | GC02P031234 | 1.31257486343384 | https://www.genecards.org/cgi-bin/carddisp.pl?gene=EHD3 |  |
| ASIC3 | Acid Sensing Ion Channel Subunit 3 | Protein Coding | Q9UHC3 | 52 | GC07P151048 | 1.29635179042816 | https://www.genecards.org/cgi-bin/carddisp.pl?gene=ASIC3 |  |
| SGMS1-AS1 | SGMS1 Antisense RNA 1 | RNA Gene |  | 15 | GC10P052972 | 1.28534173965454 | https://www.genecards.org/cgi-bin/carddisp.pl?gene=SGMS1-AS1 |  |
| EPN1 | Epsin 1 | Protein Coding | Q9Y6I3 | 50 | GC19P153145 | 1.28499054908752 | https://www.genecards.org/cgi-bin/carddisp.pl?gene=EPN1 |  |
| TRADD | TNFRSF1A Associated Via Death Domain | Protein Coding | Q15628 | 55 | GC16M067154 | 1.2752673625946 | https://www.genecards.org/cgi-bin/carddisp.pl?gene=TRADD |  |
| FGD4 | FYVE, RhoGEF And PH Domain Containing 4 | Protein Coding | Q96M96 | 56 | GC12P074679 | 1.27196037769318 | https://www.genecards.org/cgi-bin/carddisp.pl?gene=FGD4 |  |
| ZDHHC7 | Zinc Finger DHHC-Type Palmitoyltransferase 7 | Protein Coding | Q9NXF8 | 49 | GC16M084975 | 1.26891851425171 | https://www.genecards.org/cgi-bin/carddisp.pl?gene=ZDHHC7 |  |
| YES1 | YES Proto-Oncogene 1, Src Family Tyrosine Kinase | Protein Coding | P07947 | 58 | GC18M000721 | 1.2542130947113 | https://www.genecards.org/cgi-bin/carddisp.pl?gene=YES1 |  |
| GOLGA5 | Golgin A5 | Protein Coding | Q8TBA6 | 48 | GC14P092794 | 1.24346029758453 | https://www.genecards.org/cgi-bin/carddisp.pl?gene=GOLGA5 |  |
| NIPAL1 | NIPA Like Domain Containing 1 | Protein Coding | Q6NVV3 | 45 | GC04P047917 | 1.23824882507324 | https://www.genecards.org/cgi-bin/carddisp.pl?gene=NIPAL1 |  |
| ARHGAP45 | Rho GTPase Activating Protein 45 | Protein Coding | Q92619 | 49 | GC19P001065 | 1.22350418567657 | https://www.genecards.org/cgi-bin/carddisp.pl?gene=ARHGAP45 |  |
| lnc-KANK3-3 |  | RNA Gene |  | 5 | GC19M008101 | 1.22084057331085 | https://www.genecards.org/cgi-bin/carddisp.pl?gene=lnc-KANK3-3 |  |
| THNSL1 | Threonine Synthase Like 1 | Protein Coding | Q8IYQ7 | 40 | GC10P025077 | 1.22014474868774 | https://www.genecards.org/cgi-bin/carddisp.pl?gene=THNSL1 |  |
| LILRA2 | Leukocyte Immunoglobulin Like Receptor A2 | Protein Coding | Q8N149 | 50 | GC19P054572 | 1.21432542800903 | https://www.genecards.org/cgi-bin/carddisp.pl?gene=LILRA2 |  |
| LOC110806263 | TERT 5' Regulatory Region | Functional Element |  | 10 | GC05P001294 | 1.21196341514587 | https://www.genecards.org/cgi-bin/carddisp.pl?gene=LOC110806263 |  |
| IRGM | Immunity Related GTPase M | Protein Coding | A1A4Y4 | 45 | GC05P150846 | 1.19796228408813 | https://www.genecards.org/cgi-bin/carddisp.pl?gene=IRGM |  |
| CCR6 | C-C Motif Chemokine Receptor 6 | Protein Coding | P51684 | 55 | GC06P167111 | 1.1948299407959 | https://www.genecards.org/cgi-bin/carddisp.pl?gene=CCR6 |  |
| SLC26A9 | Solute Carrier Family 26 Member 9 | Protein Coding | Q7LBE3 | 50 | GC01M205883 | 1.18971836566925 | https://www.genecards.org/cgi-bin/carddisp.pl?gene=SLC26A9 |  |
| ELK4 | ETS Transcription Factor ELK4 | Protein Coding | P28324 | 50 | GC01M205598 | 1.18753945827484 | https://www.genecards.org/cgi-bin/carddisp.pl?gene=ELK4 |  |
| NEUROD6 | Neuronal Differentiation 6 | Protein Coding | Q96NK8 | 45 | GC07M031733 | 1.18690490722656 | https://www.genecards.org/cgi-bin/carddisp.pl?gene=NEUROD6 |  |
| UBE2V1 | Ubiquitin Conjugating Enzyme E2 V1 | Protein Coding | Q13404 | 53 | GC20M050082 | 1.18563783168793 | https://www.genecards.org/cgi-bin/carddisp.pl?gene=UBE2V1 |  |
| FMR1-AS1 | FMR1 Antisense RNA 1 | RNA Gene |  | 25 | GC0XM147891 | 1.17545485496521 | https://www.genecards.org/cgi-bin/carddisp.pl?gene=FMR1-AS1 |  |
| C11orf58 | Chromosome 11 Open Reading Frame 58 | Protein Coding | O00193 | 43 | GC11P016613 | 1.17454516887665 | https://www.genecards.org/cgi-bin/carddisp.pl?gene=C11orf58 |  |
| RAB39A | RAB39A, Member RAS Oncogene Family | Protein Coding | Q14964 | 44 | GC11P107928 | 1.16666769981384 | https://www.genecards.org/cgi-bin/carddisp.pl?gene=RAB39A |  |
| SPHKAP | SPHK1 Interactor, AKAP Domain Containing | Protein Coding | Q2M3C7 | 39 | GC02M227979 | 1.15884244441986 | https://www.genecards.org/cgi-bin/carddisp.pl?gene=SPHKAP |  |
| MARCHF8 | Membrane Associated Ring-CH-Type Finger 8 | Protein Coding | Q5T0T0 | 46 | GC10M049314 | 1.15271937847137 | https://www.genecards.org/cgi-bin/carddisp.pl?gene=MARCHF8 |  |
| CASP4 | Caspase 4 | Protein Coding | P49662 | 60 | GC11M104942 | 1.12365412712097 | https://www.genecards.org/cgi-bin/carddisp.pl?gene=CASP4 |  |
| GNA13 | G Protein Subunit Alpha 13 | Protein Coding | Q14344 | 55 | GC17M065009 | 1.11946058273315 | https://www.genecards.org/cgi-bin/carddisp.pl?gene=GNA13 |  |
| BCL2L15 | BCL2 Like 15 | Protein Coding | Q5TBC7 | 38 | GC01M113876 | 1.11946058273315 | https://www.genecards.org/cgi-bin/carddisp.pl?gene=BCL2L15 |  |
| GULP1 | GULP PTB Domain Containing Engulfment Adaptor 1 | Protein Coding | Q9UBP9 | 46 | GC02P188291 | 1.10711765289307 | https://www.genecards.org/cgi-bin/carddisp.pl?gene=GULP1 |  |
| S100A12 | S100 Calcium Binding Protein A12 | Protein Coding | P80511 | 50 | GC01M153373 | 1.0994701385498 | https://www.genecards.org/cgi-bin/carddisp.pl?gene=S100A12 |  |
| CCDC122 | Coiled-Coil Domain Containing 122 | Protein Coding | Q5T0U0 | 41 | GC13M043823 | 1.08902370929718 | https://www.genecards.org/cgi-bin/carddisp.pl?gene=CCDC122 |  |
| TBKBP1 | TBK1 Binding Protein 1 | Protein Coding | A7MCY6 | 47 | GC17P047694 | 1.08113598823547 | https://www.genecards.org/cgi-bin/carddisp.pl?gene=TBKBP1 |  |
| FGR | FGR Proto-Oncogene, Src Family Tyrosine Kinase | Protein Coding | P09769 | 60 | GC01M034833 | 1.07953524589539 | https://www.genecards.org/cgi-bin/carddisp.pl?gene=FGR |  |
| SNHG17 | Small Nucleolar RNA Host Gene 17 | RNA Gene |  | 25 | GC20M038993 | 1.06615006923676 | https://www.genecards.org/cgi-bin/carddisp.pl?gene=SNHG17 |  |
| CNGA2 | Cyclic Nucleotide Gated Channel Subunit Alpha 2 | Protein Coding | Q16280 | 49 | GC0XP151734 | 1.06444978713989 | https://www.genecards.org/cgi-bin/carddisp.pl?gene=CNGA2 |  |
| UNC5B | Unc-5 Netrin Receptor B | Protein Coding | Q8IZJ1 | 51 | GC10P071212 | 1.06354141235352 | https://www.genecards.org/cgi-bin/carddisp.pl?gene=UNC5B |  |
| ANKRD30A | Ankyrin Repeat Domain 30A | Protein Coding | Q9BXX3 | 45 | GC10P037134 | 1.06118011474609 | https://www.genecards.org/cgi-bin/carddisp.pl?gene=ANKRD30A |  |
| PLBD2 | Phospholipase B Domain Containing 2 | Protein Coding | Q8NHP8 | 44 | GC12P113358 | 1.05294525623322 | https://www.genecards.org/cgi-bin/carddisp.pl?gene=PLBD2 |  |
| MIR1-2 | MicroRNA 1-2 | RNA Gene |  | 23 | GC18M038258 | 1.04107308387756 | https://www.genecards.org/cgi-bin/carddisp.pl?gene=MIR1-2 |  |
| TPD52 | Tumor Protein D52 | Protein Coding | P55327 | 52 | GC08M080031 | 1.01860308647156 | https://www.genecards.org/cgi-bin/carddisp.pl?gene=TPD52 |  |
| CASP5 | Caspase 5 | Protein Coding | P51878 | 56 | GC11M140425 | 1.0175164937973 | https://www.genecards.org/cgi-bin/carddisp.pl?gene=CASP5 |  |
| TRAM2 | Translocation Associated Membrane Protein 2 | Protein Coding | Q15035 | 47 | GC06M106850 | 0.995030879974365 | https://www.genecards.org/cgi-bin/carddisp.pl?gene=TRAM2 |  |
| SEC61G | SEC61 Translocon Subunit Gamma | Protein Coding | P60059 | 43 | GC07M054752 | 0.99032735824585 | https://www.genecards.org/cgi-bin/carddisp.pl?gene=SEC61G |  |
| SNHG32 | Small Nucleolar RNA Host Gene 32 | RNA Gene |  | 25 | GC06P190453 | 0.989833056926727 | https://www.genecards.org/cgi-bin/carddisp.pl?gene=SNHG32 |  |
| EFS | Embryonal Fyn-Associated Substrate | Protein Coding | O43281 | 47 | GC14M023356 | 0.989097595214844 | https://www.genecards.org/cgi-bin/carddisp.pl?gene=EFS |  |
| KCNJ9 | Potassium Inwardly Rectifying Channel Subfamily J Member 9 | Protein Coding | Q92806 | 52 | GC01P176944 | 0.986767649650574 | https://www.genecards.org/cgi-bin/carddisp.pl?gene=KCNJ9 |  |
| HCG27 | HLA Complex Group 27 | RNA Gene |  | 27 | GC06P031197 | 0.9773850440979 | https://www.genecards.org/cgi-bin/carddisp.pl?gene=HCG27 |  |
| PPM1A | Protein Phosphatase, Mg2+/Mn2+ Dependent 1A | Protein Coding | P35813 | 56 | GC14P060245 | 0.973826110363007 | https://www.genecards.org/cgi-bin/carddisp.pl?gene=PPM1A |  |
| IL18RAP | Interleukin 18 Receptor Accessory Protein | Protein Coding | O95256 | 50 | GC02P102418 | 0.965660214424133 | https://www.genecards.org/cgi-bin/carddisp.pl?gene=IL18RAP |  |
| AP3M2 | Adaptor Related Protein Complex 3 Subunit Mu 2 | Protein Coding | P53677 | 45 | GC08P042152 | 0.944006681442261 | https://www.genecards.org/cgi-bin/carddisp.pl?gene=AP3M2 |  |
| TUBA3C | Tubulin Alpha 3c | Protein Coding | P0DPH7 | 49 | GC13M019173 | 0.935818731784821 | https://www.genecards.org/cgi-bin/carddisp.pl?gene=TUBA3C |  |
| STX6 | Syntaxin 6 | Protein Coding | O43752 | 50 | GC01M180972 | 0.902862071990967 | https://www.genecards.org/cgi-bin/carddisp.pl?gene=STX6 |  |
| PHOSPHO2 | Phosphatase, Orphan 2 | Protein Coding | Q8TCD6 | 45 | GC02P169766 | 0.89868175983429 | https://www.genecards.org/cgi-bin/carddisp.pl?gene=PHOSPHO2 |  |
| PROM2 | Prominin 2 | Protein Coding | Q8N271 | 44 | GC02P095274 | 0.87615442276001 | https://www.genecards.org/cgi-bin/carddisp.pl?gene=PROM2 |  |
| GPR4 | G Protein-Coupled Receptor 4 | Protein Coding | P46093 | 50 | GC19M045589 | 0.864201128482819 | https://www.genecards.org/cgi-bin/carddisp.pl?gene=GPR4 |  |
| CLPTM1 | CLPTM1 Regulator Of GABA Type A Receptor Forward Trafficking | Protein Coding | O96005 | 47 | GC19P044954 | 0.861262142658234 | https://www.genecards.org/cgi-bin/carddisp.pl?gene=CLPTM1 |  |
| GJA3 | Gap Junction Protein Alpha 3 | Protein Coding | Q9Y6H8 | 54 | GC13M024816 | 0.854799270629883 | https://www.genecards.org/cgi-bin/carddisp.pl?gene=GJA3 |  |
| MFSD1 | Major Facilitator Superfamily Domain Containing 1 | Protein Coding | Q9H3U5 | 44 | GC03P158809 | 0.851671040058136 | https://www.genecards.org/cgi-bin/carddisp.pl?gene=MFSD1 |  |
| IFNGR2 | Interferon Gamma Receptor 2 | Protein Coding | P38484 | 56 | GC21P033402 | 0.848173797130585 | https://www.genecards.org/cgi-bin/carddisp.pl?gene=IFNGR2 |  |
| LOC110408762 | CYP17A1 Promoter | Functional Element |  | 10 | GC10P102837 | 0.832209348678589 | https://www.genecards.org/cgi-bin/carddisp.pl?gene=LOC110408762 |  |
| LYPD1 | LY6/PLAUR Domain Containing 1 | Protein Coding | Q8N2G4 | 44 | GC02M138448 | 0.826371848583221 | https://www.genecards.org/cgi-bin/carddisp.pl?gene=LYPD1 |  |
| GPR45 | G Protein-Coupled Receptor 45 | Protein Coding | Q9Y5Y3 | 42 | GC02P105241 | 0.817898392677307 | https://www.genecards.org/cgi-bin/carddisp.pl?gene=GPR45 |  |
| ERVK-6 | Endogenous Retrovirus Group K Member 6, Envelope | Protein Coding | Q69384 | 25 | GC07U903184 | 0.817849934101105 | https://www.genecards.org/cgi-bin/carddisp.pl?gene=ERVK-6 |  |
| PWAR6 | Prader Willi/Angelman Region RNA 6 | RNA Gene |  | 18 | GC15P025031 | 0.816756248474121 | https://www.genecards.org/cgi-bin/carddisp.pl?gene=PWAR6 |  |
| CCSER1 | Coiled-Coil Serine Rich Protein 1 | Protein Coding | Q9C0I3 | 41 | GC04P090127 | 0.806355178356171 | https://www.genecards.org/cgi-bin/carddisp.pl?gene=CCSER1 |  |
| LINC01226 | Long Intergenic Non-Protein Coding RNA 1226 | RNA Gene |  | 18 | GC01P076659 | 0.804603040218353 | https://www.genecards.org/cgi-bin/carddisp.pl?gene=LINC01226 |  |
| LDC1P | Leucine Decarboxylase 1, Pseudogene | Pseudogene |  | 15 | GC01P076658 | 0.804603040218353 | https://www.genecards.org/cgi-bin/carddisp.pl?gene=LDC1P |  |
| ETV4 | ETS Variant Transcription Factor 4 | Protein Coding | P43268 | 54 | GC17M043527 | 0.789915084838867 | https://www.genecards.org/cgi-bin/carddisp.pl?gene=ETV4 |  |
| GNL3L | G Protein Nucleolar 3 Like | Protein Coding | Q9NVN8 | 41 | GC0XP061087 | 0.746143162250519 | https://www.genecards.org/cgi-bin/carddisp.pl?gene=GNL3L |  |
| THNSL2 | Threonine Synthase Like 2 | Protein Coding | Q86YJ6 | 41 | GC02P088170 | 0.710827052593231 | https://www.genecards.org/cgi-bin/carddisp.pl?gene=THNSL2 |  |
| BST2 | Bone Marrow Stromal Cell Antigen 2 | Protein Coding | Q10589 | 52 | GC19M108366 | 0.696400165557861 | https://www.genecards.org/cgi-bin/carddisp.pl?gene=BST2 |  |
| GPR68 | G Protein-Coupled Receptor 68 | Protein Coding | Q15743 | 54 | GC14M091232 | 0.673470854759216 | https://www.genecards.org/cgi-bin/carddisp.pl?gene=GPR68 |  |
| BIRC8 | Baculoviral IAP Repeat Containing 8 | Pseudogene | Q96P09 | 38 | GC19M053289 | 0.673470854759216 | https://www.genecards.org/cgi-bin/carddisp.pl?gene=BIRC8 |  |
| FAXDC2 | Fatty Acid Hydroxylase Domain Containing 2 | Protein Coding | Q96IV6 | 41 | GC05M154817 | 0.665269196033478 | https://www.genecards.org/cgi-bin/carddisp.pl?gene=FAXDC2 |  |
| MIR219B | MicroRNA 219b | RNA Gene |  | 13 | GC09P152887 | 0.651276290416718 | https://www.genecards.org/cgi-bin/carddisp.pl?gene=MIR219B |  |
| ELK3 | ETS Transcription Factor ELK3 | Protein Coding | P41970 | 45 | GC12P096194 | 0.649629235267639 | https://www.genecards.org/cgi-bin/carddisp.pl?gene=ELK3 |  |
| SH3BGRL2 | SH3 Domain Binding Glutamate Rich Protein Like 2 | Protein Coding | Q9UJC5 | 40 | GC06P182381 | 0.634405910968781 | https://www.genecards.org/cgi-bin/carddisp.pl?gene=SH3BGRL2 |  |
| NKPD1 | NTPase KAP Family P-Loop Domain Containing 1 | Protein Coding | Q17RQ9 | 39 | GC19M108881 | 0.631898999214172 | https://www.genecards.org/cgi-bin/carddisp.pl?gene=NKPD1 |  |
| TAS2R14 | Taste 2 Receptor Member 14 | Protein Coding | Q9NYV8 | 42 | GC12M010937 | 0.61936891078949 | https://www.genecards.org/cgi-bin/carddisp.pl?gene=TAS2R14 |  |
| CLDN4 | Claudin 4 | Protein Coding | O14493 | 53 | GC07P073799 | 0.540695071220398 | https://www.genecards.org/cgi-bin/carddisp.pl?gene=CLDN4 |  |
| ZNF516 | Zinc Finger Protein 516 | Protein Coding | Q92618 | 43 | GC18M076357 | 0.535876333713531 | https://www.genecards.org/cgi-bin/carddisp.pl?gene=ZNF516 |  |
| SAMSN1 | SAM Domain, SH3 Domain And Nuclear Localization Signals 1 | Protein Coding | Q9NSI8 | 45 | GC21M014485 | 0.52374666929245 | https://www.genecards.org/cgi-bin/carddisp.pl?gene=SAMSN1 |  |
| TMEM219 | Transmembrane Protein 219 | Protein Coding | Q86XT9 | 45 | GC16P120997 | 0.491839289665222 | https://www.genecards.org/cgi-bin/carddisp.pl?gene=TMEM219 |  |
| ZHX2 | Zinc Fingers And Homeoboxes 2 | Protein Coding | Q9Y6X8 | 48 | GC08P122783 | 0.485429883003235 | https://www.genecards.org/cgi-bin/carddisp.pl?gene=ZHX2 |  |
| ENDOG | Endonuclease G | Protein Coding | Q14249 | 56 | GC09P128818 | 0.481257379055023 | https://www.genecards.org/cgi-bin/carddisp.pl?gene=ENDOG |  |
| IGSF3 | Immunoglobulin Superfamily Member 3 | Protein Coding | O75054 | 51 | GC01M116574 | 0.477477490901947 | https://www.genecards.org/cgi-bin/carddisp.pl?gene=IGSF3 |  |
| LOC106627982 | GBAP1 Recombination Region | Functional Element |  | 6 | GC01P176750 | 0.432187229394913 | https://www.genecards.org/cgi-bin/carddisp.pl?gene=LOC106627982 |  |
| ZNF750 | Zinc Finger Protein 750 | Protein Coding | Q32MQ0 | 47 | GC17M082829 | 0.429039090871811 | https://www.genecards.org/cgi-bin/carddisp.pl?gene=ZNF750 |  |
| GFRA3 | GDNF Family Receptor Alpha 3 | Protein Coding | O60609 | 57 | GC05M138263 | 0.416685342788696 | https://www.genecards.org/cgi-bin/carddisp.pl?gene=GFRA3 |  |
| ANXA13 | Annexin A13 | Protein Coding | P27216 | 50 | GC08M123680 | 0.416685342788696 | https://www.genecards.org/cgi-bin/carddisp.pl?gene=ANXA13 |  |
| ZNF608 | Zinc Finger Protein 608 | Protein Coding | Q9ULD9 | 44 | GC05M124636 | 0.403100550174713 | https://www.genecards.org/cgi-bin/carddisp.pl?gene=ZNF608 |  |
| LOC129935214 | ATAC-STARR-Seq Lymphoblastoid Silent Region 12157 | Functional Element |  | 7 | GC02P181656 | 0.380257487297058 | https://www.genecards.org/cgi-bin/carddisp.pl?gene=LOC129935214 |  |
| LOC129935215 | ATAC-STARR-Seq Lymphoblastoid Silent Region 12158 | Functional Element |  | 7 | GC02P181657 | 0.380257487297058 | https://www.genecards.org/cgi-bin/carddisp.pl?gene=LOC129935215 |  |
| CDH3 | Cadherin 3 | Protein Coding | P22223 | 60 | GC16P121938 | 0.355418622493744 | https://www.genecards.org/cgi-bin/carddisp.pl?gene=CDH3 |  |
| ELF3 | E74 Like ETS Transcription Factor 3 | Protein Coding | P78545 | 51 | GC01P202007 | 0.355418622493744 | https://www.genecards.org/cgi-bin/carddisp.pl?gene=ELF3 |  |
| UPK3A | Uroplakin 3A | Protein Coding | O75631 | 50 | GC22P045284 | 0.352007865905762 | https://www.genecards.org/cgi-bin/carddisp.pl?gene=UPK3A |  |
| FAM215A | Family With Sequence Similarity 215 Member A | RNA Gene | Q9Y5M1 | 32 | GC17P043917 | 0.34783536195755 | https://www.genecards.org/cgi-bin/carddisp.pl?gene=FAM215A |  |
| GCA | Grancalcin | Protein Coding | P28676 | 48 | GC02P162318 | 0.340300351381302 | https://www.genecards.org/cgi-bin/carddisp.pl?gene=GCA |  |
| RALB | RAS Like Proto-Oncogene B | Protein Coding | P11234 | 54 | GC02P120240 | 0.323860228061676 | https://www.genecards.org/cgi-bin/carddisp.pl?gene=RALB |  |
| CLN9 | Ceroid-Lipofuscinosis, Neuronal 9 | Genetic Locus |  | 2 | GC00U921515 | 0.323351949453354 | https://www.genecards.org/cgi-bin/carddisp.pl?gene=CLN9 |  |
| UNC5A | Unc-5 Netrin Receptor A | Protein Coding | Q6ZN44 | 48 | GC05P190342 | 0.305675595998764 | https://www.genecards.org/cgi-bin/carddisp.pl?gene=UNC5A |  |
| LOC130059394 | ATAC-STARR-Seq Lymphoblastoid Silent Region 7701 | Functional Element |  | 9 | GC16P127279 | 0.291444569826126 | https://www.genecards.org/cgi-bin/carddisp.pl?gene=LOC130059394 |  |
| LOC102724528 | Uncharacterized LOC102724528 | RNA Gene |  | 5 | GC01M172393 | 0.291444569826126 | https://www.genecards.org/cgi-bin/carddisp.pl?gene=LOC102724528 |  |
| FAM171A2 | Family With Sequence Similarity 171 Member A2 | Protein Coding | A8MVW0 | 45 | GC17M044353 | 0.285035133361816 | https://www.genecards.org/cgi-bin/carddisp.pl?gene=FAM171A2 |  |
| CTNNBIP1 | Catenin Beta Interacting Protein 1 | Protein Coding | Q9NSA3 | 52 | GC01M009848 | 0.228644356131554 | https://www.genecards.org/cgi-bin/carddisp.pl?gene=CTNNBIP1 |  |
| KLRB1 | Killer Cell Lectin Like Receptor B1 | Protein Coding | Q12918 | 51 | GC12M035788 | 0.228644356131554 | https://www.genecards.org/cgi-bin/carddisp.pl?gene=KLRB1 |  |
| NPS | Neuropeptide S | Protein Coding | P0C0P6 | 41 | GC10P127549 | 0.228644356131554 | https://www.genecards.org/cgi-bin/carddisp.pl?gene=NPS |  |
| HOXA-AS3 | HOXA Cluster Antisense RNA 3 | RNA Gene |  | 24 | GC07P027129 | 0.228644356131554 | https://www.genecards.org/cgi-bin/carddisp.pl?gene=HOXA-AS3 |  |
| SNORD58B | Small Nucleolar RNA, C/D Box 58B | RNA Gene |  | 18 | GC18M050123 | 0.228644356131554 | https://www.genecards.org/cgi-bin/carddisp.pl?gene=SNORD58B |  |
| MIR4319 | MicroRNA 4319 | RNA Gene |  | 12 | GC18M044970 | 0.228644356131554 | https://www.genecards.org/cgi-bin/carddisp.pl?gene=MIR4319 |  |
