# Supplementary material for: Ceramide‐mediated mitochondrial dysfunction in nonobese nonalcoholic fatty liver disease: A regulatory role for serine palmitoyltransferase subunit 2
Source: J Cell Commun Signal. 2026 Jun 25;20(2):e70091. doi: 10.1002/ccs3.70091 (PMC13295144; doi:10.1002/ccs3.70091)

Figure S1-1

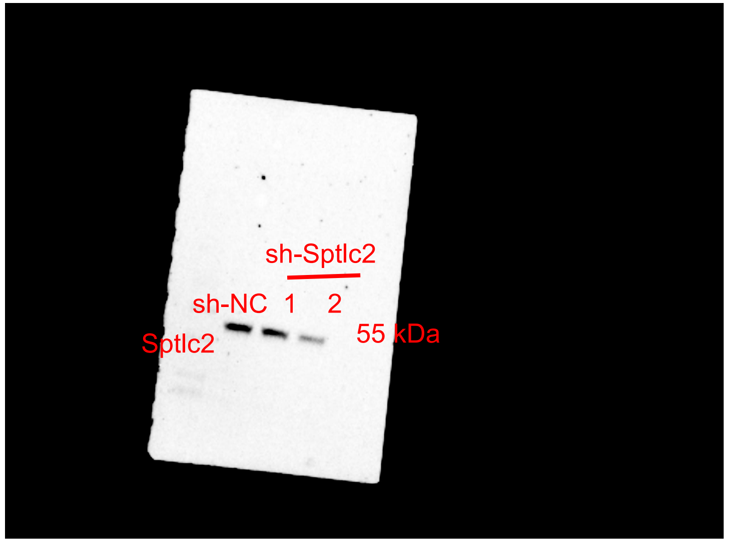

Figure S1-2

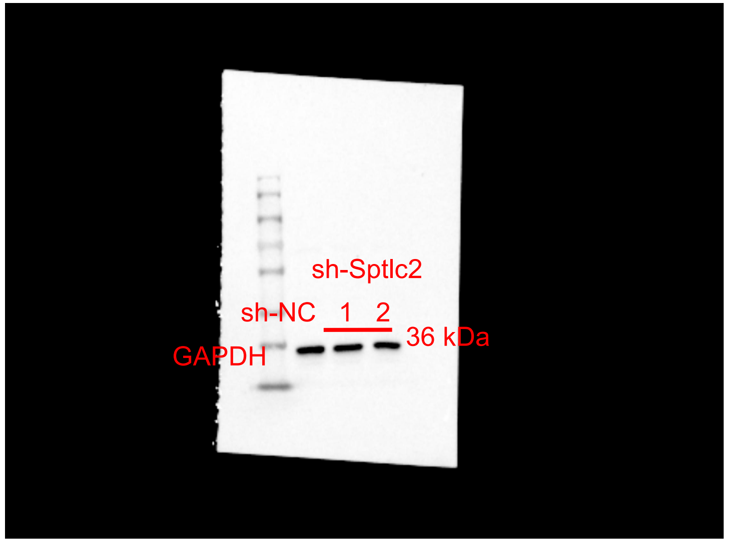

Figure 3B-1

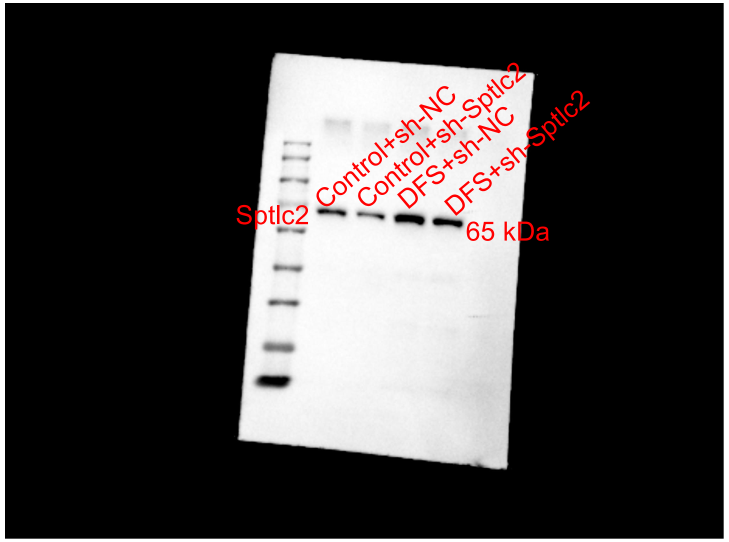

Figure 3B-2

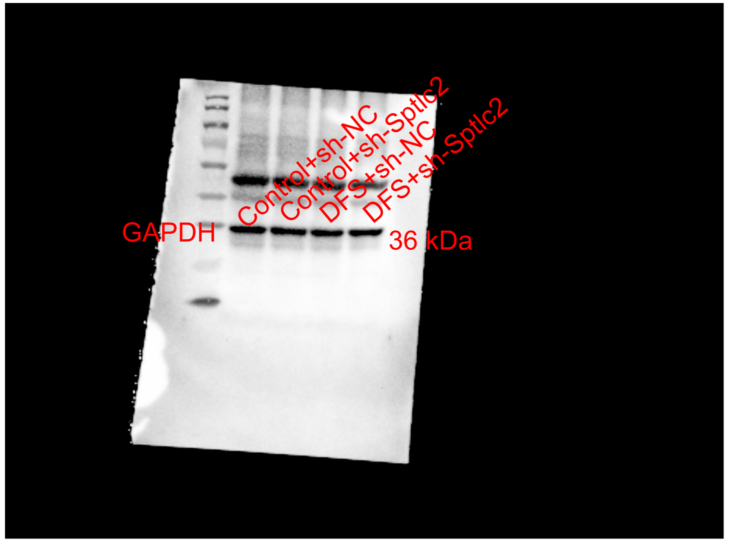

Figure 3H-1

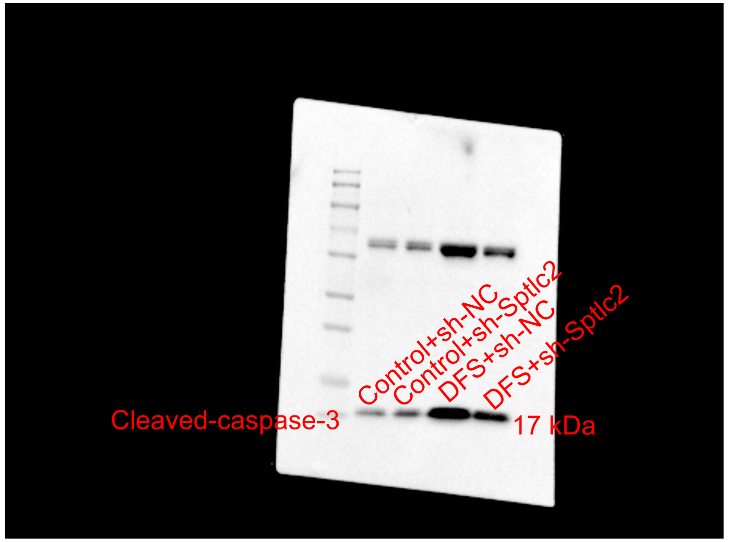

Figure 3H-2

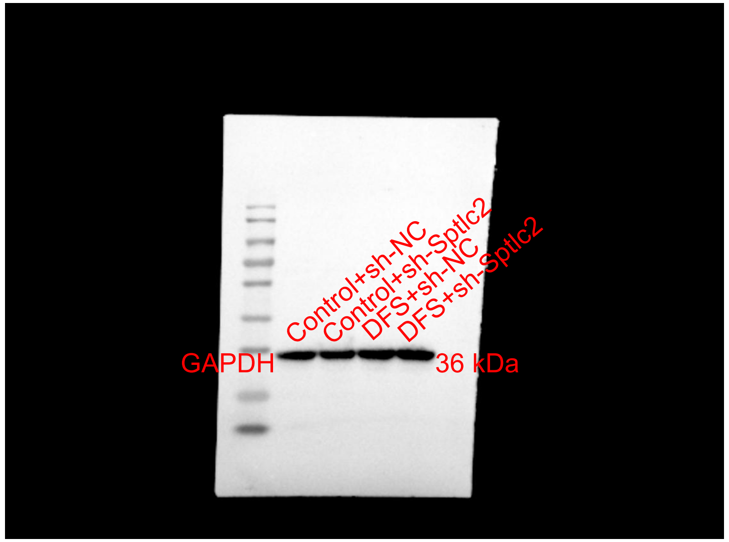

Figure 5K-1

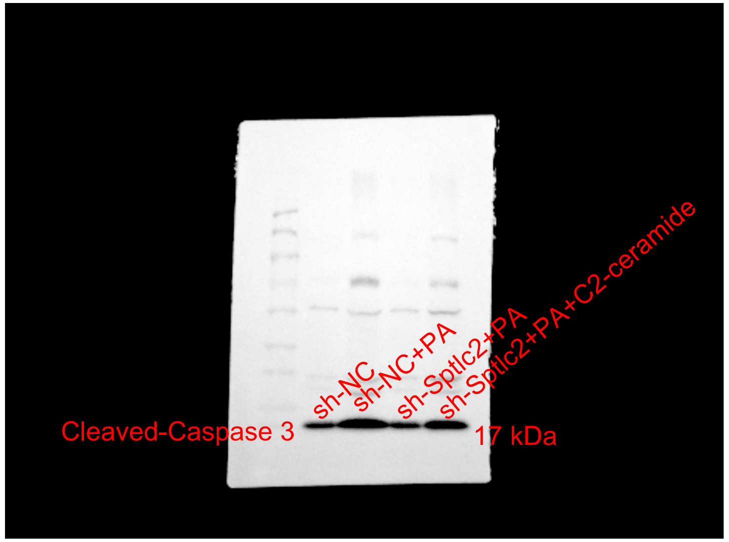

Figure 5K-2

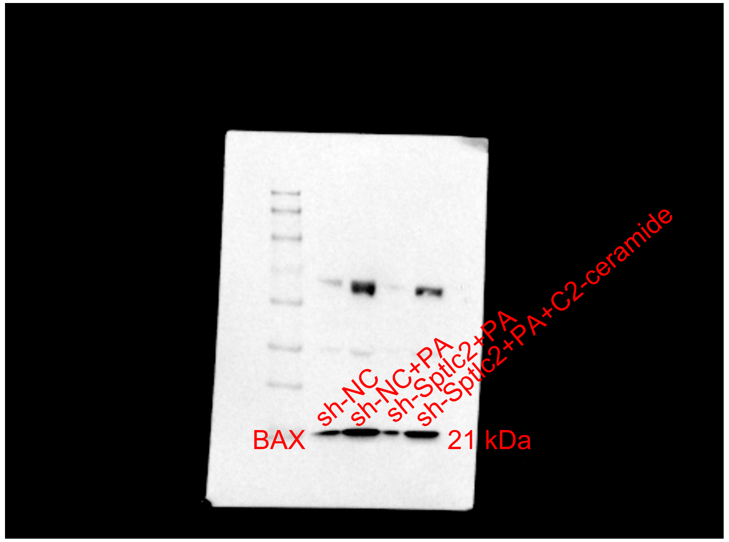

Figure 5K-3

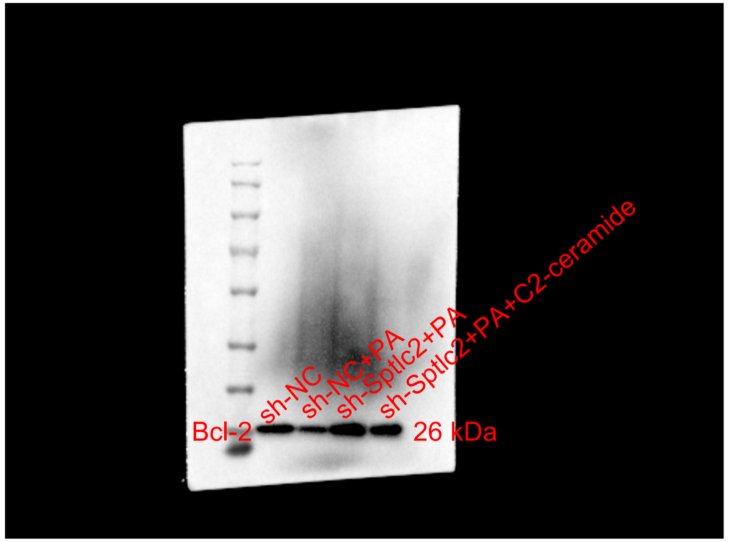

Figure 5K-4

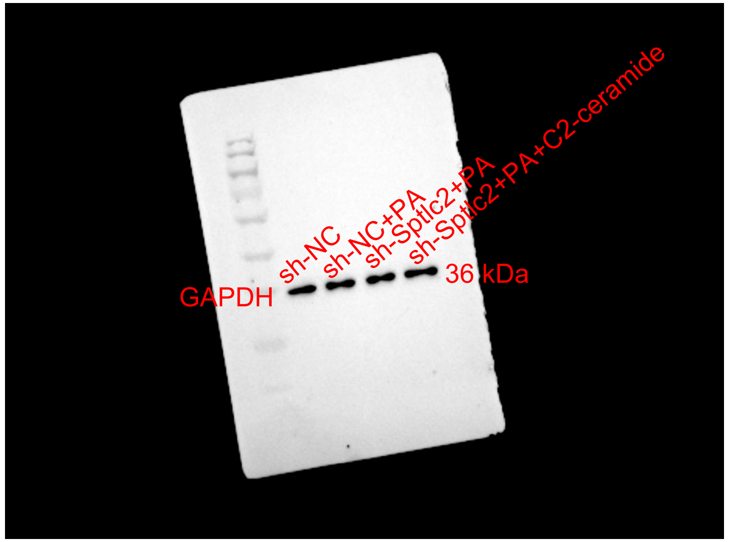

Figure 6H-1

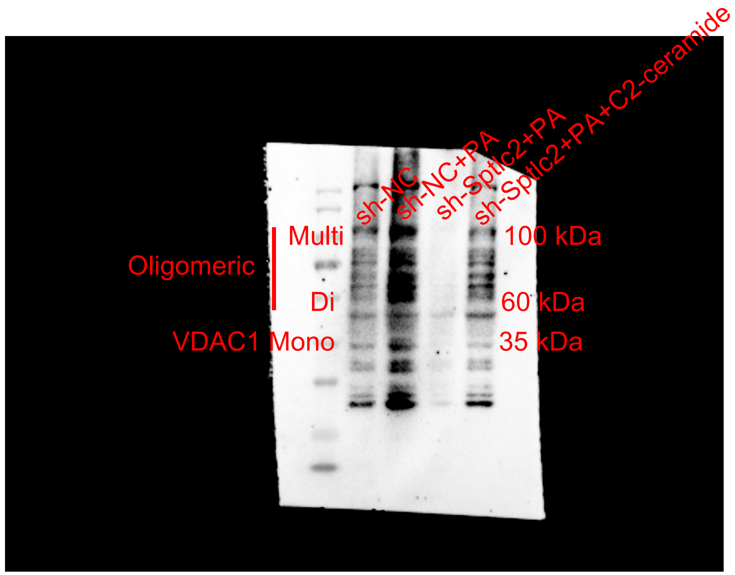

Figure 6H-4

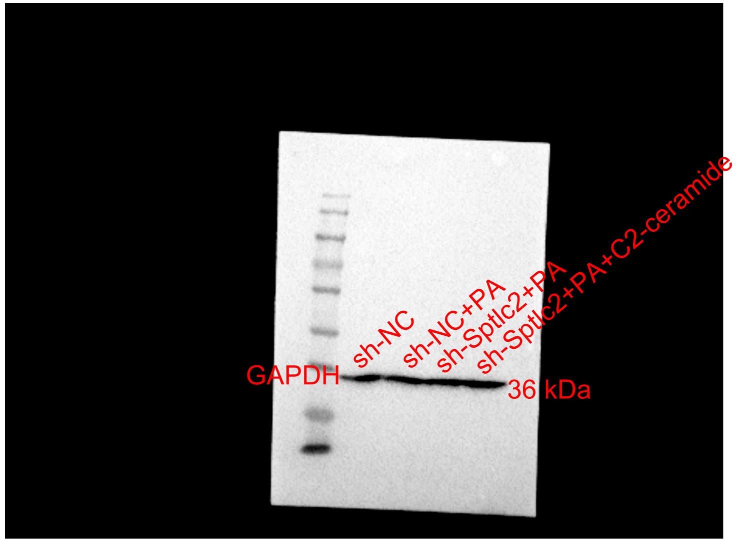

Figure 7D-1

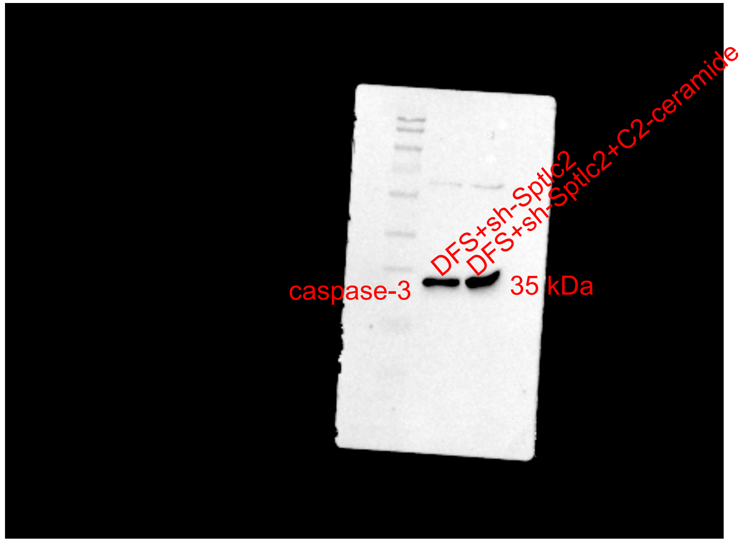

Figure 7D-2

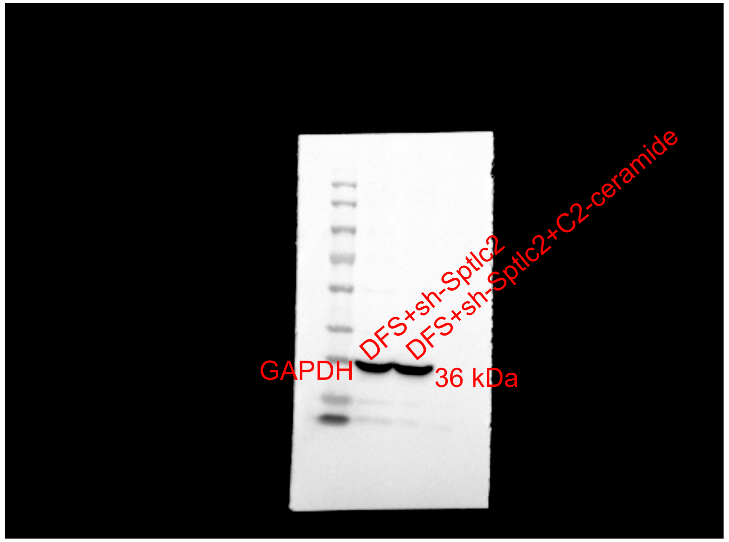

Supplement: Supplementary file 2 — Supporting Information S2 [file CCS3-20-e70091-s002.pdf]
